# Ylide‐Stabilized Phosphenium Cations: Impact of the Substitution Pattern on the Coordination Chemistry

**DOI:** 10.1002/chem.202104074

**Published:** 2022-01-05

**Authors:** Tobias Stalder, Felix Krischer, Henning Steinert, Philipp Neigenfind, Viktoria H. Gessner

**Affiliations:** ^1^ Chair of Inorganic Chemistry II Faculty of Chemistry and Biochemistry Ruhr-University Bochum Universitätsstraße 150 44801 Bochum Germany

**Keywords:** low-valent compounds, main group chemistry, phosphorus, structure elucidation, ylides

## Abstract

Although N‐heterocyclic phosphenium (NHP) cations have received considerable research interest due to their application in organocatalysis, including asymmetric synthesis, phosphenium cations with other substitution patterns have hardly been explored. Herein, the preparation of a series of ylide‐substituted cations of type [YPR]^+^ (with Y=Ph_3_PC(Ph), R=Ph, Cy or Y) and their structural and coordination properties are reported. Although the diylide‐substituted cation forms spontaneous from the chlorophosphine precursor, the monoylidylphosphenium ions required the addition of a halide‐abstraction reagent. The molecular structures of the cations reflected the different degrees of electron donation from the ylide to the phosphorus center depending on the second substituent. Molecular orbital analysis confirmed the stronger donor properties of the ylide systems compared to NHPs with the mono‐ylide substituted cations featuring a more pronounced electrophilicity. This was mirrored by the reaction of the cations towards gold chloride, in which only the diylide‐substituted cation [Y_2_P]^+^ formed the expected LAuCl]^+^ complex, while the monoylide‐substituted compounds reacted to the chlorophosphine ligands by transfer of the chloride from gold to the phosphorus center. These results demonstrate the tunability of ylide‐functionalized phosphorus cations, which should allow for further applications in coordination chemistry in the future.

## Graphical abstract

**Switching selectivity**: The isolation and coordination chemistry of a series of ylide‐substituted phosphenium cations is reported. The monoylidyl cations exhibit a distinctly higher reactivity and electrophilicity, thus yielding a chlorophosphine gold cation via chloride shift from gold to phosphorus. In contrast, the diylidyl cation formed the phosphenium AuCl complex, demonstrating the tunability of the phosphenium cation reactivity.

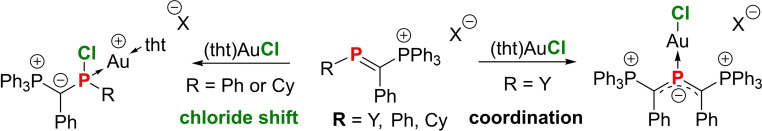

## Introduction

Phosphenium cations are compounds of type R_2_P^+^ and hence valence isoelectronic to carbenes.^[1]^ However, owing to their positive charge phosphenium ions usually exhibit a weaker σ‐donor strength but increased π‐acceptor abilities.^[2]^ These electronic properties make phosphenium cations attractive ligands for metal complexation which might be used complementarily to stable singlet carbenes, such as N‐heterocyclic carbenes (NHCs), which are in general strong donor ligands. Despite many advances made in the past years in phosphenium cation chemistry, their properties and coordination chemistry are far less explored than those of their carbon analogues. The limitation is mostly due to the higher reactivity connected with their cationic nature. Thus, only few phosphenium cations have been isolated and thoroughly explored. The most extensively studied systems are – analogous to carbenes^[3]^ – *N*‐heterocyclic phosphenium cations (NHPs). Since the first reports on NHPs in 1970s^[4]^ various derivatives^[5]^ and metal complexes have been reported[^6^, ^7^] and more recently also applied in catalysis including asymmetric organocatalysis.^[8]^

**Figure 1:**
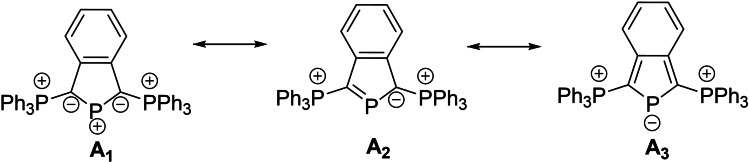
Diylidephosphenium cation reported by Schmidpeter and its different mesomeric structures.

Apart from amino groups also other π‐donor substituents have been employed to stabilize these carbene‐like species. In the 1990s Schmidpeter pioneered the use of ylide groups in phosphenium cation chemistry. He realized that depending on the second substituent R, ylide‐substituted chlorophosphines of type Y(R)PCl (Y=ylide) can spontaneously dissociate into the phosphenium cations.[^9^, ^10^] Diylide‐substituted cations are particularly stable due to the strong π‐donation from both ylide groups resulting in an efficient charge delocalization in the C−P−C linkage. In case of the cyclic cation **A** this delocalization even results in a partial phosphide character at the central P atom (**A_3_**, Figure 1).^[11]^ Nonetheless, these systems exhibit phosphenium cation reactivity and for example react with nucleophiles such as organolithium compounds at the central phosphorus atom.^[12]^ However, the coordination chemistry of ylidephosphenium cations remains almost unexplored. To the best of our knowledge, only the coordination chemistry of bis‐phosphonio‐isophosphindolides of type **A** has been explored.^[13]^

In the course of our research program on ylidic compounds we became interested in the stabilization of reactive main group compounds.^[14]^ With the isolation of metalated ylides as versatile reagents for ylide‐functionalization we were able to generate the first diylide‐substituted boron cations^[15]^ and heavier tetrylenes.^[16]^ Furthermore, the introduction of ylide substituents in phosphines led to a remarkable increase of their donor capacity and hence to excellent performance in gold^[17]^ and palladium catalysis.^[18]^ This led us to re‐investigate the chemistry of ylide‐substituted phosphenium cations to evaluate their potential as ligands in transition metal chemistry.

## Results and Discussion

### Synthesis of the phosphenium cations

**Scheme 1:**
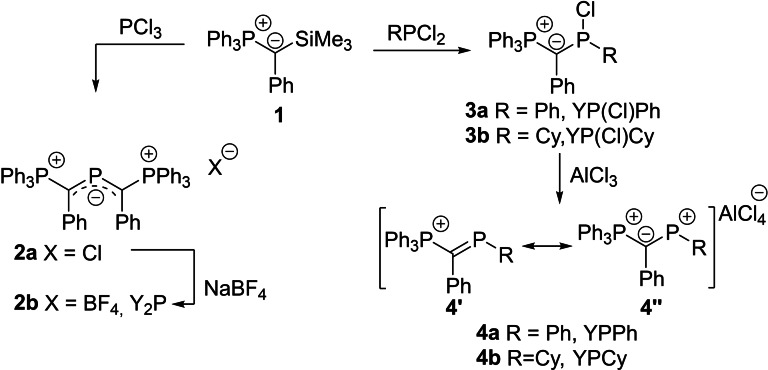
Synthesis of the phosphenium cations **2** and **4** from the silyl‐substituted ylide **1**.

**Figure 2:**
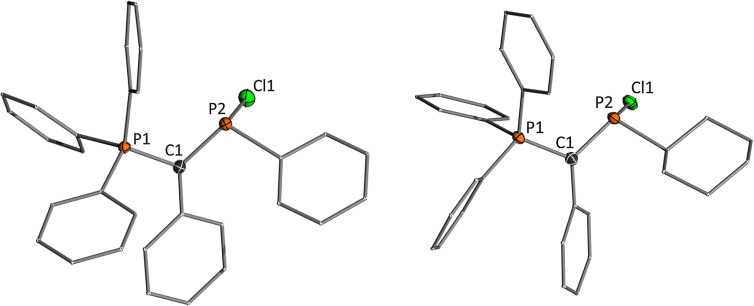
Molecular structure of 3a and 3b. Hydrogen atoms are omitted for clarity. Ellipsoids are shown at the 50 % probability level. Selected bond lengths (Å) and angles (°): **3 a**: P1−C1 1.7426(19), C1−P2 1.7424(19), P2−Cl1 2.1914(6), P1−C1−C2 119.91(14), P1−C1−P2 114.00(8), C2−C1−P2 111.73(10), C1−P2−Cl1 107.13(7), Cl1−P2−C26 97.79(6), C1−P2−C26 106.60(9). **3 b**: P1−C1 1.7359(14), C1−P2 1.7382(14), P2−Cl1 2.2560(5), P1−C1−C2 117.54(10), P1−C1−P2 114.00(8), C2−C1−P2 126.93(10), C1−P2−Cl1 108.57(5), Cl1−P2−C26 94.18(5), C1−P2−C26 104.20(7).

We began our studies with the synthesis of the diylidylphosphenium cation **2 a** reported by Schmidpeter.^[11a]^ To further explore the impact of the substitution pattern also the isolation of the monoylide cations **4** was targeted. With only one π‐donating ylide substituent, we expected these phosphenium cations to be more reactive and stronger acceptor ligands. All three ligands were prepared via a chlorosilane elimination protocol by reaction of the silyl‐substituted ylide **1** with PCl_3_ and the dichlorophosphines (PhPCl_2_ or CyPCl_2_), respectively (Scheme 1, see the Supporting Information). Whereas the reaction with PCl_3_ to form the diylide‐substituted compound directly led to the formation of cation **2** by chloride elimination, the monoylidyl compounds formed stable chlorophosphines **3a** and **3b**. In case of the diylide‐substituted compound chloride exchange to the BF_4_ salt was conducted to exclude any weak interaction of the counter‐anion with the phosphorus cation. The chlorophosphines **3** were isolated as colorless solids in 85 and 81 % yield and are characterized by two doublets in the ^31^P{^1^H} NMR spectrum appearing at approx. 24 ppm for the PPh_3_ group and at 132 ppm (for **3a**) and at 160 ppm (for **3b**), respectively. In the molecular structures (Figure 2), both chlorophosphines feature remarkably different P−Cl bond lengths. As a consequence of the stronger donor properties of the cyclohexyl substituent, **3b** (2.256(1) A) shows a 55 pm longer P−Cl bond than **3a** (2.191(1) A). Such varying P−X distances have been discussed in the past as snapshots of the pending dissociation.^[19]^

**Figure 3:**
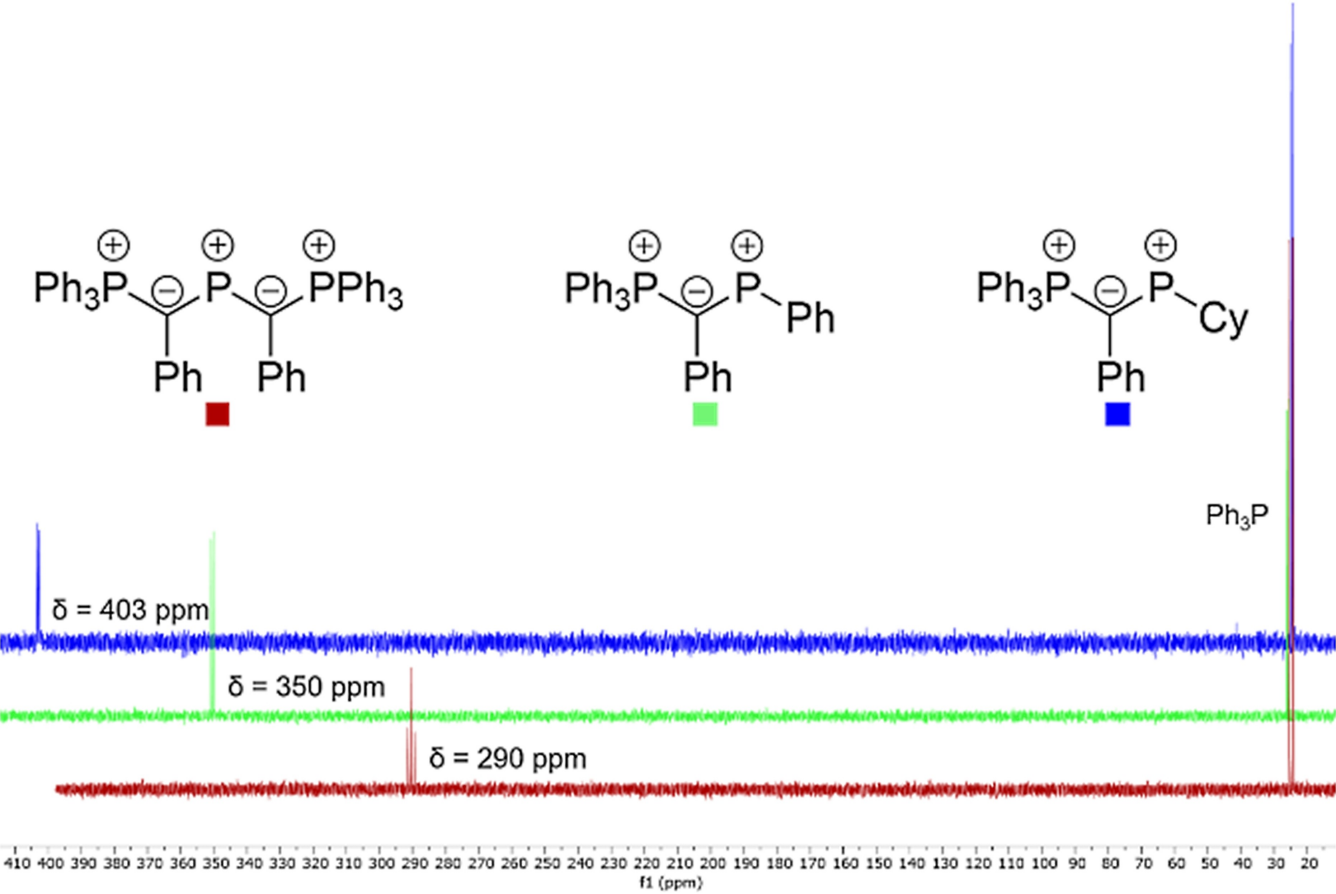
^31^P NMR shifts of the ylide‐substituted phosphenium cations.

With the chlorophosphines in hand, we next addressed their conversion into the corresponding phosphenium cations. Chloride abstraction from **3** was accomplished with AlCl_3_, thus delivering the cations **4** as colorless solids in good yields (72 for **4a**, 73 for **4b**).^[20]^ The cations **2** and **4** were characterized by multi‐nuclear NMR spectroscopy, elemental and single‐crystal X‐ray diffraction analysis. ^31^P{^1^H} NMR spectroscopy shows that the cyclohexyl‐substituted cation **4b** features the most deshielded signal (Figure 3, Table 1), which resonates at 403.1 ppm and hence is considerably downfield shifted compared to **4a**, **2**, NHPs and acyclic aminophosphenium cations.^[21]^ This suggests that **4b** exhibits the smallest HOMO‐LUMO gap in this series of compounds (see below for discussion) and presumably is the most nucleophilic species with the strongest acceptor properties. Interestingly, the ^2^ *J*_PP_ coupling constants increase from **4b** to **4a** and **2** and become significantly smaller compared to the chlorophosphine precursors. Overall, the mono‐ylidylphosphenium cations exhibited are considerably higher reactivities and more readily decomposed in solution. However, in the solid state they could be stored for weeks at −20 °C.

**Table 1:** Crystallographic and NMR spectroscopic data of the phosphorus cations **2** and **4** and their gold complexes.

Compound	Crystallographic data					NMR spectroscopic data		
	C1‐P1 [Å]	C1‐P2 [Å]	P1‐C1‐P2 [°]	δ(P1) [ppm]	δ(P2) [ppm]	^2^ *J*_PP_ [Hz]	δ(C1) [ppm]	^1^ *J*_PC_ [Hz]
YP(Cl)Ph (**3 a**)	1.7426(19)	1.7424(19)	114.00(8)	24.2 (d)	131.8 (d)	191.5	51.4	103.4, 58.5
YP(Cl)Cy (**3 b**)	1.7359(14)	1.7382(14)	114.00(8)	23.5 (d)	160.3 (d)	166.1	50.7	104.6, 57.8
[Y_2_P]BF_4_ (**2 b**)^[a]^	1.7545(14)	1.718(1)	112.85(8)	24.7 (d)	290.4 (t)	165.3	97.7	91.5, 72.0
[YPPh]AlCl_4_ (**4 a**)	1.807(5)	1.673(5)	115.4(3)	25.7 (d)	350.9 (d)	123.7	165.1	66.8, 62.9
[YPCy]AlCl_4_ (**4 b**)	1.797(2)	1.688(2)	115.93(11)	24.8 (d)	403.1 (d)	100.4	169.0	69.6, 62.7
[Y_2_P⋅AuCl]BF_4_ (**5 b**)	1.764(3)	1.691(3)	119.36(15)	26.0 (d)	229.2 (t)	98.9	86.1	93.6, 50.4
YP(Cl)Cy⋅AuCl (**7 b**)	1.736(4)	1.714(4)	121.8(2)	24.5 (d)	107.5 (d)	77.0	42.0	111.9, 55.1
[YP(Cl)Cy⋅Au(tht)]AlCl_4_ (**6 b**)	–	–	–	24.8 (d)	106.6 (d)	73.2	41.8	110.9, 54.9
YP(Cl)Ph⋅AuCl (**7 a**)	1.728(4)	1.713(5)	120.1(3)	25.0 (d)	83.5 (d)	98.5	44.9	112.4, 67.8
[YP(Cl)Ph⋅Au(tht)]AlCl_4_ (**6 a**)	1.734(7)	1.700(7)	123.9(4)	25.1 (d)	83.6 (d)	93.3	44.8	111.5, 62.8

[a] Average values of both ylide substituents **: values of the coordinated site.

**Figure 4:**
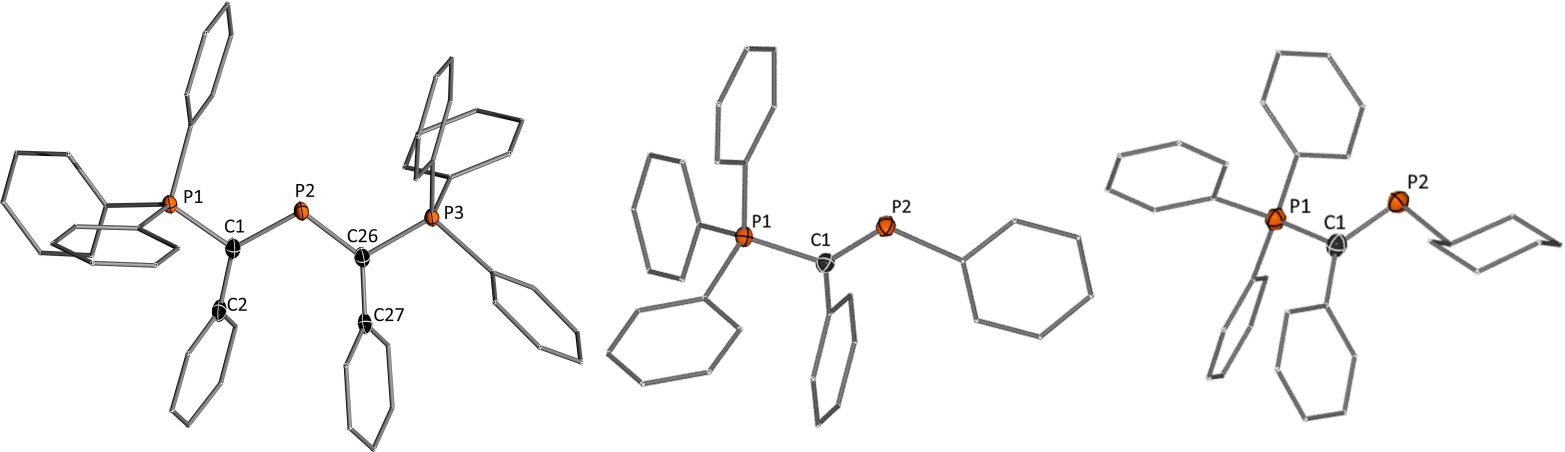
Molecular structure of (left) [Y_2_P]BF_4_ (**2 b)**, (middle) [YPPh]AlCl_4_ (**4 a**) and (right) [YPCy]AlCl_4_ (**4 b**). Hydrogen atoms, counter anions and solvent molecules are omitted for clarity. Ellipsoids are shown at the 50 % probability level. Selected bond lengths (Å) and angles (°): **2 b**: P1−C1 1.7523(14), P2−C1 1.7216(14), C26−P2 1.7141(14), C26−P3 1.7566(14), P1−C1−P2 113.34(8), P1−C1−C2 116.15(10), C2−C1−P2 130.51(10), C1−P2−C26 111.91(7), P3−C26−P2 112.36(8), P3−C26−C27 116.70(10), C27−C26−P2 130.93(10). **4 a**: P1−C1 1.807(5), C1−P2 1.673(5), C2−C1−P1 116.1(3), C2−C1−P2 128.4(4), P2−C1−P1 115.4(3), C1−P2−C26 108.4(3). **4 c**: P1−C1 1.797(2), C1−P2 1.688(2), C2−C1−P1 118.22(14), C2−C1−P2 125.79(14), P2−C1−P1 115.93(11), C1−P2−C26 104.58(9).

Single crystals for **2b**, **4a** and **4b** with BF_4_^−^ or AlCl_4_^−^ as counter‐anions were obtained by layering DCM solutions of the cations with cyclohexane.^[22]^ All structures are depicted in Figure 4, important structure parameters are listed in Table 1. The molecular structures clearly reflect the different stabilizing abilities of the second substituent at P2 and its impact on the π‐donation from the ylide substituent. Due to the competing π‐interaction by both ylide groups in **2b**, the diylide cation features the longest C1−P2 distance of approx. 1.72 Å. The P2−C1 bonds in the monoylide phosphenium cations are considerably shorter, with the phenyl compound featuring the shortest distance of 1.673(5) Å. The reverse trend is found for the P1−C1 distances to the phosphonium group. With increasing π‐bonding between the ylide and the phosphenium center the electrostatic attraction to the phosphonium group decreases. Consistent with the C1‐P2 bonds, this results in the shortest C1‐P1 bond for **2** and the longest in the phenyl‐substituted compound, suggesting that the strongest ylide‐P bond is found in cation **4a**. It is noteworthy that the phenyl substituent in **4a** is not or only little involved in π‐interaction with the central phosphorus atom. The same is true for the phenyl groups in the ylide‐backbone. While the two ylide groups in **2b** form a fully delocalized π‐system over the planar P−C−P−C‐P linkage, the phenyl group in **4 a** is oriented out of the P1−C1−P2 plane with dihedral angles of approx. 20°. Consequently, no changes in the C−C bond lengths are observed within the phenyl groups of **4a** compared to that in **3a**, which contrasts for example the quinoidal structure reported for the borinium cation Mes_2_B^+^.^[23]^ It is also noteworthy, that in contrast to some ylide‐substituted phosphines only one conformational isomer of **2** and **4** are observed in the solid‐state and in solution.^[17b]^ In these isomers, the lone pair at phosphorus is perpendicularly arranged to the lone pair at the ylidic carbon atom in order to minimize electronic repulsion and enable π‐stabilisation of the empty p orbital at P2. Due to additional steric effects an anti‐/zigzag arrangement is favored, which in case of **2** leads to the conformer with the smaller phenyl groups facing each other.

**Figure 5:**
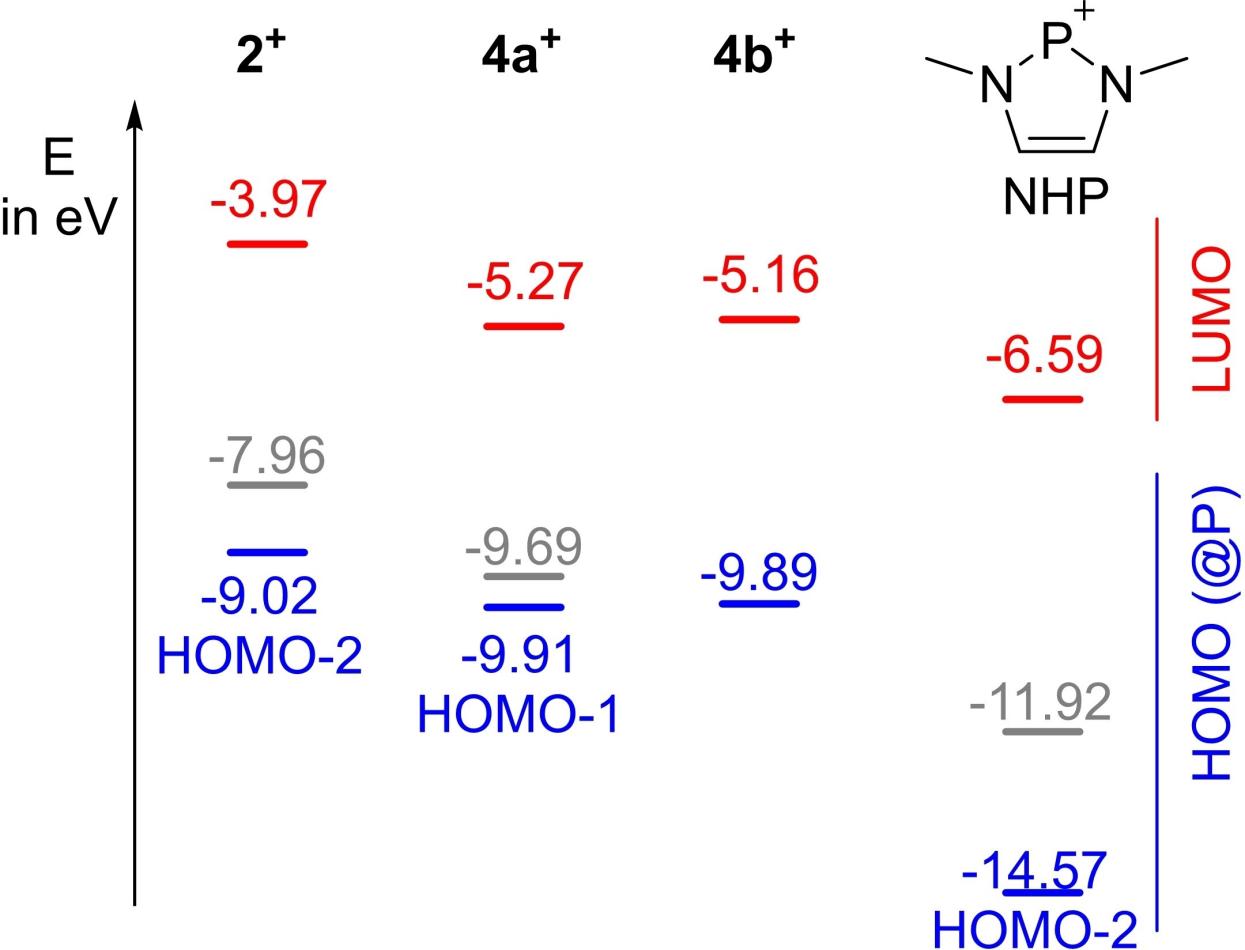
Calculated HOMO–LUMO energies of the different phosphenium cations.

To shed light on the electronic structure and the observed spectroscopic properties of the synthesized cations we performed computational studies on the PW6B95D3/def2tzvp level of theory (see the Supporting Information for details). Calculations of the molecular orbitals show that the ylide‐substituted phosphenium cations feature energetically high‐lying LUMOs (LUMO=lowest occupied molecular orbital) and hence are less electrophilic/weaker acceptors than NHPs (Figure 5). This agrees well with the stronger π‐donor ability of the ylide compared to the amino substituent. Accordingly, the diylidyl cation **2^+^** is the by far weakest acceptor in this series of compounds. While the LUMO in all cations represents the empty p orbital at P2 and thus is always predominantly localized at the phosphenium center, the HOMO (except for **4b**) displays a π‐symmetric orbital delocalized over the substituents with a strong contribution by the ylidic carbon centers. In general, the energy of the molecular orbitals representing the lone pair at the phosphenium center (shown in blue in Figure 5) only vary slightly in the ylide‐substituted cations but are again significantly higher than those of NHPs. This clearly demonstrates that ylide‐substituted phosphenium ions are particularly strong donor, but weaker acceptor ligands.

Overall, the spectroscopic and computational data clearly indicate that the diylide‐substituted phosphenium cation **2b** is electronically quite different from the monoylide congeners. It is considerably more nucleophilic as expressed by its resonance structure shown in Scheme 1 with a negative formal charge at phosphorus, whereas cations **4a** and **4b** retain a higher acceptor ability. Thus, the monoylide cations are best described by the two canonical structures **4’** and **4’’** (Scheme 1). Nonetheless, also these monoylide cations are considerably stronger donors than NHPs and thus should exhibit different donor properties.

**Scheme 2:**
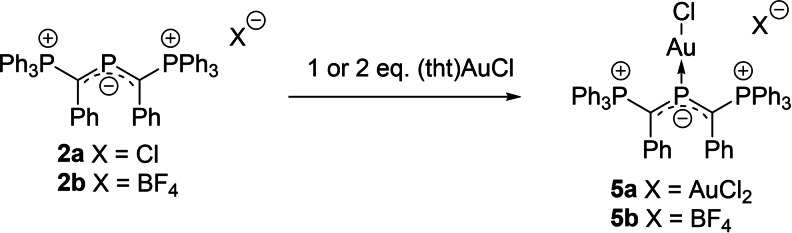
Synthesis of the gold complexes **5** from the diylide‐substituted cation **2**.

**Figure 6:**
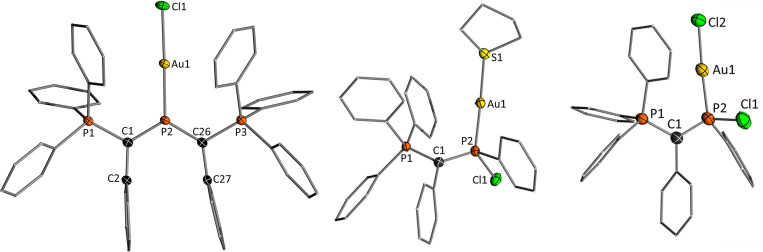
Molecular structures of [Y_2_P⋅AuCl]BF_4_, [YP(Cl)Ph⋅Au(tht)]AlCl_4_ and YP(Cl)Ph⋅AuCl. The anion, hydrogen atoms and solvent molecules are omitted for clarity. Ellipsoids are shown at the 50 % probability level. Selected bond lengths (Å) and angles (°): [Y_2_P⋅AuCl]BF_4_: P1−C1 1.768(3), P3−C26 1.758(3), P2−C1 1.692(3), P2−C26 1.691(3), Au1−P2 2.2127(6), Au1−Cl1 2.2777(7), C2−C1−P2 127.98(19), C2−C1−P1 113.18(18), P2−C1−P1 118.81(15), C27−C26−P2 123.47(19), C27−C26−P3 116.51(18), P2−C26−P3 119.87(15), C1−P2−C26 117.92(13), C1−P2−Au1 121.58(9), C26−P2−Au1 120.0(9), P2−Au1−Cl1 178.78(2). [YPPh⋅Au(tht)]AlCl_4_: P1−C1 1.734(7), P2−C1 1.700(7), Cl1−P2 2.111(2), Au1−P2 2.2601(16), Au1−S1 2.3182(15), C2−C1−P2 120.5(5), C2−C1−P1 115.4(5), P2−C1−P1 123.9(4), P2−Au1−S1 177.90(6). YP(Cl)Ph⋅AuCl: P1−C1 1.728(4), P2−C1 1.713(5), Cl1−P2 2.1111(16), Au1−P2 2.2273(11), Au1−Cl2 2.2873(11), C2−C1−P2 121.6(3), C2−C1−P1 117.9(3), P2−C1−P1 120.1(3), P2−Au1−Cl2 176.72(4).

Next, we turned our attention toward the coordination chemistry of the phosphenium cations. We first focused on the synthesis of gold complexes since complexes with cationic phosphorus ligands have shown catalytic activity.^[24]^ Treatment of **2b** with one equivalent of (tht)AuCl (tht=tetrahydrothiophene) led to the clean formation of the expected gold chloride complex [**2b**‐AuCl]BF_4_ (**5b**; Scheme 2). Using an excess of gold precursor and the chloride salt of the phosphenium ligand **2a** led to the same complex, yet with AuCl_2_ as counter anion (**5a**), which was found to weakly bind to the ylidic P−C bond in the solid state. A similar observation was made by Gudat with the cyclic diylide phosphenium cation **A** (see the Supporting Information for details).^[13a]^ **5b** is characterized by a highfield shift in the ^31^P{^1^H} NMR spectrum for P2 from 290.4 in **2b** to 229.2 ppm in **5b**. The molecular structure (Figure 6) features a linear P−Au−Cl linkage with a P−Au bond length of 2.2127(6) A. This bond length is comparable to other phosphido‐gold complexes.[^13a^, ^25^] However, in contrast to previously reported complexes, the central phosphorus atom adopts a completely planar geometry with a sum of angles of 360°. The P2‐C1 bond shortens from 1.718(1) Å to 1.692(3) Å upon coordination to gold as a consequence of the further shift of electron density from the ylide groups to the phosphenium center.

**Scheme 3:**
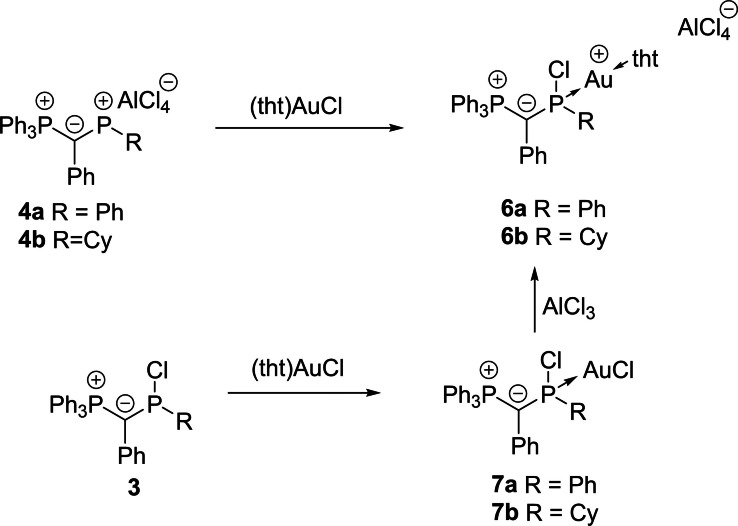
Synthesis of the cationic gold complexes **6** from the chlorophosphine ligands **3** or cations **4**.

Interestingly, the monoylide‐substituted phosphenium cations **4** showed a different coordination behavior towards gold chloride as suggested by the different orbital energies. This was already indicated by the NMR spectroscopic data. Upon treatment of **4a** and **4b**, respectively, with one equivalent (tht)AuCl, the signal of the central phosphorus atom experienced a distinct high field shift by more than 250 ppm in the ^31^P{^1^H} NMR spectrum, giving rise to a signal at 93.3 ppm for **4a** and 73.2 ppm for **4b**, respectively. This shift is much more pronounced than in case of **2 b**, suggesting considerable changes in the ligand structure. This was confirmed by XRD analysis of the complex formed with **4a**, which revealed the formation of complex **6a** (Scheme 3), in which a cationic gold center is coordinated by the chlorophosphine ligand **3a** and tht. This structure matches well with the NMR spectroscopic observations.

**6a** is presumably formed from the expected AuCl complex by chloride shift from gold to phosphorus. Thus, this gold complex should also be accessible from a simple AuCl complex of the chlorophosphines precursors **3**. Indeed, treatment of **3a** and **3b**, respectively, with (tht)AuCl delivered the corresponding gold chloride complexes **7**, which could both be isolated in moderate yields of approx. 40 % and unambiguously characterized, including XRD analysis (Figure 6 and the Supporting Information). Treatment of the gold chloride complexes with aluminium trichloride finally yielded the cationic complexes as judged by ^31^P NMR spectroscopy (Scheme 3). Using an excess of AlCl_3_ (or NaBAr^F^) did not lead to the abstraction of the chloride at the phosphorus center in **6** to form the corresponding dication. The same holds true for the cationic gold complex **5b**.

Single crystals for **6a** could be obtained by slow vapor diffusion of pentane into a saturated dichloromethane solution. In the crystal (Figure 6), the gold center adopts a linear geometry between the phosphine and tetrahydrothiophene ligand with an Au−P distance of 2.260(2) A. This bond length is slightly longer than the one found in **5b** and also longer than the one in the AuCl precursor **7a**. The P2−C1 bond shortens in the compound series **3a**>**7a**>**6a** from 1.742(2) to 1.700(7) Å, thus accounting for an increasing shift of electron density from the ylidic carbon atom to the phosphorus (and metal) center. The C1‐P1 distance increases in the same sequence of compounds.

An interesting combining structural feature of all gold complexes shown in Figure 6 are short distances between the gold center and one phenyl group of the phosphonium moiety. A survey (Table 2) of all structures revealed that the Au‐C_ipso_ distance is shorter in the cationic gold complex **6a** (3.160 Å) than in the neutral AuCl complexes **7** which exhibits an Au−C_ipso_ distance of 3.220 Å. Also, the cationic Au complex **5b** with the diylidylphosphenium ligand shows a short distance of 3.132 Å. Such short distances have been attributed to attractive arene gold interactions and have often been discussed in the context of gold catalysis and their importance for stabilizing catalytically active cationic gold species.^[26]^ Prominent examples are the Echavarren's catalysts with Buchwald's biarylphosphine JohnPhos.^[27]^ Here, an arene Au distance of 3.04 Å was reported in the gold cation which is slightly shorter than the one in **6a** thus indicating a weak attractive interaction. It is important to note that such interactions have also been suggested for gold complexes of ylide‐substituted phosphines (YPhos) and thought to be one reason for their high catalytic efficiency.[^17^, ^18d^] However, no cationic Au‐YPhos complex has been isolated until today. Thus, **6a** constitutes the first complex of its kind.

**Table 2:** Bond length of the shortest Au–arene interaction in the discussed gold complexes.

	**5 b**	**6 a**	**7 a**	**7 b**
Au‐C_ipso_	3.132	3.160	3.220	3.371

**Figure 7:**
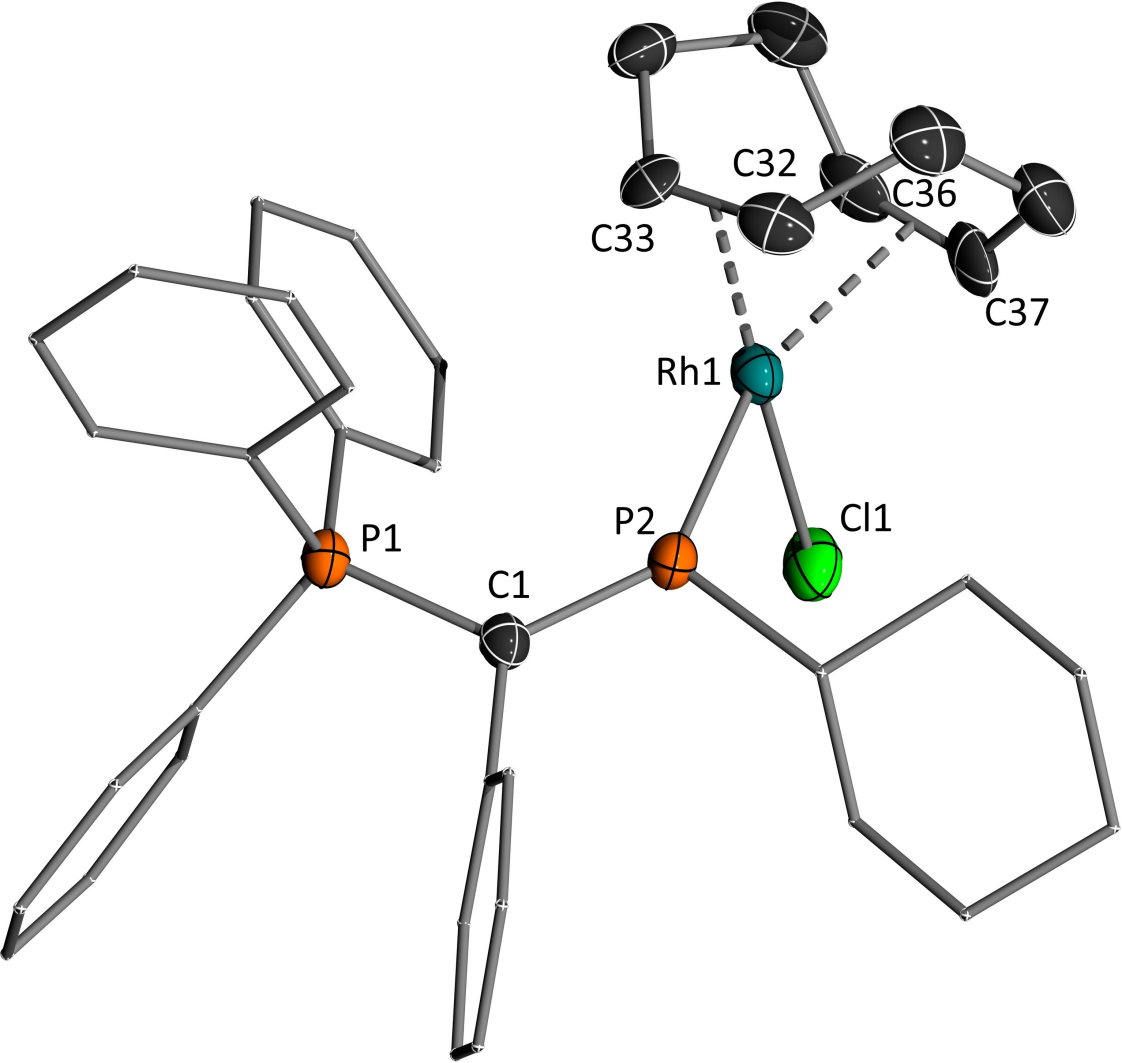
Molecular structure of [**4 b**⋅Rh(COD)Cl]AlCl_4_. Hydrogen atoms and counter anion are omitted for clarity. Ellipsoids are shown at the 50 % probability level. Selected bond lengths (Å) and angles (°): C1−P1 1.797(6), C1−P2 1.676(6), Rh1−P2 2.2165(14), Rh1−Cl1 2.3737(14), C2−C1−P2 123.2(4), C2−C1−P1 114.2(4), P2−C1−P1 122.4(3), C1−P2−C26 107.0(3), C1−P2−Rh1 137.4(2), C26−P2−Rh1 115.4(2), P2−Rh1−Cl1 86.83(6).

Unfortunately, all attempts to apply the cationic gold complexes as catalysts in hydroaminations or enyne cyclization reactions remained unsuccessful. This can probably be attributed to the partial phosphide character and the resulting lower electrophilicity compared with other cationic gold species as well as the sensitivity of the complexes. To further investigate the coordination properties of the monoylide‐substituted phosphenium cations **4** and the chloride transfer from other transition metals we probed their reaction towards a series of late transition metal complexes. Although, the isolation of any reaction product revealed to be difficult due to the formation of further side products or the sensitivity of the formed complexes, NMR spectroscopic analyses of the reaction mixtures with [Pd(cod)Cl_2_] clearly indicated the formation of the corresponding chlorophosphines **3**, for example with two doublets at δ=78.4 and 17.4 ppm with a coupling constant of 9.2 Hz for **3a**. In the case of [Rh(cod)Cl]_2_ however, the formation of a phosphenium complex was observed as evidenced by a considerably downfield‐shifted signal in the ^31^P{^1^H} NMR spectrum for the P(III) center (δ=371.5 ppm, ^1^ *J*_RhP_=163.5 Hz and ^2^ *J*_PP_=52.5 Hz for **4b**). Although all attempts to isolate these complexes failed, single crystals were obtained from the crude reaction mixture of **4b** in CD_2_Cl_2_, which confirmed the still intact Rh−Cl bond (Figure 7). The Rh−P bond amounts to 2.217(4) A and is thus longer compared with the rhodium phosphorus bond of 2.089(2) Å reported for an NHP‐RhCl(PPh_3_)_2_ complex with a bis(alkylamido)naphthalene framework,^[28]^ and in the range of Rh−P bonds in phosphine complexes.^[29]^ Overall, the marked difference between the coordination chemistry of [Y_2_P]^+^ and the monoylide cations clearly shows that a certain stabilization of the cation is required for selective applications in transition metal chemistry. One ylide moiety as sole π‐donating substituent seems to be insufficient so that future research endeavors should focus on the diylidyl or monoylide systems with a further π‐donor substituent.

## Conclusion

The synthesis of three phosphorus cations stabilized by two or one ylide moiety is reported. Due to the strong electron‐donating ability of the ylide group, these formal phosphenium cations are considerably more electron‐rich than N‐heterocyclic phosphenium cations but can be tuned by variation of the second substituent bound to the phosphorus center. Thus, the bis(ylidyl)phosphenium cation Y_2_P^+^ forms spontaneously from the corresponding chlorophosphine in solution, whereas the mono(ylidyl)phosphenium ions are only accessible by halide abstraction. The stronger electrophilic nature of the mono‐ylide substituted compounds was also confirmed by computational studies and led to a different coordination chemistry compared to [Y_2_P]^+^. Although the bis(ylidyl) cation formed the expected cationic LAuCl complex, the monoylide systems reacted with (THT)AuCl by chloride transfer from gold to phosphorus, thus resulting in the formation of a cationic chlorophosphine gold complex. Crystallographic studies on the phosphenium cations and their metal complexes reflected the electron donation from the ylide to the phosphorus center. As such, the P−C distances in the ligand backbone changed depending on the second substituent at phosphorus and the presence of a metal center.

Overall, these studies clearly demonstrate that ylide groups efficiently stabilize cationic phosphorus species and allow a tuning of their properties by changing the molecular design. Although systematic studies on the impact of the substituents in the ylide backbone on the stability and reactivity of phosphenium cations are missing, it can be assumed that a further control of reactivity should be possible.

## Crystallographic Details

Deposition Number(s) 2121597 (for HYSiMe_3_I), 2121588 (for **1**), 2121592 (for **2[BF**_**4**_ **]**), 2121599 (for **3 a**), 2121593 (for **3 b**), 2121594 (for **4 a**), 2121596 (for **4 b**), 2121589 (for **5 a**), 2121600 (for **5 b**), 2121598 (for **7 a**), 2121595 (for **7 b**), 2121590 (for **6 a**), 2121591 (for [**4 b**⋅Rh(COD)Cl]AlCl_4_) contain(s) the supplementary crystallographic data for this paper. These data are provided free of charge by the joint Cambridge Crystallographic Data Centre and Fachinformationszentrum Karlsruhe Access Structures service.

## Conflict of interest

The authors declare no conflict of interest.

1

## Supporting information

As a service to our authors and readers, this journal provides supporting information supplied by the authors. Such materials are peer reviewed and may be re‐organized for online delivery, but are not copy‐edited or typeset. Technical support issues arising from supporting information (other than missing files) should be addressed to the authors.

Supporting Information

## Data Availability

The data that support the findings of this study are available from the corresponding author upon reasonable request.
