# Supplementary material for: Ylide‐Stabilized Phosphenium Cations: Impact of the Substitution Pattern on the Coordination Chemistry
Source: Chemistry. 2022 Jan 5;28(8):e202104074. doi: 10.1002/chem.202104074 (PMC9303317; doi:10.1002/chem.202104074)
Supplement: Supplementary file 1 — Supporting Information [file CHEM-28-0-s001.pdf]

Chemistry–A European Journal

Supporting Information

Ylide-Stabilized Phosphenium Cations: Impact of the Substitution Pattern on the Coordination Chemistry

Tobias Stalder, Felix Krischer, Henning Steinert, Philipp Neigenfind, and Viktoria H. Gessner*

Table of Contents

1 Experimental Procedures 3

1.1 General 3
1.2 Preparation of cation precursors 3
1.3 Preparation of the chlorophosphines and phosphenium cations 5
1.4 Preparation of metal complexes 8

2 NMR-Spectra 13

2.1 NMR spectra of the phosphenium cation precursors 13
2.2 NMR spectra of the chlorophosphines and phosphenium cations 18
2.3 NMR spectra of the gold complexes 28

3 Crystal structure analysis 39

3.1 General information 39
3.2 Crystal structure of $HYSiMe_3I$ 39
3.3 Crystal structure of 1. 43
3.4 Crystal structure of 2[BF_4] 47
3.5 Crystal structure of 3a 52
3.6 Crystal structure of 3b 56
3.7 Crystal structure of 4a 60
3.8 Crystal structure of 4b 64
3.9 Crystal structure of 5a 70
3.10 Crystal structure of 5b 76
3.11 Crystal structure of YPPhClAuCl 86
3.12 Crystal structure of YPCyClAuCl 90
3.13 Crystal structure of [YPPhClAu(tht)]$AlCl_4$ 94
3.14 Crystal structure of [YPCyRh(cod)Cl]$AlCl_4$ 98

4 Computational Studies 103

4.1 General remarks 103
4.2 Energies 103
4.3 Natural Charges and WBIs 104
4.4 Coordinates 104
4.5 Exemplary Input Files 112

5 References 113

Supporting Information

1 Experimental Procedures

1.1 General

<u>Chemicals and conditions:</u> If not stated otherwise, all experiments were carried out using standard Schlenk techniques under an argon atmosphere, which was dry and free of oxygen. Argon (99.999%) was a product of *Air Liquide* and was used without any further drying. An MBraun SPS 800 was used to dry solvents before their usage (THF, toluene, DCM, ACN, *n*-pentane, *n*-hexane). Cyclohexane was dried in accordance with standard procedures. All solvents were stored over molecular sieves under an argon atmosphere. Commercial substrates were used as received unless stated otherwise. PCl_3, $PPhCl_2$ and $PCyCl_2$ were freshly distilled, $AlCl_3$ and $(Et_2O)AlCl_3$ freshly sublimed before usage.

<u>NMR spectroscopy:</u> ^{1}H, $^{13}C\{^{1}H\}$, $^{31}P\{^{1}H\}$, $^{19}F\{^{1}H\}$, $^{11}B\{^{1}H\}$, $^{27}Al\{^{1}H\}$ and $^{29}Si\{^{1}H\}$-^{1}H NMR spectra were recorded on an Avance-400 or Avance-250 spectrometer at 25 °C if not stated otherwise. All values of the chemical shift are in ppm regarding the δ-scale. All spin-spin coupling constants (*J*) are printed in Hertz (Hz). To display multiplicities and signal forms correctly the following abbreviations were used: s = singlet, d = doublet, m = multiplet, br = broad signal. Signal assignment was supported by DEPT, APT, HSQC and HMBC experiments.

<u>Melting points</u> were measured with the SMP30 melting point apparatus from Stuart.

<u>Elemental analyses</u> were performed on an Elementar vario MICRO cube elemental analyzer.

1.2 Preparation of cation precursors

Preparation of benzyl(triphenyl)phosphonium bromide

(similar procedures were already reported in literature[1])

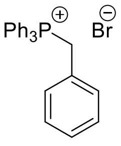

Triphenylphosphine (21.0 g, 80.1 mmol, 1.00 eq.) was dissolved in 200 ml toluene and benzylbromide (10.0 ml, 84.2 mmol, 1.05 eq.) was added quickly. The mixture was heated to 80 °C for 18 h. A colorless solid precipitated out of the reaction solution. The precipitate was filtered off, washed twice with 50 ml toluene and dried under reduced pressure. The product was obtained as a colorless solid (34.3 g, 79.1 mmol, 99%).

^{1}H NMR (400.3 MHz, $CDCl_3$) δ = 7.80-7.66 (m, 9 H), 7.66-7.56 (m, 6 H), 7.24-7.16 (m, 1 H), 7.15-7.05 (m, 4 H), 5.39 (d, $^{2}J_{PH}$ = 14.4 Hz, 2 H). **^{31}P{^{1}H} NMR** (162.1 MHz, $CDCl_3$) δ = 23.2.

The analytical and physical data were identical to those previously reported in the literature.[2]

Preparation of benzylidene(triphenyl)phosphorane

(similar procedures were already reported in literature[3])

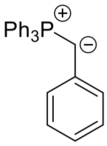

Procedure A (for small amounts): Benzyl(triphenyl)phosphonium bromide (2.00g, 4.62 mmol, 1.00 eq.) and KH (280 mg, 6.98 mmol, 1.50 eq.) were suspended in 20 ml THF, upon which the mixture turned orange and gas evolution was observed. After 18 h stirring at RT the solution was filtered and all volatiles were removed under reduced pressure, resulting in an orange solid (1.53 g, 4.34 mmol, 94 %).

Procedure B (for larger amounts): Benzyl(triphenyl)phosphonium bromide (15.0 g, 34.6 mmol, 1.00 eq.) and NaHMDS (6.35 g, 34.6 mmol, 1.00 eq.) or KHMDS (6.91 g, 34.6 mmol, 1.00 eq) were mixed and cooled to 0 °C. 200 mL THF were added slowly and the reaction mixture turned orange. The reaction was allowed to warm to room temperature overnight and the solution was filtered. The residue was washed with 20 ml THF. Under reduced pressure, the combined THF solutions were evaporated and the remaining orange solid dried at 60 °C *in vacuo* (11.7 g, 33.2 mmol, 96 %).

^{1}H NMR (400.3 MHz, C_6D_6) δ = 7.72-7.63 (m, 6 H, $CH_{PPh_3,ortho}$), 7.14-6.99 (m, 7 H, $CH_{PPh_3,para}$+$CH_{CPh,ortho+meta}$), 6.99-6.91 (m, 6 H, $CH_{PPh_3,meta}$), 6.74-6.63 (m, 1 H, $CH_{CPh,para}$), 2.86 (d, $^2J_{PH}$ = 18.9 Hz, 1 H, PCH_2Ph). **^{1}H NMR** (400.3 MHz, THF-d_8) δ = 7.75-7.66 (m, 6 H, $CH_{PPh_3,ortho}$), 7.56-7.48 (m, 3 H, $CH_{PPh_3,para}$), 7.48-7.40 (m, 6 H, $CH_{PPh_3,meta}$), 6.74-6.58 (m, 2 H, $CH_{CPh,meta}$), 6.59-6.45 (m, 2 H, $CH_{CPh,ortho}$), 6.31-6.16 (m, 1 H, $CH_{CPh,para}$), 2.44 (d, $^2J_{PH}$ = 18.7 Hz, 1 H, PCH_2Ph). **^{13}C{^{1}H} NMR** (100.7 MHz, C_6D_6) δ = 147.3 (d, $^2J_{PC}$ = 7.7 Hz, $C_{CPh,ipso}$), 133.3 (d, $^2J_{PC}$ = 9.8 Hz, $C_{PPh_3,ortho}$), 131.4 (d, $^4J_{PC}$ = 2.8 Hz, $C_{PPh_3,para}$), 130.5 (d, $^1J_{PC}$ = 86.7 Hz, $C_{PPh_3,ipso}$), 128.9 (d, $^3J_{PC}$ = 11.6 Hz, $C_{PPh_3,meta}$), 128.8 (s, $C_{CPh,meta}$), 121.8 (d, $^3J_{PC}$ = 13.9 Hz, $C_{CPh,ortho}$), 115.72 (s, $C_{CPh,para}$), 28.2 (d, $^1J_{PC}$ = 129.2 Hz, PCH_2Ph). **^{31}P{^{1}H} NMR** (162.1 MHz, C_6D_6) δ = 7.79. **^{31}P{^{1}H} NMR** (162.1 MHz, THF-d_8) δ = 7.59. **mp**: 175-178 °C

The analytical and physical data were identical to those previously reported in the literature.[4]

Preparation of protonated precursor of 1

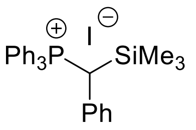

Benzylidene(triphenyl)phosphorane (8.59 g, 24.4 mmol, 1.00 eq.) was suspended in 150 ml toluene at room temperature and iodotrimethylsilane (3.5 ml, 24.7 mmol, 1.01 eq.) was added dropwise. The reaction mixture became slightly warm and white solid precipitated out. The mixture was stirred overnight. The solid was filtered off and washed twice with 75 ml of toluene. Afterwards the colorless solid was dried *in vacuo*(12.7 g, 21.2 mmol, 91 %).

^{1}H NMR (400.3 MHz, CD_2Cl_2) δ = 7.85-7.70 (m, 9 H, $CH_{PPh_3,ortho+para}$), 7.69-7.59 (m, 6 H, $CH_{PPh_3,meta}$), 7.34-7.20 (m, 4 H, $CH_{CPh,ortho+meta}$), 7.20-7.10 (m, 1 H, $CH_{CPh,para}$), 5.19 (d, $^2J_{PH}$ = 20.7 Hz, 1 H, PC*H*Ph), 0.04 (s, 9 H, $Si(CH_3)_3$). **^{1}H NMR** (250.1 MHz, $CDCl_3$) δ = 7.93-7.81 (m, 6 H, $CH_{PPh_3,ortho}$), 7.81-7.72 (m, 3 H, $CH_{PPh_3,para}$), 7.71-7.59 (m, 6 H, $CH_{PPh_3,meta}$), 7.32-7.12 (m, 5 H, CH_{CPh}), 5.71 (d, $^2J_{PH}$ = 20.7 Hz, 1 H, PC*H*Ph), 0.09 (s, 9 H, $Si(CH_3)_3$). **^{13}C{^{1}H} NMR** (100.7 MHz, CD_2Cl_2) δ = 135.4 (d, $^4J_{PC}$ = 3.1 Hz, $C_{PPh_3,para}$), 135.0 (d, $^2J_{PC}$ = 9.8 Hz, $C_{PPh_3,ortho}$), 131.8 (d, $^3J_{PC}$ = 7.36 Hz, $C_{CPh,ortho}$), 130.6 (d, $^3J_{PC}$ = 12.4 Hz, $C_{PPh_3,meta}$), 129.7 (s, $C_{CPh,meta}$), 129.1 (d, $^1J_{PC}$ = 81.6 Hz, $C_{PPh_3,ipso}$), 128.4 (d, $^5J_{PC}$ = 3.0 Hz, $C_{CPh,para}$), 120.5 (d, $^2J_{PC}$ = 85.1 Hz, $C_{CPh,ipso}$), 31.74 (d, $^1J_{PC}$ = 37.7 Hz, PCHSi), 0.50 (d, $^3J_{PC}$ = 2.1 Hz, $Si(CH_3)_3$). **^{31}P{^{1}H} NMR** (162.1 MHz, CD_2Cl_2) δ = 25.1. **^{31}P{^{1}H} NMR** (101.3 MHz, $CDCl_3$) δ = 25.0. **^{29}Si NMR** (79.5 MHz, CD_2Cl_2) δ = 7.36. The ^{29}Si NMR data were obtained from a ^{1}H-^{29}Si correlation spectra. **CHNS** for $C_{28}H_{30}IPSi$: Calcd.: C, 60.7; H, 5.47. measured: C, 60.5; H, 5.16. **mp**: decomp. at 146-147 °C

Preparation of 1

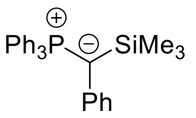

The phosphonium salt precursor to **1** (15.7 g, 28.4 mmol, 1.00 eq.) and NaHMDS (5.21 g, 28.4 mmol, 1.00 eq) were suspended in 200 ml of toluene. After 16 h the red solution was filtered, and the residue was washed twice with 50 ml toluene. The combined toluene solutions were evaporated under reduced pressure. The remaining red oil was dried under reduced pressure in an ultrasonic bath to obtain **1** as an orange solid (11.7 g, 27.5 mmol, 97 %).

^{1}H NMR (400.3 MHz, C_6D_6) δ = 7.76-7.68 (m, 6 H, C$H_{\mathrm{PPh_3,ortho}}$), 7.34-7.28 (m, 2 H, C$H_{\mathrm{CPh,ortho}}$), 7.07-6.95 (m, 11 H, C$H_{\mathrm{PPh_3,para}}$+ C$H_{\mathrm{PPh_3,meta}}$+C$H_{\mathrm{CPh,para\ or\ meta}}$), 6.88-6.81 (m, 1 H, C$H_{\mathrm{CPh,meta\ or\ para}}$), 0.22 (s, 9 H, Si(C$H_3$)$_3$). **^{13}C{^{1}H} NMR** (100.7 MHz, C_6D_6) δ = 147.0 (d, $^2J_{PC}$ = 1.5 Hz, $C_{\mathrm{CPh,ipso}}$), 134.3 (d, $^3J_{PC}$ = 9.1 Hz, $C_{\mathrm{PPh_3,meta}}$), 133.5 (d, $^3J_{PC}$ = 10.6 Hz, $C_{\mathrm{CPh,ortho}}$), 132.5 (d, $^1J_{PC}$ = 83.4 Hz, $C_{\mathrm{PPh_3,ipso}}$), 131.1 (d, ^4J = 2.8 Hz, $C_{\mathrm{PPh_3,para}}$), 128.3 (d, $^2J_{PC}$ = 11.2 Hz, $C_{\mathrm{PPh_3,ortho}}$), 127.7 (d, $^4J_{PC}$ = 0.72 Hz, $C_{\mathrm{CPh,meta}}$) 121.7 (d, $^5J_{PC}$ = 2.3 Hz, $C_{\mathrm{CPh,para}}$), 23.3 (d, $^1J_{PC}$ = 94.9 Hz, PCSi), 3.82 (Si(CH$_3$)$_3$). **^{31}P{^{1}H} NMR** (162.1 MHz, C_6D_6) δ = 13.4. **^{29}Si{^{1}H} NMR** (79.5 MHz, C_6D_6) δ = -7.5. The ^{29}Si NMR data were obtained from a ^{1}H-^{29}Si correlation spectrum. **mp**: 121-122 °C.

The analytical and physical data were identical to those previously reported in the literature.[5]

1.3 Preparation of the chlorophosphines and phosphenium cations

Preparation of the diylidylphosphenium cation 2[BF4]

(adopted from literature[6])

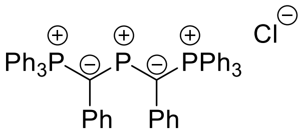

1 (2.1 g, 4.95 mmol, 1.00 eq.) was dissolved in 20 ml toluene and PCl_3 (220 µl, 2.52 mmol, 0.50 eq.) was added dropwise. The reaction was stirred for 3 d at room temperature. The precipitate was isolated by filtration and washed two times with 20 ml toluene and one time with 20 ml pentane. The yellow solid was dried under reduced pressure (1.68 g, 2.19 mmol, 87 %).

^{1}H NMR (400.3 MHz, CD_2Cl_2) δ = 7.67-7.59 (m, 6 H), 7.50-7.42 (m, 12 H), 7.41 – 7.33 (m, 12 H), 6.77-6.69 (m, 2 H), 6.64-6.56 (m, 4 H), 6.49 – 6.43 (m, 4 H). **^{31}P{^{1}H} NMR** (162.1 MHz, CD_2Cl_2) δ = 290.4 (t, J = 165.4 Hz), 24.7 (d, J = 165.4 Hz).

The analytical and physical data were identical to those previously reported in the literature.[6]

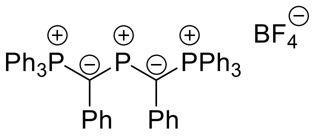

The chloride salt (593 mg, 771 µmol, 1.00 eq.) and $NaBF_4$ (306 mg, 2.79 mmol, 3.60 eq.) were mixed in 30 ml of acetonitrile. The orange suspension was stirred for 18 h at room temperature. The solution was separated from the solid by filtration and evaporated at reduced pressure. The dried filtrate was recrystallized from hot acetonitrile. Acetonitrile built in the crystals was removed by dissolving them in 20 ml DCM and removing of all volatiles under reduced pressure afterwards, yielding in an orange solid (498 mg, 607 µmol, 79 %).

^{1}H NMR (400.3 MHz, CD_2Cl_2) δ = 7.59-7.49 (m, 6 H, $CH_{PPh_3,para}$), 7.42-7.24 (m, 24 H, $CH_{PPh_3,ortho+meta}$), 6.68-6.61 (m, 2 H, $CH_{CPh,para}$), 6.56-6.48 (m, 4 H, $CH_{CPh,ortho\ or\ meta}$), 6.43-6.34 (m, 4 H, $CH_{CPh,ortho\ or\ meta}$). **^{13}C{^{1}H} NMR** (100.7 MHz, CD_2Cl_2) δ = 136.3 (d, $^2J_{PC}$ = 6.4 Hz, $C_{CPh,ipso}$), 135.4-134.6 (m, $C_{PPh_3,ortho}$), 133.8 ($C_{PPh_3,para}$), 132.3 (d, $^3J_{PC}$ = 3.0 Hz, $C_{CPh,ortho}$), 130.0-129.2 (m, $C_{PPh_3,meta}$), 128.4 ($C_{CPh,meta}$), 126.4 ($C_{CPh,para}$), 124.2 (ddd, $^1J_{PC}$ = 90.3, $^3J_{PC}$ = 4.4, $^5J_{PC}$ = 1.5 Hz, $C_{PPh3,ipso}$), 97.7 (ddd, $^1J_{PC}$ = 91.5, $^1J_{PC}$ = 72.0, $^3J_{PC}$ = 20.2 Hz, P*C*P). **^{31}P{^{1}H} NMR** (162.1 MHz, CD_2Cl_2) δ = 290.4 (t, $^2J_{PP}$ = 165.3 Hz, PhC*P*CPh), 24.7 (d, $^2J_{PP}$ = 165.3 Hz, *P*Ph$_3$). **^{11}B NMR** (128.4 MHz, CD_2Cl_2) δ = -1.10. **^{19}F NMR** (235.3 MHz, CD_2Cl_2) δ = -153.5. **CHNS** for $C_{50}H_{40}P_3BF_4$: Calcd.: C, 73.2; H, 4.91. measured: C, 72.9; H, 5.03. **mp**: 274-275 °C.

Preparation of 3a

(similar procedures were already reported in literature[7])

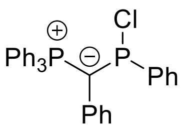

1 (4.00 g, 9.42 mmol, 1.00 eq) was suspended in 100 ml pentane and dichlorophenyl phosphine (1.30 ml, 9.52 mmol, 1.10 eq.) was added dropwise. After stirring for 3 d at room temperature the formed yellow solid was filtered off and washed two times with 20 ml of hexane. The combined organic phases were extracted with 150 ml hot toluene (100 °C). The solvent was evaporated under reduced pressure, thus giving a yellow solid. Layering a saturated DCM solution of this solid with hexane gave the product as yellow crystals (3.97 g, 8.03 mmol, 85 %).

^{1}H NMR (400.3 MHz, CD_2Cl_2) δ = 7.75-7.66 (m, 6 H, $CH_{PPh_3,ortho}$), 7.66-7.58 (m, 5 H, $CH_{PPh_3,para}$+$CH_{PPh,meta}$), 7.56-7.47 (m, 6 H, $CH_{PPh_3,meta}$), 7.23-7.11 (m, 3 H, $CH_{PPh,ortho}$ + $CH_{PPh,para}$), 7.02-6.95 (m, 2 H, $CH_{CPh,ortho}$), 6.88-6.81 (m, 2 H, $CH_{CPh,meta}$), 6.81-6.75 (m, 1 H, $CH_{CPh_3,para}$). **^{13}C{^{1}H} NMR** (100.7 MHz, CD_2Cl_2) δ = 144.6 (dd, $^1J_{PC}$ = 34.0 Hz, $^3J_{PC}$ = 19.1 Hz, $C_{PPh,ipso}$), 140.2 (dd, $^2J_{PC}$ = 8.3 Hz, $^2J_{PC}$ = 3.1 Hz, $C_{CPh,ipso}$), 134.93 (dd, $^2J_{PC}$ = 9.3 Hz, $^4J_{PC}$ = 2.4 Hz, $C_{PPh_3,ortho}$), 132.92 (d, $^4J_{PC}$ = 2.9 Hz, $C_{PPh_3,para}$), 131.8 (d, $^2J_{PC}$ = 20.0 Hz, $C_{PPh,ortho}$), 131.6 (dd, $^3J_{PC}$ = 7.0 Hz, $^3J_{PC}$ = 4.8 Hz, $C_{CPh,ortho}$), 129.3 (d, $^3J_{PC}$ = 12.1 Hz, $C_{PPh_3,meta}$), 128.0 (d, $^4J_{PC}$ = 3.8 Hz, $C_{CPh,meta}$), 127.9 (d, $^3J_{PC}$ = 1.6 Hz, $C_{PPh,meta}$), 127.74 ($C_{PPh,para}$), 127.70 (dd, $^1J_{PC}$ = 88.1 Hz, $^3J_{PC}$ = 8.4 Hz, $C_{PPh_3,ipso}$), 123.6 (d, $^5J_{PC}$ = 1.8 Hz, $C_{CPh,para}$), 51.4 (dd, $^1J_{PC}$ = 103.4 Hz, $^1J_{PC}$ = 58.5 Hz, P*C*P). **^{31}P{^{1}H} NMR** (162.1 MHz, CD_2Cl_2) δ = 131.8 (d, $^2J_{PP}$ = 191.5 Hz, Cl*P*Ph), 24.2 (d, $^2J_{PP}$ = 191.5 Hz, *P*Ph$_3$). **CHNS** for $C_{31}H_{25}P_2Cl$: Calcd.: C, 75.2; H, 5.09. Measured: C, 75.1; H, 5.08. **mp**: 203-204 °C.

Preparation of 3b

The analytical and physical data were identical to those previously reported in the literature.[7]

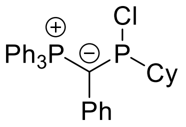

1 (4.01 g, 9.44 mmol, 1.00 eq.) was slurried in 100 ml of pentane and dichlorocyclohexyl phosphine (1.45 ml, 9.40 mmol, 0.99 eq.) was added dropwise. After stirring for 3d at room temperature the precipitated off-white solid was filtered off and washed two times with 20 ml of hexane. The product was extracted with 150 ml hot toluene (100 °C). The solvent was evaporated under reduced pressure, thus giving a colorless solid. Layering of a DCM solution of the solid with hexane gave the product as colorless crystals (3.83 g, 7.65 mmol, 81 %).

^{1}H NMR (400.3 MHz, CD_2Cl_2) δ = 7.64-7.54 (m, 9 H, C$H_{PPh_3,ortho+para}$), 7.50-7.41 (m, 6 H, C$H_{PPh_3,meta}$), 7.26-7.19 (m, 2 H, C$H_{CPh,meta}$), 7.10-7.02 (m, 2 H, C$H_{CPh,ortho}$), 7.02-6.95 (m, 1 H, C$H_{CPh,para}$), 2.23-2.10 (m, 1 H, C$H_{Cy,ipso}$), 2.10-1.86 (m, 2 H, C$H_{Cy,ortho,ax}$), 1.73-1.60 (m, 2 H, C$H_{Cy,meta,ax}$), 1.60-1.48 (m, 1 H, C$H_{Cy,para,ax}$), 1.22-0.95 (m, 5 H, C$H_{Cy,equ}$). **^{13}C{^{1}H} NMR** (101.7 MHz, CD_2Cl_2) δ = 140.7 (dd, $^1J_{PC}$ = 8.4 Hz, $^3J_{PC}$ = 4.2 Hz, $C_{PPh_3,ipso}$), 134.9 (dd, $^2J_{PC}$ = 9.3 Hz, $^4J_{PC}$ = 2.1 Hz, $C_{PPh_3,ortho}$), 132.7 (d, $^4J_{PC}$ = 2.9 Hz, $C_{PPh_3,para}$), 132.3 (dd, $^4J_{PC}$ = 5.8 Hz, $^4J_{PC}$ = 5.1 Hz, $C_{CPh,meta}$), 129.0 (d, $^3J_{PC}$ = 11.9 Hz, $C_{PPh_3,meta}$), 128.4 (dd, $^2J_{PC}$ = 87.7 Hz, $^2J_{PC}$ = 7.3 Hz, $C_{CPh,ipso}$), 128.1 (d, $^3J_{PC}$ = 1.5 Hz, $C_{CPh,orto}$), 124.7 (d, $^5J_{PC}$ = 2.2 Hz, $C_{CPh,para}$), 50.7 (dd, $^1J_{PC}$ = 104.6 Hz, $^1J_{PC}$ = 57.8 Hz, PCP), 42.8 (dd, $^1J_{PC}$ = 26.0 Hz, $^3J_{PC}$ = 13.9 Hz, $C_{Cy,ipso}$), 30.2 (d, ^2J = 18.3 Hz, $C_{Cy,ortho}$), 27.4 (d, $^3J_{PC}$ = 11.1 Hz, $C_{Cy,meta}$), 27.0 (s, C$H_{Cy,para}$). **^{31}P{^{1}H} NMR** (162.1 MHz, CD_2Cl_2) δ = 160.3 (d, $^2J_{PP}$ = 166.1 Hz, Cl*P*Cy), 23.5 (d, $^2J_{PP}$ = 166.1 Hz, *P*Ph_3). **CHNS** for $C_{31}H_{31}P_2Cl$: Calcd.: C, 74.3; H, 6.24. measured: C, 74.2; H, 6.08. **mp**: 184-185 °C.

Preparation of 4a

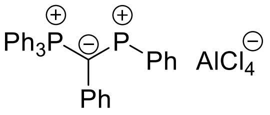

3a (200mg, 404 µmol, 1.00 eq) and $AlCl_3$ (60 mg, 450 µmol, 1.10 eq.) were mixed and cooled to -78 °C. Cold (-78 °C) DCM (4 ml) was added quickly. The yellow solution turned colorless. After 3 h at low temperatures, unreacted $AlCl_3$ was removed by filtration. It is important to keep the solution at -78 °C. The solvent was removed under reduced pressure at -78 °C. The solid was warmed up to room temperature and dried under reduced pressure giving the product as bronze-colored solid, which was stored at -30 °C (182 mg, 290 mmol, 72 %).

^{1}H NMR (400.3 MHz, CD_2Cl_2) δ = 7.93-7.85 (m, 3 H, C$H_{PPh_3,para}$), 7.74-7.65 (m, 6 H, C$H_{PPh_3,meta}$), 7.62-7.52 (m, 6 H, C$H_{PPh_3,ortho}$), 7.45-7.38 (m, 1 H, C$H_{CPh,para}$), 7.38-7.32 (m, 1 H, C$H_{PPh,para}$), 7.30-7.19 (m, 6 H, C$H_{CPh,ortho+meta}$+C$H_{PPh,ortho}$), 6.79-6.71 (m, 2 H, C$H_{PPh,meta}$). **^{13}C{^{1}H} NMR** (100.7 MHz, CD_2Cl_2) δ = 165.1 (dd, $^1J_{PC}$ = 66.8, $^1J_{PC}$ = 62.9 Hz, P*C*P), 139.2 (dd, $^1J_{PC}$ = 53.2, $^3J_{PC}$ = 20.7 Hz, $C_{PPh,ipso}$), 136.1 (d, $^4J_{PC}$ = 3.0 Hz, $C_{PPh_3,para}$), 135.5 (dd, *J* = 9.7, *J* = 2.1 Hz, $C_{PPh_3,ortho}$), 133.7 (s, $C_{CPh,para}$), 133.5 (s, $C_{CPh,meta}$), 131.1 (dd, $^2J_{PC}$ = 14.9, $^2J_{PC}$ = 12.7 Hz, $C_{CPh,ipso}$), 130.8 (d, $^3J_{PC}$ = 12.8 Hz, $C_{PPh_3,meta}$), 130.3 (d, $^4J_{PC}$ = 3.0 Hz, $C_{PPh,para}$), 130.2 (d, *J* = 2.3 Hz, $C_{PPh,meta}$), 130.0 (dd, $^3J_{PC}$ = 6.0 Hz, $^3J_{PC}$ = 6.0 Hz, $C_{CPh,ortho}$), 129.3 (d, $^2J_{PC}$ = 7.3 Hz, $C_{PPh,ortho}$), 118.2 (dd, $^1J_{PC}$ = 88.7, $^3J_{PC}$ = 5.0 Hz, $C_{PPh_3,ipso}$). **^{31}P{^{1}H} NMR** (162.1 MHz, CD_2Cl_2) δ = 350.9 (d, $^2J_{PP}$ = 123.7 Hz, *P*Ph), 25.7 (d, $^2J_{PP}$ = 123.7 Hz, *P*Ph_3). **^{27}Al NMR** (104.3 MHz, CD_2Cl_2) δ = 104.0. **CHNS** for $C_{31}H_{25}P_2AlCl_4$: Calcd.: C, 59.3; H, 4.01. measured: C, 59.3; H, 4.20. **mp**: slow decomp at 25 °C.

Preparation of 4b

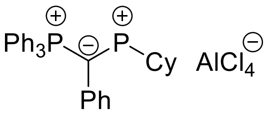

3b (201mg, 402 µmol, 1.00 eq.) and $AlCl_3$ (63.8 mg, 479 µmol, 1.20 eq.) were mixed and cooled to -78 °C. Cold (-78 °C) DCM (4 ml) was added quickly. The solution remained yellow. After 3 h the unreacted $AlCl_3$ was removed by filtration. It is important to keep the solution at -78 °C. The solvent was removed under reduced pressure at -78 °C. The solid was warmed up to room temperature and dried under reduced pressure giving the product as off-white solid, which was stored at -30 °C (185 mg, 292 mmol, 73 %).

^{1}H NMR (400.3 MHz, CD_2Cl_2) δ = 7.92-7.80 (m, 3 H, C$H_{PPh_3,para}$), 7.70-7.61 (m, 6 H, C$H_{PPh_3,meta}$), 7.50-7.37 (m, 7 H, C$H_{PPh_3,ortho}$+C$H_{CPh,para}$), 7.37-7.29 (m, 2 H, C$H_{CPh,meta}$), 6.78-6.70 (m, 2 H, C$H_{CPh,ortho}$), 2.47-2.31 (m, 1 H, C$H_{Cy,ipso,ax}$), 1.78-1.65 (m, 2 H, C$H_{Cy,ortho,ax}$), 1.65-1.45 (m, 5 H, C$H_{Cy,equ}$), 1.32-1.15 (m, 1 H, C$H_{Cy,para,ax}$), 1.15-1.00 (m, 2 H, C$H_{Cy,meta,ax}$). **^{13}C{^{1}H} NMR** (100.7 MHz, CD_2Cl_2) δ = 169.0 (dd, $^{1}J_{PC}$ = 69.6 Hz, $^{1}J_{PC}$ = 62.7 Hz, P*C*P), 136.0 (d, $^{4}J_{PC}$ = 3.1 Hz, $C_{PPh_3,para}$), 135.4 (dd, $^{2}J_{PC}$ = 9.9 Hz, $^{4}J_{PC}$ = 1.7 Hz, $C_{PPh_3,ortho}$), 135,3 (dd, $^{2}J_{PC}$ = 13.7 Hz, $^{2}J_{PC}$ = 3.2 Hz, $C_{CPh,ipso}$) 130.7 (d, $^{3}J_{PC}$ = 12.7 Hz, $C_{PPh_3,meta}$), 130.1 (d, $^{5}J_{PC}$ = 2.9 Hz, $C_{CPh,para}$), 130.0 (d, $^{4}J_{PC}$ = 2.2 Hz, $C_{CPh,para}$), 129.2 (dd, $^{3}J_{PC}$ = 6.7 Hz, $^{3}J_{PC}$ = 5.1 Hz, $C_{CPh,ortho}$), 118.0 (dd, $^{1}J_{PC}$ = 88.7 Hz, $^{3}J_{PC}$ = 4.3 Hz, $C_{PPh_3,ipso}$), 44.9 (dd, $^{1}J_{PC}$ = 43.0 Hz, $^{3}J_{PC}$ = 12.8 Hz, $C_{Cy,ipso}$), 29.7 (d, $^{2}J_{PC}$ = 12.8 Hz, $C_{Cy,ortho}$), 26.2 (d, $^{3}J_{PC}$ = 9.3 Hz, $C_{Cy,meta}$), 25.7 (s, $C_{Cy,para}$). **^{31}P{^{1}H} NMR** (162.1 MHz, CD_2Cl_2) δ = 403.1 (d, $^{2}J_{PP}$ = 100.4 Hz, *P*Cy), 24.8 (d, $^{2}J_{PP}$ = 100.4 Hz, *P*Ph$_3$). **^{27}Al NMR** (104.3 MHz, CD_2Cl_2) δ = 103.8. **CHNS** for $C_{31}H_{31}P_2AlCl_4$: Calcd.: C, 58.7; H, 4.93. measured: C, 58.5; H, 5.07. **mp**: slow decomp at 25 °C.

1.4 Preparation of metal complexes

[Y$_2$PAuCl]AuCl$_2$ (5a)

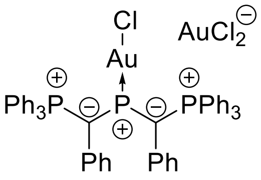

2[Cl] (100 mg, 130 µmol, 1.00 eq.) and (tht)AuCl (87.5 mg, 273 µmol, 2.10 eq.) were stirred in 20 ml toluene for 18 h. The solid was filtered off and washed twice with 15 ml toluene and then dissolved in 5 ml DCM. Insoluble impurities were filtered off. Slow vapor diffusion of pentane into the DCM solution yielded the title compound as yellow crystals (134 mg, 109 µmol, 84 %).

^{1}H NMR (400.3 MHz, CD_2Cl_2) δ = 7.72-7.66 (m, 6 H, C$H_{PPh_3,para}$), 7.58-7.49 (m, 24 H, C$H_{PPh_3,ortho+meta}$), 6.77-6.69 (m, 2 H, C$H_{CPh,para}$), 6.64-6.52 (m, 8 H, C$H_{CPh,ortho+meta}$). **^{13}C{^{1}H} NMR** (100.6 MHz, CD_2Cl_2) δ = 135.4-134.9 (m, $C_{PPh_3,ortho}$), 134.6 (s, $C_{PPh_3,para}$), 134.4-133.9 (m, $C_{CPh,meta}$), 133.0 (d, $^{2}J_{PC}$ = 7.3 Hz, $C_{CPh,ipso}$), 130.3-129.8 (m, $C_{PPh_3,meta}$), 128.8 (s, $C_{CPh,ortho}$), 127.9 (s, $C_{CPh,para}$), 123.3-121.6 (m, $C_{PPh_3,ipso}$), 86.0 (ddd, $^{1}J_{PC}$ = 93.4 Hz, $^{1}J_{PC}$ = 50.3 Hz, $^{3}J_{PC}$ = 12.3 Hz, P*C*P). **^{31}P{^{1}H} NMR** (162.1 MHz, CD_2Cl_2) δ = 228.5 (t, $^{2}J_{PP}$ = 99.5 Hz, PhC*P*CPh), 26.0 (d, $^{2}J_{PP}$ = 99.5 Hz, *P*Ph$_3$). **CHNS** for $C_{50}H_{40}Au_2Cl_3P_3$: Calcd.: C, 48.66; H, 3.27. measured: C, 48.27; H, 3.22.

[Y$_2$PAuCl]BF$_4$ (5b)

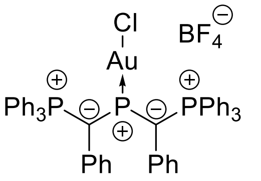

2[BF$_4$] (100 mg, 122 µmol, 1.00 eq.) and (tht)AuCl (43.0 mg, 134 µmol, 1.10 eq.) were stirred in 10 ml toluene for 18 h. The solid was filtered off and washed two times with 10 ml toluene each and then dissolved in 5 ml DCM. Insoluble impurities were filtered off. Slow vapor diffusion of pentane into the DCM solution yielded the title compound as orange crystals (104 mg, 99 µmol, 81 %).

^{1}H NMR (400.3 MHz, CD_2Cl_2) δ = 7.72-7.64 (m, 6 H, $CH_{PPh_3,para}$), 7.59-7.39 (m, 24 H, $CH_{PPh_3,ortho+meta}$), 6.76-6.68 (m, 2 H, $CH_{CPh,para}$), 6.62-6.54 (m, 4 H, $CH_{CPh,ortho}$), 6.50-6.44 (m, 4 H, $CH_{CPh,meta}$). **^{13}C{^{1}H} NMR** (100.7 MHz, CD_2Cl_2) δ = 135.3-134.9 (m, $C_{PPh_3,ortho}$), 134.7 (s, $C_{PPh_3,para}$), 134.4-133.5 (m, $C_{CPh,meta}$), 133.1 (d, $^{2}J_{PC}$ = 6.8 Hz, $C_{CPh,ipso}$), 130.4-129.7 (m, $C_{PPh_3,meta}$), 128.9 (s, $C_{CPh,ortho}$), 128.0 (s, $C_{CPh,para}$), 122.5 (m, $C_{PPh_3,ipso}$), 86.1 (ddd, $^{1}J_{PC}$ = 93.6 Hz, $^{1}J_{PC}$ = 50.4 Hz, $^{3}J_{PC}$ = 12.7 Hz, P*C*P). **^{31}P{^{1}H} NMR** (162.1 MHz, CD_2Cl_2) δ = 229.2 (t, $^{2}J_{PP}$ = 98.9 Hz, PhC*P*CPh), 26.0 (d, $^{2}J_{PP}$ = 98.9 Hz, *P*Ph$_3$). **^{19}F NMR** (75.4 MHz, CD_2Cl_2) δ = -150.7. **^{11}B NMR** (128.4 MHz, CD_2Cl_2) δ = -0.97. **CHNS** for $C_{50}H_{40}AuBClF_4P_3$: Calcd.: C, 57.03; H 3.83, measured: C, 56.58; H, 4.19.

YPPhClAuCl (7a)

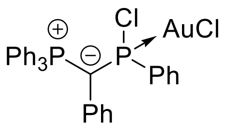

3a (30.3 mg, 61.2 µmol, 1.00 eq.) and (tht)AuCl (19.6 mg, 61.2 µmol, 1.00 eq.) were mixed and 1 ml DCM was added. The solution was stirred at room temperature overnight. Insoluble impurities were filtered off. Slow vapor diffusion of pentane into the filtrate yielded the title compound as a colorless solid (20.5 mg, 28.2 µmol, 46 %).

^{1}H NMR* (400.3 MHz, CD_2Cl_2) δ = 7.70-7.55 (m, 11 H, $CH_{PPh_3,ortho+para}$+ $CH_{PPh,ortho}$), 7.55-7.46 (m, 6 H, $CH_{PPh_3,meta}$), 7.34-7.28 (m, 1 H, $CH_{CPh,para}$), 7.28-7.20 (m, 2 H, $CH_{PPh,meta}$), 7.03-6.95 (m, 1 H, $CH_{PPh,para}$), 6.95-6.87 (m, 2 H, $CH_{CPh,meta}$), 6.86-6.77 (m, 2 H, $CH_{CPh,ortho}$), 2.90-2.72 (m, 4 H, THT), 1.98-1.86 (m, 4 H, THT). **^{13}C NMR*** (100.7 MHz, CD_2Cl_2) δ = 138.2 (dd, $^{2}J_{PC}$ = 80.3 Hz, $^{2}J_{PC}$ = 7.3 Hz, $C_{CPh,ipso}$), 136.4 (s, $C_{CPh,ortho}$), 136.0 (d, $^{1}J_{PC}$ = 68.0 Hz, $C_{PPh,ipso}$), 134.8 (d, $^{2}J_{PC}$ = 9.5 Hz, $C_{PPh_3,ortho}$), 133.5 (d, 4J_{PC} = 2.9 Hz, $C_{PPh_3,para}$), 132.1 (d, $^{2}J_{PC}$ = 16.3 Hz, $C_{PPh,ortho}$), 131.0 (s, $C_{CPh,para}$), 129.5 (d, $^{3}J_{PC}$ = 12.4 Hz, $C_{PPh_3,meta}$), 128.40 (d, $^{3}J_{PC}$ = 13.4 Hz, $C_{PPh,meta}$), 128.38 (d, $^{4}J_{PC}$ = 2.3 Hz, $C_{CPh,meta}$), 127.0 (s, $C_{PPh,para}$), 126.4 (dd, $^{1}J_{PC}$ = 91.0, $^{3}J_{PC}$ = 3.6 Hz, $C_{PPh_3,ipso}$), 44.87 (dd, $^{1}J_{PC}$ = 112.4 Hz, $^{1}J_{PC}$ = 67.8 Hz, P*C*P), 32.19 (s, THT), 31.63 (s, THT). **^{31}P NMR** (162.1 MHz, CD_2Cl_2) δ = 83.5 (d, $^{2}J_{PP}$ = 98.5 Hz, Cl*P*Ph), 25.0 (d, $^{2}J_{PP}$ = 98.5 Hz, *P*Ph$_3$).

* contains THT for stabilization.

CHNS for $C_{31}H_{25}AuCl_2P_2$.: C, 51.19; H, 3.83, measured: C, 51.75; H, 3.49.

YPCyClAuCl (7b)

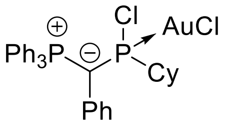

3b (30.0 mg, 59.9 µmol, 1.00 eq.) and (tht)AuCl (19.2 mg, 59.9 µmol, 1.00 eq.) were mixed and 1 ml DCM was added. The reaction mixture was stirred at room temperature overnight and insoluble impurities were filtered off. Slow vapor diffusion of pentane into the filtrate yielded the title compound as a colorless solid (18.9 mg, 25.8 µmol, 43 %).

^{1}H NMR* (400.3 MHz, CD_2Cl_2) δ = 7.68-7.56 (m, 9 H, $CH_{PPh_3,ortho+para}$), 7.53-7.44 (m, 6 H, $CH_{PPh_3,meta}$), 7.20-7.12 (m, 2 H, $CH_{CPh,ortho}$), 7.12-7.03 (m, 3 H, $CH_{CPh,meta+para}$), 2.31-2.20 (m,

1 H, C$H_{Cy,ipso}$), 2.20-2.10 (m, 1 H, C$H_{Cy,ortho,axial}$), 2.06-1.88 (m, 1 H, C$H_{Cy,ortho,axial}$), 1.86-1.72 (m, 2 H, C$H_{Cy,meta,axial}$), 1.65-1.53 (m, 1 H, C$H_{Cy,para,axial}$), 1.53-1.35 (m, 2 H, C$H_{Cy,ortho,äquatorial}$), 1.25-1.10 (m, 2 H, C$H_{Cy,meta,äquatorial}$), 1.10-0.94 (m, 1 H, C$H_{Cy,para,äquatorial}$). **^{13}C{^{1}H} NMR*** (100.7 MHz, CD_2Cl_2) δ = 136.9 (dd, $^2J_{PP}$ = 5.5 Hz, $^2J_{PC}$ = 5.5 Hz, $C_{CPh,ipso}$), 135.0 (dd, $^3J_{PC}$ = 8.4 Hz, $^3J_{PP}$ = 4.8 Hz, $C_{CPh,ortho}$), 134.9 (d, $^2J_{PC}$ = 9.3 Hz, $C_{PPh_3,ortho}$), 133.3 (d, $^4J_{PC}$ = 2.9 Hz, $C_{PPh_3,para}$), 129.4 (d, $^3J_{PC}$ = 12.2 Hz, $C_{PPh_3,meta}$), 128.6 (d, $^4J_{PC}$ = 2.2 Hz, $C_{CPh,meta}$), 127.0 (s, $C_{CPh,para}$), 126.7 (dd, $^1J_{PC}$ = 90.8 Hz, $^3J_{PC}$ = 4.6 Hz, $C_{PPh_3,para}$), 45.2 (dd, $^1J_{PC}$ = 48.3 Hz, $^3J_{PC}$ = 7.4 Hz, $C_{Cy,ipso}$), 42.0 (dd, $^1J_{PC}$ = 111.9 Hz, $^1J_{PC}$ = 55.1 Hz, P*C*P), 30.6 (d, $^2J_{PC}$ = 4.7 Hz, $C_{Cy,ortho}$), 30.1 (d, $^2J_{PC}$ = 3.4 Hz, $C_{Cy,ortho}$), 26.9 (d, $^3J_{PC}$ = 17.0 Hz, $C_{Cy,meta}$), 26.8 (d, $^3J_{PC}$ = 17.9 Hz, $C_{Cy,meta}$), 26.2 (d, $^4J_{PC}$ = 2.0 Hz, $C_{Cy,para}$). **^{31}P{^{1}H} NMR** (162.1 MHz, CD_2Cl_2) δ = 107.5 (d, $^2J_{PP}$ = 77.0 Hz, Cl*P*Cy), 24.5 (d, $^2J_{PP}$ = 77.0 Hz, *P*Ph_3).

* contains THT for stabilization.

CHNS for $C_{31}H_{31}AuCl_2P_2$.: C, 50.77; H, 4.26, measured: C, 50.27; H, 4.27.

[YPPhClAu(tht)]($AlCl_4$) (6a)

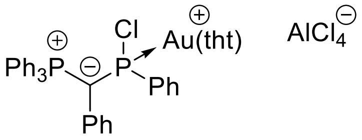

Procedure A: **3a** (30.0 mg, 60.6 µmol, 1.00 eq.) and $AlCl_3$ (8.1 mg, 60.6 µmol, 1.00 eq.) were mixed and 5 ml DCM was added. The reaction was stirred for 10 minutes and (tht)AuCl (19.4 mg, 60.5 µmol, 0.99 eq.) was added. The reaction was stirred for 5 minutes and insoluble impurities were filtered off. Slow vapor diffusion of pentane gave the title compound as a colorless solid (35.2 mg, 37.1 µmol, 61 %).

Procedure B: *In situ* generated **4a** from **3a** (30 mg, 60.6 µmol, 1.00 eq.) and (tht)AuCl (19.4 mg, 60.6 µmol, 1.00 eq.) in 1 ml DCM was mixed with $(Et_2O)AlCl_3$ (12.6 mg, 60.7 µmol, 1.00 eq.). The reaction was stirred for 20 minutes, and insoluble impurities were filtered off. Slow vapor diffusion of pentane into the filtrate gave the title compound as colorless crystals (24.7 mg, 35.7 µmol, 59 %).

^{1}H NMR (400.3 MHz, CD_2Cl_2) δ = 7.75-7.67 (m, 3 H, C$H_{PPh_3,para}$), 7.66-7.53 (m, 12 H, C$H_{PPh_3,ortho+meta}$), 7.52-7.40 (m, 1 H, C$H_{PPh,para}$), 7.40-7.27 (m, 4 H, C$H_{PPh,ortho+meta}$), 7.05-6.98 (m, 1 H, C$H_{CPh,para}$), 6.97-6.90 (m, 2 H, C$H_{CPh,meta}$), 6.85-6.78 (m, 2 H, C$H_{CPh,ortho}$), 3.28-3.11 (m, 4 H, S(CH_2CH_2)$_2$), 2.18-1.98 (m, 4 H, S(CH_2CH_2)$_2$). **^{13}C{^{1}H} NMR** (100.7 MHz, CD_2Cl_2) δ = 136.2 (dd, $^3J_{PC}$ = 8.1 Hz, $^3J_{PC}$ = 4.1 Hz, $C_{CPh,ortho}$), 135.0 (dd, $^1J_{PC}$ = 88.7 Hz, $^3J_{PC}$ = 9.5 Hz, $C_{PPh,ipso}$), 134.8 (dd, $^2J_{PC}$ = 9.6 Hz, $^4J_{PC}$ = 1.1 Hz, $C_{PPh_3,ortho}$), 134.1 (d, $^4J_{PC}$ = 2.9 Hz, $C_{PPh_3,para}$), 132.0 (d, $^3J_{PC}$ = 15.9 Hz, $C_{PPh,meta}$), 131.8 (d, $^4J_{PC}$ = 3.0 Hz, $C_{PPh,para}$), 129.9 (d, $^3J_{PC}$ = 12.3 Hz, $C_{PPh_3,meta}$), 129.5 (dd, $^2J_{PC}$ = 12.2 Hz, $^2J_{PC}$ = 6.6 Hz, $C_{CPh,ipso}$), 129.0 (d, $^2J_{PC}$ = 14.2 Hz, $C_{PPh,ortho}$), 128.7 (dd, $^4J_{PC}$ = 1.8 Hz, $^4J_{PC}$ = 1.8 Hz, $C_{CPh,meta}$), 127.5 (d, $^5J_{PC}$ = 2.7 Hz, $C_{CPh,para}$), 125.7 (dd, $^1J_{PC}$ = 90.9 Hz, $^3J_{PC}$ = 4.7 Hz, $C_{PPh_3,ipso}$), 44.8 (dd, $^1J_{PC}$ = 111.5 Hz, $^1J_{PC}$ = 62.8 Hz, P*C*P), 40.0 (s, S(*C*H_2CH$_2$)$_2$), 31.4 (s, S(CH$_2$*C*H$_2$)$_2$). **^{31}P{^{1}H} NMR** (162.1 MHz, CD_2Cl_2) δ = 83.6 (d, $^2J_{PP}$ = 92.6 Hz, Cl*P*Ph), 25.1 (d, $^2J_{PP}$ = 92.6 Hz, *P*Ph_3). **^{27}Al NMR** (104.3 MHz, CD_2Cl_2) δ = 103.8.

[YPCyClAu(tht)](AlCl$_4$) (6b)

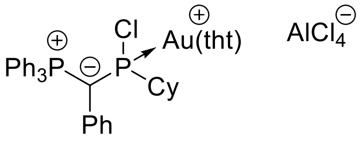

Procedure A: **3a** (30.0 mg, 59.9 µmol, 1.00 eq.) and $AlCl_3$ (8.0 mg, 60.0 µmol, 1.01 eq.) were dissolved in 1 ml DCM. The reaction was stirred for 10 minutes and (tht)AuCl (19.4 mg, 60.5 µmol, 1.05 eq.) was added. The reaction was stirred for 20 minutes and insoluble impurities were filtered off. Slow vapor diffusion of pentane into the filtrate gave the title compound as colorless crystals (37.4 mg, 39.2 µmol, 65 %).

Procedure B: *In situ* generated **4b** from **3b** (30 mg, 59.9 µmol, 1.00 eq.) and (tht)AuCl (19.2 mg, 59.9 µmol, 1.00 eq) in 1 ml DCM was mixed with (Et_2O)$AlCl_3$ (12.4 mg, 59.8 µmol, 0.99 eq.). The reaction was stirred for 20 minutes and insoluble impurities were filtered off. Slow vapor diffusion of pentane into the filtrate gave the title compound as colorless crystals (26,3 mg, 37.7 µmol, 63 %).

^{1}H NMR* (400.3 MHz, CD_2Cl_2) δ = 7.74-7.66 (m, 3 H, $CH_{PPh_3,para}$), 7.64-7.52 (m, 12 H, $CH_{PPh_3,ortho+meta}$), 7.18-7.07 (m, 5 H, CH_{CPh}), 3.11-3.01 (m, 4 H, S(CH_2CH_2)$_2$), 2.37-2.25 (m, 1 H, $CH_{Cy,ipso}$), 2.25-2.08 (m, 2 H, $CH_{Cy,ortho,ax}$), 2.08-1.97 (m, 4 H, S(CH_2CH_2)$_2$), 1.93-1.75 (m, 2 H, $CH_{Cy,meta,ax}$), 1.64 (dd, *J* = 9.2, 4.5 Hz, 1 H, $CH_{Cy,para,ax}$), 1.43-0.99 (m, 5 H, $CH_{Cy,equ}$). **^{13}C{^{1}H} NMR*** (100.6 MHz, CD_2Cl_2) δ = 135.5 (dd, $^2J_{PC}$ = 5.3 Hz, $^2J_{PC}$ = 5.3 Hz, $C_{CPh,ipso}$), 134.9 (dd, $^3J_{PC}$ = 8.7 Hz, $^3J_{PC}$ = 4.4 Hz, $C_{CPh,ortho}$), 134.8 (d, $^2J_{PC}$ = 9.6 Hz, $C_{PPh_3,ortho}$), 134.0 (d, $^4J_{PC}$ = 2.9 Hz, $C_{PPh_3,para}$), 129.8 (d, $^3J_{PC}$ = 12.2 Hz, $C_{PPh_3,meta}$), 129.0 (d, $^4J_{PC}$ = 2.3 Hz, $C_{CPh,meta}$), 127.6 (d, $^5J_{PC}$ = 2.2 Hz, $C_{CPh,para}$), 125.9 (dd, $^1J_{PC}$ = 90.6 Hz, $^3J_{PC}$ = 4.6 Hz, $C_{PPh_3,ipso}$), 45.2 (dd, $^1J_{PC}$ = 47.0 Hz, $^3J_{PC}$ = 7.1 Hz, $C_{Cy,ipso}$), 41.8 (dd, $^1J_{PC}$ = 110.9 Hz, $^1J_{PC}$ = 54.9 Hz, P*C*P), 39.4 (d, 3J_{PC} = 1.8 Hz, S(CH_2CH_2)$_2$), 31.5 (s, S(CH_2CH_2)$_2$), 31.1 (d, $^2J_{PC}$ = 3.9 Hz, $C_{Cy,ortho}$), 30.7 (d, $^2J_{PC}$= 2.5 Hz, $C_{Cy,ortho}$), 26.9 (d, $^3J_{PC}$ = 17.1 Hz, $C_{Cy,meta}$), 26.8 (d, $^3J_{PC}$ = 17.8 Hz, $C_{Cy,meta}$), 26.2 (d, $^4J_{PC}$ = 2.1 Hz, $C_{Cy,para}$). **^{31}P{^{1}H} NMR** (162.1 MHz, CD_2Cl_2) δ = 106.6 (d, $^2J_{PP}$ = 73.2 Hz, Cl*P*Cy), 24.8 (d, $^2J_{PP}$ = 73.2 Hz, *P*Ph$_3$). **^{27}Al NMR** (104.3 MHz, CD_2Cl_2) δ = 103.8.

* contains THT for stabilization.

[YPCyRhCl(cod)]AlCl$_4$

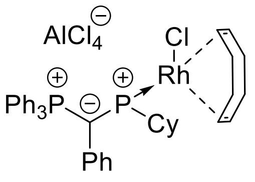

3b (30.0 mg, 59.9 µmol, 1.00 eq.) and $AlCl_3$ (8.0 mg, 60.0 µmol, 1.01 eq.) were mixed in a J-Young NMR tube and 0.5 ml CD_2Cl_2 were added. [((cod)RhCl)$_2$] (14.8 mg, 30.0 µmol, 0.50 eq.) was added and the solution turned dark red. Without any workup, the mixture was investigated via ^{31}P{^{1}H} NMR spectroscopy. Single crystals for XRD could be obtained by slow vapor diffusion of pentane into the reaction mixture.

^{31}P{^{1}H} NMR (162.1 MHz, CD_2Cl_2) δ = 371.5 (br dd, $^1J_{PRh}$ = 163.5 Hz, $^2J_{PP}$ = 52.5 Hz, *P*Cy), 24.3 (d, $^2J_{PP}$ = 52.5 Hz, Ph$_3$*P*)

[YPPhClPdCl(cod)]AlCl$_4$

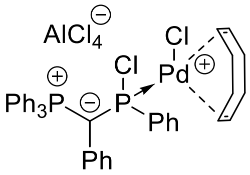

3a (30.0 mg, 60.6 µmol, 1.00 eq.) and $AlCl_3$ (8.1 mg, 60.6 µmol, 1.00 eq.) were mixed in a J-Young NMR tube and 0.5 ml CD_2Cl_2 were added. (cod)$PdCl_2$ (17.3 mg, 60.6 µmol) was added and the solution turned dark red. Without any workup, the mixture was investigated *via* ^{31}P{^{1}H} NMR spectroscopy.

^{31}P{^{1}H} NMR (162.1 MHz, CD_2Cl_2) δ = 78.4 (d, $^{2}J_{PP}$ = 9.2 Hz, *P*Ph), 17.4 (d, $^{2}J_{PP}$ = 9.2 Hz, *P*Ph$_3$).

2 NMR-Spectra

2.1 NMR spectra of the phosphenium cation precursors

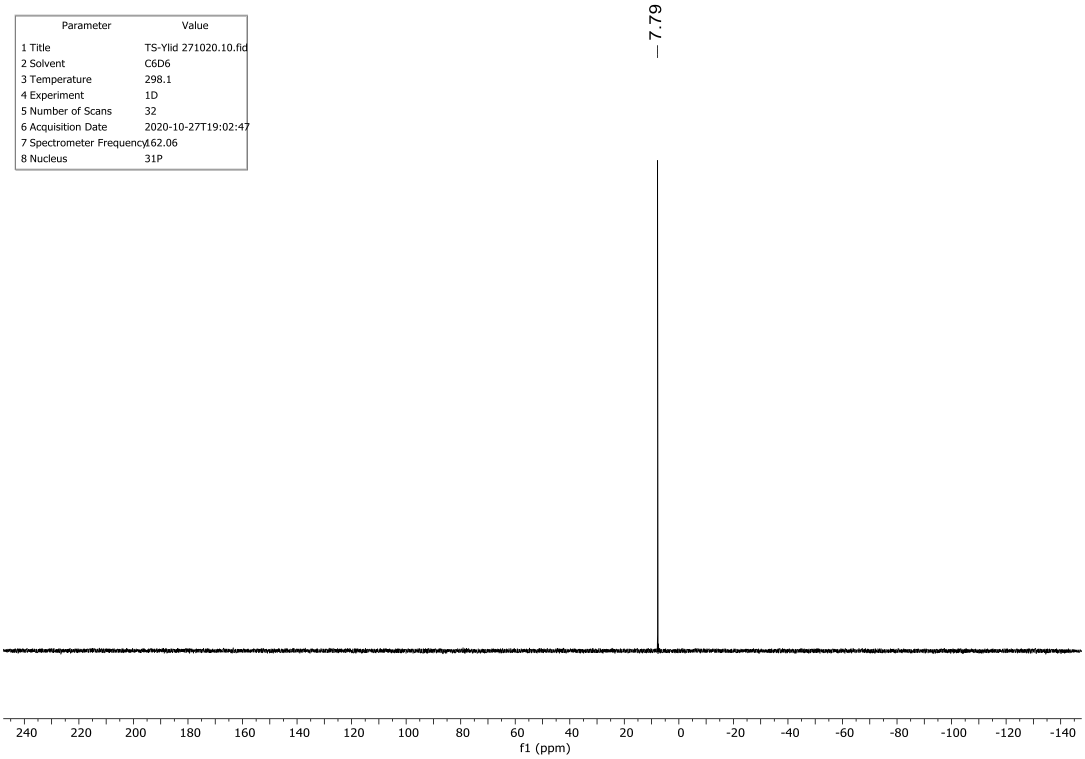

Figure S1: $^{31}P\{^{1}H\}$ NMR spectrum of benzylidene(triphenyl)phosphorane.

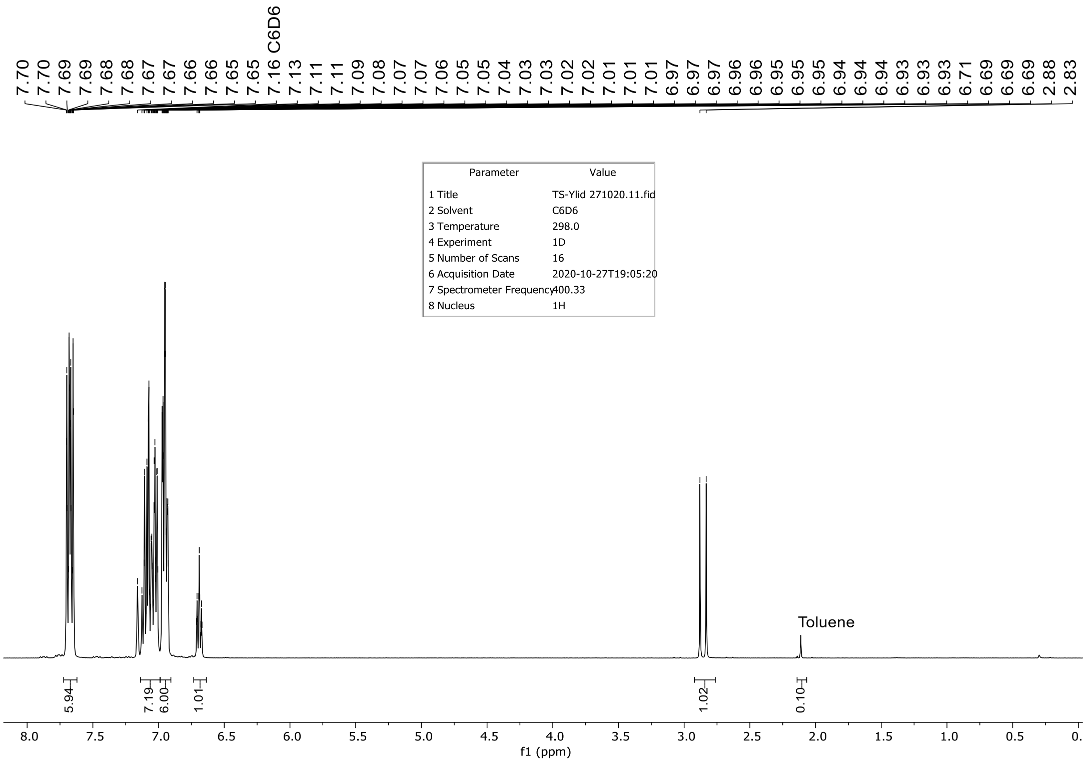

Figure S2: ^{1}H NMR spectrum of benzylidene(triphenyl)phosphorane.

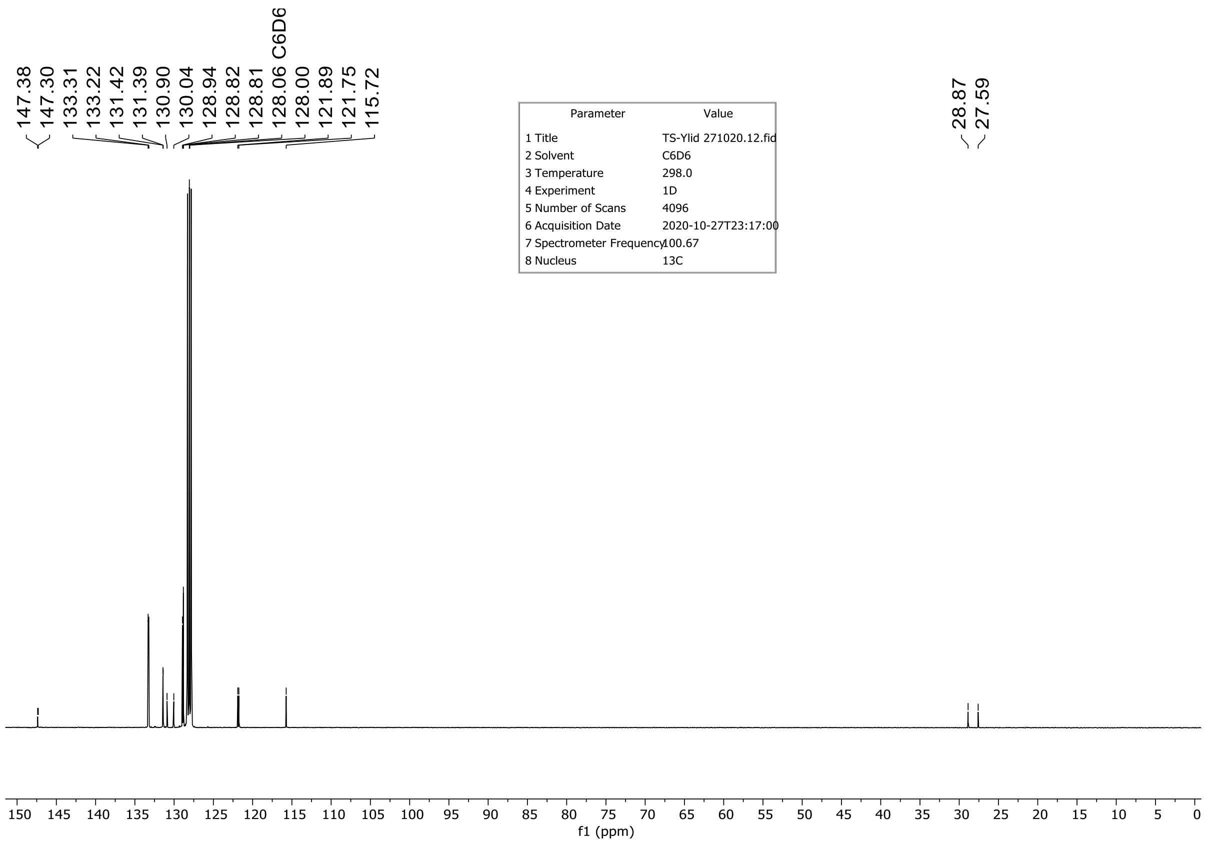

Figure S3: $^{13}C\{^{1}H\}$ NMR spectrum of benzylidene(triphenyl)phosphorane.

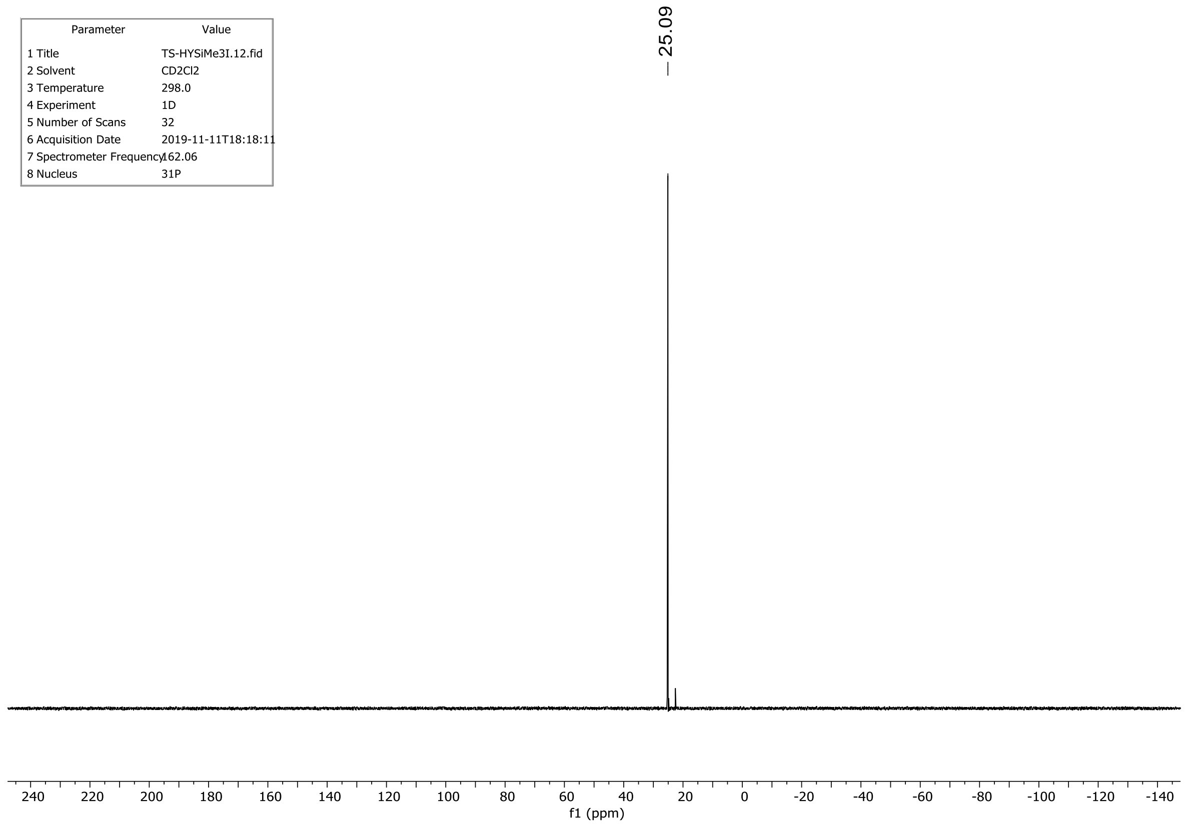

Figure S4: $^{31}P\{^{1}H\}$ NMR spectrum of the phosphonium salt precursor of **1**.

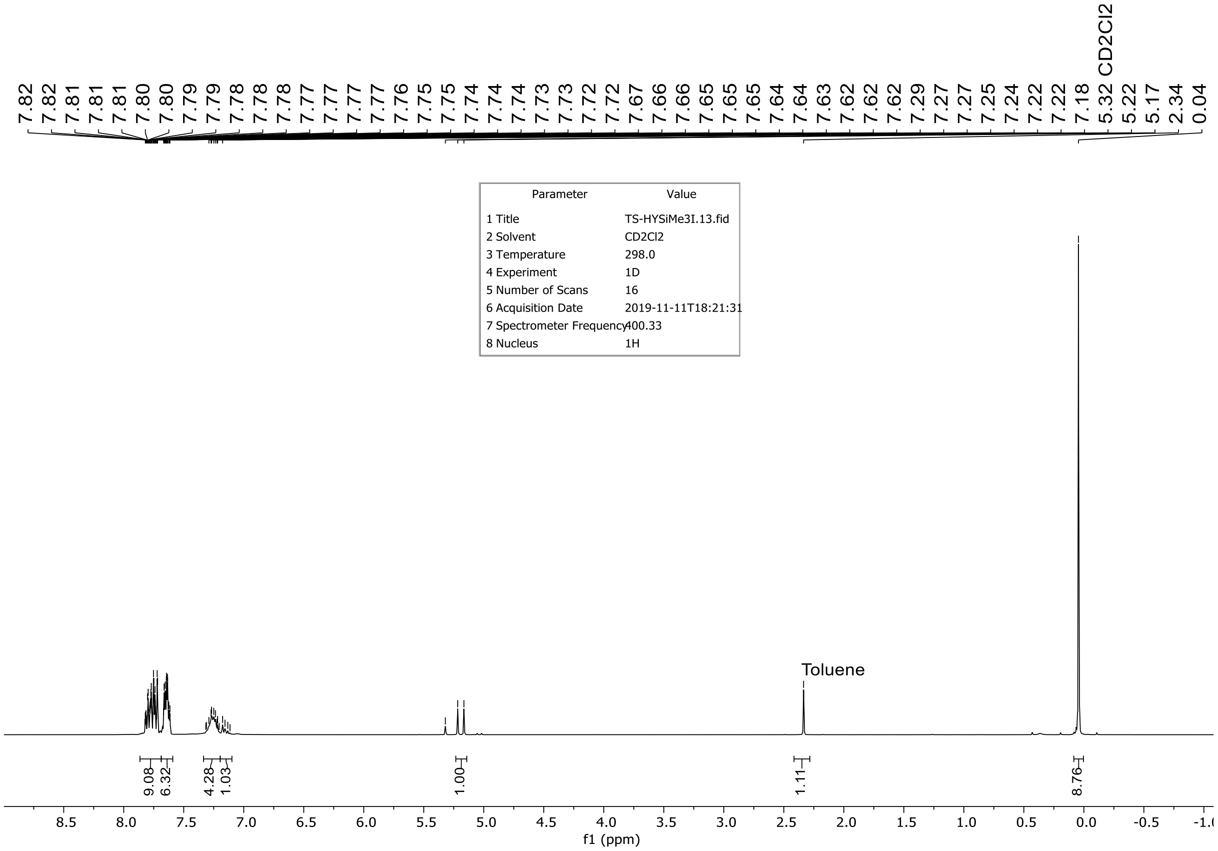

Figure S5: ^{1}H NMR spectrum of the phosphonium salt precursor of **1**.

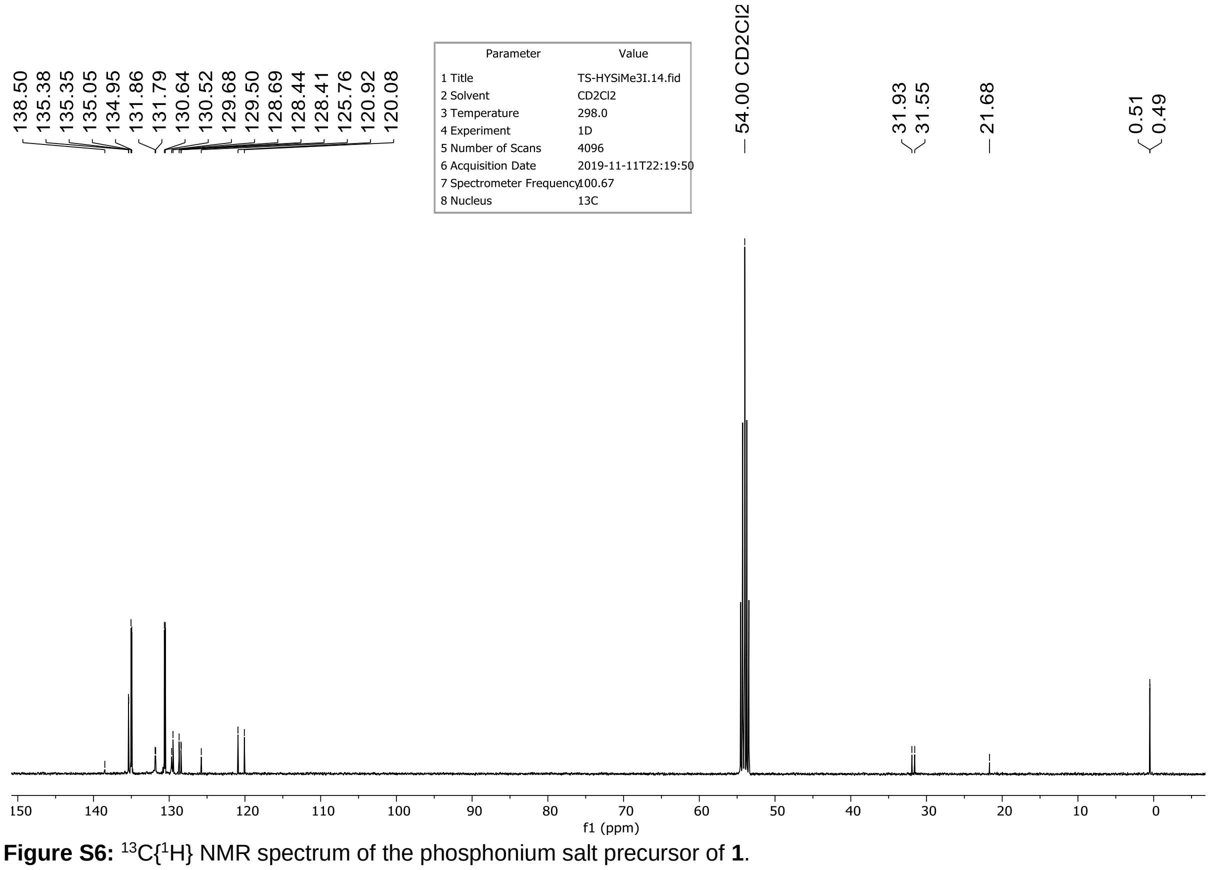

Figure S6: $^{13}C\{^{1}H\}$ NMR spectrum of the phosphonium salt precursor of **1**.

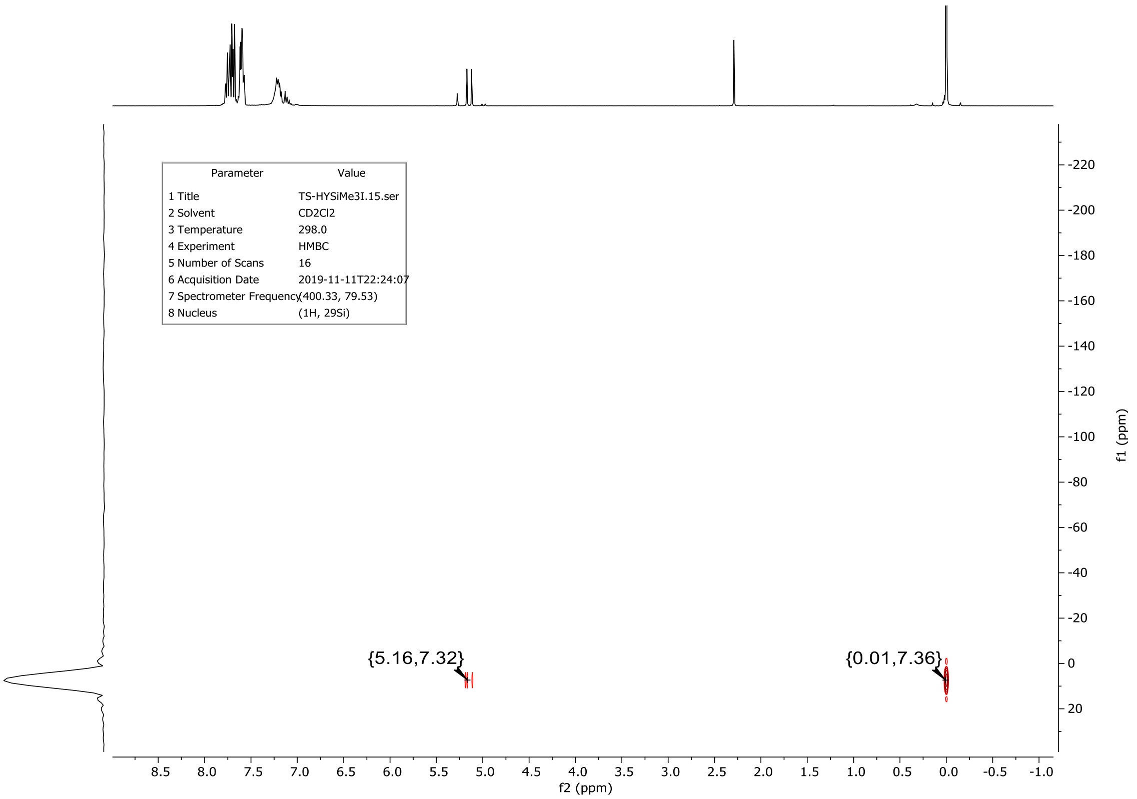

Figure S7: ^{29}Si-^{1}H NMR spectrum of the phosphonium salt precursor of **1**.

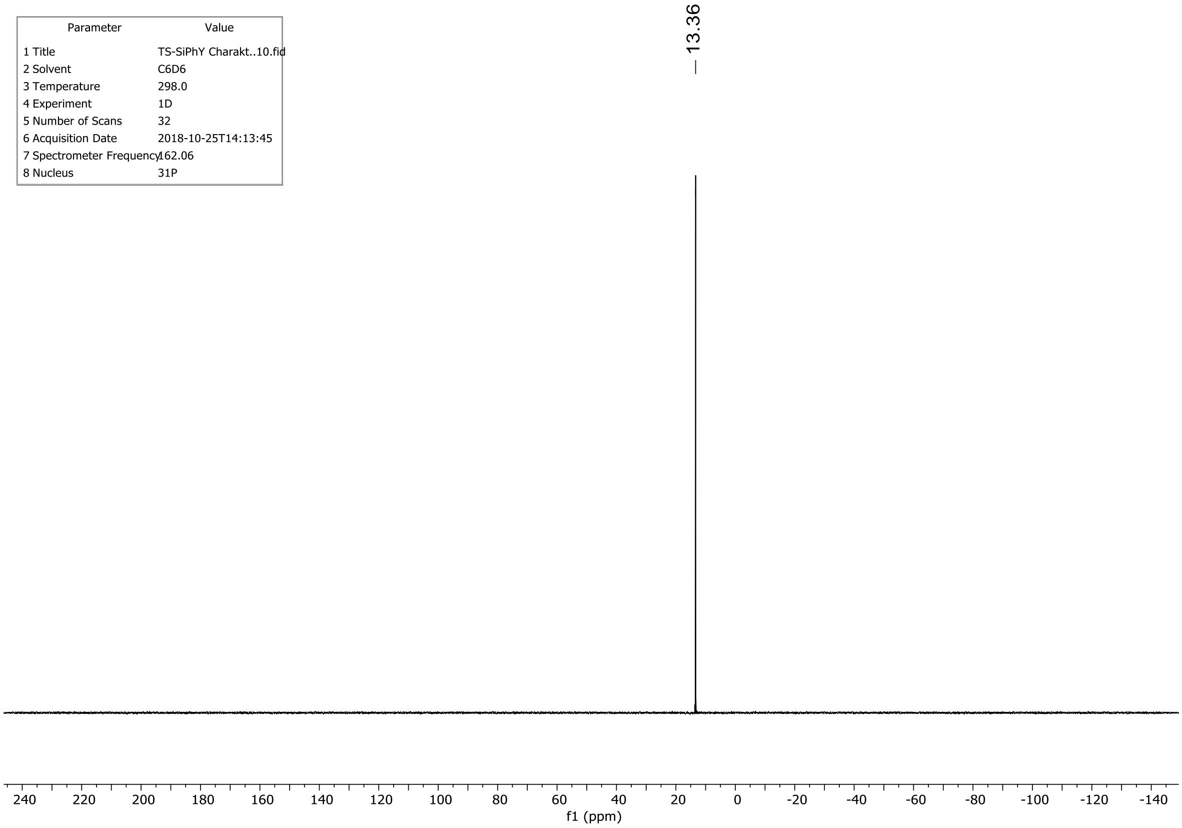

Figure S8: $^{31}P\{^{1}H\}$ NMR spectrum of **1**.

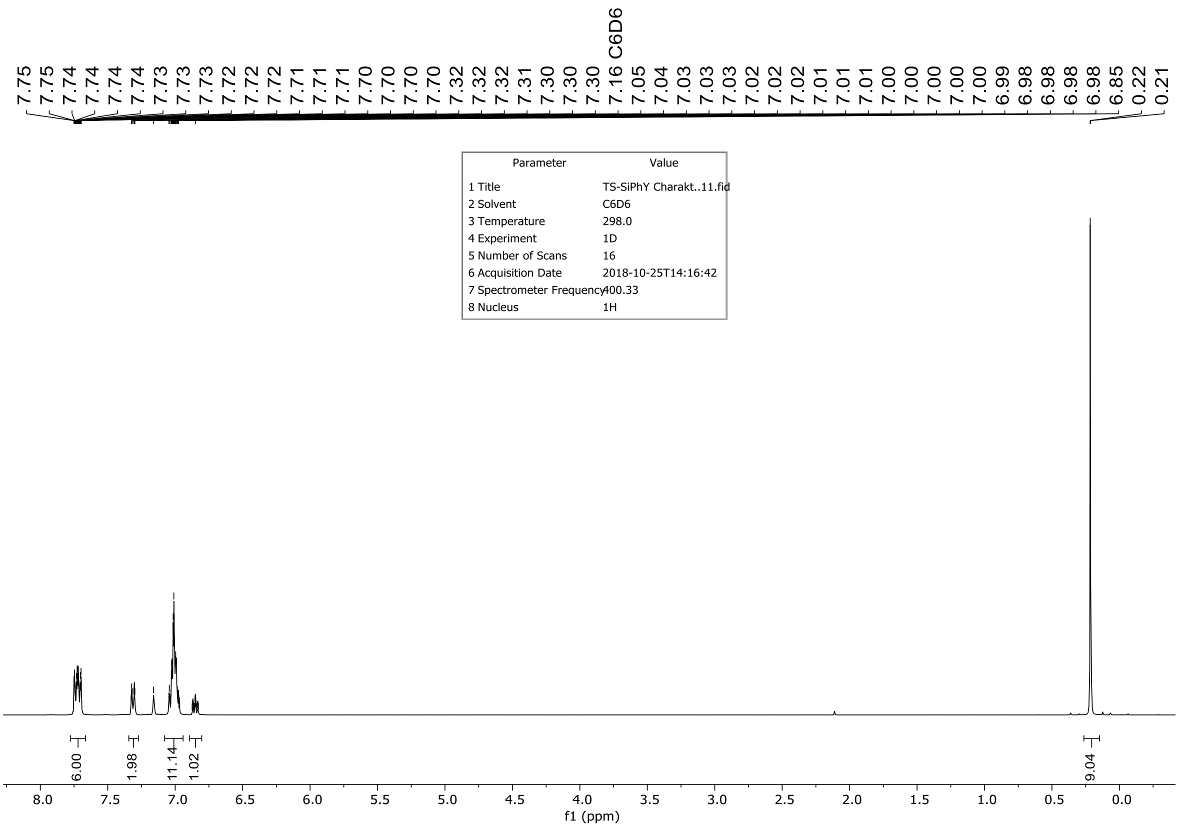

Figure S9: ^{1}H NMR spectrum of **1**.

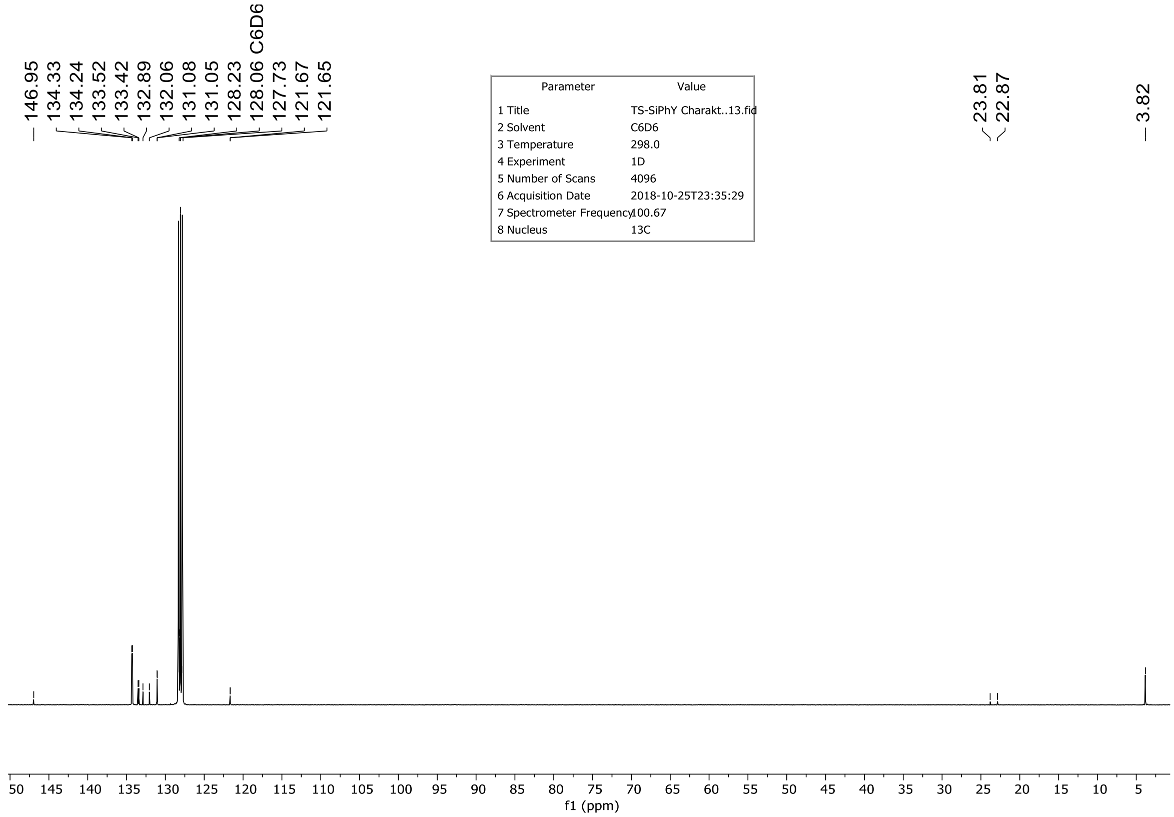

Figure S10: $^{13}C\{^{1}H\}$ NMR spectrum of **1**.

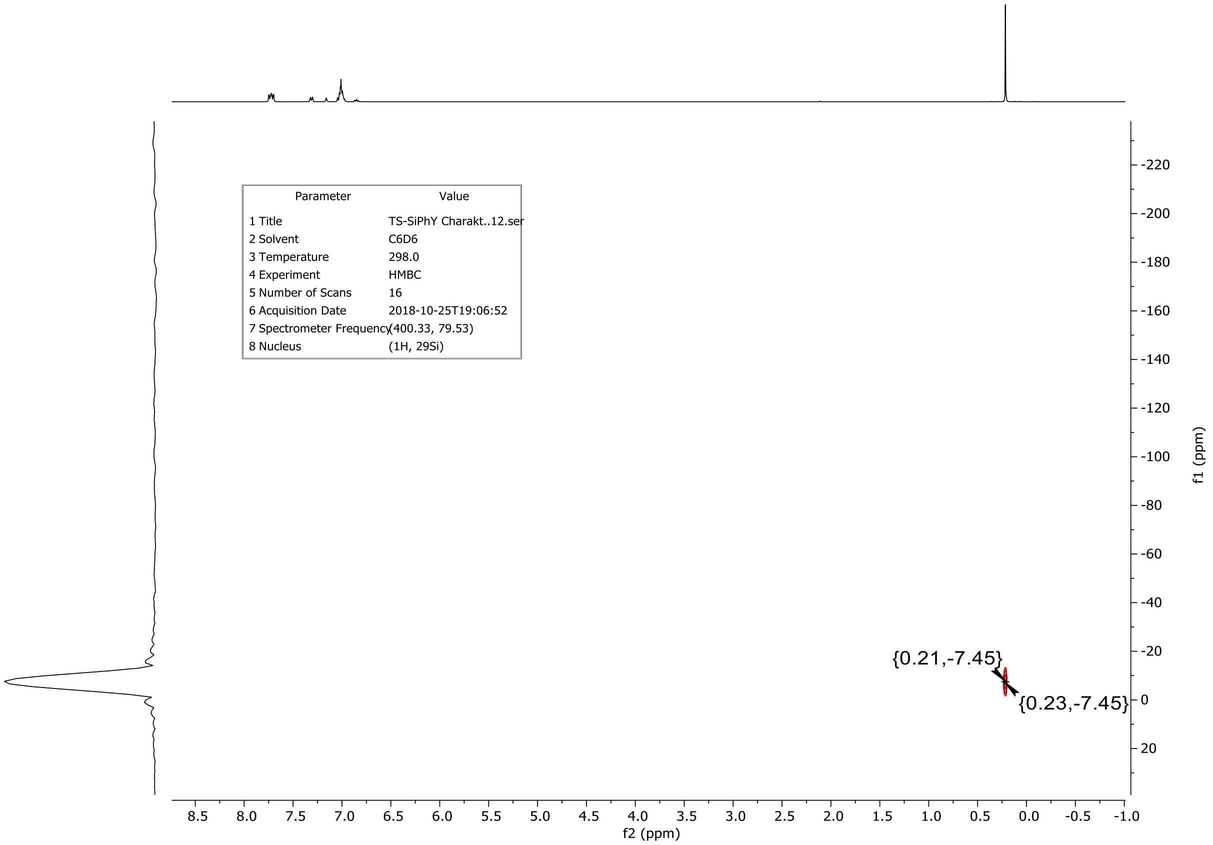

Figure S11: ^{29}Si-^{1}H NMR spectrum of **1**.

2.2 NMR spectra of the chlorophosphines and phosphenium cations

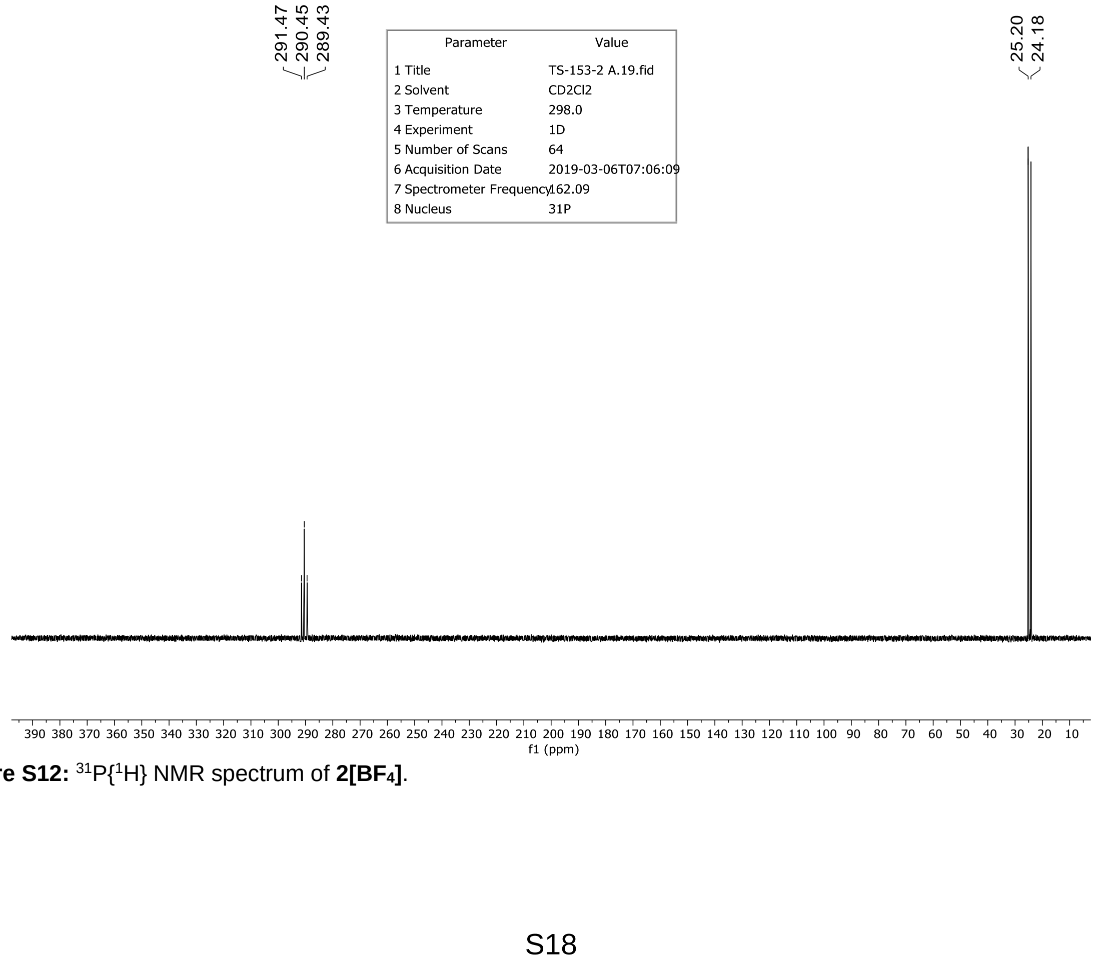

Figure S12: ^{31}P{^{1}H} NMR spectrum of **2[BF$_4$]**.

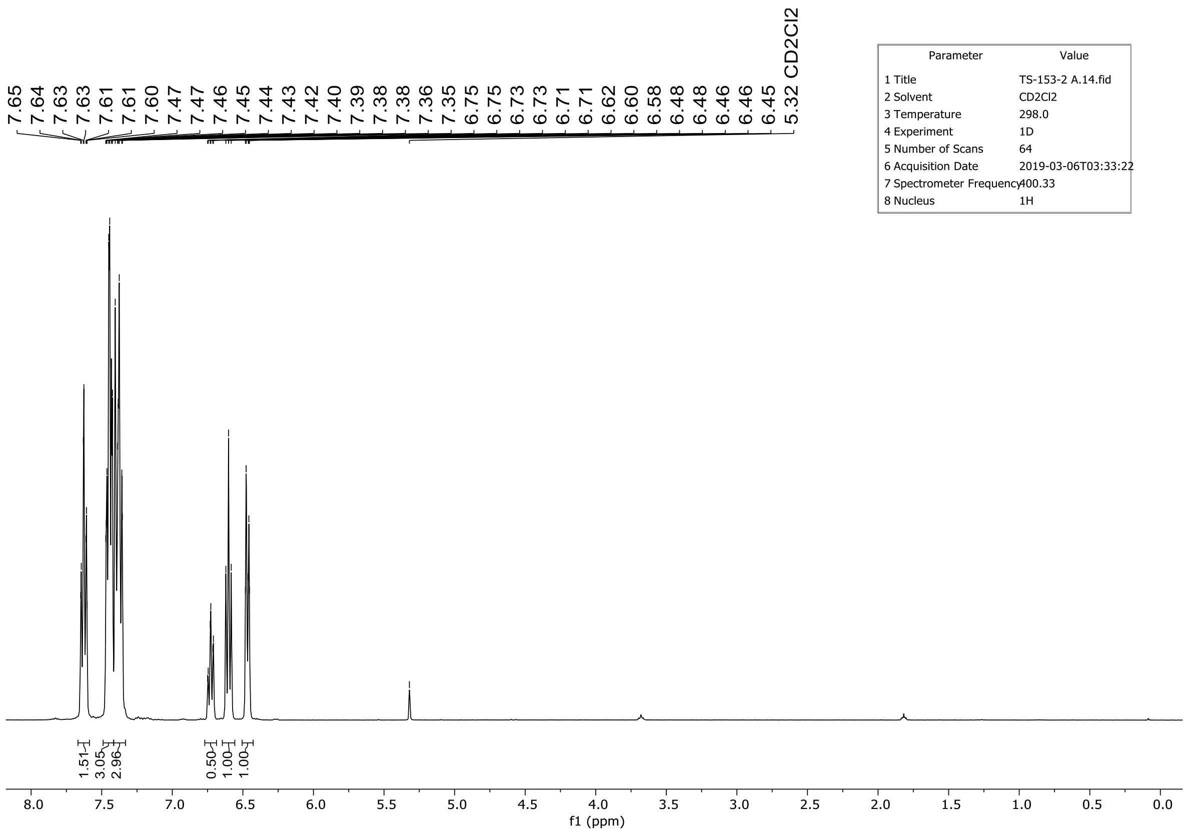

Figure S13: ^{1}H NMR spectrum of **2[BF$_4$]**.

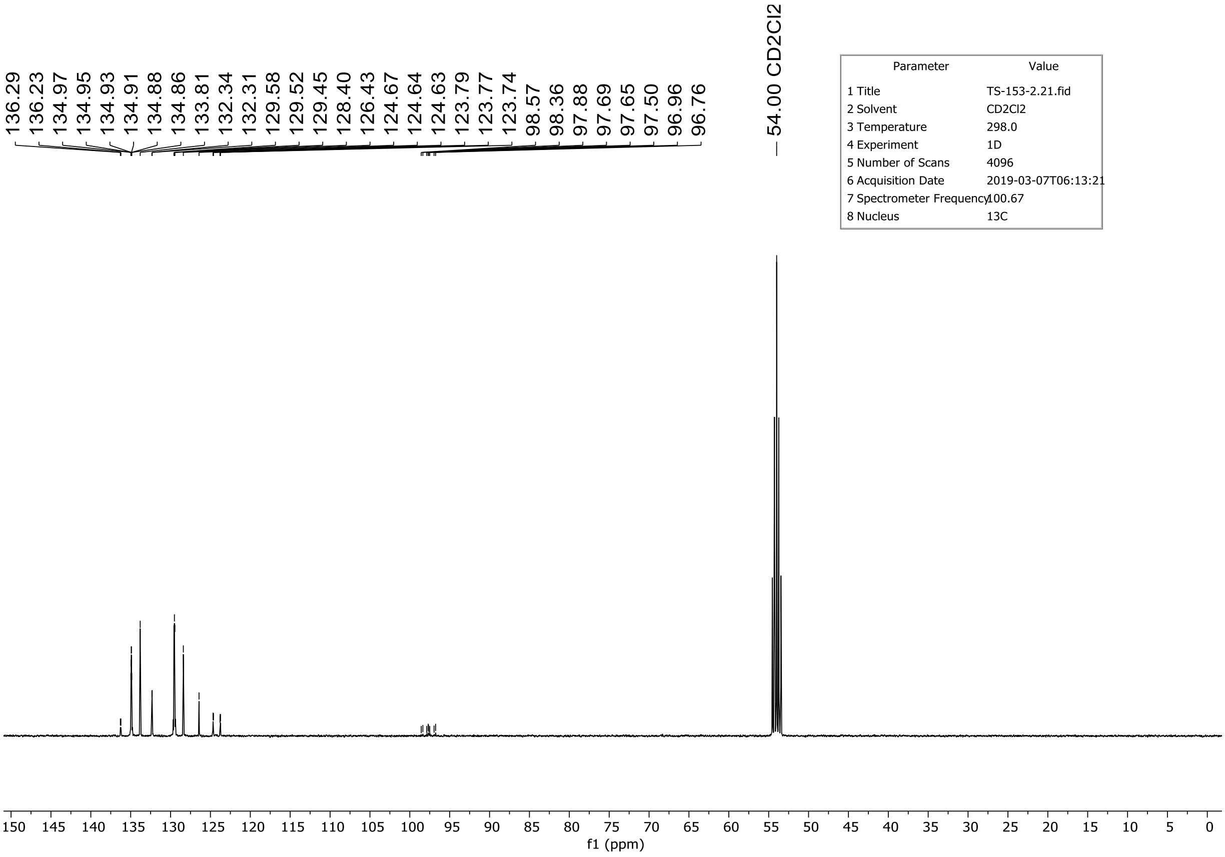

Figure S14: $^{13}C\{^{1}H\}$ NMR spectrum of **2[BF$_4$]**.

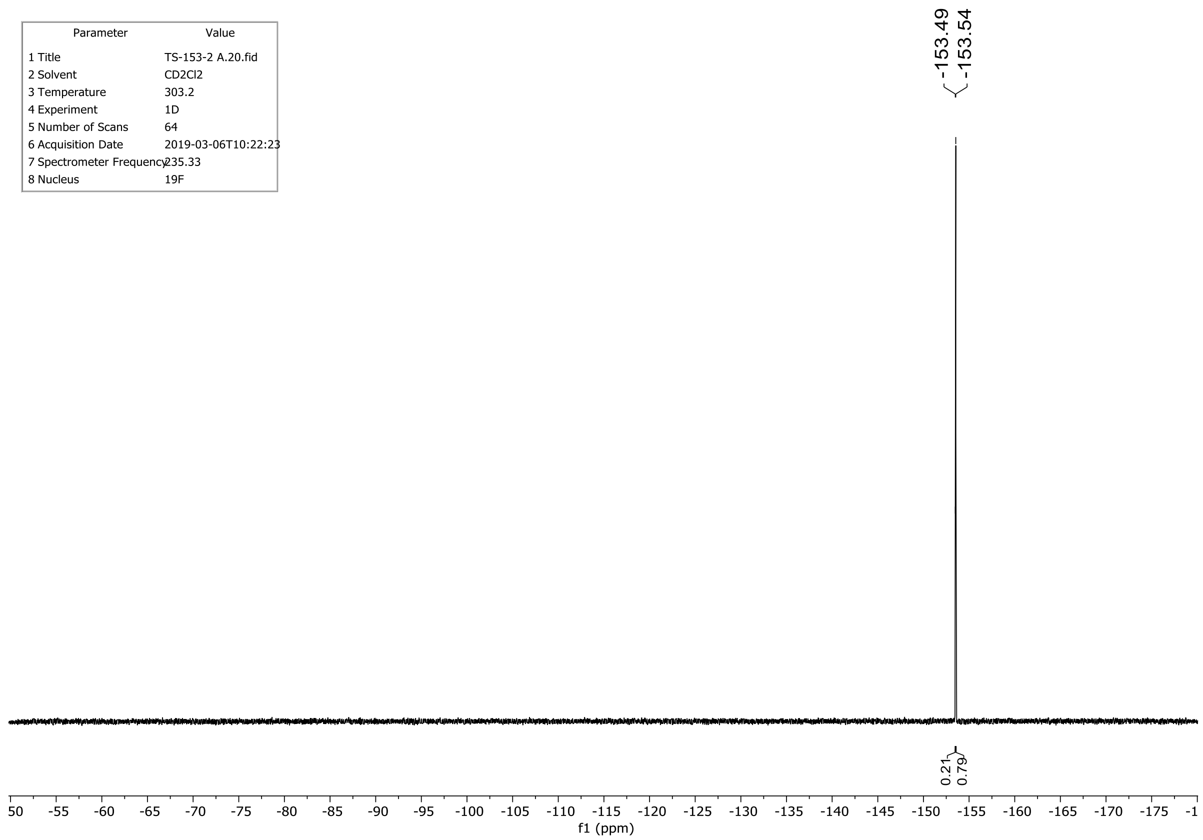

Figure S15: $^{19}F\{^{1}H\}$ NMR spectrum of **2[BF$_4$]**.

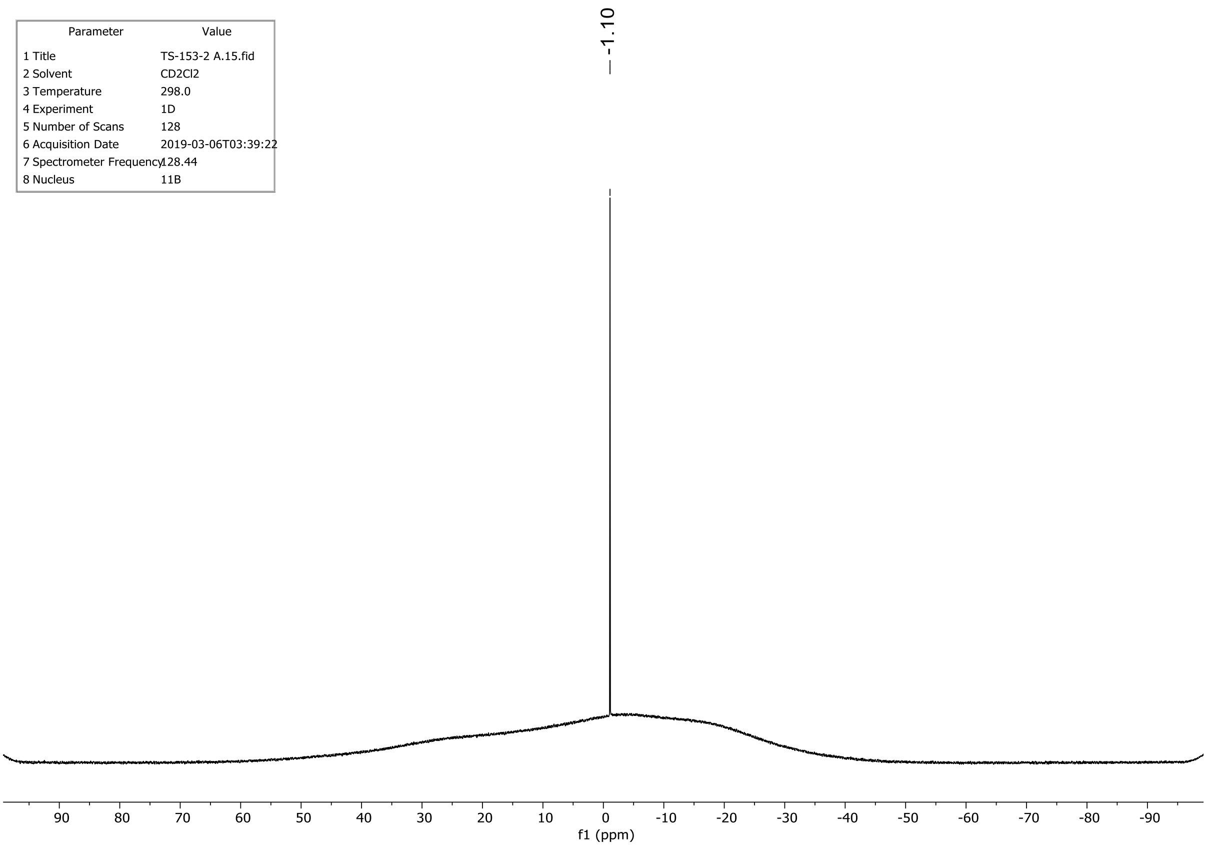

Figure S16: $^{11}B\{^{1}H\}$ NMR spectrum of **2[BF$_4$]**.

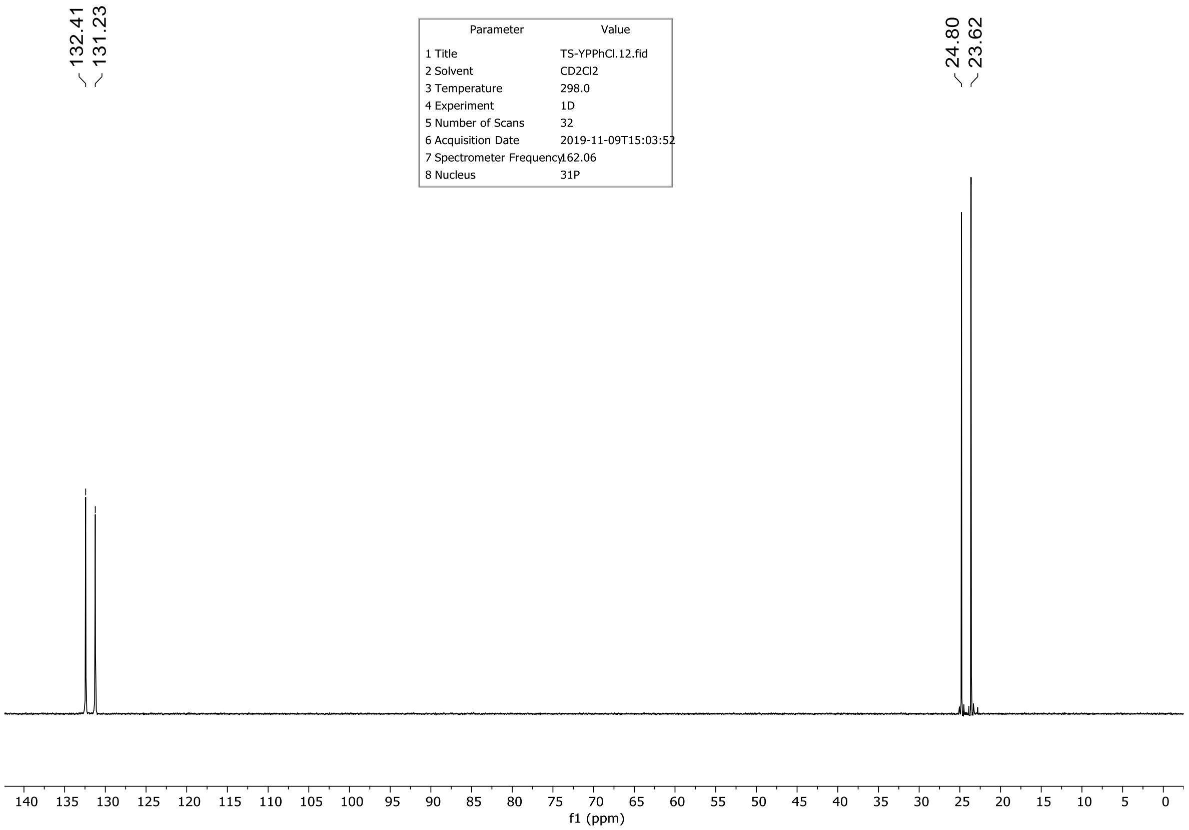

Figure S17: $^{31}P\{^{1}H\}$ NMR spectrum of **3a**.

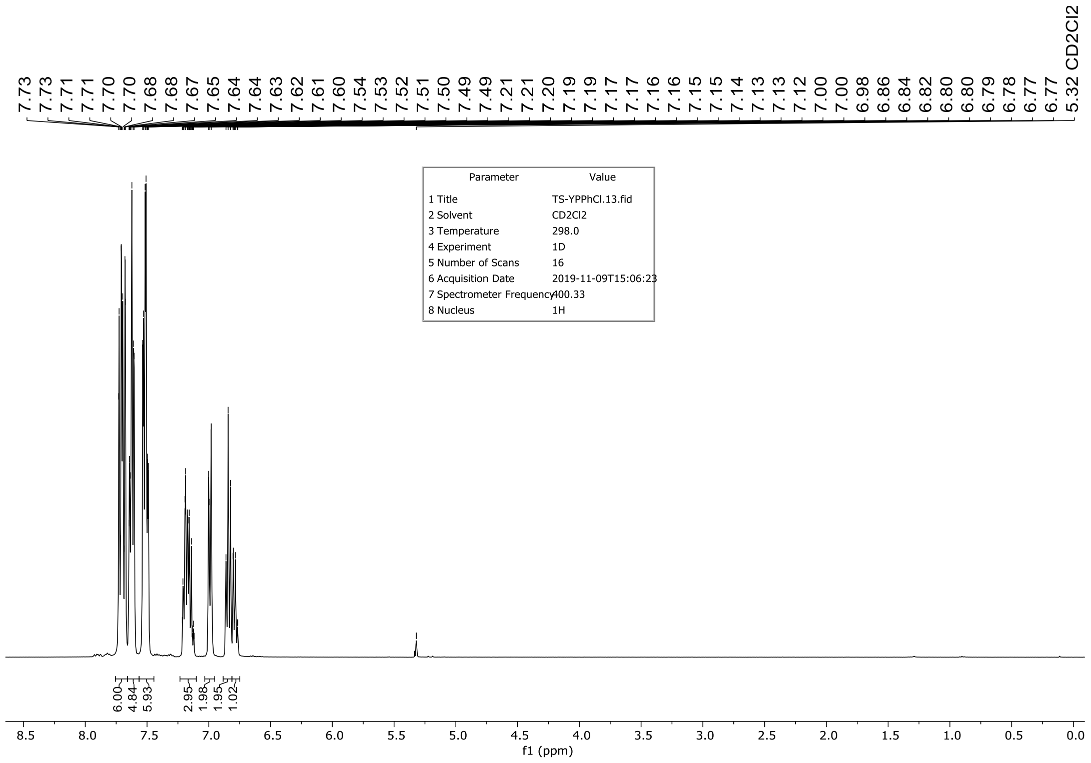

Figure S18: ^{1}H NMR spectrum of **3a**.

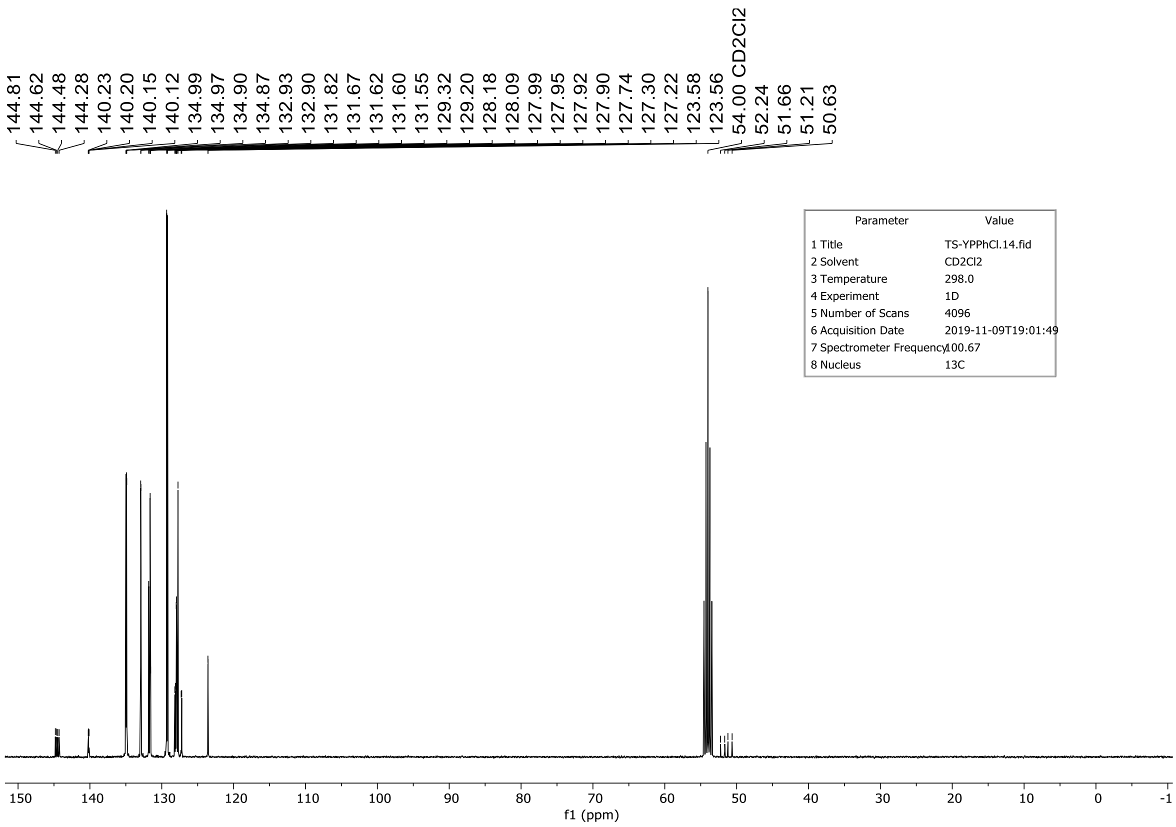

Figure S19: $^{13}C\{^{1}H\}$ NMR spectrum of **3a**.

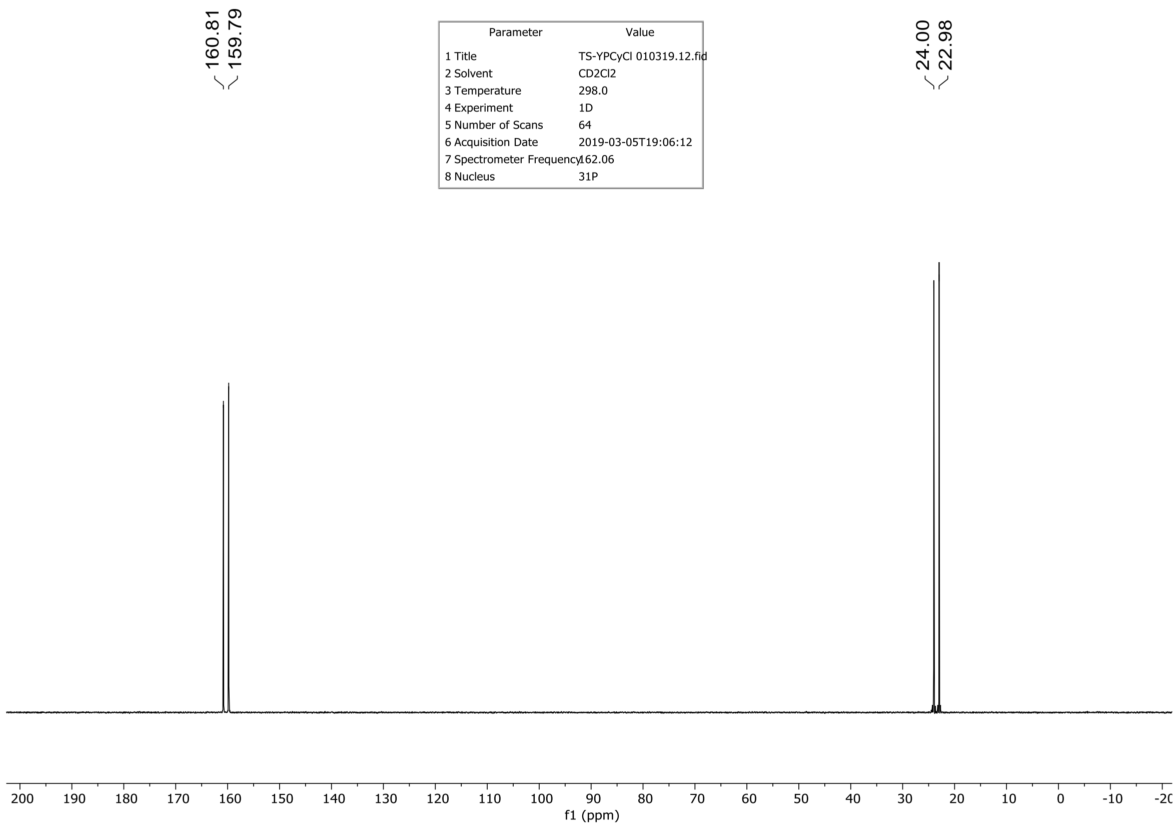

Figure S20: $^{31}P\{^{1}H\}$ NMR spectrum of **3b**.

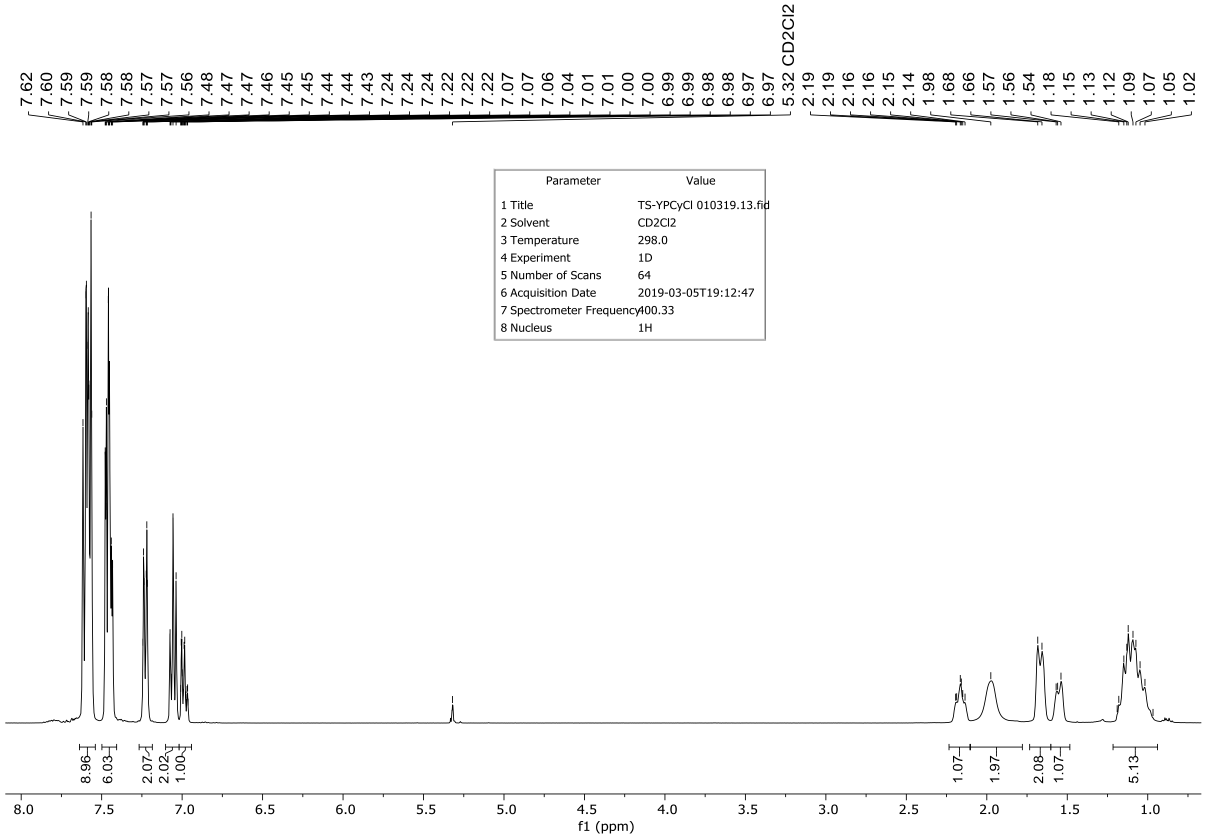

Figure S21: ^{1}H NMR spectrum of **3b**.

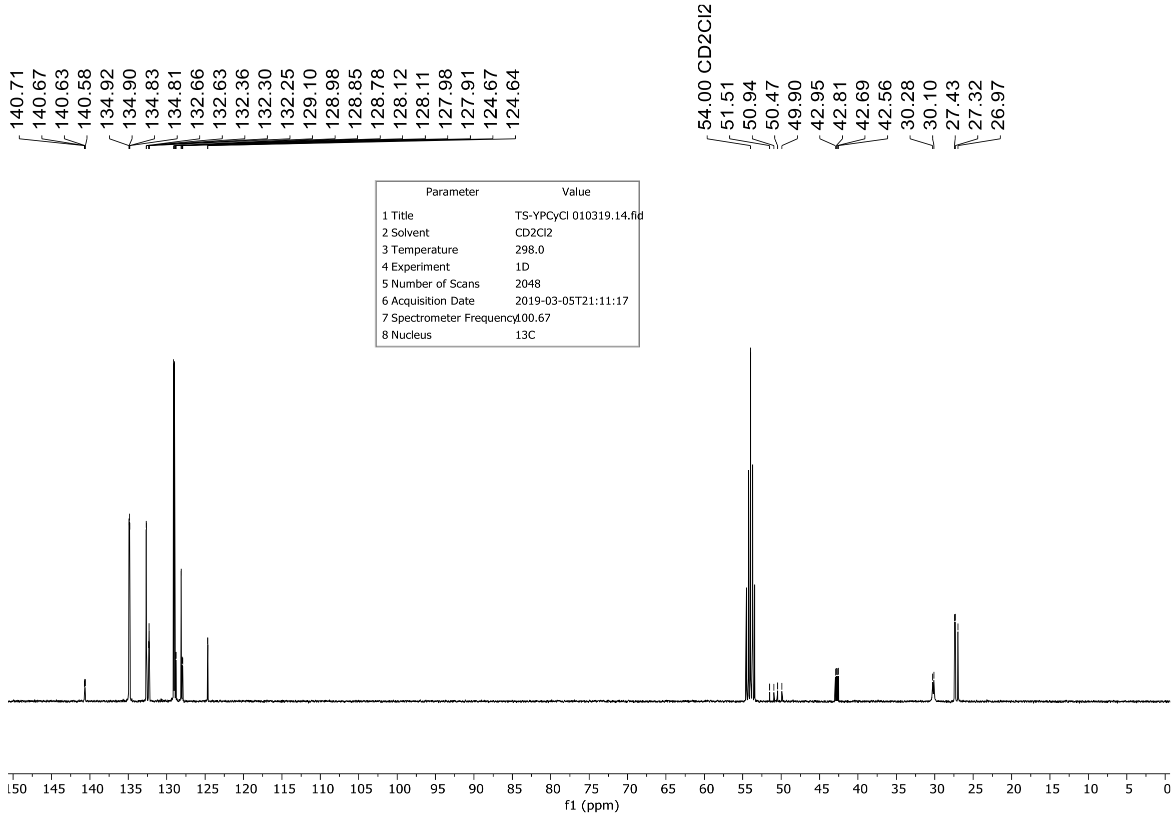

Figure S22: $^{13}C\{^{1}H\}$ NMR spectrum of **3b**.

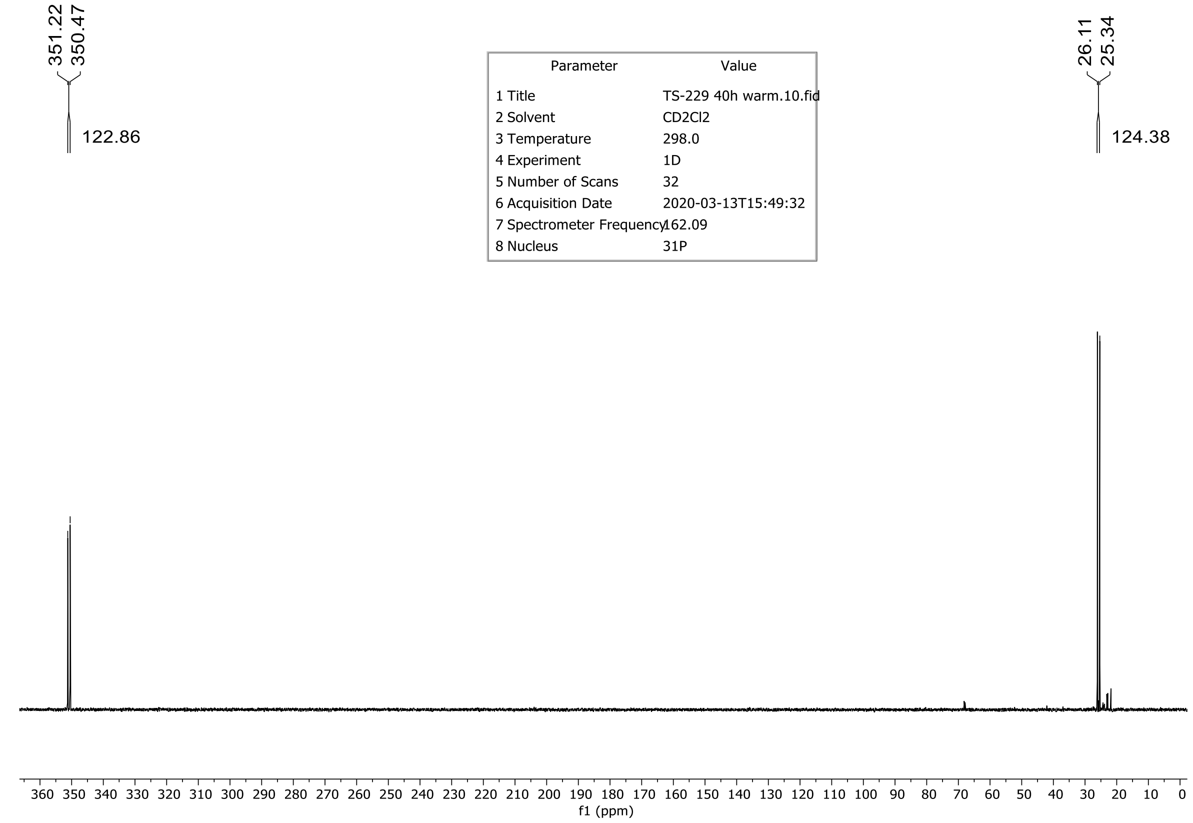

Figure S23: $^{31}P\{^{1}H\}$ NMR spectrum of **4a**.

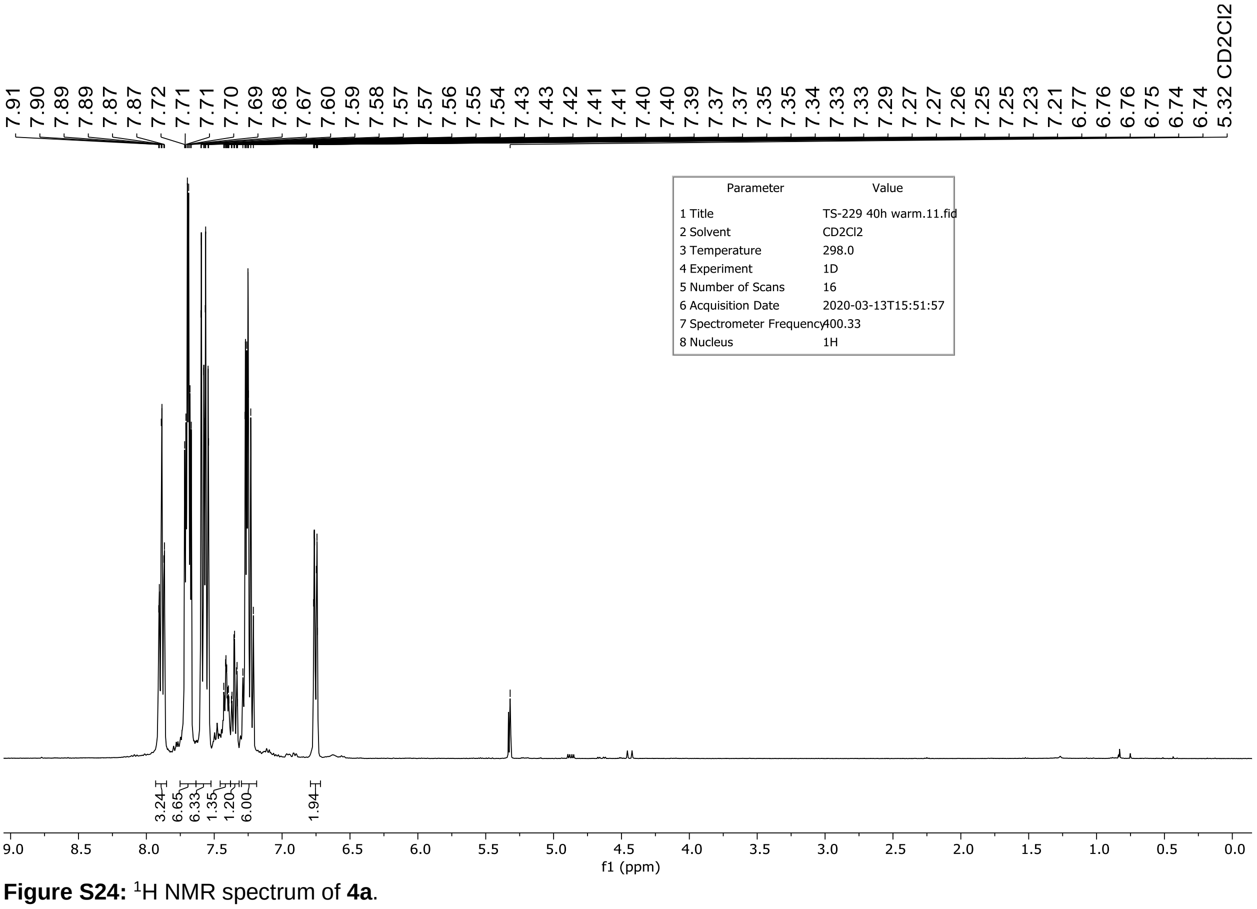

Figure S24: ^{1}H NMR spectrum of **4a**.

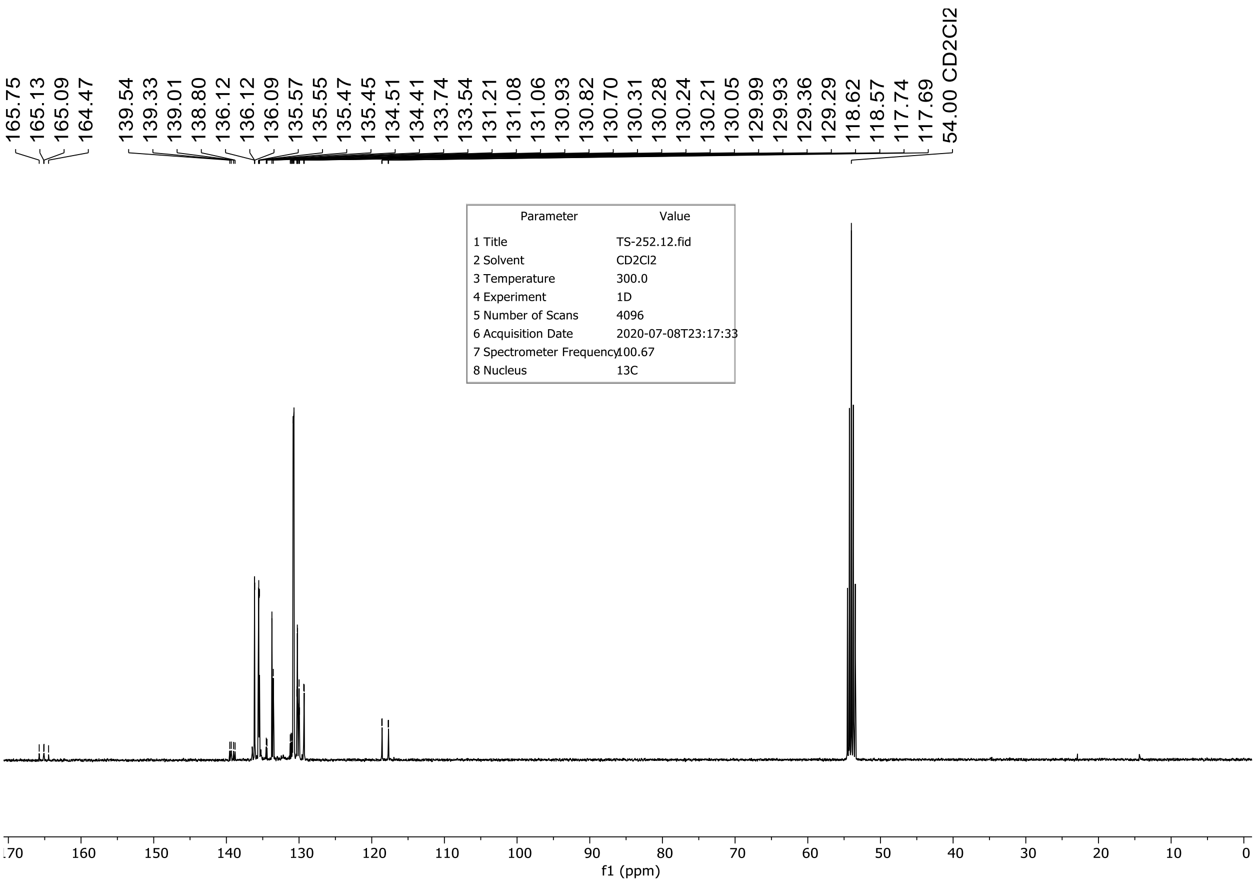

Figure S25: $^{13}C\{^{1}H\}$ NMR spectrum of **4a**.

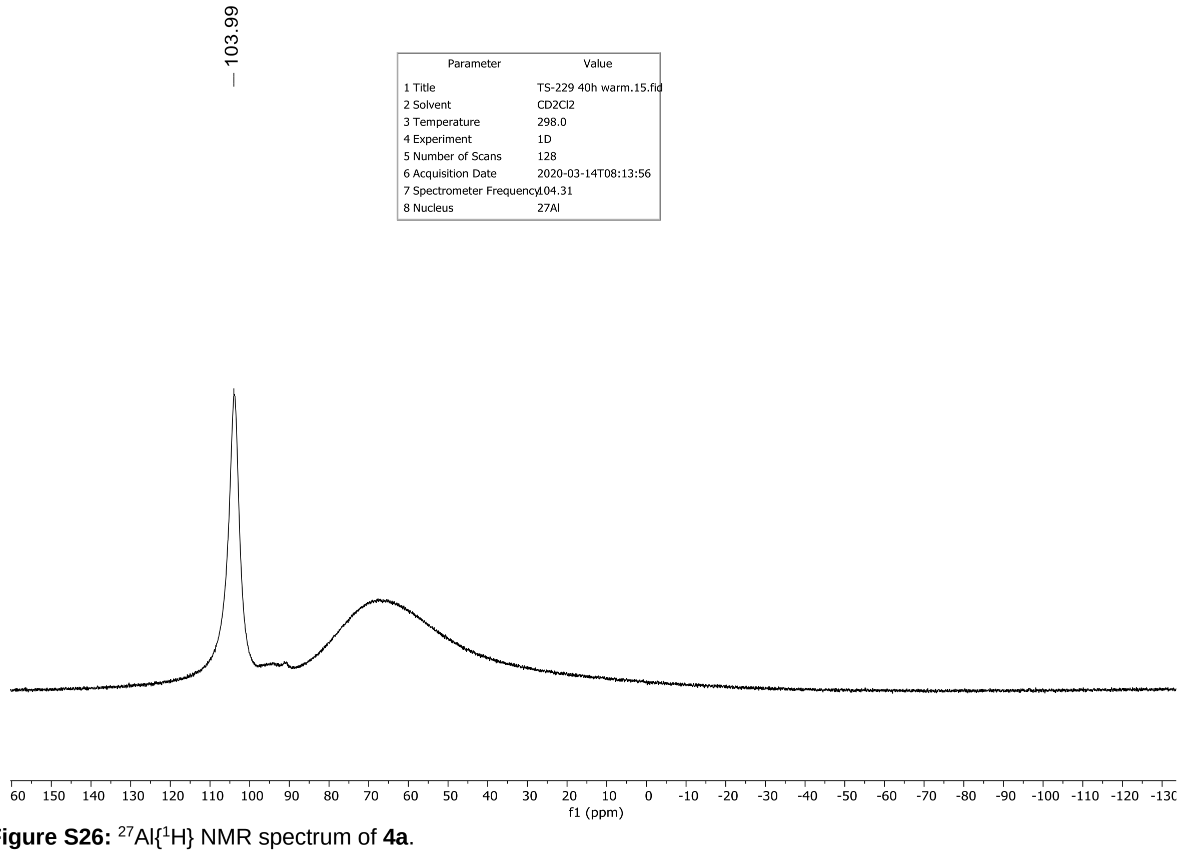

Figure S26: $^{27}Al\{^{1}H\}$ NMR spectrum of **4a**.

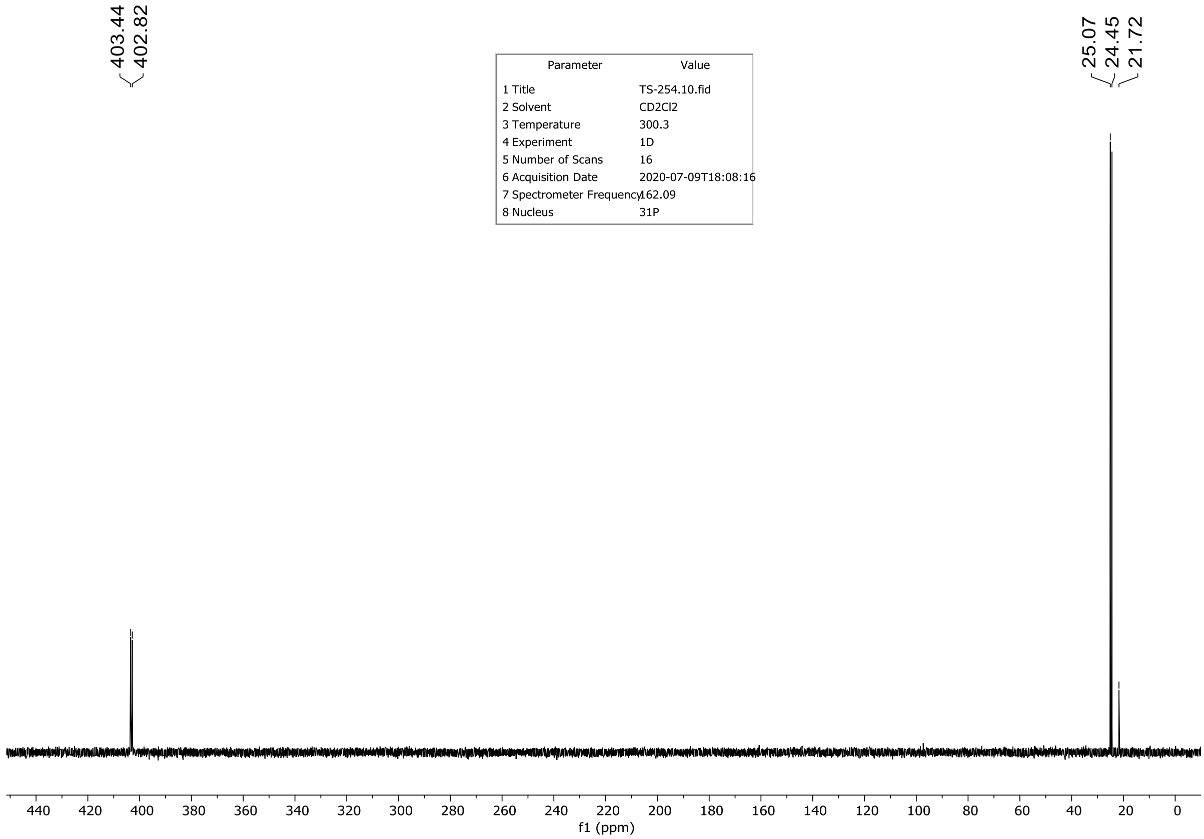

Figure S27: $^{31}P\{^{1}H\}$ NMR spectrum of **4b**.

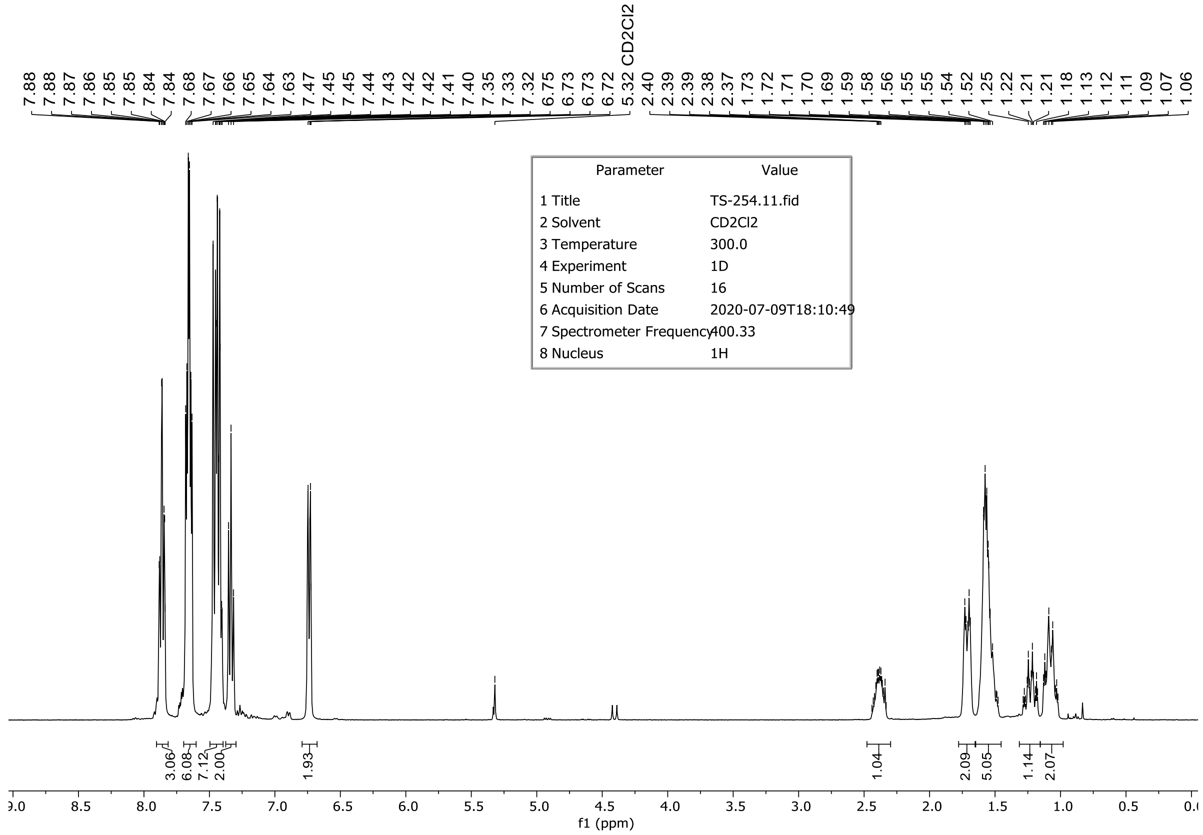

Figure S28: ^{1}H NMR spectrum of **4b**.

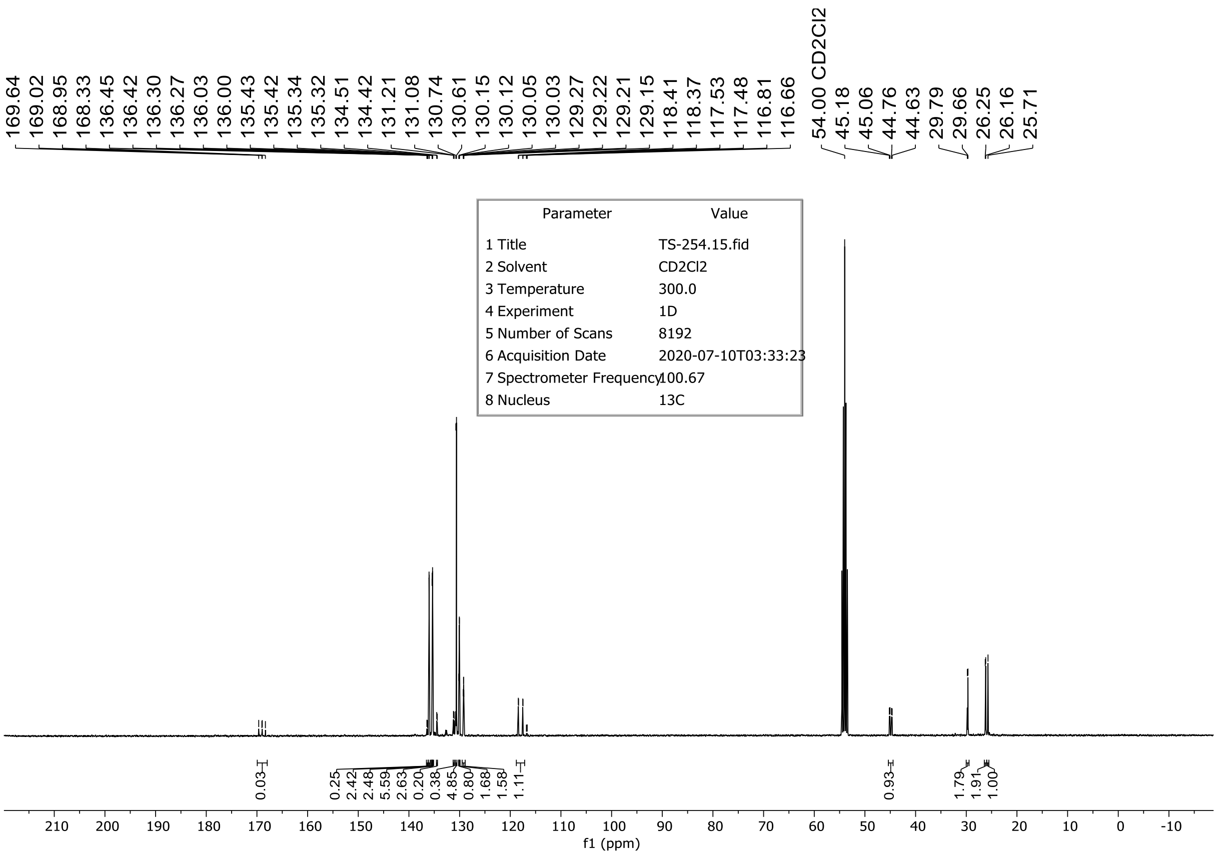

Figure S29: $^{13}C\{^1H\}$ NMR spectrum of **4b**.

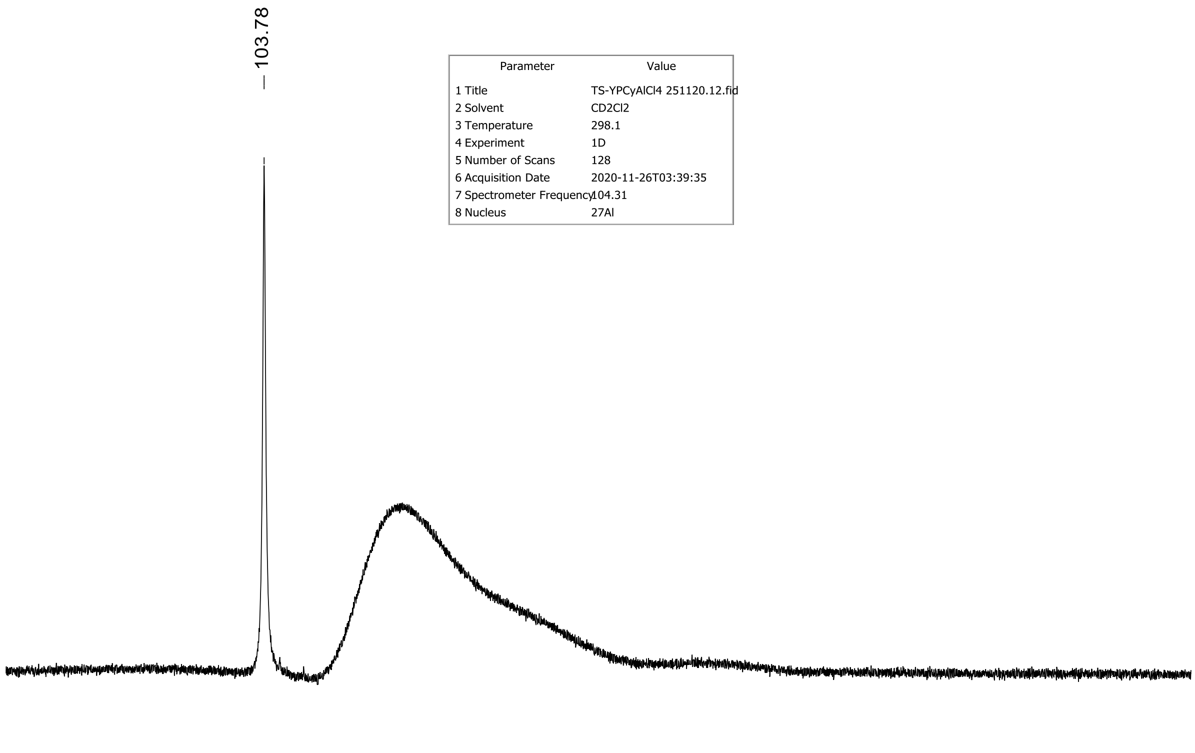

Figure S30: $^{27}Al\{^1H\}$ NMR spectrum of **4b**.

2.3 NMR spectra of the gold complexes

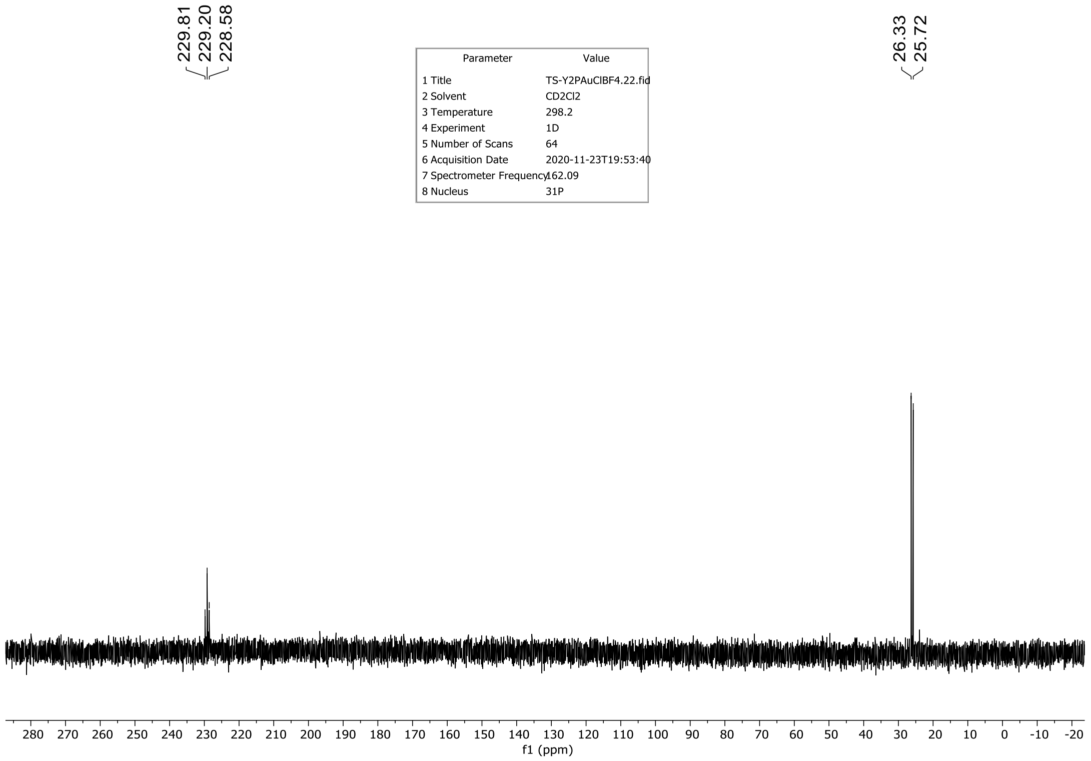

Figure S31: $^{31}P\{^{1}H\}$ NMR spectrum of **[Y_2PAuCl]BF_4**.

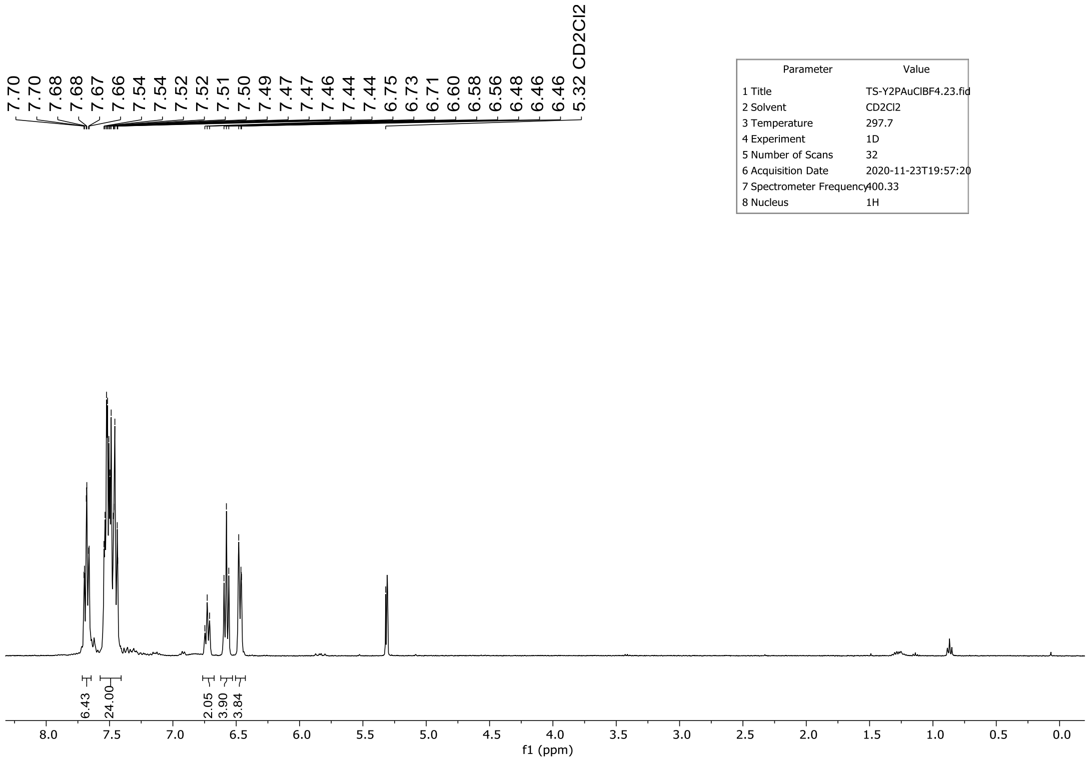

Figure S32: ^{1}H NMR spectrum of **[Y_2PAuCl]BF_4**.

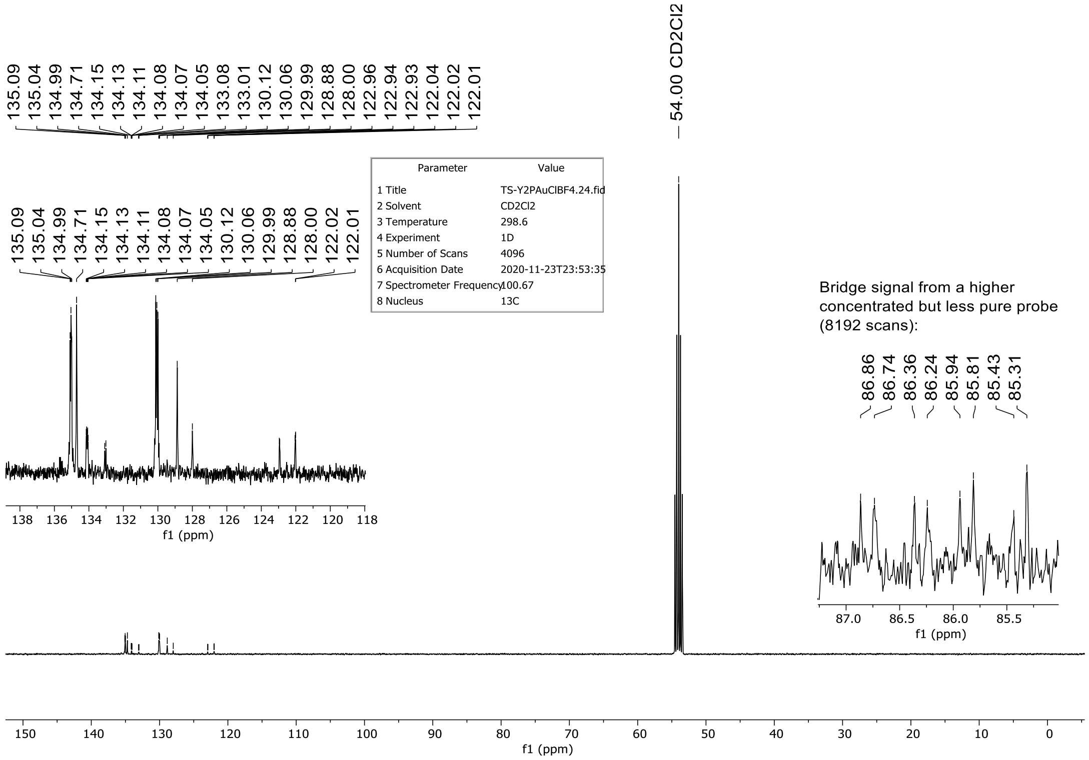

Figure S33: $^{13}C\{^{1}H\}$ NMR spectrum of **$[Y_2PAuCl]BF_4$**.

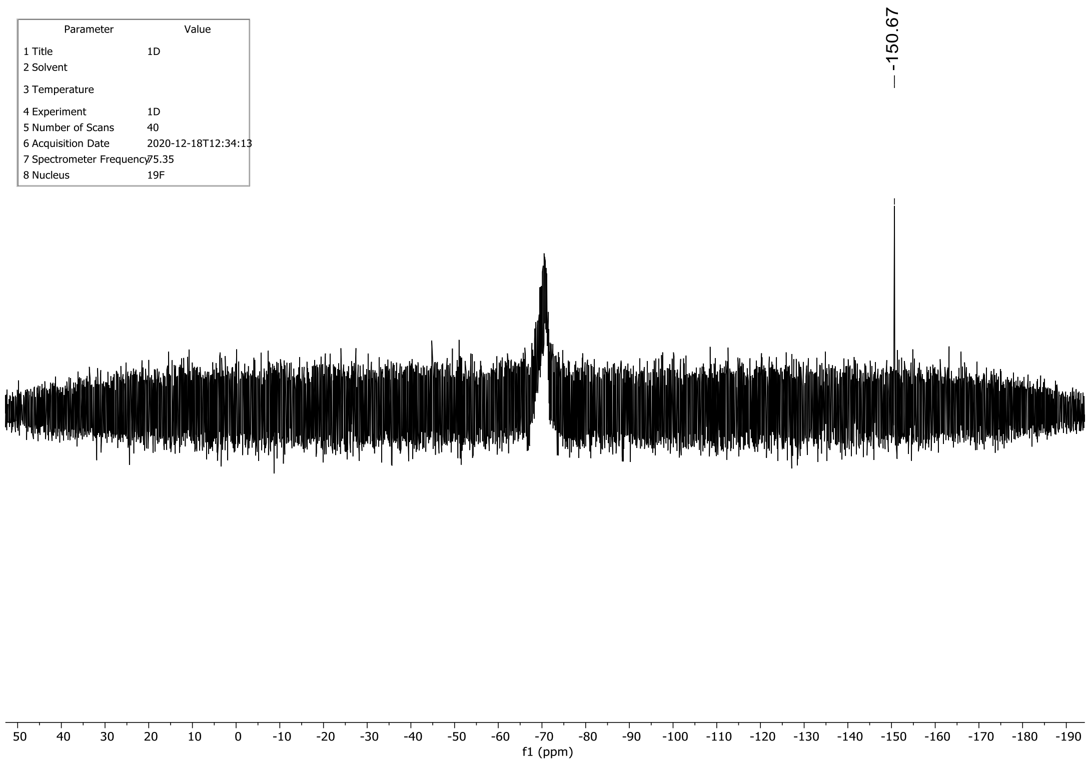

Figure S34: $^{19}F\{^{1}H\}$ NMR spectrum of **$[Y_2PAuCl]BF_4$**.

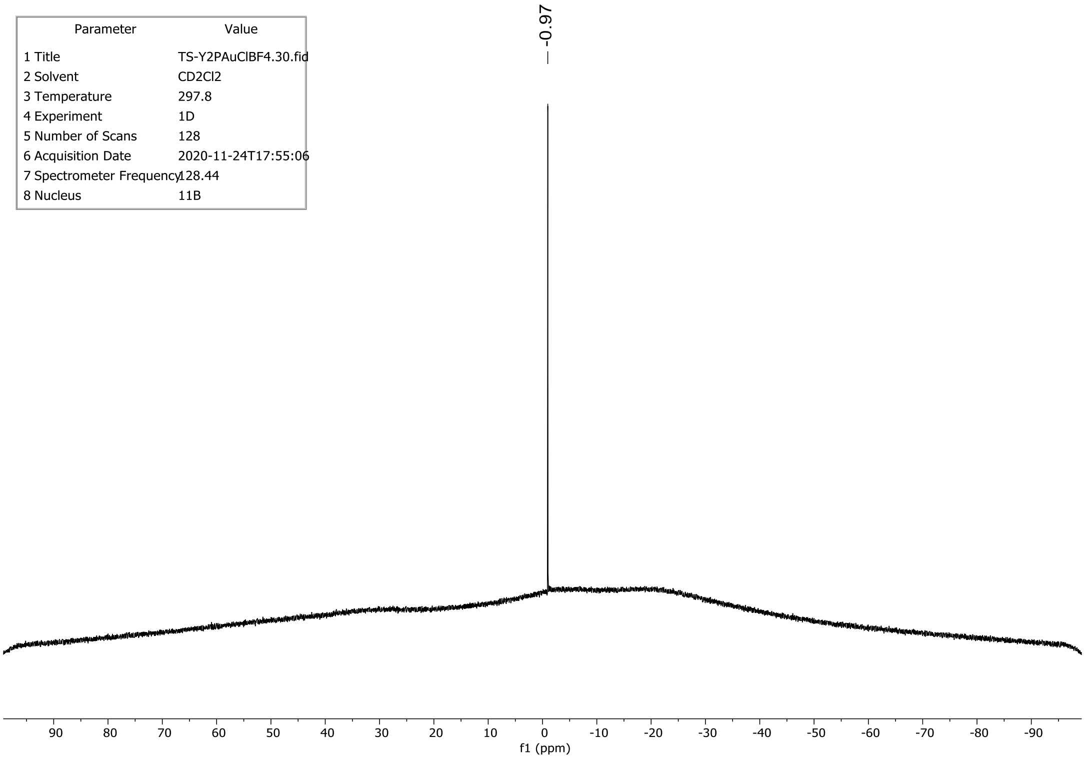

Figure S35: $^{11}B\{^{1}H\}$ NMR spectrum of **$[Y_2PAuCl]BF_4$**.

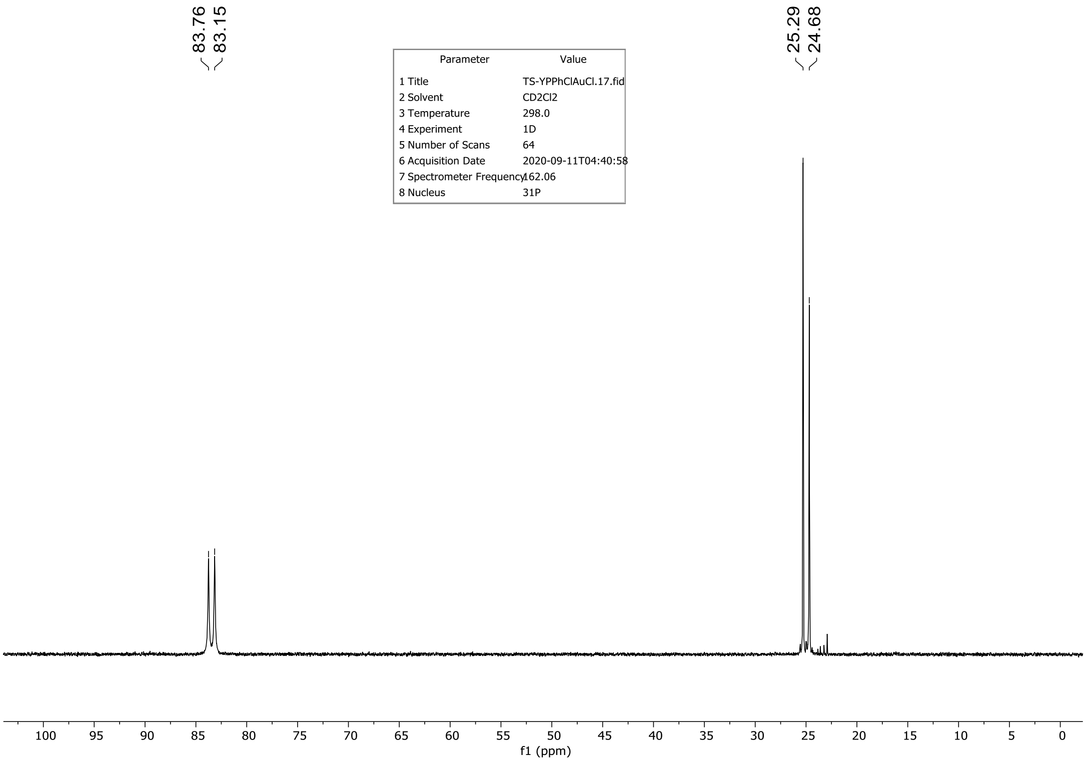

Figure S36: $^{31}P\{^{1}H\}$ NMR spectrum of **YPPhClAuCl**.

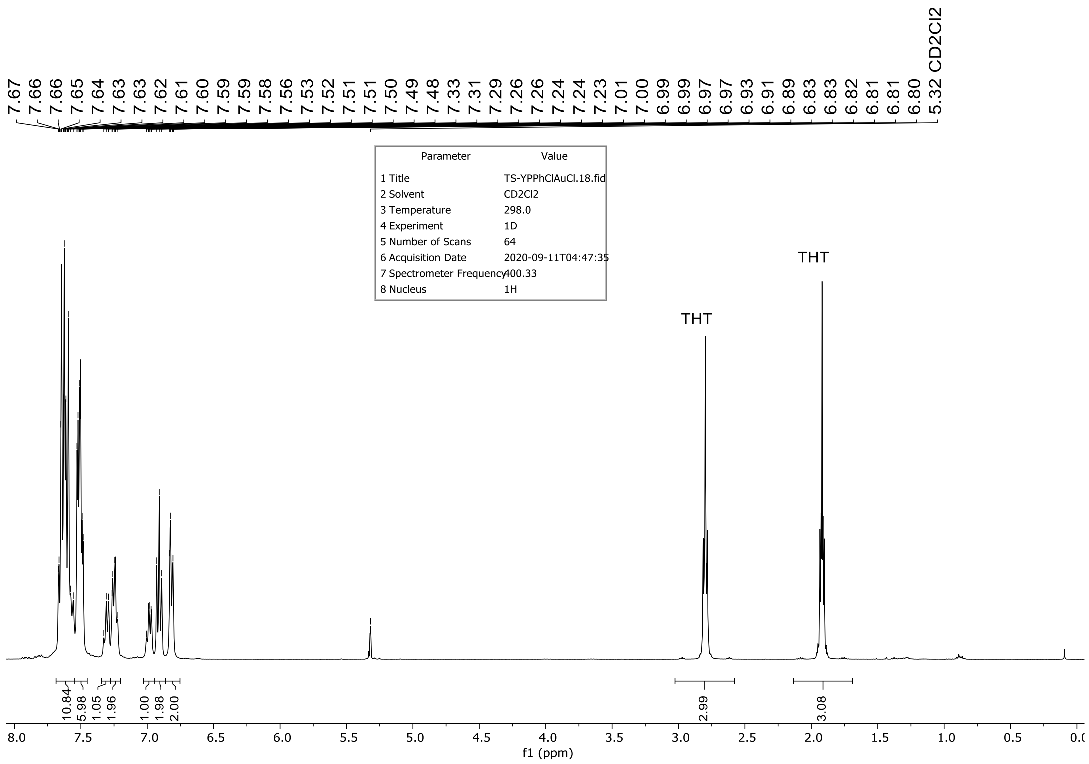

Figure S37: ^{1}H NMR spectrum of **YPPhClAuCl**.

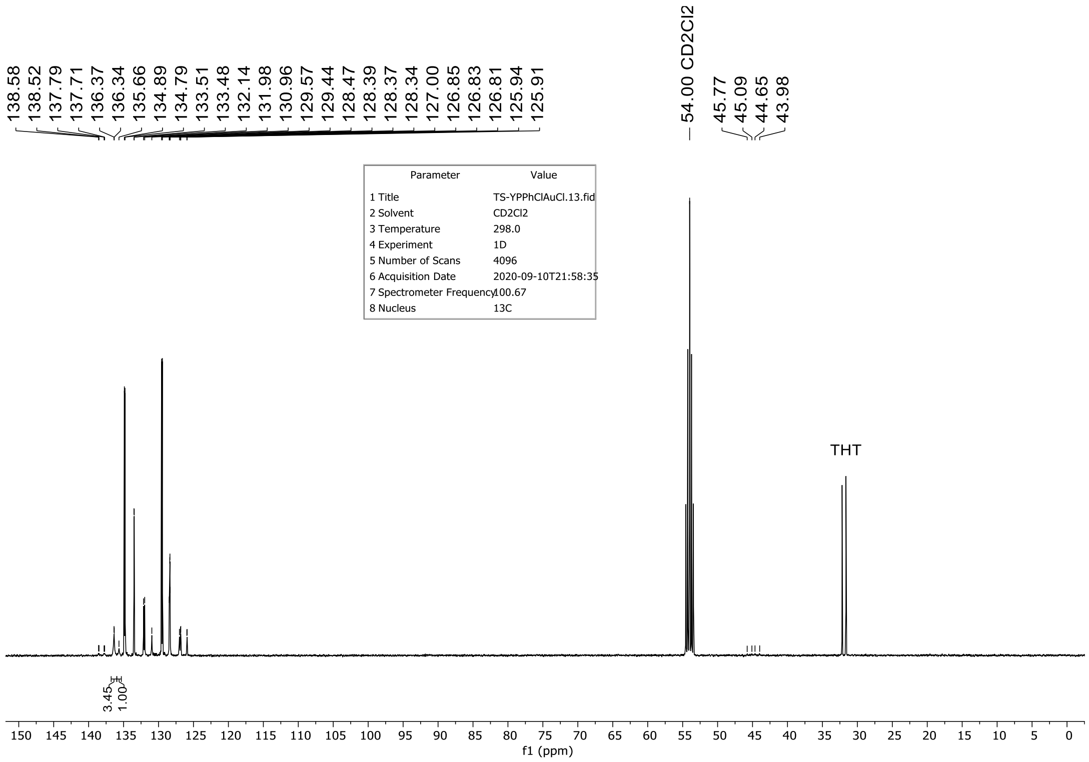

Figure S38: ^{13}C{^{1}H} NMR spectrum of **YPPhClAuCl**.

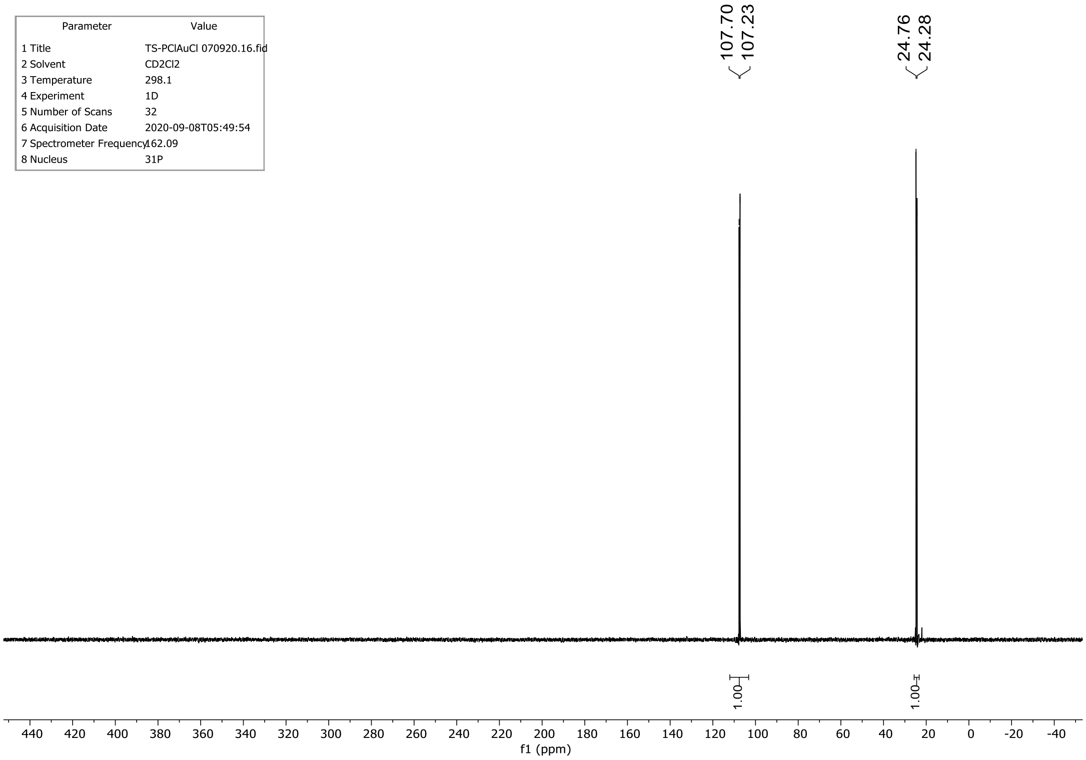

Figure S39: $^{31}P\{^1H\}$ NMR spectrum of **YPCyClAuCl**.

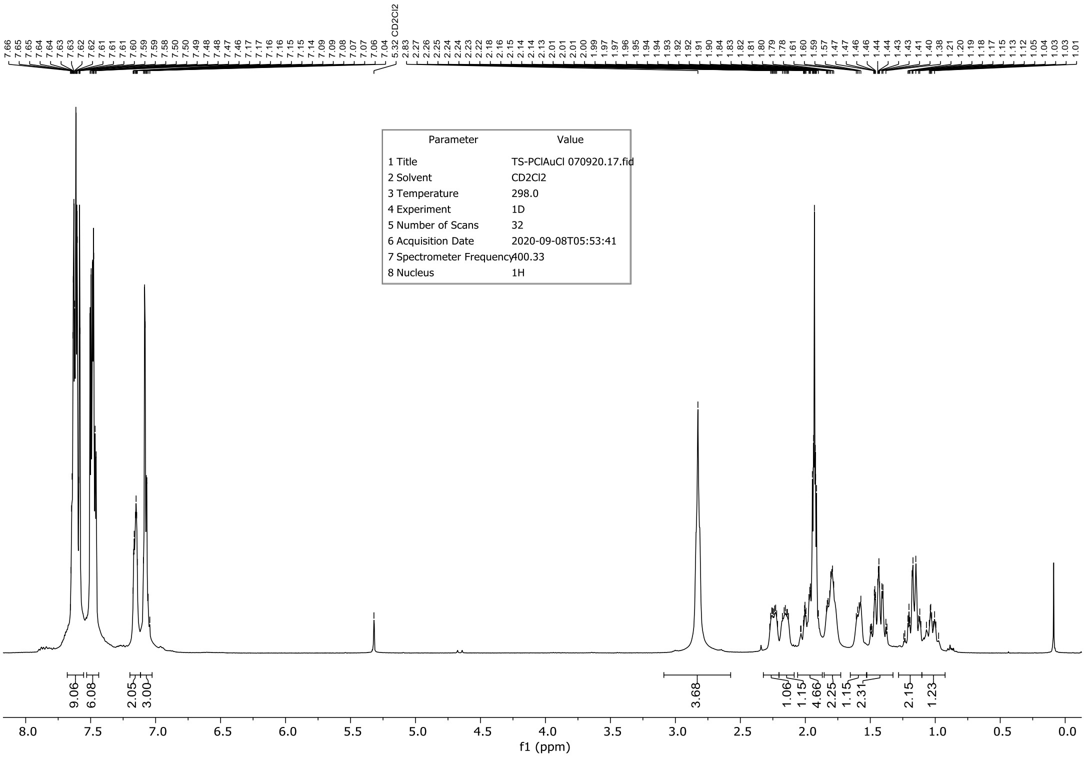

Figure S40: ^1H NMR spectrum of **YPCyClAuCl**.

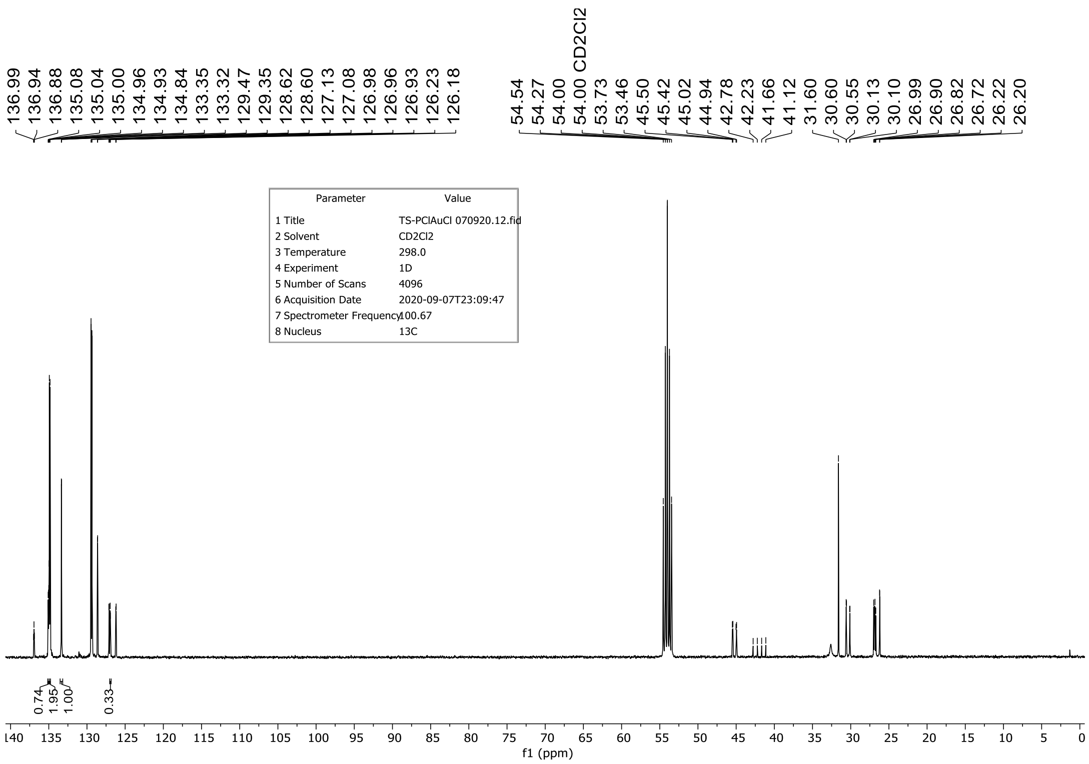

Figure S41: $^{13}C\{^1H\}$ NMR spectrum of **YPCyClAuCl**.

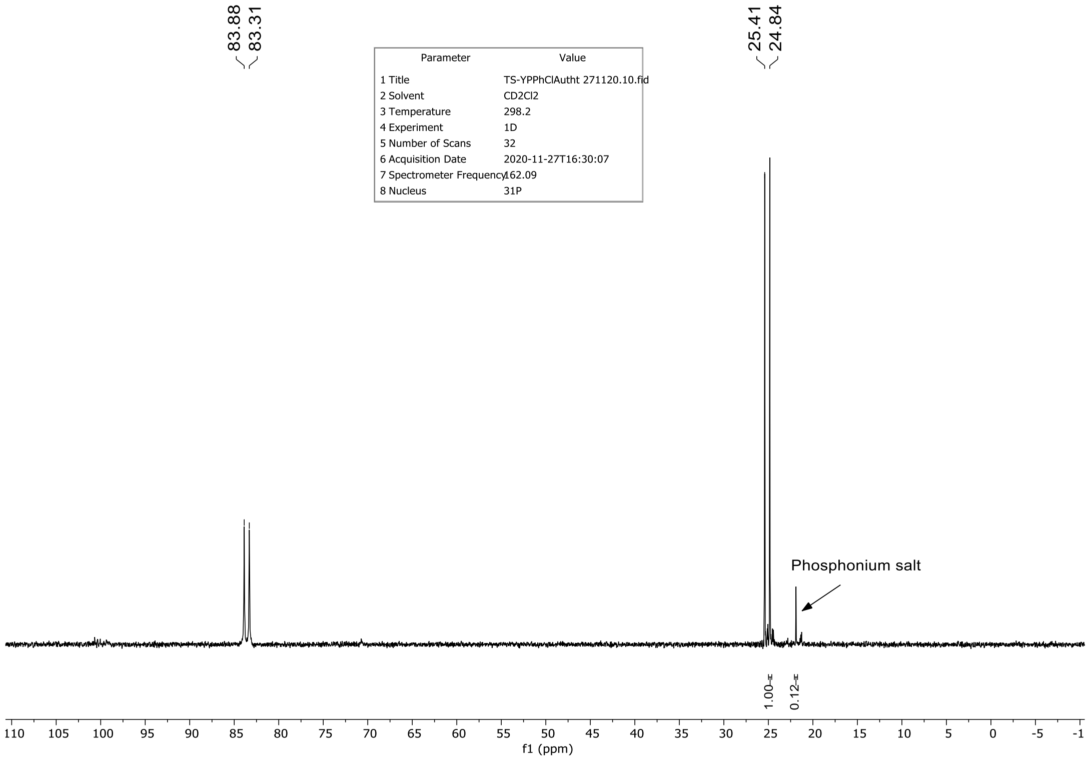

Figure S42: $^{31}P\{^1H\}$ NMR spectrum of **[YPPhClAu(tht)]AlCl$_4$**.

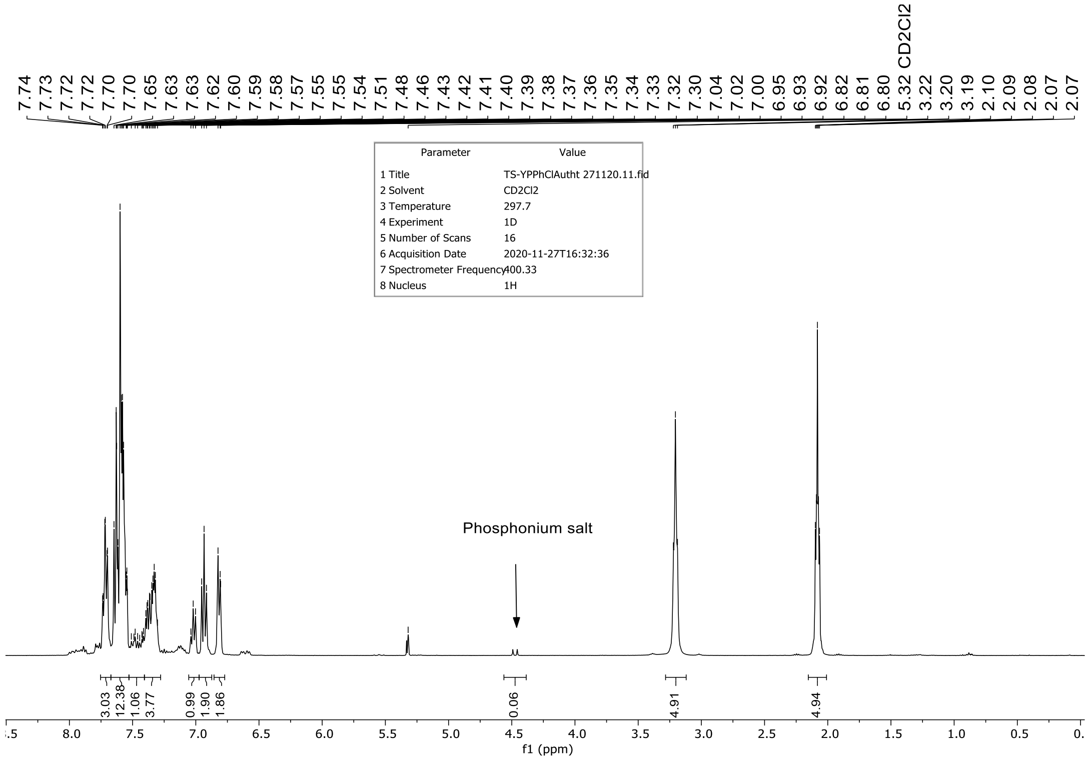

Figure S43: ^{1}H NMR spectrum of **[YPPhClAu(tht)]AlCl$_4$**.

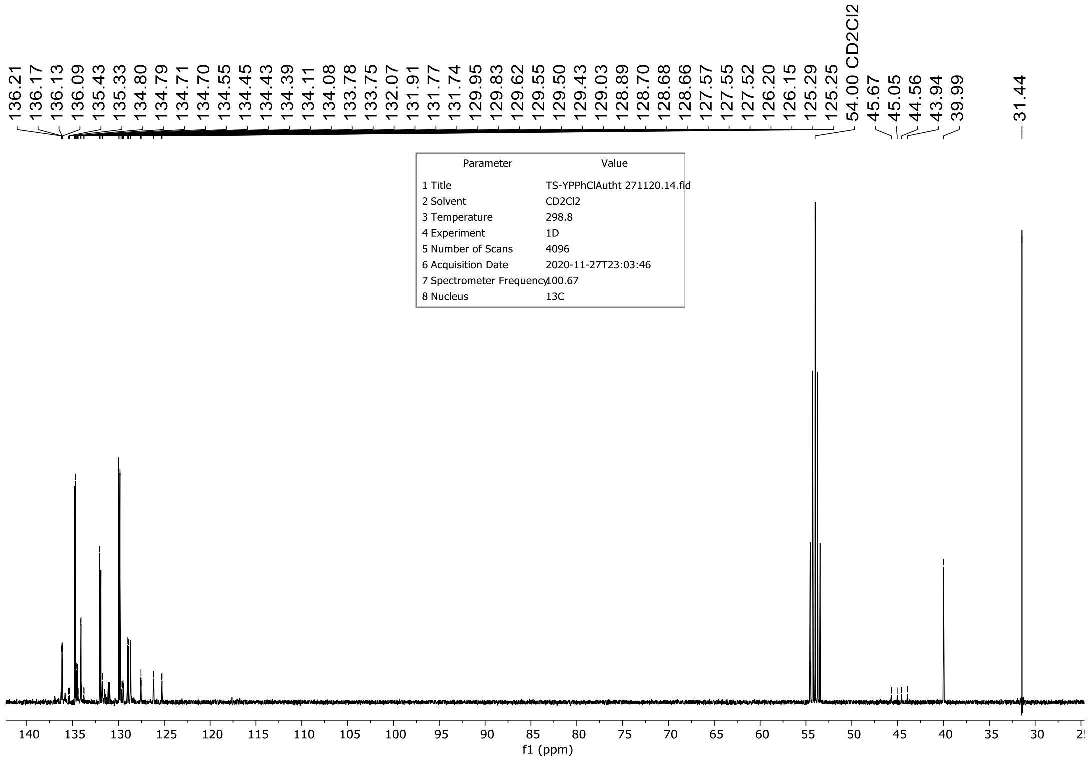

Figure S44: ^{13}C{^{1}H} NMR spectrum of **[YPPhClAu(tht)]AlCl$_4$**.

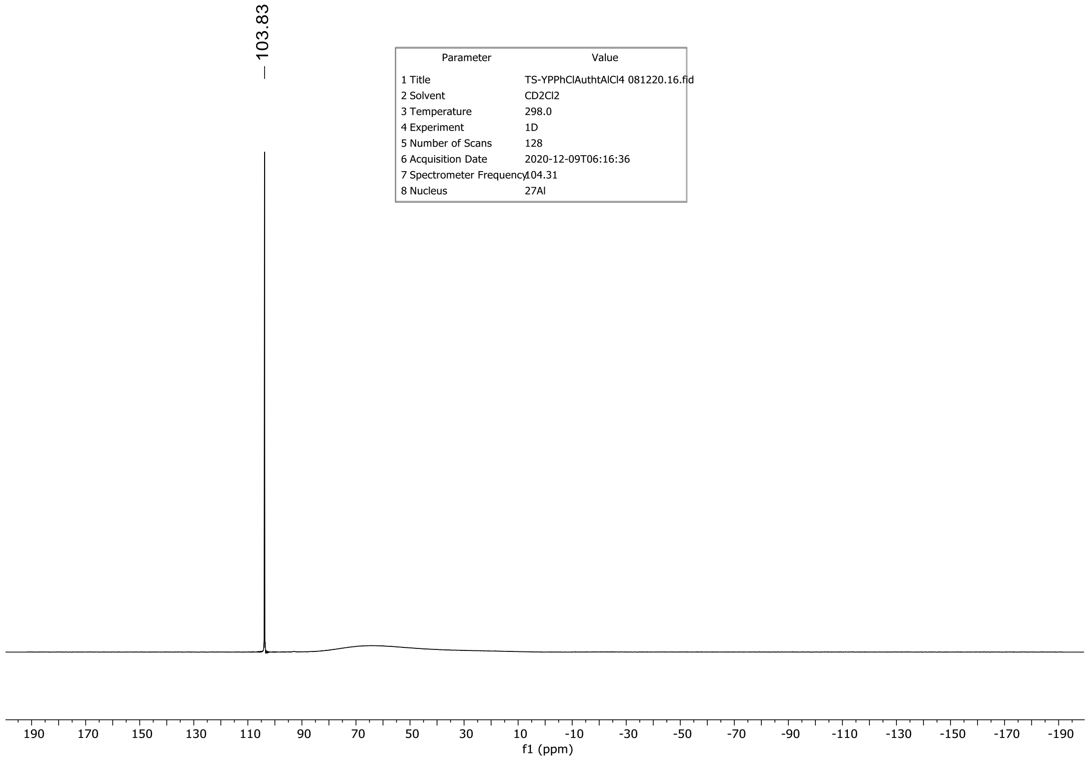

Figure S45: ^{27}Al{^{1}H} NMR spectrum of **[YPPhClAu(tht)]AlCl$_4$**.

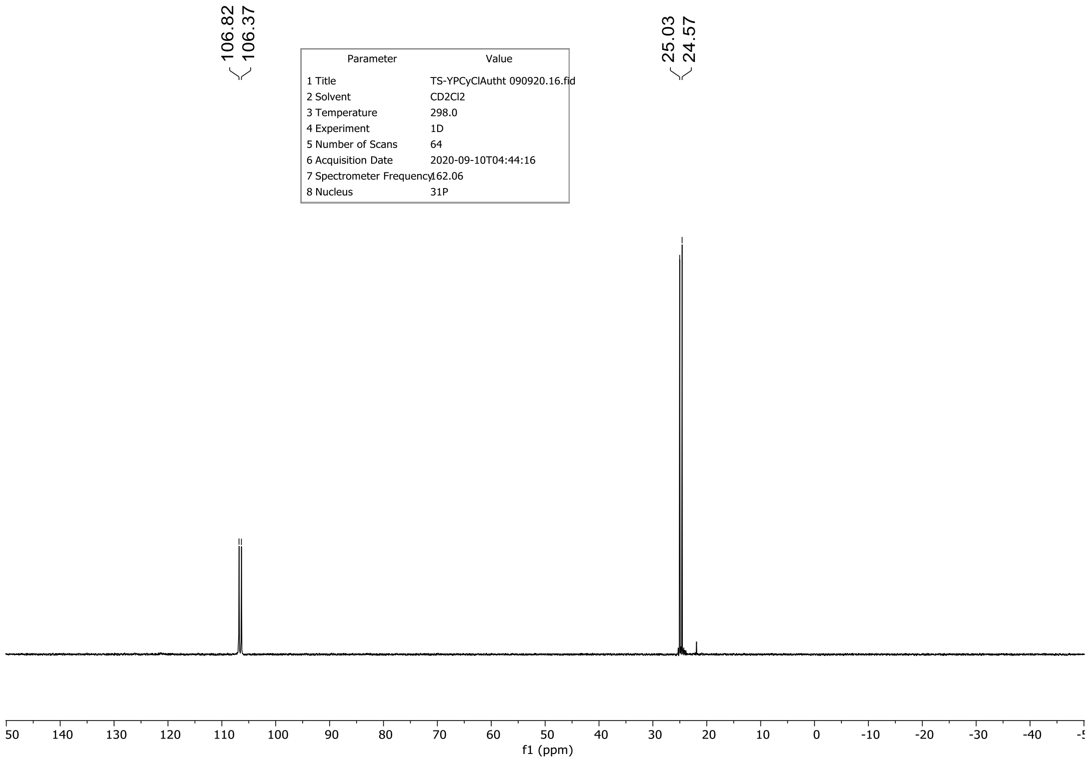

Figure S46: ^{31}P{^{1}H} NMR spectrum of **[YPCyClAu(tht)]AlCl$_4$**.

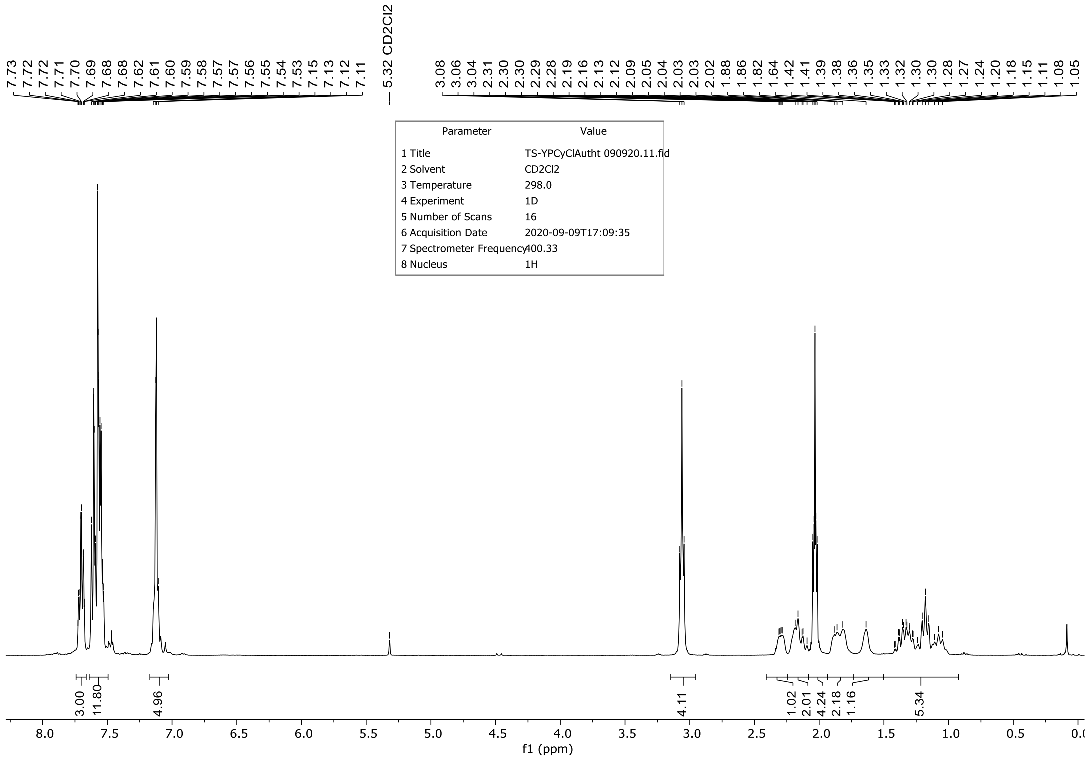

Figure S47: ^{1}H NMR spectrum of **[YPCyClAu(tht)]AlCl$_4$**.

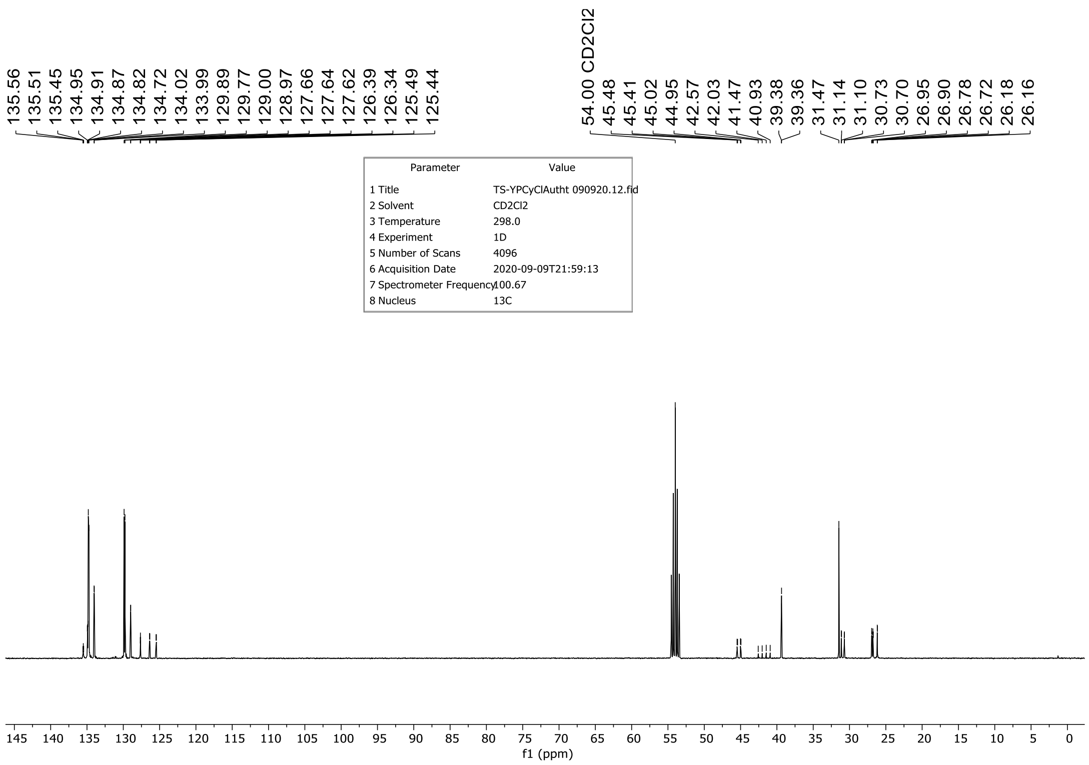

Figure S48: ^{13}C{^{1}H} NMR spectrum of **[YPCyClAu(tht)]AlCl$_4$**.

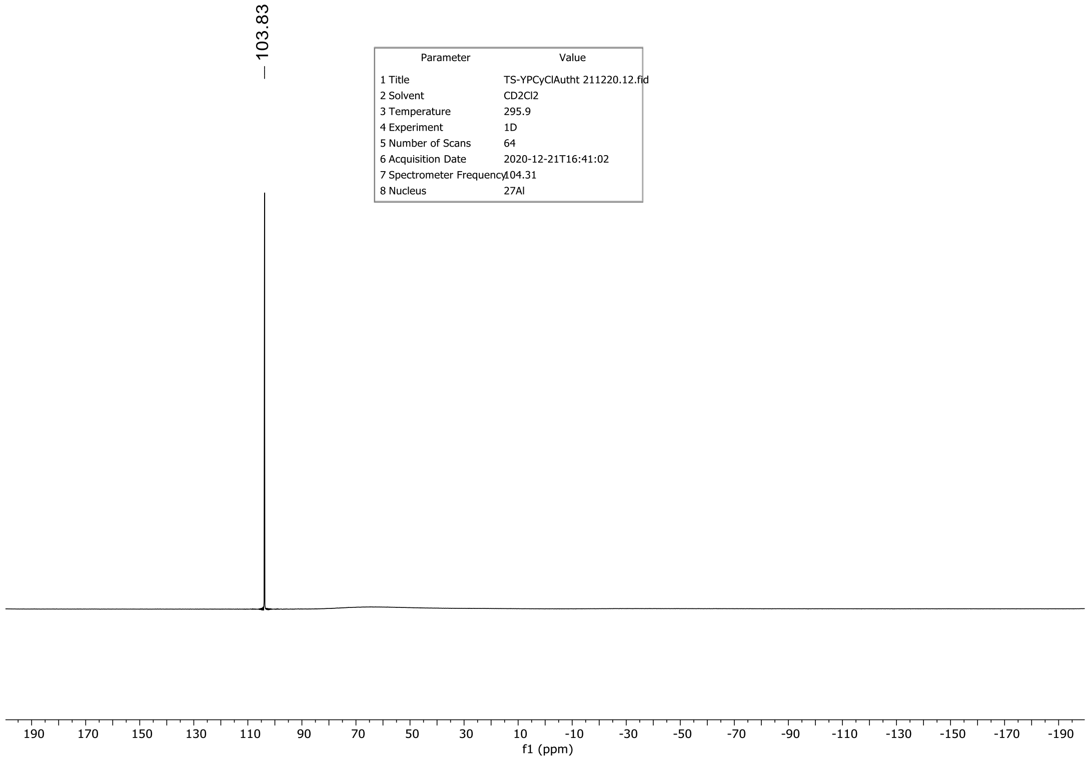

Figure S49: ^{27}Al{^{1}H} NMR spectrum of **[YPCyClAu(tht)]AlCl$_4$**.

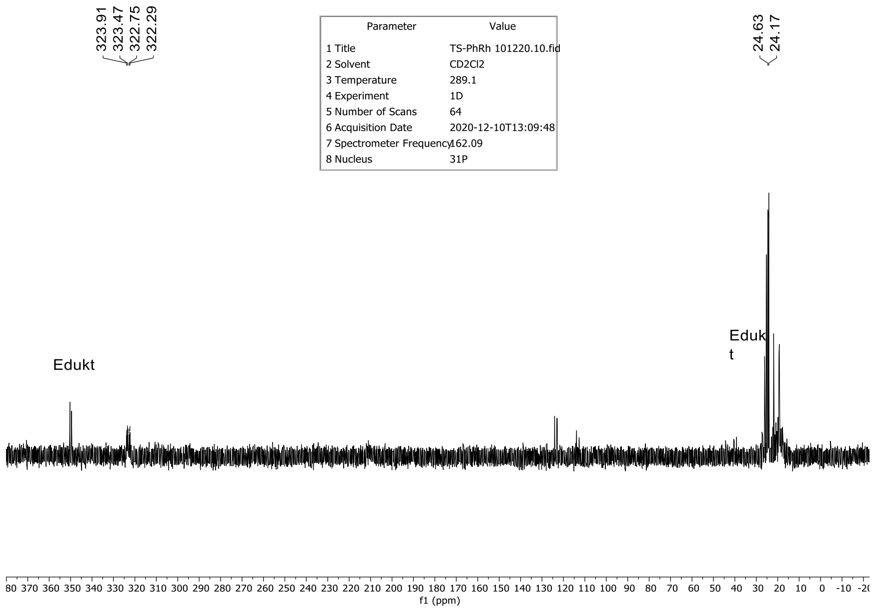

Figure S50: ^{31}P{^{1}H} NMR spectrum of **[YPPhRhCl(cod)]AlCl$_4$**.

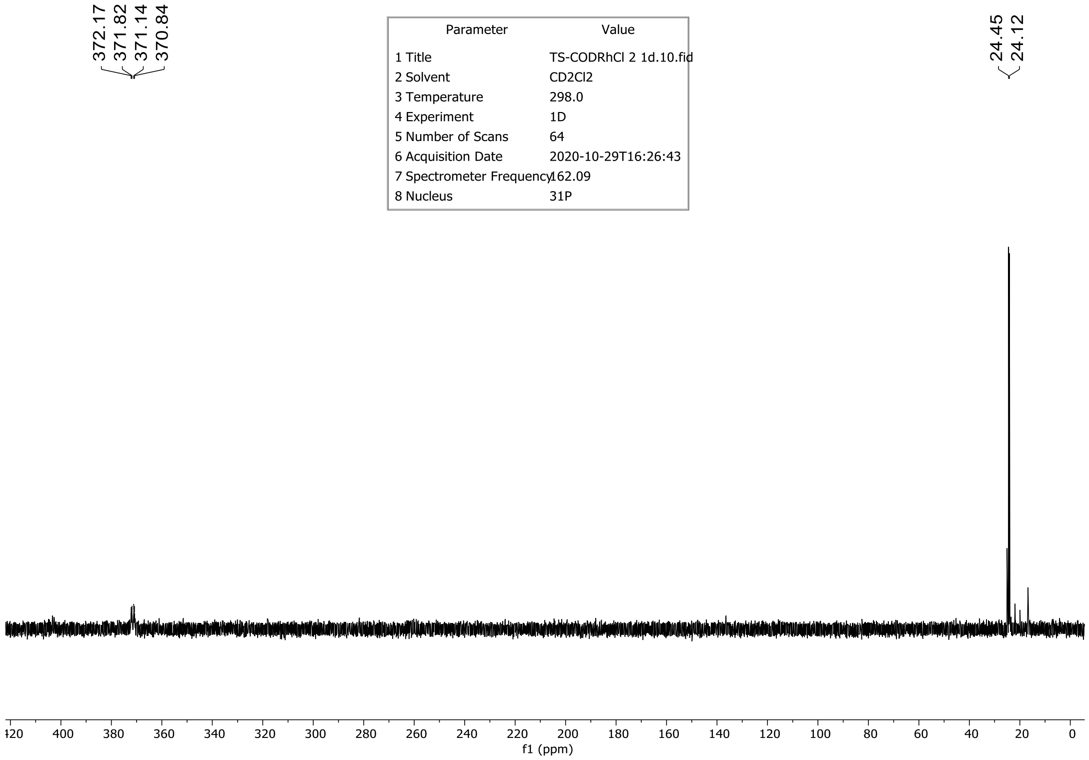

Figure S51: $^{31}P\{^{1}H\}$ NMR spectrum of **[YPCyRhCl(cod)]AlCl$_4$**.

3 Crystal structure analysis

3.1 General information

Data collection of all compounds was conducted with a Rigaku Synergy. The structures were solved using direct methods, refined with the Shelx software package[8,9] and expanded using Fourier techniques. The crystals of all compounds were mounted in an inert oil (perfluoropolyalkylether). Crystal structure determinations were affected at 100 K.

3.2 Crystal structure of $HYSiMe_3I$

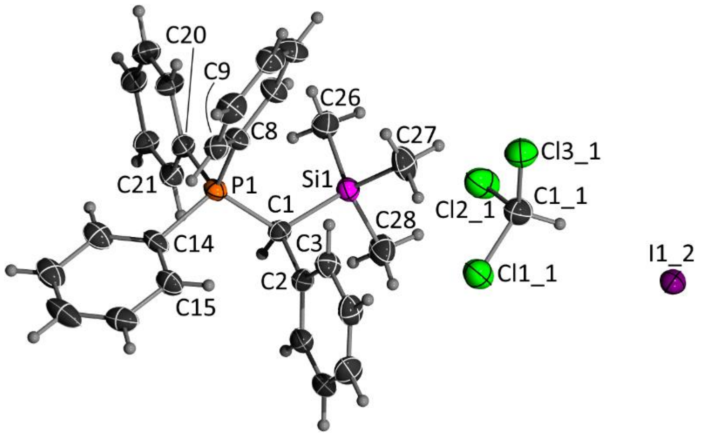

Figure S52**:** ORTEP Plot of compound **$HYSiMe_3I$**. Ellipsoids are drawn at the 50% probability level.

Table C1. Crystal data and structure refinement for **$HYSiMe_3I$**.

CCDC number	2121597	
Empirical formula	C29 H31 Cl3 I P Si	
Formula weight	671.85	
Temperature	293(2) K	
Wavelength	1.54184 Å	
Crystal system	Triclinic	
Space group	P-1	
Unit cell dimensions	a = 9.5511(2) Å	a= 103.805(2)°.
	b = 9.6548(3) Å	b= 103.790(2)°.
	c = 17.7296(3) Å	g = 95.521(2)°.
Volume	1521.48(7) $Å^3$	
Z	2	
Density (calculated)	1.467 Mg/m^3	
Absorption coefficient	11.686 mm^{-1}	
F(000)	676	
Crystal size	0.342 x 0.083 x 0.046 mm^3	
Theta range for data collection	2.666 to 76.839°.	
Index ranges	-12<=h<=11, -12<=k<=11, -22<=l<=22	
Reflections collected	6241	
Independent reflections	6241 [R(int) = 0.]	
Completeness to theta = 67.684°	99.9 %	
Absorption correction	Gaussian	
Max. and min. transmission	1.000 and 0.167	
Refinement method	Full-matrix least-squares on F^2	
Data / restraints / parameters	6241 / 0 / 320	
Goodness-of-fit on F^2	1.137	

Final R indices [I>2sigma(I)]	R1 = 0.0817, wR2 = 0.2393
R indices (all data)	R1 = 0.0833, wR2 = 0.2405
Extinction coefficient	n/a
Largest diff. peak and hole	1.860 and -1.416 e.$Å^{-3}$

Table C2. Atomic coordinates (x 10^4) and equivalent isotropic displacement parameters ($Å^2$x 10^3) for **HYSiMe$_3$I**. U(eq) is defined as one third of the trace of the orthogonalized U^{ij} tensor.

	x	y	z	U(eq)
P(1)	4432(2)	4946(3)	1697(2)	26(1)
Si(1)	5174(3)	3044(3)	3000(2)	30(1)
C(1)	4099(10)	4393(10)	2551(6)	27(2)
C(2)	4007(11)	5664(11)	3231(6)	29(2)
C(3)	5233(12)	6740(11)	3653(7)	34(2)
C(4)	5141(13)	7836(12)	4283(7)	39(2)
C(5)	3845(14)	7915(14)	4507(7)	45(3)
C(6)	2645(12)	6836(14)	4097(7)	41(3)
C(7)	2729(12)	5735(12)	3470(7)	34(2)
C(8)	6290(10)	5823(11)	1908(6)	29(2)
C(9)	6604(11)	7296(11)	1925(6)	29(2)
C(10)	8005(14)	7972(13)	2066(8)	43(3)
C(11)	9148(12)	7190(13)	2218(7)	39(2)
C(12)	8857(12)	5714(14)	2201(8)	43(3)
C(13)	7422(11)	5068(12)	2040(7)	35(2)
C(14)	3167(10)	6132(11)	1374(7)	32(2)
C(15)	3160(12)	7463(11)	1917(7)	36(2)
C(16)	2214(13)	8352(13)	1626(9)	43(3)
C(17)	1389(13)	7970(14)	838(10)	52(4)
C(18)	1407(12)	6721(14)	313(8)	43(3)
C(19)	2333(11)	5754(12)	583(8)	37(2)
C(20)	4118(11)	3348(11)	876(6)	28(2)
C(21)	2826(11)	2371(11)	689(6)	31(2)
C(22)	2542(11)	1141(11)	52(6)	31(2)
C(23)	3576(12)	863(12)	-385(7)	37(2)
C(24)	4842(13)	1816(13)	-190(7)	40(2)
C(25)	5123(12)	3060(11)	418(6)	33(2)
C(26)	5451(13)	1494(11)	2218(7)	37(2)
C(27)	6950(14)	3945(14)	3729(8)	46(3)
C(28)	3932(14)	2328(14)	3536(8)	44(3)
Cl11	8766(3)	10057(3)	6054(2)	50(1)
Cl21	9267(4)	7205(4)	5280(2)	58(1)
Cl31	11698(3)	9590(4)	6023(2)	51(1)
C11	9976(13)	8794(14)	6088(8)	44(3)
I12	10113(1)	8100(1)	8133(1)	32(1)

Table C3. Bond lengths [Å] and angles [°] for **HYSiMe$_3$I**.

P(1)-C(20)	1.795(10)
P(1)-C(8)	1.802(10)
P(1)-C(1)	1.806(10)
P(1)-C(14)	1.831(10)
Si(1)-C(27)	1.854(12)
Si(1)-C(28)	1.866(12)
Si(1)-C(26)	1.869(11)
Si(1)-C(1)	1.935(10)
C(1)-C(2)	1.527(14)
C(2)-C(7)	1.387(14)
C(2)-C(3)	1.410(14)
C(3)-C(4)	1.370(15)
C(4)-C(5)	1.391(18)
C(5)-C(6)	1.392(18)
C(6)-C(7)	1.366(16)
C(8)-C(13)	1.367(14)
C(8)-C(9)	1.417(13)
C(9)-C(10)	1.368(15)

C(10)-C(11)	1.397(18)
C(11)-C(12)	1.417(17)
C(12)-C(13)	1.383(15)
C(14)-C(19)	1.378(16)
C(14)-C(15)	1.412(15)
C(15)-C(16)	1.399(16)
C(16)-C(17)	1.37(2)
C(17)-C(18)	1.34(2)
C(18)-C(19)	1.434(16)
C(20)-C(21)	1.397(14)
C(20)-C(25)	1.405(14)
C(21)-C(22)	1.382(14)
C(22)-C(23)	1.401(15)
C(23)-C(24)	1.365(16)
C(24)-C(25)	1.364(15)
Cl11-C11	1.760(13)
Cl21-C11	1.776(13)
Cl31-C11	1.787(13)
C(20)-P(1)-C(8)	108.4(5)
C(20)-P(1)-C(1)	107.6(5)
C(8)-P(1)-C(1)	111.7(5)
C(20)-P(1)-C(14)	107.5(5)
C(8)-P(1)-C(14)	109.9(5)
C(1)-P(1)-C(14)	111.6(5)
C(27)-Si(1)-C(28)	109.9(6)
C(27)-Si(1)-C(26)	110.2(6)
C(28)-Si(1)-C(26)	108.2(6)
C(27)-Si(1)-C(1)	112.3(5)
C(28)-Si(1)-C(1)	102.4(5)
C(26)-Si(1)-C(1)	113.5(5)
C(2)-C(1)-P(1)	112.7(7)
C(2)-C(1)-Si(1)	109.8(7)
P(1)-C(1)-Si(1)	121.9(5)
C(7)-C(2)-C(3)	118.9(10)
C(7)-C(2)-C(1)	120.1(9)
C(3)-C(2)-C(1)	120.9(9)
C(4)-C(3)-C(2)	119.8(10)
C(3)-C(4)-C(5)	120.9(11)
C(4)-C(5)-C(6)	119.1(10)
C(7)-C(6)-C(5)	120.5(11)
C(6)-C(7)-C(2)	120.9(10)
C(13)-C(8)-C(9)	118.7(9)
C(13)-C(8)-P(1)	120.4(8)
C(9)-C(8)-P(1)	120.8(7)
C(10)-C(9)-C(8)	121.7(10)
C(9)-C(10)-C(11)	118.6(10)
C(10)-C(11)-C(12)	120.5(10)
C(13)-C(12)-C(11)	118.8(11)
C(8)-C(13)-C(12)	121.6(11)
C(19)-C(14)-C(15)	121.7(10)
C(19)-C(14)-P(1)	118.7(9)
C(15)-C(14)-P(1)	119.4(8)
C(16)-C(15)-C(14)	116.9(11)
C(17)-C(16)-C(15)	121.1(12)
C(18)-C(17)-C(16)	122.5(11)
C(17)-C(18)-C(19)	118.8(12)
C(14)-C(19)-C(18)	119.0(11)
C(21)-C(20)-C(25)	119.5(9)
C(21)-C(20)-P(1)	118.5(8)
C(25)-C(20)-P(1)	122.0(8)
C(22)-C(21)-C(20)	119.9(10)
C(21)-C(22)-C(23)	119.5(10)
C(24)-C(23)-C(22)	120.1(10)
C(25)-C(24)-C(23)	121.3(10)
C(24)-C(25)-C(20)	119.6(10)
Cl11-C11-Cl21	110.6(7)
Cl11-C11-Cl31	109.1(7)
Cl21-C11-Cl31	109.8(7)

Table C4. Anisotropic displacement parameters ($Å^2 x 10^3$) for **HYSiMe3I**. The anisotropic displacement factor exponent takes the form: $-2p^2[h^2 a^{*2}U^{11} + ... + 2 h k a^* b^* U^{12}]$

	U^{11}	U^{22}	U^{33}	U^{23}	U^{13}	U^{12}
P(1)	20(1)	28(1)	30(1)	9(1)	7(1)	4(1)
Si(1)	28(1)	31(1)	28(1)	8(1)	4(1)	6(1)
C(1)	25(4)	30(5)	30(5)	12(4)	11(4)	5(4)
C(2)	26(5)	35(5)	24(5)	5(4)	6(4)	6(4)
C(3)	30(5)	35(5)	33(5)	5(4)	5(4)	4(4)
C(4)	46(6)	34(5)	30(5)	2(4)	0(5)	7(5)
C(5)	53(7)	45(6)	31(6)	-3(5)	8(5)	16(6)
C(6)	34(5)	58(7)	35(6)	17(5)	10(5)	22(5)
C(7)	29(5)	42(6)	33(5)	14(5)	6(4)	9(4)
C(8)	23(4)	31(5)	35(5)	10(4)	9(4)	6(4)
C(9)	32(5)	27(5)	32(5)	11(4)	11(4)	5(4)
C(10)	46(6)	37(6)	48(7)	13(5)	15(5)	-5(5)
C(11)	27(5)	47(6)	39(6)	8(5)	8(4)	-6(4)
C(12)	23(5)	48(7)	55(7)	7(6)	11(5)	4(5)
C(13)	27(5)	35(5)	40(6)	9(4)	6(4)	5(4)
C(14)	19(4)	35(5)	48(6)	22(5)	10(4)	9(4)
C(15)	36(5)	31(5)	44(6)	15(5)	10(5)	14(4)
C(16)	39(6)	39(6)	65(8)	23(6)	27(6)	14(5)
C(17)	27(5)	48(7)	98(11)	48(8)	21(6)	13(5)
C(18)	25(5)	50(7)	58(8)	25(6)	6(5)	1(5)
C(19)	26(5)	36(5)	51(7)	16(5)	8(5)	0(4)
C(20)	28(5)	31(5)	29(5)	10(4)	9(4)	5(4)
C(21)	25(5)	35(5)	30(5)	7(4)	5(4)	1(4)
C(22)	30(5)	33(5)	31(5)	7(4)	11(4)	3(4)
C(23)	40(6)	36(5)	32(5)	4(4)	9(5)	9(5)
C(24)	37(6)	48(6)	35(6)	6(5)	15(5)	11(5)
C(25)	32(5)	34(5)	34(5)	5(4)	14(4)	1(4)
C(26)	45(6)	31(5)	34(6)	6(4)	10(5)	8(5)
C(27)	43(6)	43(6)	39(6)	6(5)	-7(5)	5(5)
C(28)	43(6)	51(7)	45(7)	25(6)	11(5)	7(5)
Cl11	46(2)	52(2)	53(2)	15(1)	11(1)	13(1)
Cl21	49(2)	55(2)	57(2)	-2(2)	7(1)	11(1)
Cl31	44(2)	59(2)	54(2)	18(1)	18(1)	7(1)
C11	38(6)	49(7)	43(7)	11(5)	12(5)	9(5)
I12	29(1)	34(1)	33(1)	8(1)	7(1)	1(1)

3.3 Crystal structure of 1.

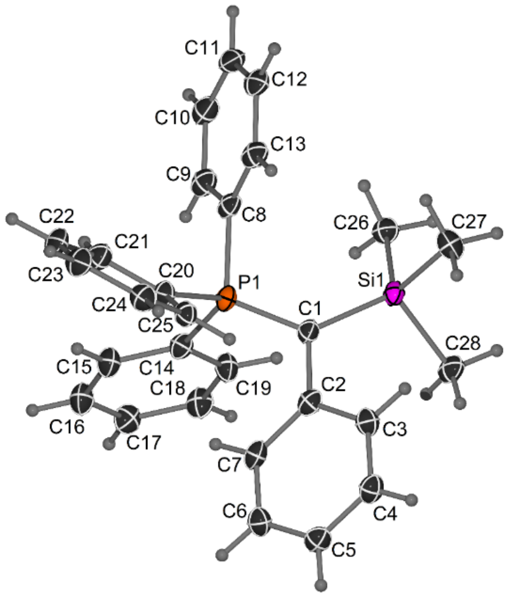

Figure S53**:** ORTEP Plot of **protonated precursor of 1**. Ellipsoids are drawn at the 50% probability level.

Table C5. Crystal data and structure refinement for **1**.

CCDC number	2121588	
Empirical formula	C28 H29 P Si	
Formula weight	424.57	
Temperature	100(2) K	
Wavelength	1.54184 Å	
Crystal system	Monoclinic	
Space group	$P2_1/c$	
Unit cell dimensions	a = 9.1300(3) Å	a= 90°.
	b = 27.7779(9) Å	b= 100.627(3)°.
	c = 9.1873(3) Å	g = 90°.
Volume	2290.06(13) $Å^3$	
Z	4	
Density (calculated)	1.231 Mg/m^3	
Absorption coefficient	1.639 mm^{-1}	
F(000)	904	
Crystal size	0.158 x 0.117 x 0.073 mm^3	
Theta range for data collection	4.928 to 74.999°.	
Index ranges	-11<=h<=11, -34<=k<=31, -11<=l<=9	
Reflections collected	10401	
Independent reflections	4543 [R(int) = 0.0287]	
Completeness to theta = 67.684°	98.2 %	
Absorption correction	Gaussian	
Max. and min. transmission	1.000 and 0.831	
Refinement method	Full-matrix least-squares on F^2	
Data / restraints / parameters	4543 / 0 / 274	
Goodness-of-fit on F^2	1.033	
Final R indices [I>2sigma(I)]	R1 = 0.0450, wR2 = 0.1128	
R indices (all data)	R1 = 0.0529, wR2 = 0.1198	
Extinction coefficient	n/a	
Largest diff. peak and hole	1.152 and -0.499 e.$Å^{-3}$	

Table C6. Atomic coordinates (x 10^4) and equivalent isotropic displacement parameters ($Å^2$x 10^3) for **1**. U(eq) is defined as one third of the trace of the orthogonalized U^{ij} tensor.

	x	y	z	U(eq)
P(1)	3207(1)	6042(1)	3102(1)	19(1)
Si(1)	183(1)	6568(1)	1839(1)	19(1)
C(1)	2223(2)	6456(1)	1960(2)	20(1)
C(2)	2965(2)	6659(1)	793(2)	21(1)
C(3)	2575(2)	7118(1)	194(2)	23(1)
C(4)	3222(2)	7314(1)	-925(2)	25(1)
C(5)	4305(2)	7064(1)	-1500(2)	24(1)
C(6)	4738(2)	6614(1)	-919(2)	24(1)
C(7)	4080(2)	6414(1)	195(2)	23(1)
C(8)	2339(2)	5896(1)	4674(2)	22(1)
C(9)	1933(2)	5430(1)	4980(2)	26(1)
C(10)	1325(2)	5342(1)	6233(2)	29(1)
C(11)	1135(2)	5715(1)	7181(2)	28(1)
C(12)	1555(2)	6182(1)	6898(2)	27(1)
C(13)	2163(2)	6270(1)	5651(2)	25(1)
C(14)	3435(2)	5459(1)	2214(2)	22(1)
C(15)	4711(2)	5171(1)	2449(2)	25(1)
C(16)	4722(2)	4736(1)	1695(3)	29(1)
C(17)	3464(2)	4585(1)	711(2)	26(1)
C(18)	2193(2)	4869(1)	473(2)	28(1)
C(19)	2187(2)	5307(1)	1205(2)	24(1)
C(20)	5041(2)	6243(1)	4027(2)	20(1)
C(21)	5525(2)	6709(1)	3804(2)	21(1)
C(22)	6796(2)	6893(1)	4714(2)	25(1)
C(23)	7591(2)	6611(1)	5826(2)	29(1)
C(24)	7132(2)	6146(1)	6044(2)	29(1)
C(25)	5850(2)	5963(1)	5167(2)	26(1)
C(26)	-860(2)	6095(1)	2698(2)	24(1)
C(27)	-300(2)	7159(1)	2636(3)	27(1)
C(28)	-733(2)	6578(1)	-171(2)	26(1)

Table C7. Bond lengths [Å] and angles [°] for **1**.

P(1)-C(1)	1.6992(19)
P(1)-C(8)	1.816(2)
P(1)-C(20)	1.8215(19)
P(1)-C(14)	1.840(2)
Si(1)-C(1)	1.871(2)
Si(1)-C(26)	1.879(2)
Si(1)-C(28)	1.881(2)
Si(1)-C(27)	1.884(2)
C(1)-C(2)	1.481(3)
C(2)-C(3)	1.409(3)
C(2)-C(7)	1.416(3)
C(3)-C(4)	1.388(3)
C(3)-H(3)	0.9500
C(4)-C(5)	1.390(3)
C(4)-H(4)	0.9500
C(5)-C(6)	1.388(3)
C(5)-H(5)	0.9500
C(6)-C(7)	1.393(3)
C(6)-H(6)	0.9500
C(7)-H(7)	0.9500
C(8)-C(9)	1.390(3)
C(8)-C(13)	1.402(3)
C(9)-C(10)	1.389(3)
C(9)-H(9)	0.9500
C(10)-C(11)	1.386(3)
C(10)-H(10)	0.9500
C(11)-C(12)	1.391(3)
C(11)-H(11)	0.9500
C(12)-C(13)	1.384(3)
C(12)-H(12)	0.9500
C(13)-H(13)	0.9500

C(14)-C(19)	1.395(3)
C(14)-C(15)	1.397(3)
C(15)-C(16)	1.394(3)
C(15)-H(15)	0.9500
C(16)-C(17)	1.390(3)
C(16)-H(16)	0.9500
C(17)-C(18)	1.388(3)
C(17)-H(17)	0.9500
C(18)-C(19)	1.392(3)
C(18)-H(18)	0.9500
C(19)-H(19)	0.9500
C(20)-C(21)	1.394(3)
C(20)-C(25)	1.401(3)
C(21)-C(22)	1.396(3)
C(21)-H(21)	0.9500
C(22)-C(23)	1.383(3)
C(22)-H(22)	0.9500
C(23)-C(24)	1.383(3)
C(23)-H(23)	0.9500
C(24)-C(25)	1.389(3)
C(24)-H(24)	0.9500
C(25)-H(25)	0.9500
C(26)-H(26A)	0.9800
C(26)-H(26B)	0.9800
C(26)-H(26C)	0.9800
C(27)-H(27A)	0.9800
C(27)-H(27B)	0.9800
C(27)-H(27C)	0.9800
C(28)-H(28A)	0.9800
C(28)-H(28B)	0.9800
C(28)-H(28C)	0.9800
C(1)-P(1)-C(8)	112.56(9)
C(1)-P(1)-C(20)	114.53(9)
C(8)-P(1)-C(20)	101.29(9)
C(1)-P(1)-C(14)	114.39(9)
C(8)-P(1)-C(14)	104.95(9)
C(20)-P(1)-C(14)	107.91(9)
C(1)-Si(1)-C(26)	115.84(9)
C(1)-Si(1)-C(28)	108.58(9)
C(26)-Si(1)-C(28)	104.83(9)
C(1)-Si(1)-C(27)	114.98(9)
C(26)-Si(1)-C(27)	105.81(10)
C(28)-Si(1)-C(27)	105.91(10)
C(2)-C(1)-P(1)	116.38(14)
C(2)-C(1)-Si(1)	118.51(13)
P(1)-C(1)-Si(1)	123.77(11)
C(3)-C(2)-C(7)	115.51(18)
C(3)-C(2)-C(1)	120.70(18)
C(7)-C(2)-C(1)	123.79(18)
C(4)-C(3)-C(2)	122.21(19)
C(4)-C(3)-H(3)	118.9
C(2)-C(3)-H(3)	118.9
C(3)-C(4)-C(5)	121.05(19)
C(3)-C(4)-H(4)	119.5
C(5)-C(4)-H(4)	119.5
C(6)-C(5)-C(4)	118.38(19)
C(6)-C(5)-H(5)	120.8
C(4)-C(5)-H(5)	120.8
C(5)-C(6)-C(7)	120.67(19)
C(5)-C(6)-H(6)	119.7
C(7)-C(6)-H(6)	119.7
C(6)-C(7)-C(2)	122.15(19)
C(6)-C(7)-H(7)	118.9
C(2)-C(7)-H(7)	118.9
C(9)-C(8)-C(13)	119.62(19)
C(9)-C(8)-P(1)	122.73(16)
C(13)-C(8)-P(1)	117.55(15)
C(10)-C(9)-C(8)	119.6(2)
C(10)-C(9)-H(9)	120.2
C(8)-C(9)-H(9)	120.2
C(11)-C(10)-C(9)	120.3(2)
C(11)-C(10)-H(10)	119.9

C(9)-C(10)-H(10)	119.9
C(10)-C(11)-C(12)	120.7(2)
C(10)-C(11)-H(11)	119.7
C(12)-C(11)-H(11)	119.7
C(13)-C(12)-C(11)	119.0(2)
C(13)-C(12)-H(12)	120.5
C(11)-C(12)-H(12)	120.5
C(12)-C(13)-C(8)	120.7(2)
C(12)-C(13)-H(13)	119.6
C(8)-C(13)-H(13)	119.6
C(19)-C(14)-C(15)	119.02(19)
C(19)-C(14)-P(1)	114.51(15)
C(15)-C(14)-P(1)	126.47(15)
C(16)-C(15)-C(14)	120.18(19)
C(16)-C(15)-H(15)	119.9
C(14)-C(15)-H(15)	119.9
C(17)-C(16)-C(15)	120.3(2)
C(17)-C(16)-H(16)	119.9
C(15)-C(16)-H(16)	119.9
C(18)-C(17)-C(16)	119.8(2)
C(18)-C(17)-H(17)	120.1
C(16)-C(17)-H(17)	120.1
C(17)-C(18)-C(19)	119.97(19)
C(17)-C(18)-H(18)	120.0
C(19)-C(18)-H(18)	120.0
C(18)-C(19)-C(14)	120.69(19)
C(18)-C(19)-H(19)	119.7
C(14)-C(19)-H(19)	119.7
C(21)-C(20)-C(25)	119.22(18)
C(21)-C(20)-P(1)	120.39(15)
C(25)-C(20)-P(1)	119.35(15)
C(20)-C(21)-C(22)	120.14(19)
C(20)-C(21)-H(21)	119.9
C(22)-C(21)-H(21)	119.9
C(23)-C(22)-C(21)	120.1(2)
C(23)-C(22)-H(22)	120.0
C(21)-C(22)-H(22)	120.0
C(22)-C(23)-C(24)	120.23(19)
C(22)-C(23)-H(23)	119.9
C(24)-C(23)-H(23)	119.9
C(23)-C(24)-C(25)	120.2(2)
C(23)-C(24)-H(24)	119.9
C(25)-C(24)-H(24)	119.9
C(24)-C(25)-C(20)	120.1(2)
C(24)-C(25)-H(25)	120.0
C(20)-C(25)-H(25)	120.0
Si(1)-C(26)-H(26A)	109.5
Si(1)-C(26)-H(26B)	109.5
H(26A)-C(26)-H(26B)	109.5
Si(1)-C(26)-H(26C)	109.5
H(26A)-C(26)-H(26C)	109.5
H(26B)-C(26)-H(26C)	109.5
Si(1)-C(27)-H(27A)	109.5
Si(1)-C(27)-H(27B)	109.5
H(27A)-C(27)-H(27B)	109.5
Si(1)-C(27)-H(27C)	109.5
H(27A)-C(27)-H(27C)	109.5
H(27B)-C(27)-H(27C)	109.5
Si(1)-C(28)-H(28A)	109.5
Si(1)-C(28)-H(28B)	109.5
H(28A)-C(28)-H(28B)	109.5
Si(1)-C(28)-H(28C)	109.5
H(28A)-C(28)-H(28C)	109.5
H(28B)-C(28)-H(28C)	109.5

Table C8. Anisotropic displacement parameters (Å^{2}x 10^3) for **1**. The anisotropic displacement factor exponent takes the form: $-2p^2[h^2 a^{*2}U^{11} + ... + 2 h k a^* b^* U^{12}]$

	U^{11}	U^{22}	U^{33}	U^{23}	U^{13}	U^{12}
P(1)	16(1)	21(1)	18(1)	0(1)	-1(1)	0(1)

Si(1)	17(1)	21(1)	18(1)	0(1)	-1(1)	1(1)
C(1)	19(1)	22(1)	18(1)	1(1)	0(1)	2(1)
C(2)	19(1)	23(1)	19(1)	-2(1)	-2(1)	-4(1)
C(3)	20(1)	22(1)	26(1)	-1(1)	3(1)	0(1)
C(4)	24(1)	22(1)	27(1)	4(1)	3(1)	-1(1)
C(5)	22(1)	25(1)	24(1)	3(1)	4(1)	-3(1)
C(6)	19(1)	25(1)	28(1)	0(1)	3(1)	0(1)
C(7)	21(1)	23(1)	23(1)	3(1)	-3(1)	0(1)
C(8)	17(1)	27(1)	19(1)	4(1)	-2(1)	-3(1)
C(9)	22(1)	29(1)	24(1)	0(1)	-1(1)	-2(1)
C(10)	25(1)	34(1)	27(1)	8(1)	0(1)	-3(1)
C(11)	23(1)	42(1)	19(1)	7(1)	3(1)	0(1)
C(12)	21(1)	37(1)	20(1)	0(1)	-1(1)	0(1)
C(13)	23(1)	29(1)	21(1)	0(1)	-1(1)	-3(1)
C(14)	21(1)	21(1)	21(1)	0(1)	1(1)	-1(1)
C(15)	22(1)	25(1)	26(1)	1(1)	-1(1)	1(1)
C(16)	27(1)	24(1)	33(1)	1(1)	1(1)	6(1)
C(17)	32(1)	19(1)	28(1)	-1(1)	4(1)	1(1)
C(18)	26(1)	27(1)	28(1)	-6(1)	-2(1)	-2(1)
C(19)	21(1)	24(1)	26(1)	-2(1)	-1(1)	3(1)
C(20)	15(1)	25(1)	19(1)	-1(1)	0(1)	-1(1)
C(21)	17(1)	23(1)	21(1)	-2(1)	1(1)	1(1)
C(22)	21(1)	27(1)	26(1)	-4(1)	3(1)	-3(1)
C(23)	20(1)	41(1)	23(1)	-5(1)	-2(1)	-3(1)
C(24)	23(1)	40(1)	21(1)	3(1)	-3(1)	3(1)
C(25)	24(1)	29(1)	24(1)	3(1)	1(1)	0(1)
C(26)	23(1)	28(1)	23(1)	0(1)	3(1)	-3(1)
C(27)	22(1)	25(1)	35(1)	-4(1)	7(1)	1(1)
C(28)	20(1)	36(1)	22(1)	2(1)	-2(1)	-3(1)

3.4 Crystal structure of 2[BF$_4$]

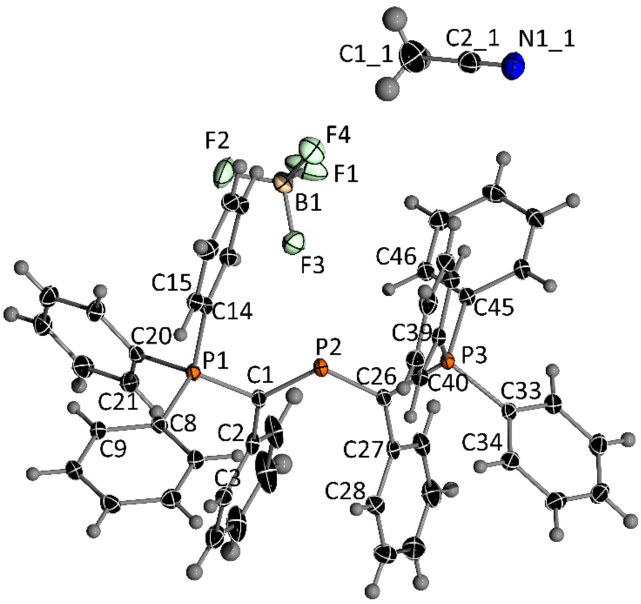

Figure S54**:** ORTEP Plot of compound **2[BF$_4$]**. Ellipsoids are drawn at the 50% probability level.

Table C9. Crystal data and structure refinement for **2[BF$_4$]**.

Identification code	2121592
Empirical formula	C52 H43 B F4 N P3
Formula weight	861.59
Temperature	100(2) K
Wavelength	1.54184 Å
Crystal system	Triclinic
Space group	P-1

Unit cell dimensions	a = 10.4362(2) Å	a= 81.7370(10)°.
	b = 13.0753(2) Å	b= 83.5180(10)°.
	c = 16.9374(2) Å	g = 72.0620(10)°.
Volume	2170.26(6) Å^3	
Z	2	
Density (calculated)	1.318 Mg/m^3	
Absorption coefficient	1.709 mm^{-1}	
F(000)	896	
Crystal size	0.222 x 0.217 x 0.051 mm^3	
Theta range for data collection	2.643 to 77.410°.	
Index ranges	-13<=h<=12, -16<=k<=16, -20<=l<=21	
Reflections collected	29253	
Independent reflections	8882 [R(int) = 0.0196]	
Completeness to theta = 67.684°	99.9 %	
Absorption correction	Gaussian	
Max. and min. transmission	1.000 and 0.447	
Refinement method	Full-matrix least-squares on F^2	
Data / restraints / parameters	8882 / 0 / 551	
Goodness-of-fit on F^2	1.026	
Final R indices [I>2sigma(I)]	R1 = 0.0353, wR2 = 0.0923	
R indices (all data)	R1 = 0.0364, wR2 = 0.0932	
Extinction coefficient	n/a	
Largest diff. peak and hole	1.000 and -0.674 e.Å^{-3}	

Table C10. Atomic coordinates (x 10^4) and equivalent isotropic displacement parameters (Å^{2}x 10^3) for shelxt_a. U(eq) is defined as one third of the trace of the orthogonalized U^{ij} tensor.

	x	y	z	U(eq)
P(1)	5558(1)	8184(1)	6645(1)	14(1)
P(2)	6735(1)	5908(1)	7186(1)	17(1)
P(3)	7826(1)	3644(1)	7743(1)	14(1)
C(1)	6061(1)	7197(1)	7457(1)	17(1)
C(2)	5852(1)	7593(1)	8259(1)	19(1)
C(3)	6435(2)	8359(1)	8425(1)	21(1)
C(4)	6147(2)	8779(1)	9157(1)	30(1)
C(5)	5303(2)	8419(1)	9741(1)	42(1)
C(6)	4743(2)	7640(1)	9591(1)	47(1)
C(7)	5000(2)	7238(1)	8856(1)	32(1)
C(8)	6730(1)	8973(1)	6361(1)	15(1)
C(9)	6283(1)	10073(1)	6084(1)	18(1)
C(10)	7212(2)	10646(1)	5852(1)	20(1)
C(11)	8582(2)	10129(1)	5900(1)	22(1)
C(12)	9036(1)	9033(1)	6181(1)	21(1)
C(13)	8114(1)	8455(1)	6412(1)	18(1)
C(14)	5444(1)	7561(1)	5782(1)	17(1)
C(15)	6252(1)	7643(1)	5078(1)	20(1)
C(16)	6144(2)	7125(1)	4437(1)	24(1)
C(17)	5244(2)	6523(1)	4501(1)	26(1)
C(18)	4428(2)	6446(1)	5201(1)	24(1)
C(19)	4514(1)	6970(1)	5842(1)	20(1)
C(20)	3910(1)	9167(1)	6807(1)	17(1)
C(21)	3357(2)	9507(1)	7550(1)	23(1)
C(22)	2115(2)	10297(1)	7606(1)	27(1)
C(23)	1424(2)	10771(1)	6929(1)	26(1)
C(24)	1971(2)	10448(1)	6191(1)	24(1)
C(25)	3202(1)	9643(1)	6129(1)	21(1)
C(26)	7320(1)	4980(1)	7986(1)	17(1)
C(27)	7472(1)	5126(1)	8822(1)	17(1)
C(28)	8338(1)	5698(1)	8959(1)	20(1)
C(29)	8530(2)	5822(1)	9732(1)	26(1)
C(30)	7875(2)	5367(1)	10384(1)	28(1)
C(31)	7009(2)	4800(1)	10253(1)	27(1)
C(32)	6803(2)	4684(1)	9480(1)	22(1)

C(33)	9024(1)	2812(1)	8450(1)	17(1)
C(34)	10277(2)	2991(1)	8445(1)	21(1)
C(35)	11175(2)	2403(1)	9008(1)	24(1)
C(36)	10829(2)	1644(1)	9584(1)	25(1)
C(37)	9583(2)	1477(1)	9598(1)	25(1)
C(38)	8676(2)	2058(1)	9034(1)	21(1)
C(39)	8632(1)	3566(1)	6739(1)	16(1)
C(40)	9669(1)	4045(1)	6513(1)	19(1)
C(41)	10329(2)	3963(1)	5757(1)	22(1)
C(42)	9950(2)	3424(1)	5214(1)	24(1)
C(43)	8910(2)	2968(1)	5429(1)	23(1)
C(44)	8254(1)	3030(1)	6190(1)	20(1)
C(45)	6479(1)	3032(1)	7791(1)	17(1)
C(46)	5140(1)	3682(1)	7814(1)	19(1)
C(47)	4106(2)	3223(1)	7806(1)	23(1)
C(48)	4402(2)	2117(1)	7782(1)	24(1)
C(49)	5736(2)	1463(1)	7762(1)	23(1)
C(50)	6776(2)	1912(1)	7760(1)	20(1)
C11	9033(2)	2235(2)	1656(2)	53(1)
N11	10980(2)	443(1)	1624(1)	40(1)
C21	10125(2)	1225(1)	1643(1)	30(1)
B12	1823(2)	5820(1)	6994(1)	23(1)
F22	1167(1)	6812(1)	6618(1)	55(1)
F32	2860(1)	5923(1)	7406(1)	34(1)
F42	2360(1)	5096(1)	6435(1)	38(1)
F12	974(1)	5415(1)	7553(1)	54(1)

Table C11. Bond lengths [Å] and angles [°] for **2[BF$_4$]**.

P(1)-C(1)	1.7523(14)
P(1)-C(14)	1.8042(14)
P(1)-C(8)	1.8143(14)
P(1)-C(20)	1.8219(14)
P(2)-C(26)	1.7141(14)
P(2)-C(1)	1.7218(14)
P(3)-C(26)	1.7567(13)
P(3)-C(45)	1.8094(14)
P(3)-C(39)	1.8134(14)
P(3)-C(33)	1.8178(14)
C(1)-C(2)	1.4917(19)
C(2)-C(7)	1.394(2)
C(2)-C(3)	1.396(2)
C(3)-C(4)	1.392(2)
C(4)-C(5)	1.378(3)
C(5)-C(6)	1.385(3)
C(6)-C(7)	1.387(3)
C(8)-C(9)	1.3960(19)
C(8)-C(13)	1.4004(19)
C(9)-C(10)	1.390(2)
C(10)-C(11)	1.386(2)
C(11)-C(12)	1.394(2)
C(12)-C(13)	1.389(2)
C(14)-C(15)	1.392(2)
C(14)-C(19)	1.4028(19)
C(15)-C(16)	1.393(2)
C(16)-C(17)	1.387(2)
C(17)-C(18)	1.390(2)
C(18)-C(19)	1.388(2)
C(20)-C(21)	1.398(2)
C(20)-C(25)	1.398(2)
C(21)-C(22)	1.390(2)
C(22)-C(23)	1.387(2)
C(23)-C(24)	1.381(2)
C(24)-C(25)	1.392(2)
C(26)-C(27)	1.4881(19)
C(27)-C(28)	1.395(2)
C(27)-C(32)	1.397(2)
C(28)-C(29)	1.385(2)

C(29)-C(30)	1.390(2)
C(30)-C(31)	1.386(2)
C(31)-C(32)	1.388(2)
C(33)-C(38)	1.394(2)
C(33)-C(34)	1.396(2)
C(34)-C(35)	1.389(2)
C(35)-C(36)	1.389(2)
C(36)-C(37)	1.381(2)
C(37)-C(38)	1.392(2)
C(39)-C(44)	1.3960(19)
C(39)-C(40)	1.402(2)
C(40)-C(41)	1.384(2)
C(41)-C(42)	1.393(2)
C(42)-C(43)	1.384(2)
C(43)-C(44)	1.391(2)
C(45)-C(46)	1.3929(19)
C(45)-C(50)	1.4075(19)
C(46)-C(47)	1.390(2)
C(47)-C(48)	1.388(2)
C(48)-C(49)	1.392(2)
C(49)-C(50)	1.383(2)
C11-C21	1.455(3)
N11-C21	1.134(2)
B12-F22	1.368(2)
B12-F12	1.384(2)
B12-F42	1.3843(19)
B12-F32	1.402(2)
C(1)-P(1)-C(14)	110.33(6)
C(1)-P(1)-C(8)	112.50(6)
C(14)-P(1)-C(8)	107.47(6)
C(1)-P(1)-C(20)	114.82(7)
C(14)-P(1)-C(20)	105.59(6)
C(8)-P(1)-C(20)	105.63(6)
C(26)-P(2)-C(1)	111.90(7)
C(26)-P(3)-C(45)	115.02(6)
C(26)-P(3)-C(39)	111.07(6)
C(45)-P(3)-C(39)	106.42(6)
C(26)-P(3)-C(33)	108.20(6)
C(45)-P(3)-C(33)	106.93(6)
C(39)-P(3)-C(33)	109.01(6)
C(2)-C(1)-P(2)	130.50(10)
C(2)-C(1)-P(1)	116.16(9)
P(2)-C(1)-P(1)	113.34(8)
C(7)-C(2)-C(3)	118.01(14)
C(7)-C(2)-C(1)	119.97(14)
C(3)-C(2)-C(1)	121.94(13)
C(4)-C(3)-C(2)	121.07(15)
C(5)-C(4)-C(3)	120.15(17)
C(4)-C(5)-C(6)	119.39(16)
C(5)-C(6)-C(7)	120.71(17)
C(6)-C(7)-C(2)	120.63(17)
C(9)-C(8)-C(13)	119.70(12)
C(9)-C(8)-P(1)	121.51(10)
C(13)-C(8)-P(1)	118.78(10)
C(10)-C(9)-C(8)	119.99(13)
C(11)-C(10)-C(9)	120.14(13)
C(10)-C(11)-C(12)	120.24(13)
C(13)-C(12)-C(11)	119.92(13)
C(12)-C(13)-C(8)	120.00(13)
C(15)-C(14)-C(19)	120.20(13)
C(15)-C(14)-P(1)	122.02(11)
C(19)-C(14)-P(1)	117.78(11)
C(14)-C(15)-C(16)	119.65(14)
C(17)-C(16)-C(15)	120.14(14)
C(16)-C(17)-C(18)	120.33(14)
C(19)-C(18)-C(17)	120.08(14)
C(18)-C(19)-C(14)	119.58(14)
C(21)-C(20)-C(25)	118.96(13)
C(21)-C(20)-P(1)	124.23(11)
C(25)-C(20)-P(1)	116.72(11)
C(22)-C(21)-C(20)	119.79(14)
C(23)-C(22)-C(21)	120.85(14)

C(24)-C(23)-C(22)	119.68(14)
C(23)-C(24)-C(25)	120.06(14)
C(24)-C(25)-C(20)	120.65(14)
C(27)-C(26)-P(2)	130.94(10)
C(27)-C(26)-P(3)	116.70(10)
P(2)-C(26)-P(3)	112.36(8)
C(28)-C(27)-C(32)	118.47(13)
C(28)-C(27)-C(26)	119.25(12)
C(32)-C(27)-C(26)	122.25(13)
C(29)-C(28)-C(27)	120.63(14)
C(28)-C(29)-C(30)	120.51(15)
C(31)-C(30)-C(29)	119.33(14)
C(30)-C(31)-C(32)	120.29(14)
C(31)-C(32)-C(27)	120.75(14)
C(38)-C(33)-C(34)	119.48(13)
C(38)-C(33)-P(3)	121.14(11)
C(34)-C(33)-P(3)	119.20(11)
C(35)-C(34)-C(33)	119.96(14)
C(36)-C(35)-C(34)	120.32(14)
C(37)-C(36)-C(35)	119.87(14)
C(36)-C(37)-C(38)	120.34(14)
C(37)-C(38)-C(33)	120.03(14)
C(44)-C(39)-C(40)	119.41(13)
C(44)-C(39)-P(3)	121.58(11)
C(40)-C(39)-P(3)	119.01(10)
C(41)-C(40)-C(39)	119.93(13)
C(40)-C(41)-C(42)	120.38(14)
C(43)-C(42)-C(41)	119.88(14)
C(42)-C(43)-C(44)	120.26(14)
C(43)-C(44)-C(39)	120.12(13)
C(46)-C(45)-C(50)	119.77(13)
C(46)-C(45)-P(3)	119.71(10)
C(50)-C(45)-P(3)	120.43(11)
C(47)-C(46)-C(45)	119.88(13)
C(48)-C(47)-C(46)	120.20(14)
C(47)-C(48)-C(49)	120.15(13)
C(50)-C(49)-C(48)	120.20(13)
C(49)-C(50)-C(45)	119.80(13)
N11-C21-C11	179.1(2)
F22-B12-F12	112.03(15)
F22-B12-F42	109.79(14)
F12-B12-F42	109.12(14)
F22-B12-F32	108.92(13)
F12-B12-F32	107.11(13)
F42-B12-F32	109.81(13)

Table C12. Anisotropic displacement parameters (Å^{2}x 10^3) for **2[BF$_4$]**. The anisotropic displacement factor exponent takes the form: -2p^2[h^2 a*^{2}U^{11} + ... + 2 h k a* b* U^{12}]

	U^{11}	U^{22}	U^{33}	U^{23}	U^{13}	U^{12}
P(1)	14(1)	10(1)	18(1)	-2(1)	-1(1)	-3(1)
P(2)	19(1)	10(1)	21(1)	-1(1)	-5(1)	-2(1)
P(3)	15(1)	10(1)	18(1)	-1(1)	-3(1)	-2(1)
C(1)	17(1)	12(1)	21(1)	0(1)	-2(1)	-4(1)
C(2)	20(1)	12(1)	20(1)	1(1)	0(1)	1(1)
C(3)	22(1)	18(1)	20(1)	-2(1)	-5(1)	-1(1)
C(4)	38(1)	21(1)	24(1)	-6(1)	-9(1)	3(1)
C(5)	68(1)	23(1)	22(1)	-4(1)	7(1)	5(1)
C(6)	66(1)	22(1)	38(1)	-1(1)	30(1)	-4(1)
C(7)	37(1)	15(1)	37(1)	-1(1)	15(1)	-4(1)
C(8)	18(1)	14(1)	15(1)	-3(1)	-1(1)	-5(1)
C(9)	18(1)	15(1)	21(1)	-2(1)	-3(1)	-3(1)
C(10)	24(1)	14(1)	24(1)	-1(1)	-4(1)	-7(1)
C(11)	21(1)	22(1)	26(1)	-2(1)	-1(1)	-11(1)
C(12)	16(1)	22(1)	25(1)	-2(1)	-2(1)	-6(1)
C(13)	18(1)	15(1)	20(1)	-2(1)	-2(1)	-3(1)
C(14)	16(1)	11(1)	22(1)	-3(1)	-4(1)	-1(1)
C(15)	17(1)	18(1)	24(1)	-4(1)	-2(1)	-2(1)
C(16)	20(1)	26(1)	24(1)	-7(1)	-3(1)	1(1)
C(17)	25(1)	20(1)	29(1)	-10(1)	-11(1)	2(1)

C(18)	23(1)	15(1)	33(1)	-3(1)	-11(1)	-4(1)
C(19)	20(1)	16(1)	24(1)	-1(1)	-5(1)	-4(1)
C(20)	14(1)	12(1)	25(1)	-2(1)	-1(1)	-4(1)
C(21)	21(1)	20(1)	25(1)	-6(1)	-3(1)	-2(1)
C(22)	24(1)	24(1)	31(1)	-12(1)	0(1)	-1(1)
C(23)	15(1)	19(1)	40(1)	-6(1)	-2(1)	0(1)
C(24)	17(1)	22(1)	31(1)	1(1)	-6(1)	-3(1)
C(25)	18(1)	21(1)	23(1)	-2(1)	-1(1)	-3(1)
C(26)	16(1)	11(1)	22(1)	-2(1)	-2(1)	-4(1)
C(27)	17(1)	11(1)	21(1)	-3(1)	-1(1)	0(1)
C(28)	20(1)	19(1)	22(1)	-4(1)	1(1)	-6(1)
C(29)	27(1)	26(1)	26(1)	-8(1)	-2(1)	-9(1)
C(30)	36(1)	26(1)	20(1)	-6(1)	-2(1)	-5(1)
C(31)	34(1)	21(1)	23(1)	0(1)	6(1)	-7(1)
C(32)	24(1)	15(1)	28(1)	-2(1)	2(1)	-6(1)
C(33)	19(1)	12(1)	18(1)	-4(1)	-3(1)	-1(1)
C(34)	21(1)	20(1)	20(1)	-2(1)	-3(1)	-5(1)
C(35)	20(1)	27(1)	23(1)	-7(1)	-5(1)	-3(1)
C(36)	27(1)	23(1)	21(1)	-3(1)	-9(1)	0(1)
C(37)	32(1)	19(1)	22(1)	1(1)	-5(1)	-5(1)
C(38)	23(1)	17(1)	22(1)	-1(1)	-3(1)	-4(1)
C(39)	17(1)	12(1)	19(1)	0(1)	-4(1)	-1(1)
C(40)	19(1)	16(1)	22(1)	0(1)	-5(1)	-5(1)
C(41)	19(1)	20(1)	25(1)	1(1)	-1(1)	-5(1)
C(42)	25(1)	21(1)	21(1)	-2(1)	1(1)	-1(1)
C(43)	27(1)	19(1)	22(1)	-5(1)	-4(1)	-4(1)
C(44)	20(1)	16(1)	22(1)	-2(1)	-5(1)	-4(1)
C(45)	19(1)	15(1)	18(1)	-1(1)	-2(1)	-6(1)
C(46)	20(1)	16(1)	22(1)	-2(1)	-2(1)	-5(1)
C(47)	18(1)	24(1)	28(1)	-2(1)	-1(1)	-6(1)
C(48)	25(1)	27(1)	25(1)	-3(1)	0(1)	-16(1)
C(49)	31(1)	17(1)	23(1)	-3(1)	0(1)	-10(1)
C(50)	22(1)	15(1)	22(1)	-2(1)	-2(1)	-4(1)
C11	54(1)	40(1)	58(1)	-23(1)	-2(1)	3(1)
N11	39(1)	32(1)	48(1)	3(1)	-10(1)	-7(1)
C21	35(1)	30(1)	26(1)	-4(1)	-4(1)	-12(1)
B12	22(1)	22(1)	23(1)	-6(1)	2(1)	-5(1)
F22	66(1)	32(1)	53(1)	-6(1)	-31(1)	13(1)
F32	27(1)	32(1)	42(1)	-8(1)	-9(1)	-3(1)
F42	42(1)	35(1)	35(1)	-16(1)	3(1)	-4(1)
F12	59(1)	80(1)	44(1)	-28(1)	21(1)	-50(1)

3.5 Crystal structure of 3a

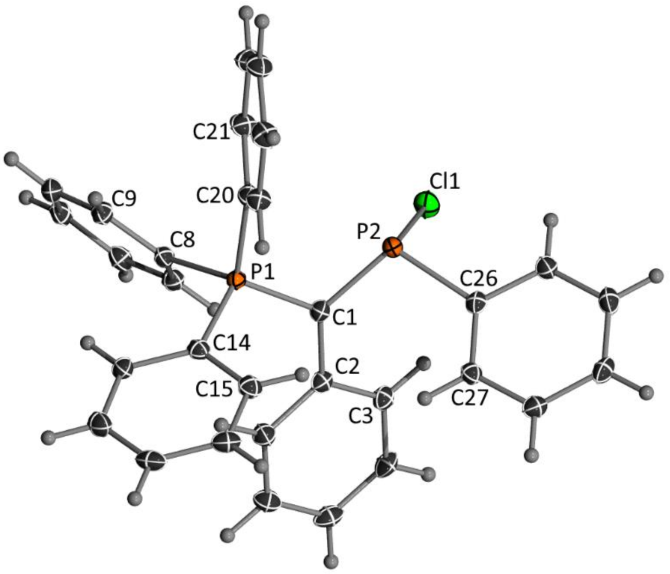

Figure S56**:** ORTEP Plot of compound **3a**. Ellipsoids are drawn at the 50% probability level.

Table C13. Crystal data and structure refinement for **3a**.

CCDC number	2121599	
Empirical formula	C31 H25 Cl P2	
Formula weight	494.90	
Temperature	100(2) K	
Wavelength	1.54184 Å	
Crystal system	Monoclinic	
Space group	$P2_1$	
Unit cell dimensions	a = 9.84156(7) Å	a= 90°.
	b = 14.39482(7) Å	b= 119.3897(10)°.
	c = 9.94133(8) Å	g = 90°.
Volume	1227.108(18) Å^3	
Z	2	
Density (calculated)	1.339 Mg/m^3	
Absorption coefficient	2.739 mm^{-1}	
F(000)	516	
Crystal size	0.358 x 0.253 x 0.152 mm^3	
Theta range for data collection	5.106 to 77.439°.	
Index ranges	-12<=h<=12, -17<=k<=17, -12<=l<=12	
Reflections collected	19208	
Independent reflections	5103 [R(int) = 0.0148]	
Completeness to theta = 67.684°	100.0 %	
Absorption correction	Gaussian	
Max. and min. transmission	1.000 and 0.278	
Refinement method	Full-matrix least-squares on F^2	
Data / restraints / parameters	5103 / 1 / 307	
Goodness-of-fit on F^2	1.048	
Final R indices [I>2sigma(I)]	R1 = 0.0207, wR2 = 0.0563	
R indices (all data)	R1 = 0.0207, wR2 = 0.0563	
Absolute structure parameter	0.004(5)	
Extinction coefficient	n/a	
Largest diff. peak and hole	0.219 and -0.266 e.Å^{-3}	

Table C14. Atomic coordinates (x 10^4) and equivalent isotropic displacement parameters (Å^{2}x 10^3) for **3a**. U(eq) is defined as one third of the trace of the orthogonalized U^{ij} tensor.

	x	y	z	U(eq)
Cl(1)	1939(1)	4076(1)	-222(1)	23(1)
P(1)	4024(1)	5826(1)	3895(1)	12(1)
P(2)	2856(1)	4091(1)	2280(1)	14(1)
C(1)	2372(2)	5164(1)	2742(2)	15(1)
C(2)	797(2)	5558(1)	2216(2)	15(1)
C(3)	-527(2)	5138(1)	996(2)	16(1)
C(4)	-2018(2)	5500(1)	486(2)	18(1)
C(5)	-2240(2)	6284(2)	1165(2)	20(1)
C(6)	-951(2)	6713(1)	2354(2)	20(1)
C(7)	535(2)	6361(1)	2867(2)	18(1)
C(8)	4219(2)	6865(1)	2967(2)	14(1)
C(9)	5658(2)	7324(1)	3565(2)	17(1)
C(10)	5822(2)	8062(1)	2757(2)	20(1)
C(11)	4556(2)	8369(1)	1387(2)	20(1)
C(12)	3118(2)	7935(1)	815(2)	19(1)
C(13)	2949(2)	7177(1)	1586(2)	17(1)
C(14)	4028(2)	6174(1)	5652(2)	15(1)
C(15)	3528(2)	5512(1)	6339(2)	18(1)
C(16)	3480(2)	5723(2)	7678(2)	20(1)
C(17)	3895(2)	6606(2)	8305(2)	22(1)
C(18)	4376(2)	7273(2)	7617(2)	22(1)
C(19)	4457(2)	7060(1)	6293(2)	17(1)

C(20)	5819(2)	5183(1)	4506(2)	15(1)
C(21)	6514(2)	5166(1)	3575(2)	19(1)
C(22)	7927(2)	4711(2)	4075(3)	23(1)
C(23)	8641(2)	4267(1)	5496(2)	22(1)
C(24)	7938(2)	4263(1)	6416(2)	21(1)
C(25)	6535(2)	4725(1)	5930(2)	18(1)
C(26)	1508(2)	3239(1)	2353(2)	14(1)
C(27)	874(2)	3361(1)	3334(2)	16(1)
C(28)	152(2)	2624(1)	3639(2)	18(1)
C(29)	78(2)	1751(1)	3004(2)	19(1)
C(30)	684(2)	1628(1)	2015(2)	20(1)
C(31)	1384(2)	2365(1)	1688(2)	18(1)

Table C15. Bond lengths [Å] and angles [°] for **3a**.

Cl(1)-P(2)	2.1914(6)
P(1)-C(1)	1.7426(19)
P(1)-C(20)	1.8150(19)
P(1)-C(14)	1.8159(19)
P(1)-C(8)	1.8160(19)
P(2)-C(1)	1.7424(19)
P(2)-C(26)	1.8340(19)
C(1)-C(2)	1.484(3)
C(2)-C(3)	1.408(3)
C(2)-C(7)	1.408(3)
C(3)-C(4)	1.396(3)
C(4)-C(5)	1.386(3)
C(5)-C(6)	1.384(3)
C(6)-C(7)	1.388(3)
C(8)-C(13)	1.402(3)
C(8)-C(9)	1.403(3)
C(9)-C(10)	1.389(3)
C(10)-C(11)	1.392(3)
C(11)-C(12)	1.388(3)
C(12)-C(13)	1.389(3)
C(14)-C(15)	1.395(3)
C(14)-C(19)	1.396(3)
C(15)-C(16)	1.388(3)
C(16)-C(17)	1.386(3)
C(17)-C(18)	1.389(3)
C(18)-C(19)	1.391(3)
C(20)-C(21)	1.395(3)
C(20)-C(25)	1.398(3)
C(21)-C(22)	1.390(3)
C(22)-C(23)	1.387(3)
C(23)-C(24)	1.391(3)
C(24)-C(25)	1.388(3)
C(26)-C(31)	1.398(3)
C(26)-C(27)	1.403(3)
C(27)-C(28)	1.392(3)
C(28)-C(29)	1.392(3)
C(29)-C(30)	1.389(3)
C(30)-C(31)	1.388(3)
C(1)-P(1)-C(20)	112.46(9)
C(1)-P(1)-C(14)	110.12(9)
C(20)-P(1)-C(14)	106.04(9)
C(1)-P(1)-C(8)	114.45(9)
C(20)-P(1)-C(8)	105.18(8)
C(14)-P(1)-C(8)	108.12(9)
C(1)-P(2)-C(26)	106.60(9)
C(1)-P(2)-Cl(1)	107.13(7)
C(26)-P(2)-Cl(1)	97.79(6)
C(2)-C(1)-P(2)	128.27(14)
C(2)-C(1)-P(1)	119.91(14)
P(2)-C(1)-P(1)	111.73(10)
C(3)-C(2)-C(7)	116.56(17)
C(3)-C(2)-C(1)	120.31(17)

C(7)-C(2)-C(1)	123.12(17)
C(4)-C(3)-C(2)	121.08(18)
C(5)-C(4)-C(3)	121.01(18)
C(6)-C(5)-C(4)	118.79(18)
C(5)-C(6)-C(7)	120.65(19)
C(6)-C(7)-C(2)	121.89(18)
C(13)-C(8)-C(9)	119.56(17)
C(13)-C(8)-P(1)	119.73(14)
C(9)-C(8)-P(1)	120.58(14)
C(10)-C(9)-C(8)	119.64(18)
C(9)-C(10)-C(11)	120.46(18)
C(12)-C(11)-C(10)	120.08(18)
C(11)-C(12)-C(13)	120.08(18)
C(12)-C(13)-C(8)	120.12(17)
C(15)-C(14)-C(19)	120.11(17)
C(15)-C(14)-P(1)	116.62(15)
C(19)-C(14)-P(1)	123.24(15)
C(16)-C(15)-C(14)	120.36(19)
C(17)-C(16)-C(15)	119.44(19)
C(16)-C(17)-C(18)	120.50(18)
C(17)-C(18)-C(19)	120.40(19)
C(18)-C(19)-C(14)	119.17(19)
C(21)-C(20)-C(25)	119.73(17)
C(21)-C(20)-P(1)	120.01(14)
C(25)-C(20)-P(1)	120.23(14)
C(22)-C(21)-C(20)	120.07(18)
C(23)-C(22)-C(21)	119.96(19)
C(22)-C(23)-C(24)	120.30(18)
C(25)-C(24)-C(23)	119.97(19)
C(24)-C(25)-C(20)	119.95(18)
C(31)-C(26)-C(27)	118.43(17)
C(31)-C(26)-P(2)	118.71(14)
C(27)-C(26)-P(2)	121.34(14)
C(28)-C(27)-C(26)	120.31(18)
C(27)-C(28)-C(29)	120.53(18)
C(30)-C(29)-C(28)	119.44(18)
C(31)-C(30)-C(29)	120.17(18)
C(30)-C(31)-C(26)	121.06(18)

Table C16. Anisotropic displacement parameters ($Å^2$ x 10^3) for **3a**. The anisotropic displacement factor exponent takes the form: $-2p^2[h^2 a^{*2}U^{11} + ... + 2 h k a^* b^* U^{12}]$

	U^{11}	U^{22}	U^{33}	U^{23}	U^{13}	U^{12}
Cl(1)	31(1)	24(1)	18(1)	-3(1)	15(1)	-5(1)
P(1)	11(1)	11(1)	14(1)	0(1)	6(1)	0(1)
P(2)	14(1)	12(1)	17(1)	-2(1)	8(1)	-1(1)
C(1)	14(1)	14(1)	16(1)	-2(1)	7(1)	-2(1)
C(2)	14(1)	15(1)	15(1)	3(1)	7(1)	1(1)
C(3)	17(1)	14(1)	18(1)	2(1)	8(1)	-1(1)
C(4)	14(1)	20(1)	18(1)	4(1)	5(1)	-2(1)
C(5)	14(1)	26(1)	22(1)	8(1)	9(1)	4(1)
C(6)	19(1)	20(1)	21(1)	2(1)	10(1)	5(1)
C(7)	15(1)	18(1)	19(1)	-2(1)	7(1)	1(1)
C(8)	16(1)	12(1)	16(1)	0(1)	9(1)	1(1)
C(9)	15(1)	15(1)	20(1)	0(1)	7(1)	0(1)
C(10)	20(1)	14(1)	28(1)	-3(1)	14(1)	-2(1)
C(11)	29(1)	12(1)	25(1)	1(1)	18(1)	1(1)
C(12)	23(1)	16(1)	16(1)	1(1)	9(1)	3(1)
C(13)	16(1)	18(1)	16(1)	-2(1)	8(1)	0(1)
C(14)	12(1)	16(1)	15(1)	-1(1)	6(1)	2(1)
C(15)	18(1)	17(1)	20(1)	1(1)	9(1)	3(1)
C(16)	18(1)	25(1)	21(1)	4(1)	12(1)	4(1)
C(17)	15(1)	32(1)	17(1)	-3(1)	8(1)	5(1)
C(18)	19(1)	23(1)	22(1)	-8(1)	9(1)	-2(1)
C(19)	14(1)	17(1)	20(1)	-3(1)	7(1)	-1(1)
C(20)	13(1)	13(1)	18(1)	-1(1)	6(1)	-1(1)
C(21)	19(1)	18(1)	20(1)	3(1)	10(1)	2(1)

C(22)	22(1)	22(1)	29(1)	2(1)	17(1)	4(1)
C(23)	18(1)	21(1)	26(1)	-1(1)	9(1)	5(1)
C(24)	19(1)	21(1)	19(1)	3(1)	6(1)	5(1)
C(25)	18(1)	19(1)	18(1)	1(1)	8(1)	2(1)
C(26)	12(1)	14(1)	15(1)	1(1)	4(1)	0(1)
C(27)	15(1)	15(1)	16(1)	-1(1)	7(1)	1(1)
C(28)	17(1)	20(1)	20(1)	2(1)	10(1)	1(1)
C(29)	16(1)	16(1)	24(1)	4(1)	8(1)	-2(1)
C(30)	20(1)	14(1)	22(1)	-2(1)	7(1)	-1(1)
C(31)	18(1)	17(1)	19(1)	-2(1)	9(1)	-1(1)

3.6 Crystal structure of 3b

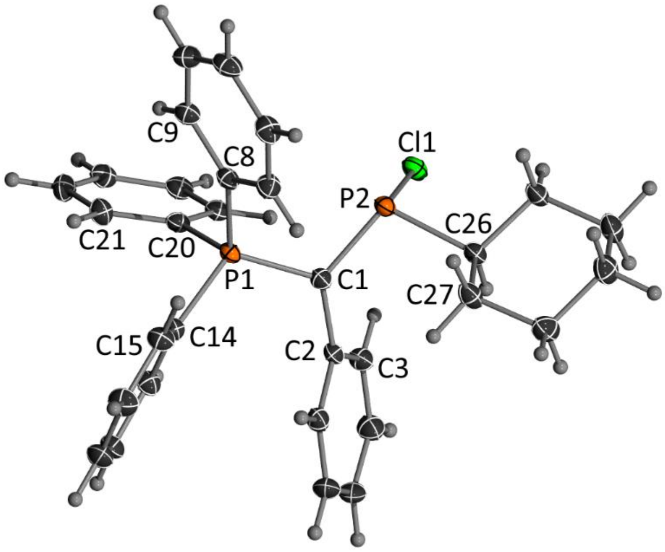

Figure S57**:** ORTEP Plot of compound **3b** Ellipsoids are drawn at the 50% probability level.

Table C17. Crystal data and structure refinement for **3b**.

CCDC number	2121593	
Empirical formula	C31 H31 Cl P2	
Formula weight	500.95	
Temperature	100(2) K	
Wavelength	1.54184 Å	
Crystal system	Monoclinic	
Space group	C2/c	
Unit cell dimensions	a = 15.7794(2) Å	a= 90°.
	b = 10.11510(10) Å	b= 90.4160(10)°.
	c = 32.7012(4) Å	g = 90°.
Volume	5219.31(11) $Å^3$	
Z	8	
Density (calculated)	1.275 Mg/m^3	
Absorption coefficient	2.576 mm^{-1}	
F(000)	2112	
Crystal size	0.480 x 0.288 x 0.241 mm^3	
Theta range for data collection	2.703 to 77.279°.	
Index ranges	-19<=h<=19, -12<=k<=6, -40<=l<=38	
Reflections collected	17888	
Independent reflections	5264 [R(int) = 0.0240]	
Completeness to theta = 67.684°	99.0 %	

Absorption correction	Gaussian
Max. and min. transmission	1.000 and 0.144
Refinement method	Full-matrix least-squares on F^2
Data / restraints / parameters	5264 / 0 / 307
Goodness-of-fit on F^2	1.070
Final R indices [I>2sigma(I)]	R1 = 0.0339, wR2 = 0.0912
R indices (all data)	R1 = 0.0343, wR2 = 0.0915
Extinction coefficient	n/a
Largest diff. peak and hole	0.448 and -0.313 e.Å^{-3}

Table C18. Atomic coordinates (x 10^4) and equivalent isotropic displacement parameters (Å^{2}x 10^3) for **3b**. U(eq) is defined as one third of the trace of the orthogonalized U^{ij} tensor.

	x	y	z	U(eq)
Cl(1)	5083(1)	3664(1)	3682(1)	20(1)
P(1)	3545(1)	6900(1)	4105(1)	14(1)
P(2)	3826(1)	4660(1)	3563(1)	16(1)
C(1)	3946(1)	6348(1)	3643(1)	16(1)
C(2)	4506(1)	7272(1)	3410(1)	16(1)
C(3)	5368(1)	6990(1)	3358(1)	20(1)
C(4)	5901(1)	7860(2)	3157(1)	24(1)
C(5)	5590(1)	9039(2)	3002(1)	26(1)
C(6)	4736(1)	9327(2)	3042(1)	26(1)
C(7)	4196(1)	8450(2)	3240(1)	20(1)
C(8)	2637(1)	5917(1)	4250(1)	16(1)
C(9)	2581(1)	5352(1)	4638(1)	20(1)
C(10)	1883(1)	4580(2)	4739(1)	24(1)
C(11)	1235(1)	4389(2)	4456(1)	24(1)
C(12)	1286(1)	4954(2)	4070(1)	23(1)
C(13)	1984(1)	5709(2)	3964(1)	21(1)
C(14)	3217(1)	8620(1)	4088(1)	16(1)
C(15)	2377(1)	8991(2)	4025(1)	20(1)
C(16)	2170(1)	10321(2)	3977(1)	26(1)
C(17)	2797(1)	11283(2)	3986(1)	25(1)
C(18)	3636(1)	10920(2)	4049(1)	22(1)
C(19)	3845(1)	9601(1)	4105(1)	19(1)
C(20)	4302(1)	6865(1)	4526(1)	17(1)
C(21)	4144(1)	7482(2)	4902(1)	21(1)
C(22)	4741(1)	7418(2)	5216(1)	24(1)
C(23)	5506(1)	6770(2)	5154(1)	22(1)
C(24)	5674(1)	6185(1)	4780(1)	20(1)
C(25)	5070(1)	6216(1)	4467(1)	18(1)
C(26)	3887(1)	4467(1)	3000(1)	17(1)
C(27)	3143(1)	5234(2)	2807(1)	22(1)
C(28)	3086(1)	4997(2)	2345(1)	26(1)
C(29)	3011(1)	3534(2)	2245(1)	26(1)
C(30)	3757(1)	2774(2)	2429(1)	26(1)
C(31)	3822(1)	2992(1)	2891(1)	21(1)

Table C19. Bond lengths [Å] and angles [°] for **3b**.

Cl(1)-P(2)	2.2560(5)
P(1)-C(1)	1.7359(14)
P(1)-C(8)	1.8096(14)
P(1)-C(14)	1.8158(14)
P(1)-C(20)	1.8166(14)
P(2)-C(1)	1.7382(14)
P(2)-C(26)	1.8568(14)
C(1)-C(2)	1.4984(19)
C(2)-C(3)	1.400(2)
C(2)-C(7)	1.401(2)

C(3)-C(4)	1.387(2)
C(4)-C(5)	1.385(2)
C(5)-C(6)	1.385(2)
C(6)-C(7)	1.393(2)
C(8)-C(9)	1.392(2)
C(8)-C(13)	1.403(2)
C(9)-C(10)	1.392(2)
C(10)-C(11)	1.388(2)
C(11)-C(12)	1.389(2)
C(12)-C(13)	1.387(2)
C(14)-C(15)	1.391(2)
C(14)-C(19)	1.402(2)
C(15)-C(16)	1.392(2)
C(16)-C(17)	1.387(2)
C(17)-C(18)	1.388(2)
C(18)-C(19)	1.387(2)
C(20)-C(25)	1.393(2)
C(20)-C(21)	1.401(2)
C(21)-C(22)	1.390(2)
C(22)-C(23)	1.390(2)
C(23)-C(24)	1.385(2)
C(24)-C(25)	1.393(2)
C(26)-C(31)	1.5364(19)
C(26)-C(27)	1.539(2)
C(27)-C(28)	1.531(2)
C(28)-C(29)	1.520(2)
C(29)-C(30)	1.526(2)
C(30)-C(31)	1.530(2)
C(1)-P(1)-C(8)	110.23(7)
C(1)-P(1)-C(14)	112.70(7)
C(8)-P(1)-C(14)	108.01(6)
C(1)-P(1)-C(20)	114.34(7)
C(8)-P(1)-C(20)	107.91(7)
C(14)-P(1)-C(20)	103.22(6)
C(1)-P(2)-C(26)	104.20(7)
C(1)-P(2)-Cl(1)	108.57(5)
C(26)-P(2)-Cl(1)	94.18(5)
C(2)-C(1)-P(1)	117.54(10)
C(2)-C(1)-P(2)	126.93(10)
P(1)-C(1)-P(2)	114.00(8)
C(3)-C(2)-C(7)	117.50(13)
C(3)-C(2)-C(1)	120.73(13)
C(7)-C(2)-C(1)	121.77(13)
C(4)-C(3)-C(2)	121.38(14)
C(5)-C(4)-C(3)	120.30(15)
C(4)-C(5)-C(6)	119.38(14)
C(5)-C(6)-C(7)	120.45(14)
C(6)-C(7)-C(2)	120.93(14)
C(9)-C(8)-C(13)	119.58(13)
C(9)-C(8)-P(1)	121.33(11)
C(13)-C(8)-P(1)	119.08(11)
C(8)-C(9)-C(10)	120.18(14)
C(11)-C(10)-C(9)	119.98(14)
C(10)-C(11)-C(12)	120.12(14)
C(13)-C(12)-C(11)	120.26(14)
C(12)-C(13)-C(8)	119.85(14)
C(15)-C(14)-C(19)	119.16(13)
C(15)-C(14)-P(1)	122.27(11)
C(19)-C(14)-P(1)	118.37(11)
C(14)-C(15)-C(16)	119.93(14)
C(17)-C(16)-C(15)	120.59(14)
C(16)-C(17)-C(18)	119.78(14)
C(19)-C(18)-C(17)	119.97(14)
C(18)-C(19)-C(14)	120.53(14)
C(25)-C(20)-C(21)	119.52(13)
C(25)-C(20)-P(1)	118.16(11)
C(21)-C(20)-P(1)	122.32(11)
C(22)-C(21)-C(20)	120.13(14)
C(21)-C(22)-C(23)	119.98(14)
C(24)-C(23)-C(22)	120.09(14)
C(23)-C(24)-C(25)	120.31(14)
C(20)-C(25)-C(24)	119.95(14)

C(31)-C(26)-C(27)	110.20(12)
C(31)-C(26)-P(2)	109.05(10)
C(27)-C(26)-P(2)	108.02(10)
C(28)-C(27)-C(26)	111.40(13)
C(29)-C(28)-C(27)	111.66(13)
C(28)-C(29)-C(30)	110.30(13)
C(29)-C(30)-C(31)	111.35(13)
C(30)-C(31)-C(26)	111.82(12)

Table C20. Anisotropic displacement parameters ($Å^2$x 10^3) for **3b**. The anisotropic displacement factor exponent takes the form: $-2p^2[h^2 a^{*2}U^{11} + ... + 2 h k a^* b^* U^{12}]$

	U^{11}	U^{22}	U^{33}	U^{23}	U^{13}	U^{12}
Cl(1)	22(1)	15(1)	23(1)	1(1)	-3(1)	2(1)
P(1)	13(1)	13(1)	17(1)	-1(1)	0(1)	-1(1)
P(2)	17(1)	12(1)	19(1)	0(1)	1(1)	-1(1)
C(1)	16(1)	14(1)	18(1)	-1(1)	1(1)	0(1)
C(2)	19(1)	13(1)	16(1)	-2(1)	0(1)	-2(1)
C(3)	20(1)	16(1)	22(1)	1(1)	1(1)	0(1)
C(4)	22(1)	23(1)	28(1)	-1(1)	5(1)	-3(1)
C(5)	35(1)	19(1)	24(1)	2(1)	6(1)	-9(1)
C(6)	39(1)	16(1)	22(1)	4(1)	1(1)	1(1)
C(7)	24(1)	17(1)	19(1)	-1(1)	-1(1)	3(1)
C(8)	14(1)	13(1)	22(1)	-1(1)	2(1)	-1(1)
C(9)	19(1)	16(1)	23(1)	1(1)	0(1)	1(1)
C(10)	24(1)	20(1)	28(1)	7(1)	3(1)	0(1)
C(11)	19(1)	18(1)	36(1)	1(1)	5(1)	-4(1)
C(12)	18(1)	23(1)	28(1)	-4(1)	0(1)	-4(1)
C(13)	20(1)	21(1)	21(1)	-1(1)	1(1)	-2(1)
C(14)	18(1)	15(1)	16(1)	-1(1)	1(1)	1(1)
C(15)	17(1)	19(1)	25(1)	1(1)	0(1)	-1(1)
C(16)	20(1)	25(1)	32(1)	2(1)	0(1)	6(1)
C(17)	32(1)	16(1)	26(1)	2(1)	2(1)	5(1)
C(18)	27(1)	16(1)	23(1)	-2(1)	1(1)	-4(1)
C(19)	17(1)	19(1)	22(1)	-2(1)	0(1)	-1(1)
C(20)	17(1)	14(1)	19(1)	1(1)	-2(1)	-2(1)
C(21)	21(1)	20(1)	23(1)	-3(1)	0(1)	2(1)
C(22)	30(1)	22(1)	19(1)	-4(1)	-3(1)	0(1)
C(23)	24(1)	18(1)	22(1)	1(1)	-7(1)	-3(1)
C(24)	18(1)	18(1)	26(1)	2(1)	-2(1)	0(1)
C(25)	18(1)	15(1)	20(1)	1(1)	0(1)	-2(1)
C(26)	18(1)	13(1)	19(1)	-1(1)	-1(1)	-1(1)
C(27)	25(1)	16(1)	26(1)	-2(1)	-6(1)	3(1)
C(28)	32(1)	22(1)	24(1)	2(1)	-7(1)	1(1)
C(29)	28(1)	26(1)	24(1)	-5(1)	-6(1)	-2(1)
C(30)	30(1)	20(1)	26(1)	-7(1)	-3(1)	2(1)
C(31)	25(1)	14(1)	24(1)	-2(1)	-2(1)	1(1)

3.7 Crystal structure of 4a

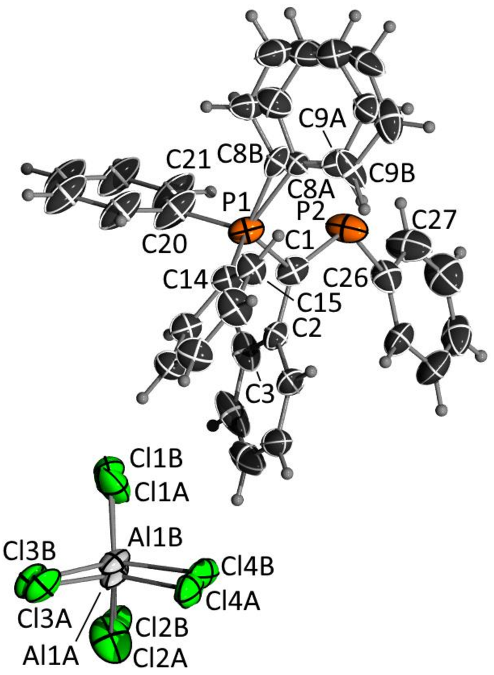

Figure S58**:** ORTEP Plot of compound **YPPhAlCl$_4$**. Ellipsoids are drawn at the 50% probability level.

Table C21. Crystal data and structure refinement for **4a**.

CCDC number	2121594	
Empirical formula	C31 H25 Al Cl4 P2	
Formula weight	628.23	
Temperature	100(2) K	
Wavelength	1.54184 Å	
Crystal system	Monoclinic	
Space group	Pn	
Unit cell dimensions	a = 10.4942(3) Å	a= 90°.
	b = 10.4579(3) Å	b= 97.132(2)°.
	c = 14.3996(4) Å	g = 90°.
Volume	1568.09(8) Å^3	
Z	2	
Density (calculated)	1.331 Mg/m^3	
Absorption coefficient	4.814 mm^{-1}	
F(000)	644	
Crystal size	0.505 x 0.186 x 0.122 mm^3	
Theta range for data collection	4.227 to 77.176°.	
Index ranges	-13<=h<=11, -12<=k<=12, -12<=l<=18	
Reflections collected	11649	
Independent reflections	3949 [R(int) = 0.0444]	
Completeness to theta = 67.684°	99.9 %	
Absorption correction	Gaussian	
Max. and min. transmission	1.000 and 0.192	
Refinement method	Full-matrix least-squares on F^2	
Data / restraints / parameters	3949 / 194 / 415	
Goodness-of-fit on F^2	1.045	
Final R indices [I>2sigma(I)]	R1 = 0.0476, wR2 = 0.1225	
R indices (all data)	R1 = 0.0478, wR2 = 0.1226	
Absolute structure parameter	0.06(3)	
Extinction coefficient	n/a	
Largest diff. peak and hole	0.589 and -0.657 e.Å^{-3}	

Table C22. Atomic coordinates (x 10^4) and equivalent isotropic displacement parameters ($Å^2$x 10^3) for **4a**. U(eq) is defined as one third of the trace of the orthogonalized U^{ij} tensor.

	x	y	z	U(eq)
P(1)	-7517(1)	7582(1)	-7087(2)	36(1)
P(2)	-8013(2)	9394(2)	-8654(2)	53(1)
C(1)	-7232(5)	9126(5)	-7586(4)	36(1)
C(2)	-6279(4)	9957(4)	-7027(4)	33(1)
C(3)	-5067(5)	10130(6)	-7294(4)	46(1)
C(4)	-4201(6)	10964(8)	-6760(5)	65(2)
C(5)	-4540(7)	11576(6)	-5983(5)	62(2)
C(6)	-5724(7)	11408(5)	-5722(5)	54(2)
C(7)	-6594(5)	10605(5)	-6232(4)	42(1)
C(8A)	-9150(20)	7080(20)	-7710(13)	27(4)
C(9A)	-10290(20)	7700(30)	-7635(17)	41(5)
C(10A)	-11420(20)	7370(30)	-8150(20)	60(6)
C(11A)	-11450(30)	6230(30)	-8682(15)	62(6)
C(12A)	-10310(30)	5490(30)	-8711(17)	60(5)
C(13A)	-9240(30)	5910(20)	-8229(18)	58(5)
C(8B)	-8845(18)	6800(15)	-7632(14)	36(3)
C(9B)	-9990(20)	7529(17)	-7779(14)	37(4)
C(10B)	-11100(18)	6956(17)	-8241(12)	46(4)
C(11B)	-10990(20)	5745(18)	-8637(13)	52(4)
C(12B)	-9850(20)	5058(19)	-8502(15)	60(4)
C(13B)	-8802(17)	5618(16)	-8026(12)	45(4)
C(14)	-7554(5)	7699(4)	-5848(4)	35(1)
C(15)	-8683(5)	7502(5)	-5443(4)	42(1)
C(16)	-8656(6)	7591(6)	-4488(4)	51(1)
C(17)	-7534(6)	7846(6)	-3933(4)	49(1)
C(18)	-6397(5)	8025(5)	-4322(4)	43(1)
C(19)	-6412(5)	7949(5)	-5266(4)	39(1)
C(20)	-6196(8)	6555(6)	-7255(6)	65(1)
C(21)	-5613(8)	6634(6)	-8064(6)	65(1)
C(22)	-4596(7)	5847(6)	-8203(6)	65(1)
C(23)	-4142(7)	4977(6)	-7526(5)	65(1)
C(24)	-4729(8)	4862(6)	-6717(6)	65(1)
C(25)	-5758(7)	5645(6)	-6581(6)	65(1)
C(26)	-7562(5)	10971(5)	-9040(4)	43(1)
C(27)	-7586(8)	11096(7)	-9989(5)	70(2)
C(28)	-7357(7)	12256(7)	-10396(6)	70(2)
C(29)	-7083(7)	13302(6)	-9850(7)	70(2)
C(30)	-7028(8)	13199(6)	-8909(7)	74(2)
C(31)	-7286(6)	12053(5)	-8471(5)	51(1)
Cl(1A)	-180(20)	11270(20)	-6927(14)	76(4)
Cl(2A)	1560(11)	13802(13)	-5842(13)	76(3)
Cl(3A)	700(20)	11381(19)	-4631(15)	55(3)
Cl(4A)	-1517(9)	13322(8)	-5388(13)	57(2)
Al(1A)	224(19)	12499(18)	-5783(14)	45(3)
Cl(1B)	-249(12)	11018(9)	-7039(9)	48(2)
Cl(2B)	1565(7)	13727(6)	-6177(7)	54(2)
Cl(3B)	877(15)	11171(14)	-4637(9)	43(2)
Cl(4B)	-1641(5)	13371(9)	-5824(11)	62(2)
Al(1B)	82(13)	12285(14)	-5878(9)	35(2)

Table C23. Bond lengths [Å] and angles [°] for **4a**.

P(1)-C(8B)	1.720(18)
P(1)-C(20)	1.794(7)
P(1)-C(14)	1.794(5)
P(1)-C(1)	1.807(5)
P(1)-C(8A)	1.912(19)
P(2)-C(1)	1.673(5)
P(2)-C(26)	1.822(5)
C(1)-C(2)	1.485(6)
C(2)-C(3)	1.385(7)
C(2)-C(7)	1.404(7)
C(3)-C(4)	1.416(9)
C(4)-C(5)	1.373(11)

C(5)-C(6)	1.353(10)
C(6)-C(7)	1.383(7)
C(8A)-C(9A)	1.38(2)
C(8A)-C(13A)	1.43(3)
C(9A)-C(10A)	1.36(3)
C(10A)-C(11A)	1.42(3)
C(11A)-C(12A)	1.43(3)
C(12A)-C(13A)	1.33(3)
C(8B)-C(13B)	1.36(2)
C(8B)-C(9B)	1.41(2)
C(9B)-C(10B)	1.41(2)
C(10B)-C(11B)	1.40(2)
C(11B)-C(12B)	1.39(2)
C(12B)-C(13B)	1.35(2)
C(14)-C(15)	1.398(7)
C(14)-C(19)	1.398(7)
C(15)-C(16)	1.375(8)
C(16)-C(17)	1.365(8)
C(17)-C(18)	1.392(8)
C(18)-C(19)	1.361(7)
C(20)-C(21)	1.383(9)
C(20)-C(25)	1.396(10)
C(21)-C(22)	1.382(9)
C(22)-C(23)	1.374(10)
C(23)-C(24)	1.389(9)
C(24)-C(25)	1.389(9)
C(26)-C(27)	1.370(9)
C(26)-C(31)	1.406(8)
C(27)-C(28)	1.381(9)
C(28)-C(29)	1.357(11)
C(29)-C(30)	1.354(12)
C(30)-C(31)	1.396(10)
Cl(1A)-Al(1A)	2.09(2)
Cl(2A)-Al(1A)	1.96(2)
Cl(3A)-Al(1A)	2.04(3)
Cl(4A)-Al(1A)	2.16(2)
Cl(1B)-Al(1B)	2.128(16)
Cl(2B)-Al(1B)	2.247(16)
Cl(3B)-Al(1B)	2.208(19)
Cl(4B)-Al(1B)	2.144(15)
C(8B)-P(1)-C(20)	104.5(6)
C(8B)-P(1)-C(14)	111.7(6)
C(20)-P(1)-C(14)	106.8(3)
C(8B)-P(1)-C(1)	114.1(7)
C(20)-P(1)-C(1)	108.2(3)
C(14)-P(1)-C(1)	111.1(2)
C(20)-P(1)-C(8A)	115.8(8)
C(14)-P(1)-C(8A)	110.9(7)
C(1)-P(1)-C(8A)	104.1(6)
C(1)-P(2)-C(26)	108.4(3)
C(2)-C(1)-P(2)	128.4(4)
C(2)-C(1)-P(1)	116.1(3)
P(2)-C(1)-P(1)	115.4(3)
C(3)-C(2)-C(7)	118.7(5)
C(3)-C(2)-C(1)	120.3(5)
C(7)-C(2)-C(1)	120.9(4)
C(2)-C(3)-C(4)	118.6(6)
C(5)-C(4)-C(3)	121.0(6)
C(6)-C(5)-C(4)	120.4(5)
C(5)-C(6)-C(7)	119.9(6)
C(6)-C(7)-C(2)	121.3(5)
C(9A)-C(8A)-C(13A)	116.7(19)
C(9A)-C(8A)-P(1)	124.8(17)
C(13A)-C(8A)-P(1)	118.3(16)
C(10A)-C(9A)-C(8A)	123(2)
C(9A)-C(10A)-C(11A)	118(2)
C(10A)-C(11A)-C(12A)	121.0(17)
C(13A)-C(12A)-C(11A)	118(2)
C(12A)-C(13A)-C(8A)	124(2)
C(13B)-C(8B)-C(9B)	119.9(15)
C(13B)-C(8B)-P(1)	123.6(12)
C(9B)-C(8B)-P(1)	115.8(12)

C(10B)-C(9B)-C(8B)	118.7(14)
C(11B)-C(10B)-C(9B)	118.4(15)
C(12B)-C(11B)-C(10B)	121.4(16)
C(13B)-C(12B)-C(11B)	118.6(15)
C(12B)-C(13B)-C(8B)	122.4(14)
C(15)-C(14)-C(19)	119.1(5)
C(15)-C(14)-P(1)	121.9(4)
C(19)-C(14)-P(1)	119.0(4)
C(16)-C(15)-C(14)	119.5(5)
C(17)-C(16)-C(15)	120.6(5)
C(16)-C(17)-C(18)	120.7(5)
C(19)-C(18)-C(17)	119.4(5)
C(18)-C(19)-C(14)	120.8(5)
C(21)-C(20)-C(25)	119.0(6)
C(21)-C(20)-P(1)	120.5(6)
C(25)-C(20)-P(1)	120.5(5)
C(22)-C(21)-C(20)	121.1(7)
C(23)-C(22)-C(21)	119.8(6)
C(22)-C(23)-C(24)	120.1(6)
C(25)-C(24)-C(23)	120.0(7)
C(24)-C(25)-C(20)	119.9(6)
C(27)-C(26)-C(31)	118.8(5)
C(27)-C(26)-P(2)	114.7(5)
C(31)-C(26)-P(2)	126.4(4)
C(26)-C(27)-C(28)	121.6(7)
C(29)-C(28)-C(27)	119.9(7)
C(30)-C(29)-C(28)	119.6(6)
C(29)-C(30)-C(31)	122.3(6)
C(30)-C(31)-C(26)	117.7(6)
Cl(2A)-Al(1A)-Cl(3A)	109.3(11)
Cl(2A)-Al(1A)-Cl(1A)	117.9(12)
Cl(3A)-Al(1A)-Cl(1A)	107.2(11)
Cl(2A)-Al(1A)-Cl(4A)	111.5(9)
Cl(3A)-Al(1A)-Cl(4A)	98.3(11)
Cl(1A)-Al(1A)-Cl(4A)	110.8(10)
Cl(1B)-Al(1B)-Cl(4B)	107.6(7)
Cl(1B)-Al(1B)-Cl(3B)	108.4(8)
Cl(4B)-Al(1B)-Cl(3B)	119.1(9)
Cl(1B)-Al(1B)-Cl(2B)	108.4(6)
Cl(4B)-Al(1B)-Cl(2B)	105.0(8)
Cl(3B)-Al(1B)-Cl(2B)	107.9(7)

Table C24. Anisotropic displacement parameters ($Å^2$x 10^3) for **4a**. The anisotropic displacement factor exponent takes the form: $-2p^2[h^2 a^{*2}U^{11} + ... + 2 h k a^* b^* U^{12}]$

	U^{11}	U^{22}	U^{33}	U^{23}	U^{13}	U^{12}
P(1)	45(1)	35(1)	32(1)	-3(1)	20(1)	-11(1)
P(2)	72(1)	53(1)	33(1)	6(1)	4(1)	-29(1)
C(1)	40(2)	37(2)	36(2)	-2(2)	23(2)	-5(2)
C(2)	33(2)	30(2)	40(2)	2(2)	11(2)	2(2)
C(3)	29(2)	62(3)	48(3)	19(2)	11(2)	1(2)
C(4)	33(3)	93(5)	66(4)	44(4)	-5(2)	-18(3)
C(5)	66(4)	57(3)	55(4)	21(3)	-19(3)	-20(3)
C(6)	70(4)	38(2)	48(3)	-4(2)	-12(3)	1(2)
C(7)	42(3)	41(2)	41(3)	-9(2)	4(2)	7(2)
C(8A)	27(6)	28(6)	26(5)	6(4)	4(4)	-7(5)
C(9A)	14(8)	84(12)	26(8)	11(7)	11(6)	-2(7)
C(10A)	46(10)	63(12)	72(11)	35(10)	6(8)	2(8)
C(11A)	64(12)	66(12)	49(8)	15(9)	-22(8)	-35(10)
C(12A)	61(13)	51(11)	65(10)	-10(9)	-1(10)	-20(10)
C(13A)	50(11)	61(11)	63(10)	-3(8)	3(8)	-11(8)
C(8B)	41(8)	30(6)	41(6)	1(5)	19(5)	9(5)
C(9B)	34(10)	42(6)	40(7)	-6(4)	23(6)	-9(6)
C(10B)	44(8)	44(8)	48(6)	4(6)	-1(6)	-4(6)
C(11B)	58(11)	46(8)	52(7)	-6(6)	9(8)	-7(8)
C(12B)	59(10)	49(8)	73(10)	-22(7)	13(7)	-8(7)
C(13B)	42(8)	48(7)	48(8)	-17(5)	13(6)	-9(6)
C(14)	37(2)	36(2)	35(2)	-2(2)	13(2)	-6(2)

C(15)	34(2)	54(3)	40(3)	2(2)	14(2)	0(2)
C(16)	44(3)	75(4)	41(3)	6(3)	22(2)	-2(3)
C(17)	57(3)	63(3)	29(2)	1(2)	18(2)	-4(3)
C(18)	42(3)	48(3)	38(3)	0(2)	6(2)	-2(2)
C(19)	38(2)	42(2)	41(3)	-2(2)	16(2)	-4(2)
C(20)	90(2)	36(1)	81(2)	-1(1)	56(2)	1(1)
C(21)	90(2)	36(1)	81(2)	-1(1)	56(2)	1(1)
C(22)	90(2)	36(1)	81(2)	-1(1)	56(2)	1(1)
C(23)	90(2)	36(1)	81(2)	-1(1)	56(2)	1(1)
C(24)	90(2)	36(1)	81(2)	-1(1)	56(2)	1(1)
C(25)	90(2)	36(1)	81(2)	-1(1)	56(2)	1(1)
C(26)	35(2)	47(3)	48(3)	11(2)	7(2)	-8(2)
C(27)	97(5)	60(4)	52(4)	10(3)	5(3)	-34(4)
C(28)	75(5)	73(4)	63(4)	31(4)	9(3)	-18(4)
C(29)	54(4)	44(3)	108(6)	29(3)	-3(3)	-5(3)
C(30)	87(5)	32(3)	99(6)	-3(3)	-1(4)	9(3)
C(31)	56(3)	37(3)	63(4)	-3(2)	12(3)	12(2)
Cl(1A)	67(4)	108(10)	48(5)	-3(6)	-15(3)	8(7)
Cl(2A)	51(3)	89(5)	90(7)	38(4)	17(4)	-24(3)
Cl(3A)	67(6)	47(6)	56(4)	-13(3)	32(3)	12(4)
Cl(4A)	36(2)	47(2)	90(7)	-3(3)	12(3)	9(2)
Al(1A)	24(4)	20(5)	92(7)	-2(4)	8(3)	-6(3)
Cl(1B)	52(3)	46(2)	44(3)	-14(2)	-2(2)	10(2)
Cl(2B)	49(2)	38(2)	80(4)	13(2)	30(3)	-2(1)
Cl(3B)	60(4)	38(4)	37(2)	2(2)	28(2)	10(3)
Cl(4B)	32(2)	55(2)	99(6)	-35(3)	4(2)	11(1)
Al(1B)	26(3)	22(4)	60(3)	-5(2)	19(2)	-8(2)

3.8 Crystal structure of 4b

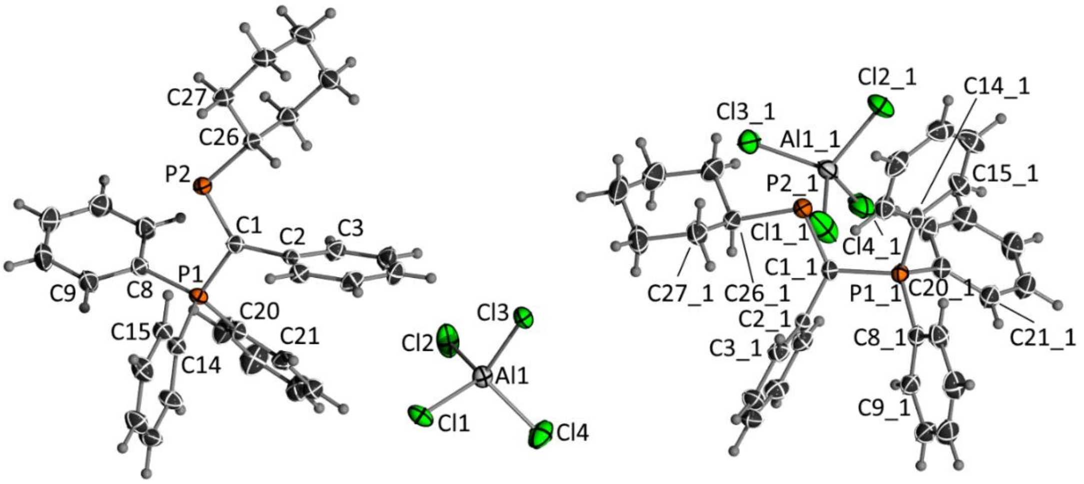

Figure S59**:** ORTEP Plot of compound **4b**. Ellipsoids are drawn at the 50% probability level.

Table C25. Crystal data and structure refinement for **4b**.

Identification code	2121596	
Empirical formula	C31 H31 Al1 Cl4 P2	
Formula weight	5074.21	
Temperature	110(2) K	
Wavelength	1.54184 Å	
Crystal system	Monoclinic	
Space group	$P2_1/c$	
Unit cell dimensions	a = 20.64420(10) Å	a= 90°.
	b = 20.23140(10) Å	b= 93.4520(10)°.
	c = 15.39730(10) Å	g = 90°.
Volume	6419.19(6) $Å^3$	

Z	8
Density (calculated)	1.313 Mg/m^3
Absorption coefficient	4.704 mm^{-1}
F(000)	2624
Crystal size	0.617 x 0.316 x 0.250 mm^3
Theta range for data collection	3.061 to 77.548°.
Index ranges	-25<=h<=25, -23<=k<=25, -19<=l<=18
Reflections collected	56356
Independent reflections	13133 [R(int) = 0.0357]
Completeness to theta = 67.684°	99.7 %
Absorption correction	Gaussian
Max. and min. transmission	1.000 and 0.243
Refinement method	Full-matrix least-squares on F^2
Data / restraints / parameters	13133 / 0 / 685
Goodness-of-fit on F^2	1.065
Final R indices [I>2sigma(I)]	R1 = 0.0437, wR2 = 0.1141
R indices (all data)	R1 = 0.0447, wR2 = 0.1151
Extinction coefficient	n/a
Largest diff. peak and hole	1.102 and -0.741 e.Å^{-3}

Table C26. Atomic coordinates (x 10^4) and equivalent isotropic displacement parameters (Å^{2}x 10^3) for **4b**. U(eq) is defined as one third of the trace of the orthogonalized U^{ij} tensor.

	x	y	z	U(eq)
Cl(1)	8894(1)	6583(1)	2781(1)	29(1)
Cl(2)	9433(1)	5001(1)	3314(1)	43(1)
Cl(3)	7771(1)	5336(1)	3263(1)	31(1)
Cl(4)	8556(1)	5269(1)	1356(1)	42(1)
P(1)	10695(1)	7117(1)	6190(1)	18(1)
P(2)	9988(1)	6839(1)	7762(1)	24(1)
Al(1)	8662(1)	5553(1)	2692(1)	23(1)
C(1)	9962(1)	6858(1)	6665(1)	21(1)
C(2)	9381(1)	6710(1)	6069(1)	19(1)
C(3)	8906(1)	7191(1)	5898(1)	24(1)
C(4)	8344(1)	7036(1)	5394(1)	28(1)
C(5)	8250(1)	6409(1)	5060(1)	30(1)
C(6)	8724(1)	5933(1)	5214(1)	31(1)
C(7)	9289(1)	6080(1)	5707(1)	25(1)
C(8)	11354(1)	7094(1)	7012(1)	22(1)
C(9)	11661(1)	7678(1)	7282(2)	31(1)
C(10)	12153(1)	7654(1)	7935(2)	42(1)
C(11)	12346(1)	7057(2)	8299(2)	40(1)
C(12)	12046(1)	6475(1)	8031(2)	34(1)
C(13)	11543(1)	6492(1)	7396(2)	29(1)
C(14)	10605(1)	7952(1)	5798(1)	21(1)
C(15)	10233(1)	8404(1)	6235(1)	24(1)
C(16)	10206(1)	9059(1)	5965(2)	31(1)
C(17)	10542(1)	9258(1)	5264(2)	33(1)
C(18)	10907(1)	8811(1)	4820(2)	33(1)
C(19)	10942(1)	8155(1)	5083(2)	28(1)
C(20)	10881(1)	6619(1)	5271(1)	21(1)
C(21)	10454(1)	6620(1)	4529(1)	26(1)
C(22)	10602(1)	6263(1)	3803(1)	31(1)
C(23)	11171(1)	5904(1)	3808(2)	37(1)
C(24)	11593(1)	5901(1)	4537(2)	42(1)
C(25)	11452(1)	6257(1)	5274(2)	33(1)
C(26)	9186(1)	6516(1)	8024(1)	22(1)
C(27)	9032(1)	6732(1)	8945(1)	26(1)
C(28)	8387(1)	6434(1)	9200(1)	28(1)
C(29)	8372(1)	5685(1)	9093(2)	34(1)
C(30)	8553(1)	5468(1)	8192(2)	35(1)
C(31)	9203(1)	5757(1)	7964(1)	27(1)
Cl11	6295(1)	1534(1)	1651(1)	63(1)
Cl21	5830(1)	117(1)	2728(1)	43(1)

Cl31	7363(1)	865(1)	3247(1)	36(1)
Cl41	7062(1)	15(1)	1314(1)	52(1)
P11	4212(1)	2860(1)	804(1)	21(1)
P21	5003(1)	2985(1)	2465(1)	33(1)
Al11	6639(1)	639(1)	2248(1)	31(1)
C11	4923(1)	3170(1)	1397(1)	24(1)
C21	5401(1)	3532(1)	884(1)	21(1)
C31	5340(1)	4212(1)	776(1)	26(1)
C41	5781(1)	4560(1)	312(2)	31(1)
C51	6283(1)	4231(1)	-54(2)	34(1)
C61	6344(1)	3555(1)	36(2)	32(1)
C71	5905(1)	3200(1)	509(1)	26(1)
C81	3971(1)	3377(1)	-114(1)	25(1)
C91	4390(1)	3459(1)	-786(1)	29(1)
C101	4192(1)	3829(1)	-1513(2)	36(1)
C111	3579(1)	4108(1)	-1576(2)	41(1)
C121	3162(1)	4028(1)	-917(2)	40(1)
C131	3353(1)	3663(1)	-179(2)	32(1)
C141	3567(1)	2798(1)	1525(1)	25(1)
C151	3224(1)	2204(1)	1587(2)	31(1)
C161	2735(1)	2156(1)	2159(2)	40(1)
C171	2587(1)	2685(2)	2666(2)	40(1)
C181	2917(1)	3279(2)	2607(2)	38(1)
C191	3408(1)	3340(1)	2037(2)	31(1)
C201	4355(1)	2041(1)	398(1)	24(1)
C211	4075(1)	1838(1)	-409(2)	31(1)
C221	4109(1)	1181(1)	-649(2)	36(1)
C231	4414(1)	724(1)	-92(2)	38(1)
C241	4706(1)	926(1)	698(2)	37(1)
C251	4681(1)	1584(1)	942(2)	30(1)
C261	5797(1)	3345(1)	2838(1)	31(1)
C271	5705(1)	4074(1)	3073(2)	37(1)
C281	6354(1)	4366(2)	3429(2)	45(1)
C291	6633(1)	3970(2)	4205(2)	52(1)
C301	6713(2)	3243(2)	3987(2)	53(1)
C311	6072(1)	2944(2)	3624(2)	48(1)

Table C27. Bond lengths [Å] and angles [°] for **4b**.

Cl(1)-Al(1)	2.1412(8)
Cl(2)-Al(1)	2.1251(8)
Cl(3)-Al(1)	2.1319(8)
Cl(4)-Al(1)	2.1338(8)
P(1)-C(1)	1.797(2)
P(1)-C(20)	1.797(2)
P(1)-C(14)	1.799(2)
P(1)-C(8)	1.801(2)
P(2)-C(1)	1.688(2)
P(2)-C(26)	1.847(2)
C(1)-C(2)	1.497(3)
C(2)-C(3)	1.396(3)
C(2)-C(7)	1.398(3)
C(3)-C(4)	1.393(3)
C(4)-C(5)	1.378(3)
C(5)-C(6)	1.384(3)
C(6)-C(7)	1.386(3)
C(8)-C(9)	1.392(3)
C(8)-C(13)	1.400(3)
C(9)-C(10)	1.387(3)
C(10)-C(11)	1.381(4)
C(11)-C(12)	1.381(4)
C(12)-C(13)	1.383(3)
C(14)-C(15)	1.392(3)
C(14)-C(19)	1.400(3)
C(15)-C(16)	1.389(3)
C(16)-C(17)	1.379(3)
C(17)-C(18)	1.384(3)
C(18)-C(19)	1.388(3)

C(20)-C(25)	1.387(3)
C(20)-C(21)	1.401(3)
C(21)-C(22)	1.381(3)
C(22)-C(23)	1.380(3)
C(23)-C(24)	1.378(4)
C(24)-C(25)	1.390(3)
C(26)-C(27)	1.536(3)
C(26)-C(31)	1.538(3)
C(27)-C(28)	1.535(3)
C(28)-C(29)	1.525(3)
C(29)-C(30)	1.523(3)
C(30)-C(31)	1.525(3)
Cl11-Al11	2.1320(10)
Cl21-Al11	2.1457(8)
Cl31-Al11	2.1284(9)
Cl41-Al11	2.1384(10)
P11-C141	1.789(2)
P11-C11	1.795(2)
P11-C201	1.802(2)
P11-C81	1.802(2)
P21-C11	1.686(2)
P21-C261	1.854(2)
C11-C21	1.492(3)
C21-C31	1.391(3)
C21-C71	1.394(3)
C31-C41	1.382(3)
C41-C51	1.381(4)
C51-C61	1.380(4)
C61-C71	1.396(3)
C81-C91	1.398(3)
C81-C131	1.399(3)
C91-C101	1.388(3)
C101-C111	1.384(4)
C111-C121	1.380(4)
C121-C131	1.392(3)
C141-C151	1.400(3)
C141-C191	1.401(3)
C151-C161	1.383(3)
C161-C171	1.370(4)
C171-C181	1.388(4)
C181-C191	1.387(3)
C201-C251	1.393(3)
C201-C211	1.400(3)
C211-C221	1.383(3)
C221-C231	1.386(3)
C231-C241	1.387(4)
C241-C251	1.385(3)
C261-C271	1.533(4)
C261-C311	1.537(3)
C271-C281	1.536(3)
C281-C291	1.521(4)
C291-C301	1.520(5)
C301-C311	1.530(4)

C(1)-P(1)-C(20)	112.38(9)
C(1)-P(1)-C(14)	109.74(9)
C(20)-P(1)-C(14)	106.56(9)
C(1)-P(1)-C(8)	108.97(9)
C(20)-P(1)-C(8)	110.51(9)
C(14)-P(1)-C(8)	108.60(10)
C(1)-P(2)-C(26)	104.58(9)
Cl(2)-Al(1)-Cl(3)	110.34(4)
Cl(2)-Al(1)-Cl(4)	108.87(4)
Cl(3)-Al(1)-Cl(4)	107.53(3)
Cl(2)-Al(1)-Cl(1)	108.89(3)
Cl(3)-Al(1)-Cl(1)	111.68(3)
Cl(4)-Al(1)-Cl(1)	109.47(4)
C(2)-C(1)-P(2)	125.79(14)
C(2)-C(1)-P(1)	118.22(14)
P(2)-C(1)-P(1)	115.93(11)
C(3)-C(2)-C(7)	118.97(18)
C(3)-C(2)-C(1)	120.23(18)
C(7)-C(2)-C(1)	120.74(18)

C(4)-C(3)-C(2)	119.99(19)
C(5)-C(4)-C(3)	120.6(2)
C(4)-C(5)-C(6)	119.7(2)
C(5)-C(6)-C(7)	120.5(2)
C(6)-C(7)-C(2)	120.23(19)
C(9)-C(8)-C(13)	120.18(19)
C(9)-C(8)-P(1)	119.90(16)
C(13)-C(8)-P(1)	119.89(16)
C(10)-C(9)-C(8)	119.2(2)
C(11)-C(10)-C(9)	120.3(2)
C(10)-C(11)-C(12)	120.8(2)
C(11)-C(12)-C(13)	119.7(2)
C(12)-C(13)-C(8)	119.8(2)
C(15)-C(14)-C(19)	120.15(19)
C(15)-C(14)-P(1)	120.22(15)
C(19)-C(14)-P(1)	119.52(16)
C(16)-C(15)-C(14)	119.75(19)
C(17)-C(16)-C(15)	120.0(2)
C(16)-C(17)-C(18)	120.6(2)
C(17)-C(18)-C(19)	120.1(2)
C(18)-C(19)-C(14)	119.3(2)
C(25)-C(20)-C(21)	119.70(19)
C(25)-C(20)-P(1)	121.06(16)
C(21)-C(20)-P(1)	119.19(15)
C(22)-C(21)-C(20)	120.0(2)
C(23)-C(22)-C(21)	120.1(2)
C(24)-C(23)-C(22)	120.2(2)
C(23)-C(24)-C(25)	120.6(2)
C(20)-C(25)-C(24)	119.5(2)
C(27)-C(26)-C(31)	110.31(16)
C(27)-C(26)-P(2)	109.75(14)
C(31)-C(26)-P(2)	108.47(14)
C(28)-C(27)-C(26)	110.83(17)
C(29)-C(28)-C(27)	111.99(19)
C(30)-C(29)-C(28)	112.36(19)
C(29)-C(30)-C(31)	111.5(2)
C(30)-C(31)-C(26)	110.16(18)
C141-P11-C11	109.02(10)
C141-P11-C201	107.10(10)
C11-P11-C201	110.59(10)
C141-P11-C81	110.25(10)
C11-P11-C81	112.03(10)
C201-P11-C81	107.73(10)
C11-P21-C261	104.30(10)
Cl31-Al11-Cl11	109.29(4)
Cl31-Al11-Cl41	108.41(4)
Cl11-Al11-Cl41	110.50(5)
Cl31-Al11-Cl21	112.56(4)
Cl11-Al11-Cl21	108.66(4)
Cl41-Al11-Cl21	107.40(4)
C21-C11-P21	126.67(15)
C21-C11-P11	116.66(14)
P21-C11-P11	116.57(12)
C31-C21-C71	119.47(19)
C31-C21-C11	119.44(19)
C71-C21-C11	121.09(19)
C41-C31-C21	120.6(2)
C51-C41-C31	120.0(2)
C61-C51-C41	120.2(2)
C51-C61-C71	120.3(2)
C21-C71-C61	119.5(2)
C91-C81-C131	120.0(2)
C91-C81-P11	119.71(17)
C131-C81-P11	120.23(17)
C101-C91-C81	119.8(2)
C111-C101-C91	119.9(2)
C121-C111-C101	120.6(2)
C111-C121-C131	120.3(2)
C121-C131-C81	119.3(2)
C151-C141-C191	119.9(2)
C151-C141-P11	120.04(17)
C191-C141-P11	120.10(17)
C161-C151-C141	119.7(2)

C171-C161-C151	120.3(2)
C161-C171-C181	120.8(2)
C191-C181-C171	120.0(2)
C181-C191-C141	119.4(2)
C251-C201-C211	119.8(2)
C251-C201-P11	119.03(16)
C211-C201-P11	120.65(16)
C221-C211-C201	119.6(2)
C211-C221-C231	120.3(2)
C221-C231-C241	120.2(2)
C251-C241-C231	119.9(2)
C241-C251-C201	120.0(2)
C271-C261-C311	111.6(2)
C271-C261-P21	109.30(16)
C311-C261-P21	108.27(17)
C261-C271-C281	109.6(2)
C291-C281-C271	111.0(2)
C301-C291-C281	112.2(2)
C291-C301-C311	111.2(3)
C301-C311-C261	110.4(2)

Table C28. Anisotropic displacement parameters ($Å^2$x 10^3) for **4b**. The anisotropic displacement factor exponent takes the form: $-2p^2[h^2 a^{*2}U^{11} + ... + 2 h k a^* b^* U^{12}]$

	U^{11}	U^{22}	U^{33}	U^{23}	U^{13}	U^{12}
Cl(1)	27(1)	21(1)	40(1)	5(1)	10(1)	1(1)
Cl(2)	33(1)	24(1)	68(1)	2(1)	-19(1)	5(1)
Cl(3)	27(1)	37(1)	30(1)	0(1)	1(1)	-11(1)
Cl(4)	38(1)	57(1)	30(1)	-13(1)	4(1)	4(1)
P(1)	17(1)	18(1)	19(1)	1(1)	2(1)	0(1)
P(2)	24(1)	32(1)	18(1)	1(1)	1(1)	-7(1)
Al(1)	21(1)	22(1)	27(1)	0(1)	0(1)	2(1)
C(1)	22(1)	21(1)	20(1)	2(1)	2(1)	0(1)
C(2)	20(1)	22(1)	15(1)	4(1)	3(1)	-2(1)
C(3)	26(1)	22(1)	23(1)	0(1)	1(1)	2(1)
C(4)	25(1)	32(1)	25(1)	2(1)	-1(1)	7(1)
C(5)	26(1)	38(1)	24(1)	0(1)	-8(1)	-2(1)
C(6)	40(1)	25(1)	27(1)	-3(1)	-7(1)	-2(1)
C(7)	29(1)	22(1)	23(1)	0(1)	-3(1)	4(1)
C(8)	18(1)	27(1)	22(1)	1(1)	1(1)	-1(1)
C(9)	28(1)	30(1)	35(1)	3(1)	-2(1)	-7(1)
C(10)	34(1)	44(1)	47(1)	0(1)	-10(1)	-15(1)
C(11)	25(1)	57(2)	36(1)	5(1)	-9(1)	-6(1)
C(12)	26(1)	41(1)	34(1)	9(1)	-3(1)	2(1)
C(13)	25(1)	28(1)	33(1)	1(1)	-2(1)	-1(1)
C(14)	21(1)	19(1)	23(1)	0(1)	3(1)	-1(1)
C(15)	25(1)	22(1)	26(1)	-2(1)	6(1)	-1(1)
C(16)	31(1)	22(1)	39(1)	-3(1)	7(1)	2(1)
C(17)	37(1)	20(1)	42(1)	6(1)	3(1)	-2(1)
C(18)	39(1)	29(1)	31(1)	6(1)	9(1)	-5(1)
C(19)	31(1)	25(1)	30(1)	0(1)	11(1)	-1(1)
C(20)	23(1)	18(1)	22(1)	-1(1)	5(1)	0(1)
C(21)	24(1)	31(1)	24(1)	2(1)	4(1)	2(1)
C(22)	31(1)	36(1)	24(1)	0(1)	2(1)	-5(1)
C(23)	42(1)	35(1)	33(1)	-11(1)	7(1)	3(1)
C(24)	36(1)	47(2)	44(1)	-15(1)	3(1)	18(1)
C(25)	28(1)	38(1)	33(1)	-8(1)	-3(1)	9(1)
C(26)	23(1)	26(1)	17(1)	4(1)	1(1)	-2(1)
C(27)	28(1)	28(1)	21(1)	1(1)	4(1)	-2(1)
C(28)	29(1)	32(1)	24(1)	4(1)	7(1)	1(1)
C(29)	39(1)	33(1)	33(1)	5(1)	14(1)	-7(1)
C(30)	41(1)	31(1)	34(1)	-3(1)	10(1)	-11(1)
C(31)	31(1)	27(1)	24(1)	0(1)	6(1)	-2(1)
Cl11	35(1)	52(1)	98(1)	38(1)	-21(1)	-8(1)
Cl21	34(1)	47(1)	48(1)	6(1)	12(1)	-14(1)
Cl31	28(1)	45(1)	34(1)	3(1)	1(1)	-2(1)
Cl41	58(1)	59(1)	42(1)	-10(1)	23(1)	-25(1)

P11	19(1)	22(1)	22(1)	0(1)	-2(1)	-3(1)
P21	32(1)	43(1)	24(1)	6(1)	-3(1)	-16(1)
Al11	26(1)	33(1)	35(1)	9(1)	2(1)	-7(1)
C11	20(1)	28(1)	22(1)	0(1)	-2(1)	-5(1)
C21	19(1)	26(1)	16(1)	-1(1)	-2(1)	-3(1)
C31	29(1)	25(1)	24(1) -2(1)	3(1)	2(1)	
C41	39(1)	26(1)	29(1)	3(1)	2(1)	-5(1)
C51	33(1)	43(1)	27(1)	7(1)	5(1)	-8(1)
C61	26(1)	45(1)	27(1)	1(1)	6(1)	5(1)
C71	26(1)	28(1)	23(1)	0(1)	-1(1)	6(1)
C81	27(1)	22(1)	26(1)	-1(1)	-6(1)	-3(1)
C91	32(1)	30(1)	25(1)	0(1)	-4(1)	-1(1)
C101	46(1)	34(1)	27(1)	2(1)	-5(1)	-4(1)
C111	49(2)	33(1)	38(1)	9(1)	-17(1)	-2(1)
C121	33(1)	34(1)	53(2)	5(1)	-14(1)	4(1)
C131	26(1)	30(1)	40(1)	2(1)	-5(1)	-1(1)
C141	19(1)	29(1)	27(1)	1(1)	-1(1)	-2(1)
C151	23(1)	29(1)	42(1)	4(1)	1(1)	-2(1)
C161	25(1)	41(1)	54(2)	12(1)	7(1)	-2(1)
C171	24(1)	63(2)	33(1)	12(1)	5(1)	3(1)
C181	28(1)	56(2)	30(1)	-8(1)	-2(1)	7(1)
C191	25(1)	37(1)	31(1)	-5(1)	-1(1)	-3(1)
C201	23(1)	24(1)	26(1)	-2(1)	-1(1)	0(1)
C211	34(1)	28(1)	29(1)	-2(1)	-5(1)	1(1)
C221	42(1)	32(1)	33(1)	-6(1)	-6(1)	1(1)
C231	43(1)	26(1)	43(1)	-6(1)	-4(1)	5(1)
C241	39(1)	30(1)	41(1)	2(1)	-5(1)	11(1)
C251	28(1)	31(1)	29(1)	-2(1)	-5(1)	4(1)
C261	28(1)	44(1)	21(1)	2(1)	-2(1)	-10(1)
C271	30(1)	47(1)	35(1)	-5(1)	2(1)	-10(1)
C281	36(1)	55(2)	46(2)	-13(1)	2(1)	-16(1)
C291	38(1)	83(2)	36(1)	-14(1)	-4(1)	-20(1)
C301	42(2)	74(2)	41(2)	9(1)	-16(1)	-9(1)
C311	48(2)	58(2)	35(1)	12(1)	-13(1)	-12(1)

3.9 Crystal structure of 5a

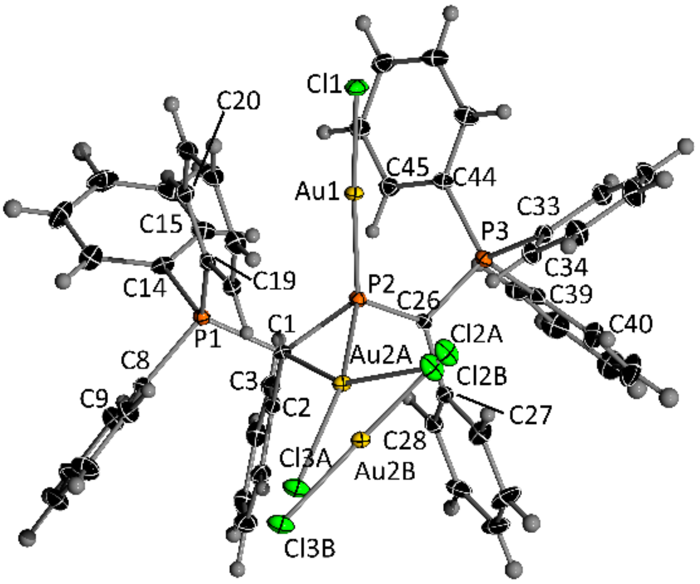

Figure S60**:** ORTEP Plot of compound **5a**. Ellipsoids are drawn at the 50% probability level.

Table C29. Crystal data and structure refinement for **5a**.

CCDC number	2121589
Empirical formula	C50 H40 Au2 Cl3 P3
Formula weight	1234.01
Temperature	100(2) K

Wavelength	0.71073 Å	
Crystal system	Monoclinic	
Space group	$P2_1/c$	
Unit cell dimensions	a = 14.4021(3) Å	α= 90°.
	b = 18.3946(3) Å	β= 111.290(2)°.
	c = 18.0279(3) Å	γ = 90°.
Volume	4450.03(15) $Å^3$	
Z	4	
Density (calculated)	1.842 Mg/m^3	
Absorption coefficient	6.909 mm^{-1}	
F(000)	2376	
Crystal size	0.180 x 0.140 x 0.090 mm^3	
Theta range for data collection	2.69 to 25.0 °.	
Index ranges	-20<=h<=20, -25<=k<=23, -22<=l<=26	
Reflections collected	71308	
Independent reflections	7814 [R(int) = 0.0565]	
Completeness to theta = 25.242°	99.9 %	
Absorption correction	Gaussian	
Max. and min. transmission	1.000 and 0.623	
Refinement method	Full-matrix least-squares on F^2	
Data / restraints / parameters	7814 / 0 / 533	
Goodness-of-fit on F^2	1.007	
Final R indices [I>2sigma(I)]	R1 = 0.0204, wR2 = 0.0482	
R indices (all data)	R1 = 0.0234, wR2 = 0.0491	
Extinction coefficient	n/a	
Largest diff. peak and hole	1.468 and -0.780 e.$Å^{-3}$	

Table C30. Atomic coordinates (x 10^4) and equivalent isotropic displacement parameters ($Å^2$x 10^3) for **5a**. U(eq) is defined as one third of the trace of the orthogonalized U^{ij} tensor.

	x	y	z	U(eq)
Au(1)	7452(1)	6217(1)	7944(1)	11(1)
Cl(1)	7764(1)	5113(1)	8567(1)	19(1)
P(1)	9319(1)	7654(1)	7823(1)	10(1)
P(2)	7087(1)	7318(1)	7413(1)	11(1)
P(3)	5084(1)	6765(1)	6426(1)	11(1)
C(1)	8039(2)	7949(2)	7414(2)	12(1)
C(10)	11089(3)	9368(2)	8912(2)	25(1)
C(2)	7880(2)	8506(2)	6759(2)	13(1)
C(11)	11217(3)	9711(2)	8274(2)	24(1)
C(12)	10794(3)	9427(2)	7514(2)	23(1)
C(13)	10233(3)	8801(2)	7394(2)	19(1)
C(14)	9645(3)	7139(2)	7100(2)	15(1)
C(15)	8931(3)	6729(2)	6515(2)	17(1)
C(16)	9210(3)	6320(2)	5983(2)	24(1)
C(17)	10187(3)	6288(2)	6046(2)	27(1)
C(18)	10911(3)	6689(2)	6629(2)	28(1)
C(19)	10640(3)	7119(2)	7149(2)	22(1)
C(20)	9612(2)	7083(2)	8697(2)	12(1)
C(21)	9995(2)	6387(2)	8683(2)	15(1)
C(22)	10248(3)	5944(2)	9351(2)	20(1)
C(23)	10123(3)	6191(2)	10038(2)	21(1)
C(24)	9742(3)	6882(2)	10056(2)	19(1)
C(25)	9498(2)	7327(2)	9394(2)	16(1)
C(26)	5926(2)	7484(2)	6722(2)	11(1)
C(27)	5488(2)	8230(2)	6522(2)	13(1)
C(28)	5335(3)	8533(2)	5775(2)	15(1)
C(29)	4838(3)	9187(2)	5551(2)	21(1)
C(30)	4480(3)	9552(2)	6063(2)	25(1)

C(31)	4633(3)	9262(2)	6805(2)	27(1)
C(32)	5141(3)	8607(2)	7039(2)	18(1)
C(33)	4555(3)	6462(2)	7143(2)	14(1)
C(34)	4978(3)	6690(2)	7929(2)	17(1)
C(35)	4588(3)	6442(2)	8483(2)	22(1)
C(36)	3789(3)	5971(2)	8253(2)	26(1)
C(37)	3362(3)	5741(2)	7472(2)	24(1)
C(38)	3737(3)	5989(2)	6912(2)	19(1)
C(39)	4030(2)	7091(2)	5586(2)	14(1)
C(40)	4020(3)	7045(2)	4813(2)	19(1)
C(41)	3245(3)	7361(2)	4186(2)	26(1)
C(42)	2503(3)	7731(2)	4343(2)	32(1)
C(43)	2512(3)	7782(2)	5106(2)	32(1)
C(44)	3269(3)	7462(2)	5732(2)	23(1)
C(45)	5619(2)	5976(2)	6135(2)	13(1)
C(46)	5457(3)	5280(2)	6351(2)	19(1)
C(47)	5850(3)	4691(2)	6082(2)	20(1)
C(48)	6374(3)	4791(2)	5585(2)	20(1)
C(49)	6534(3)	5489(2)	5359(2)	18(1)
C(50)	6169(2)	6084(2)	5643(2)	15(1)
C(3)	7654(3)	8292(2)	5972(2)	16(1)
C(4)	7579(3)	8799(2)	5385(2)	18(1)
C(5)	7735(3)	9525(2)	5574(2)	21(1)
C(6)	7958(3)	9750(2)	6352(2)	20(1)
C(7)	8024(2)	9244(2)	6945(2)	16(1)
C(8)	10107(2)	8444(2)	8031(2)	14(1)
C(9)	10536(3)	8735(2)	8799(2)	19(1)
Au(2A)	7536(1)	8234(1)	8362(1)	11(1)
Cl(3A)	8260(1)	9387(1)	9017(1)	18(1)
Cl(2A)	6641(1)	7998(1)	9209(1)	21(1)
Au(2B)	7463(5)	8822(3)	9074(4)	11(1)
Cl(3B)	8460(40)	9780(30)	9060(20)	18(1)
Cl(2B)	6480(60)	7820(40)	9250(50)	21(1)

Table C31. Bond lengths [Å] and angles [°] for **5a**.

Au(1)-P(2)	2.2196(8)
Au(1)-Cl(1)	2.2846(8)
P(1)-C(8)	1.797(3)
P(1)-C(1)	1.802(3)
P(1)-C(14)	1.807(3)
P(1)-C(20)	1.812(3)
P(2)-C(26)	1.711(3)
P(2)-C(1)	1.797(3)
P(2)-Au(2A)	2.3204(8)
P(3)-C(26)	1.742(3)
P(3)-C(45)	1.808(3)
P(3)-C(33)	1.811(3)
P(3)-C(39)	1.813(3)
C(1)-C(2)	1.517(4)
C(1)-Au(2A)	2.150(3)
C(10)-C(11)	1.382(5)
C(10)-C(9)	1.384(5)
C(2)-C(3)	1.391(5)
C(2)-C(7)	1.395(5)
C(11)-C(12)	1.384(5)
C(12)-C(13)	1.378(5)
C(13)-C(8)	1.392(5)
C(14)-C(15)	1.397(5)
C(14)-C(19)	1.403(5)
C(15)-C(16)	1.388(5)
C(16)-C(17)	1.371(6)
C(17)-C(18)	1.394(6)
C(18)-C(19)	1.385(5)
C(20)-C(21)	1.398(5)

C(20)-C(25)	1.398(5)
C(21)-C(22)	1.387(5)
C(22)-C(23)	1.391(5)
C(23)-C(24)	1.389(5)
C(24)-C(25)	1.384(5)
C(26)-C(27)	1.498(4)
C(27)-C(32)	1.394(5)
C(27)-C(28)	1.400(5)
C(28)-C(29)	1.383(5)
C(29)-C(30)	1.383(5)
C(30)-C(31)	1.380(6)
C(31)-C(32)	1.393(5)
C(33)-C(34)	1.388(5)
C(33)-C(38)	1.400(5)
C(34)-C(35)	1.390(5)
C(35)-C(36)	1.378(5)
C(36)-C(37)	1.381(5)
C(37)-C(38)	1.384(5)
C(39)-C(40)	1.391(5)
C(39)-C(44)	1.395(5)
C(40)-C(41)	1.394(5)
C(41)-C(42)	1.380(6)
C(42)-C(43)	1.373(6)
C(43)-C(44)	1.384(5)
C(45)-C(46)	1.384(5)
C(45)-C(50)	1.401(5)
C(46)-C(47)	1.388(5)
C(47)-C(48)	1.378(5)
C(48)-C(49)	1.392(5)
C(49)-C(50)	1.389(5)
C(3)-C(4)	1.385(5)
C(4)-C(5)	1.377(5)
C(5)-C(6)	1.384(5)
C(6)-C(7)	1.395(5)
C(8)-C(9)	1.401(5)
Au(2A)-Cl(2A)	2.3686(10)
Au(2A)-Cl(3A)	2.4670(9)
Au(2B)-Cl(3B)	2.27(5)
Au(2B)-Cl(2B)	2.42(7)
P(2)-Au(1)-Cl(1)	175.00(3)
C(8)-P(1)-C(1)	108.47(15)
C(8)-P(1)-C(14)	105.31(16)
C(1)-P(1)-C(14)	110.97(15)
C(8)-P(1)-C(20)	110.95(15)
C(1)-P(1)-C(20)	114.28(15)
C(14)-P(1)-C(20)	106.49(15)
C(26)-P(2)-C(1)	115.40(15)
C(26)-P(2)-Au(1)	119.52(11)
C(1)-P(2)-Au(1)	121.89(11)
C(26)-P(2)-Au(2A)	110.10(11)
C(1)-P(2)-Au(2A)	61.40(10)
Au(1)-P(2)-Au(2A)	112.68(3)
C(26)-P(3)-C(45)	112.08(16)
C(26)-P(3)-C(33)	116.22(15)
C(45)-P(3)-C(33)	107.08(15)
C(26)-P(3)-C(39)	106.49(15)
C(45)-P(3)-C(39)	109.80(15)
C(33)-P(3)-C(39)	104.84(16)
C(2)-C(1)-P(2)	122.4(2)
C(2)-C(1)-P(1)	111.4(2)
P(2)-C(1)-P(1)	118.19(17)
C(2)-C(1)-Au(2A)	117.1(2)
P(2)-C(1)-Au(2A)	71.39(11)
P(1)-C(1)-Au(2A)	109.77(15)
C(11)-C(10)-C(9)	120.3(3)
C(3)-C(2)-C(7)	118.7(3)
C(3)-C(2)-C(1)	121.1(3)
C(7)-C(2)-C(1)	120.1(3)
C(10)-C(11)-C(12)	120.6(3)
C(13)-C(12)-C(11)	119.5(3)
C(12)-C(13)-C(8)	120.6(3)
C(15)-C(14)-C(19)	119.2(3)

C(15)-C(14)-P(1)	121.0(3)
C(19)-C(14)-P(1)	119.7(3)
C(16)-C(15)-C(14)	119.7(3)
C(17)-C(16)-C(15)	120.8(4)
C(16)-C(17)-C(18)	120.2(3)
C(19)-C(18)-C(17)	119.7(4)
C(18)-C(19)-C(14)	120.3(3)
C(21)-C(20)-C(25)	119.1(3)
C(21)-C(20)-P(1)	119.0(2)
C(25)-C(20)-P(1)	121.9(2)
C(22)-C(21)-C(20)	120.2(3)
C(21)-C(22)-C(23)	120.2(3)
C(24)-C(23)-C(22)	120.0(3)
C(25)-C(24)-C(23)	119.9(3)
C(24)-C(25)-C(20)	120.6(3)
C(27)-C(26)-P(2)	123.6(2)
C(27)-C(26)-P(3)	115.8(2)
P(2)-C(26)-P(3)	118.36(18)
C(32)-C(27)-C(28)	118.4(3)
C(32)-C(27)-C(26)	121.1(3)
C(28)-C(27)-C(26)	120.2(3)
C(29)-C(28)-C(27)	120.8(3)
C(30)-C(29)-C(28)	120.4(3)
C(31)-C(30)-C(29)	119.5(3)
C(30)-C(31)-C(32)	120.7(3)
C(31)-C(32)-C(27)	120.3(3)
C(34)-C(33)-C(38)	120.1(3)
C(34)-C(33)-P(3)	119.5(3)
C(38)-C(33)-P(3)	120.4(3)
C(33)-C(34)-C(35)	119.5(3)
C(36)-C(35)-C(34)	120.1(3)
C(35)-C(36)-C(37)	120.9(3)
C(36)-C(37)-C(38)	119.7(3)
C(37)-C(38)-C(33)	119.8(3)
C(40)-C(39)-C(44)	119.7(3)
C(40)-C(39)-P(3)	121.2(3)
C(44)-C(39)-P(3)	118.7(3)
C(39)-C(40)-C(41)	120.0(3)
C(42)-C(41)-C(40)	119.4(3)
C(43)-C(42)-C(41)	120.9(3)
C(42)-C(43)-C(44)	120.4(4)
C(43)-C(44)-C(39)	119.6(4)
C(46)-C(45)-C(50)	120.0(3)
C(46)-C(45)-P(3)	122.0(3)
C(50)-C(45)-P(3)	118.0(2)
C(45)-C(46)-C(47)	119.5(3)
C(48)-C(47)-C(46)	120.8(3)
C(47)-C(48)-C(49)	120.1(3)
C(50)-C(49)-C(48)	119.6(3)
C(49)-C(50)-C(45)	119.9(3)
C(4)-C(3)-C(2)	120.9(3)
C(5)-C(4)-C(3)	120.2(3)
C(4)-C(5)-C(6)	119.9(3)
C(5)-C(6)-C(7)	120.2(3)
C(6)-C(7)-C(2)	120.2(3)
C(13)-C(8)-C(9)	119.5(3)
C(13)-C(8)-P(1)	118.1(3)
C(9)-C(8)-P(1)	122.2(3)
C(10)-C(9)-C(8)	119.4(3)
C(1)-Au(2A)-P(2)	47.21(9)
C(1)-Au(2A)-Cl(2A)	152.58(9)
P(2)-Au(2A)-Cl(2A)	105.77(3)
C(1)-Au(2A)-Cl(3A)	112.58(9)
P(2)-Au(2A)-Cl(3A)	159.00(3)
Cl(2A)-Au(2A)-Cl(3A)	94.77(3)
Cl(3B)-Au(2B)-Cl(2B)	174(2)

Table C32. Anisotropic displacement parameters ($Å^2$x 10^3) for **5a**. The anisotropic displacement factor exponent takes the form: $-2p^2[h^2 a^{*2}U^{11} + ... + 2 h k a^* b^* U^{12}]$

	U^{11}	U^{22}	U^{33}	U^{23}	U^{13}	U^{12}
Au(1)	12(1)	9(1)	12(1)	1(1)	5(1)	0(1)
Cl(1)	24(1)	12(1)	23(1)	6(1)	12(1)	3(1)
P(1)	10(1)	11(1)	10(1)	0(1)	3(1)	0(1)
P(2)	12(1)	10(1)	11(1)	1(1)	4(1)	-1(1)
P(3)	12(1)	11(1)	11(1)	1(1)	4(1)	-1(1)
C(1)	12(2)	10(2)	12(2)	-1(1)	4(1)	1(1)
C(10)	25(2)	22(2)	23(2)	-7(2)	2(2)	-7(2)
C(2)	10(2)	14(2)	15(2)	4(1)	5(1)	2(1)
C(11)	19(2)	16(2)	36(2)	-2(2)	8(2)	-6(2)
C(12)	22(2)	21(2)	27(2)	6(2)	10(2)	-4(2)
C(13)	18(2)	20(2)	19(2)	-1(1)	8(2)	-2(1)
C(14)	21(2)	13(2)	13(2)	1(1)	7(1)	4(1)
C(15)	24(2)	14(2)	16(2)	-1(1)	8(2)	0(1)
C(16)	39(2)	15(2)	21(2)	-2(1)	12(2)	-2(2)
C(17)	45(3)	19(2)	26(2)	-1(2)	23(2)	8(2)
C(18)	24(2)	33(2)	31(2)	5(2)	16(2)	13(2)
C(19)	22(2)	25(2)	17(2)	-1(2)	5(2)	3(2)
C(20)	7(2)	13(2)	13(2)	3(1)	2(1)	-1(1)
C(21)	10(2)	16(2)	17(2)	-3(1)	3(1)	-3(1)
C(22)	16(2)	14(2)	28(2)	3(2)	3(2)	0(1)
C(23)	19(2)	20(2)	21(2)	11(1)	3(2)	-1(1)
C(24)	17(2)	23(2)	17(2)	0(1)	6(2)	-3(2)
C(25)	12(2)	15(2)	19(2)	1(1)	5(1)	1(1)
C(26)	11(2)	10(2)	12(2)	-1(1)	4(1)	0(1)
C(27)	9(2)	12(2)	17(2)	-2(1)	3(1)	-1(1)
C(28)	14(2)	14(2)	16(2)	-1(1)	4(1)	-3(1)
C(29)	22(2)	13(2)	22(2)	3(1)	1(2)	-4(1)
C(30)	24(2)	12(2)	31(2)	-1(2)	-2(2)	3(2)
C(31)	28(2)	21(2)	26(2)	-10(2)	3(2)	11(2)
C(32)	17(2)	24(2)	12(2)	-1(1)	2(1)	2(2)
C(33)	16(2)	12(2)	18(2)	2(1)	9(1)	2(1)
C(34)	19(2)	17(2)	17(2)	2(1)	9(2)	-1(1)
C(35)	26(2)	27(2)	16(2)	-1(2)	9(2)	-2(2)
C(36)	27(2)	29(2)	25(2)	5(2)	15(2)	-5(2)
C(37)	19(2)	22(2)	30(2)	5(2)	10(2)	-5(2)
C(38)	17(2)	18(2)	23(2)	1(1)	7(2)	-1(1)
C(39)	13(2)	11(2)	16(2)	2(1)	2(1)	-3(1)
C(40)	20(2)	13(2)	19(2)	0(1)	3(2)	-2(1)
C(41)	28(2)	27(2)	16(2)	1(2)	0(2)	-3(2)
C(42)	23(2)	33(2)	25(2)	9(2)	-8(2)	-1(2)
C(43)	14(2)	41(2)	35(2)	7(2)	3(2)	7(2)
C(44)	18(2)	27(2)	24(2)	4(2)	8(2)	2(2)
C(45)	14(2)	13(2)	12(2)	-2(1)	4(1)	-1(1)
C(46)	23(2)	17(2)	15(2)	1(1)	5(2)	-1(1)
C(47)	25(2)	12(2)	20(2)	-1(1)	4(2)	0(1)
C(48)	21(2)	14(2)	22(2)	-5(1)	4(2)	3(1)
C(49)	16(2)	21(2)	19(2)	-6(1)	6(1)	-2(1)
C(50)	15(2)	12(2)	16(2)	-1(1)	3(1)	-1(1)
C(3)	13(2)	17(2)	16(2)	0(1)	4(1)	1(1)
C(4)	12(2)	28(2)	14(2)	5(1)	4(1)	2(1)
C(5)	18(2)	27(2)	20(2)	14(2)	9(2)	3(2)
C(6)	23(2)	14(2)	24(2)	6(1)	12(2)	3(1)
C(7)	16(2)	18(2)	17(2)	1(1)	8(1)	3(1)
C(8)	10(2)	15(2)	17(2)	-1(1)	5(1)	0(1)
C(9)	18(2)	20(2)	16(2)	-1(1)	5(2)	-2(1)
Au(2A)	13(1)	10(1)	9(1)	0(1)	5(1)	1(1)
Cl(3A)	27(1)	14(1)	13(1)	-3(1)	7(1)	-4(1)
Cl(2A)	28(1)	26(1)	16(1)	-4(1)	15(1)	-7(1)
Au(2B)	13(1)	10(1)	9(1)	0(1)	5(1)	1(1)
Cl(3B)	27(1)	14(1)	13(1)	-3(1)	7(1)	-4(1)
Cl(2B)	28(1)	26(1)	16(1)	-4(1)	15(1)	-7(1)

3.10 Crystal structure of 5b

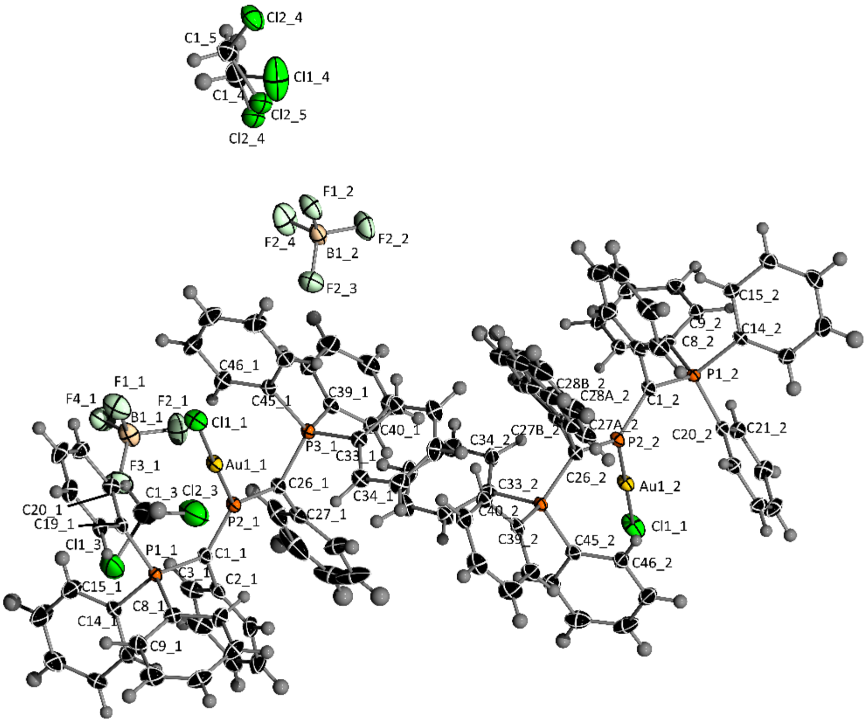

Figure S61**:** ORTEP Plot of compound **5b**. Ellipsoids are drawn at the 50% probability level.

Table C33. Crystal data and structure refinement for **5b**.

CCDC number	2121600	
Empirical formula	C51 H42 Au B Cl3 F4 P3	
Formula weight	1137.88	
Temperature	100.00(10) K	
Wavelength	1.54184 Å	
Crystal system	Triclinic	
Space group	P-1	
Unit cell dimensions	a = 9.87470(10) Å	□= 95.02°.
	b = 22.00250(10) Å	□= 97.0910(10)°.
	c = 24.04700(10) Å	□ = 94.8000(10)°.
Volume	5141.14(6) $Å^3$	
Z	4	
Density (calculated)	1.470 Mg/m^3	
Absorption coefficient	8.084 mm^{-1}	
F(000)	2256	
Crystal size	0.313 x 0.081 x 0.068 mm^3	
Theta range for data collection	2.611 to 78.025°.	
Index ranges	-12<=h<=12, -27<=k<=27, -30<=l<=29	
Reflections collected	21379	
Independent reflections	21379 [R(int) = 0.0239]	
Completeness to theta = 67.684°	99.8 %	
Absorption correction	Gaussian	
Max. and min. transmission	0.974 and 0.205	
Refinement method	Full-matrix least-squares on F^2	
Data / restraints / parameters	21379 / 90 / 1212	
Goodness-of-fit on F^2	1.056	
Final R indices [I>2sigma(I)]	R1 = 0.0281, wR2 = 0.0726	
R indices (all data)	R1 = 0.0298, wR2 = 0.0742	
Extinction coefficient	n/a	

Largest diff. peak and hole	1.189 and -1.883 e.Å^{-3}

Table C34. Atomic coordinates (x 10^4) and equivalent isotropic displacement parameters (Å^{2}x 10^3) for **5b**. U(eq) is defined as one third of the trace of the orthogonalized U^{ij} tensor.

	x	y	z	U(eq)
Au11	5739(1)	7447(1)	7301(1)	20(1)
Cl11	5718(1)	8050(1)	6577(1)	32(1)
P11	6048(1)	7938(1)	8836(1)	16(1)
P21	5743(1)	6845(1)	7991(1)	16(1)
P31	5274(1)	5747(1)	7145(1)	16(1)
B11	1035(4)	6648(2)	8223(2)	33(1)
F11	968(3)	7010(1)	7774(1)	50(1)
F21	-278(2)	6518(1)	8359(1)	47(1)
F31	1571(3)	6111(1)	8060(1)	55(1)
F41	1862(2)	6961(1)	8689(1)	46(1)
C11	5972(3)	7135(1)	8675(1)	18(1)
C21	6176(3)	6795(1)	9186(1)	21(1)
C31	7469(3)	6665(2)	9404(1)	31(1)
C41	7663(4)	6404(2)	9914(1)	42(1)
C51	6573(4)	6278(1)	10202(1)	36(1)
C61	5266(4)	6399(2)	9988(1)	35(1)
C71	5070(3)	6658(1)	9480(1)	29(1)
C81	7790(3)	8290(1)	8939(1)	20(1)
C91	8826(3)	7978(1)	8733(1)	24(1)
C101	10172(3)	8240(2)	8830(1)	30(1)
C111	10475(3)	8820(2)	9120(1)	33(1)
C121	9447(3)	9137(2)	9316(2)	35(1)
C131	8101(3)	8872(1)	9231(1)	28(1)
C141	5412(3)	8100(1)	9503(1)	19(1)
C151	4137(3)	8313(2)	9530(1)	37(1)
C161	3656(4)	8414(2)	10044(2)	53(1)
C171	4452(4)	8307(2)	10536(1)	40(1)
C181	5723(3)	8101(1)	10515(1)	26(1)
C191	6216(3)	8002(1)	10000(1)	23(1)
C201	5000(3)	8307(1)	8327(1)	17(1)
C211	3679(3)	8029(1)	8133(1)	21(1)
C221	2787(3)	8335(1)	7787(1)	26(1)
C231	3211(3)	8910(2)	7639(1)	29(1)
C241	4524(3)	9182(1)	7829(1)	28(1)
C251	5426(3)	8886(1)	8178(1)	21(1)
C261	5578(3)	6074(1)	7848(1)	18(1)
C271	5777(3)	5639(1)	8290(1)	22(1)
C281	7061(4)	5443(1)	8437(2)	37(1)
C291	7276(5)	5071(2)	8871(2)	60(1)
C301	6237(6)	4900(2)	9164(2)	65(2)
C311	4949(6)	5083(2)	9018(2)	59(1)
C321	4694(4)	5447(1)	8572(1)	37(1)
C331	6846(3)	5622(1)	6857(1)	19(1)
C341	8052(3)	5985(1)	7080(1)	23(1)
C351	9249(3)	5910(1)	6839(1)	29(1)
C361	9241(3)	5486(2)	6382(1)	31(1)
C371	8053(3)	5119(2)	6163(1)	32(1)
C381	6854(3)	5182(1)	6400(1)	25(1)
C391	4326(3)	5001(1)	7099(1)	18(1)
C401	4981(3)	4503(1)	7280(1)	24(1)
C411	4270(3)	3924(1)	7232(1)	29(1)
C421	2907(3)	3839(1)	7006(1)	31(1)
C431	2247(3)	4332(2)	6830(2)	37(1)
C441	2944(3)	4914(1)	6873(1)	29(1)
C451	4295(3)	6204(1)	6687(1)	18(1)
C461	3136(3)	6449(1)	6864(1)	23(1)
C471	2336(3)	6779(1)	6502(1)	29(1)
C481	2681(3)	6856(2)	5971(2)	34(1)
C491	3828(3)	6615(2)	5796(1)	33(1)
C501	4639(3)	6287(1)	6151(1)	25(1)
Au12	7554(1)	994(1)	7890(1)	20(1)
Cl12	7348(1)	467(1)	8660(1)	36(1)
P12	7194(1)	301(1)	6442(1)	15(1)

P22	7693(1)	1496(1)	7132(1)	16(1)
P32	8142(1)	2678(1)	7849(1)	16(1)
B12	7871(4)	8243(2)	3158(2)	34(1)
F12	7931(2)	8584(1)	2690(1)	45(1)
F22	6552(2)	8221(1)	3290(1)	60(1)
F32	8224(3)	7663(1)	3008(1)	52(1)
F42	8792(2)	8544(1)	3595(1)	55(1)
C12	7392(3)	1109(1)	6482(1)	18(1)
C22	7058(3)	1381(1)	5937(1)	21(1)
C32	5696(3)	1439(1)	5732(1)	29(1)
C42	5365(4)	1675(1)	5220(1)	36(1)
C52	6395(4)	1864(1)	4915(1)	37(1)
C62	7744(4)	1812(2)	5116(1)	38(1)
C72	8086(3)	1566(1)	5623(1)	28(1)
C82	5423(3)	26(1)	6450(1)	17(1)
C92	4901(3)	-559(1)	6200(1)	19(1)
C102	3535(3)	-759(1)	6214(1)	23(1)
C112	2678(3)	-378(1)	6459(1)	23(1)
C122	3192(3)	205(1)	6708(1)	22(1)
C132	4559(3)	405(1)	6708(1)	19(1)
C142	7673(3)	-38(1)	5792(1)	17(1)
C152	8809(3)	-371(2)	5796(1)	38(1)
C162	9129(4)	-654(2)	5300(2)	50(1)
C172	8313(4)	-625(2)	4796(1)	33(1)
C182	7172(3)	-298(1)	4788(1)	24(1)
C192	6859(3)	-1(1)	5282(1)	22(1)
C202	8254(3)	-1(1)	6997(1)	17(1)
C212	9591(3)	275(1)	7168(1)	20(1)
C222	10435(3)	20(1)	7571(1)	24(1)
C232	9961(3)	-506(2)	7797(1)	29(1)
C242	8659(3)	-785(1)	7618(1)	27(1)
C252	7788(3)	-533(1)	7218(1)	20(1)
C262	7917(3)	2270(1)	7174(1)	19(1)
C27A2	7618(16)	2656(11)	6702(9)	22(2)
C28A2	8709(16)	2933(7)	6466(6)	27(2)
C29A2	6046(18)	3179(4)	6117(5)	42(3)
C30A2	7140(20)	3459(4)	5880(4)	37(3)
C31A2	8471(19)	3335(5)	6048(5)	40(3)
C32A2	6286(14)	2772(7)	6520(6)	28(2)
C27B2	7918(17)	2658(10)	6685(9)	22(2)
C28B2	9139(16)	2863(7)	6500(7)	30(2)
C29B2	6630(20)	3200(6)	5986(5)	42(3)
C30B2	7870(20)	3398(5)	5811(5)	45(3)
C31B2	9100(20)	3235(6)	6068(5)	43(3)
C32B2	6672(16)	2825(7)	6428(6)	29(2)
C332	6510(3)	2818(1)	8076(1)	19(1)
C342	5386(3)	2384(1)	7911(1)	23(1)
C352	4128(3)	2478(1)	8091(1)	28(1)
C362	3991(3)	3003(1)	8430(1)	28(1)
C372	5102(3)	3437(1)	8590(1)	28(1)
C382	6363(3)	3348(1)	8418(1)	23(1)
C392	9051(3)	3425(1)	7851(1)	19(1)
C402	8457(3)	3871(1)	7549(1)	21(1)
C412	9118(3)	4456(1)	7582(1)	27(1)
C422	10379(3)	4601(1)	7911(1)	32(1)
C432	10982(4)	4159(2)	8204(2)	38(1)
C442	10323(3)	3571(1)	8178(1)	29(1)
C452	9091(3)	2284(1)	8374(1)	19(1)
C462	10205(3)	1975(1)	8224(1)	22(1)
C472	10921(3)	1659(1)	8620(2)	32(1)
C482	10553(4)	1656(2)	9154(2)	37(1)
C492	9500(4)	1986(2)	9311(1)	36(1)
C502	8750(3)	2296(1)	8915(1)	26(1)
Cl13	1485(1)	6326(1)	10135(1)	91(1)
C13	1175(5)	5917(2)	9463(2)	53(1)
Cl23	1322(2)	5138(1)	9519(1)	75(1)
Cl14	6411(3)	10038(1)	60(1)	52(1)
C14	5852(8)	9971(4)	721(3)	34(2)
Cl24	7112(3)	9715(1)	1198(1)	51(1)
Cl15	7331(2)	9714(1)	773(2)	82(1)
C15	5852(8)	9653(3)	1121(3)	48(2)
Cl25	6166(2)	9249(1)	1721(1)	51(1)

Table C35. Bond lengths [Å] and angles [°] for **5b**.

Au11-P21	2.2127(6)
Au11-Cl11	2.2777(7)
P11-C11	1.768(3)
P11-C201	1.798(3)
P11-C81	1.805(3)
P11-C141	1.810(3)
P21-C261	1.691(3)
P21-C11	1.692(3)
P31-C261	1.758(3)
P31-C451	1.804(3)
P31-C331	1.807(3)
P31-C391	1.808(3)
B11-F31	1.379(4)
B11-F21	1.389(4)
B11-F41	1.394(4)
B11-F11	1.394(5)
C11-C21	1.496(4)
C21-C31	1.381(4)
C21-C71	1.398(4)
C31-C41	1.398(4)
C41-C51	1.373(5)
C51-C61	1.385(5)
C61-C71	1.392(4)
C81-C91	1.395(4)
C81-C131	1.398(4)
C91-C101	1.388(4)
C101-C111	1.388(5)
C111-C121	1.385(5)
C121-C131	1.390(4)
C141-C151	1.386(4)
C141-C191	1.396(4)
C151-C161	1.384(5)
C161-C171	1.387(5)
C171-C181	1.375(5)
C181-C191	1.390(4)
C201-C251	1.398(4)
C201-C211	1.400(4)
C211-C221	1.392(4)
C221-C231	1.388(4)
C231-C241	1.387(5)
C241-C251	1.389(4)
C261-C271	1.498(4)
C271-C281	1.388(5)
C271-C321	1.391(4)
C281-C291	1.389(5)
C291-C301	1.360(8)
C301-C311	1.379(8)
C311-C321	1.405(5)
C331-C341	1.397(4)
C331-C381	1.401(4)
C341-C351	1.394(4)
C351-C361	1.377(5)
C361-C371	1.385(5)
C371-C381	1.388(4)
C391-C401	1.393(4)
C391-C441	1.395(4)
C401-C411	1.390(4)
C411-C421	1.378(5)
C421-C431	1.382(5)
C431-C441	1.391(4)
C451-C501	1.397(4)
C451-C461	1.401(4)
C461-C471	1.395(4)
C471-C481	1.383(5)
C481-C491	1.385(5)
C491-C501	1.387(4)

Au12-P22	2.2262(6)
Au12-Cl12	2.2897(7)
P12-C12	1.765(3)
P12-C202	1.802(3)
P12-C82	1.805(3)
P12-C142	1.806(3)
P22-C262	1.690(3)
P22-C12	1.692(3)
P32-C262	1.763(3)
P32-C392	1.805(3)
P32-C452	1.805(3)
P32-C332	1.806(3)
B12-F22	1.375(4)
B12-F32	1.381(4)
B12-F42	1.382(4)
B12-F12	1.411(5)
C12-C22	1.500(4)
C22-C72	1.392(4)
C22-C32	1.394(4)
C32-C42	1.392(4)
C42-C52	1.383(5)
C52-C62	1.378(5)
C62-C72	1.392(4)
C82-C92	1.399(4)
C82-C132	1.403(4)
C92-C102	1.388(4)
C102-C112	1.386(4)
C112-C122	1.394(4)
C122-C132	1.385(4)
C142-C152	1.390(4)
C142-C192	1.394(4)
C152-C162	1.380(5)
C162-C172	1.380(5)
C172-C182	1.387(4)
C182-C192	1.386(4)
C202-C252	1.395(4)
C202-C212	1.403(4)
C212-C222	1.387(4)
C222-C232	1.391(4)
C232-C242	1.379(4)
C242-C252	1.393(4)
C262-C27A2	1.494(15)
C262-C27B2	1.513(15)
C27A2-C32A2	1.385(13)
C27A2-C28A2	1.398(12)
C28A2-C31A2	1.407(12)
C29A2-C32A2	1.390(11)
C29A2-C30A2	1.408(14)
C30A2-C31A2	1.379(15)
C27B2-C28B2	1.391(13)
C27B2-C32B2	1.400(13)
C28B2-C31B2	1.377(13)
C29B2-C30B2	1.396(16)
C29B2-C32B2	1.399(13)
C30B2-C31B2	1.379(16)
C332-C382	1.396(4)
C332-C342	1.397(4)
C342-C352	1.391(4)
C352-C362	1.379(4)
C362-C372	1.386(4)
C372-C382	1.383(4)
C392-C442	1.394(4)
C392-C402	1.398(4)
C402-C412	1.385(4)
C412-C422	1.387(5)
C422-C432	1.382(5)
C432-C442	1.393(4)
C452-C502	1.384(4)
C452-C462	1.407(4)
C462-C472	1.388(4)
C472-C482	1.377(5)
C482-C492	1.387(5)
C492-C502	1.394(4)

Cl13-C13	1.759(4)
C13-Cl23	1.747(5)
Cl14-C14	1.759(8)
C14-Cl24	1.748(8)
Cl15-C15	1.772(7)
C15-Cl25	1.769(8)

P21-Au11-Cl11	178.78(2)
C11-P11-C201	113.24(12)
C11-P11-C81	111.81(13)
C201-P11-C81	110.65(12)
C11-P11-C141	109.19(12)
C201-P11-C141	106.28(12)
C81-P11-C141	105.19(12)
C261-P21-C11	117.91(13)
C261-P21-Au11	120.47(9)
C11-P21-Au11	121.58(9)
C261-P31-C451	112.87(12)
C261-P31-C331	112.19(13)
C451-P31-C331	107.94(12)
C261-P31-C391	110.32(12)
C451-P31-C391	107.24(12)
C331-P31-C391	105.91(12)
F31-B11-F21	110.0(3)
F31-B11-F41	110.3(3)
F21-B11-F41	109.3(3)
F31-B11-F11	108.6(3)
F21-B11-F11	108.9(3)
F41-B11-F11	109.8(3)
C21-C11-P21	127.98(19)
C21-C11-P11	113.18(18)
P21-C11-P11	118.81(15)
C31-C21-C71	119.3(3)
C31-C21-C11	120.8(3)
C71-C21-C11	119.7(3)
C21-C31-C41	120.0(3)
C51-C41-C31	120.3(3)
C41-C51-C61	120.5(3)
C51-C61-C71	119.4(3)
C61-C71-C21	120.5(3)
C91-C81-C131	120.1(3)
C91-C81-P11	120.0(2)
C131-C81-P11	119.8(2)
C101-C91-C81	119.9(3)
C111-C101-C91	119.8(3)
C121-C111-C101	120.6(3)
C111-C121-C131	120.0(3)
C121-C131-C81	119.6(3)
C151-C141-C191	119.1(3)
C151-C141-P11	121.6(2)
C191-C141-P11	119.3(2)
C161-C151-C141	120.4(3)
C151-C161-C171	120.1(3)
C181-C171-C161	120.0(3)
C171-C181-C191	120.1(3)
C181-C191-C141	120.2(3)
C251-C201-C211	120.8(2)
C251-C201-P11	121.2(2)
C211-C201-P11	117.68(19)
C221-C211-C201	119.2(3)
C231-C221-C211	120.0(3)
C241-C231-C221	120.7(3)
C231-C241-C251	120.2(3)
C241-C251-C201	119.2(3)
C271-C261-P21	123.47(19)
C271-C261-P31	116.51(18)
P21-C261-P31	119.87(15)
C281-C271-C321	119.5(3)
C281-C271-C261	120.0(3)
C321-C271-C261	120.4(3)
C271-C281-C291	120.4(4)
C301-C291-C281	120.6(4)
C291-C301-C311	119.7(3)

C301-C311-C321	121.0(4)
C271-C321-C311	118.7(4)
C341-C331-C381	119.8(3)
C341-C331-P31	119.5(2)
C381-C331-P31	120.7(2)
C351-C341-C331	119.5(3)
C361-C351-C341	120.3(3)
C351-C361-C371	120.6(3)
C361-C371-C381	120.0(3)
C371-C381-C331	119.8(3)
C401-C391-C441	119.3(2)
C401-C391-P31	119.9(2)
C441-C391-P31	120.7(2)
C411-C401-C391	120.3(3)
C421-C411-C401	120.2(3)
C411-C421-C431	119.8(3)
C421-C431-C441	120.8(3)
C431-C441-C391	119.6(3)
C501-C451-C461	120.1(2)
C501-C451-P31	120.7(2)
C461-C451-P31	119.1(2)
C471-C461-C451	119.5(3)
C481-C471-C461	119.9(3)
C471-C481-C491	120.6(3)
C481-C491-C501	120.3(3)
C491-C501-C451	119.5(3)
P22-Au12-Cl12	178.39(3)
C12-P12-C202	112.91(12)
C12-P12-C82	110.67(12)
C202-P12-C82	109.56(12)
C12-P12-C142	111.42(12)
C202-P12-C142	105.75(12)
C82-P12-C142	106.21(12)
C262-P22-C12	117.76(13)
C262-P22-Au12	121.99(9)
C12-P22-Au12	119.89(9)
C262-P32-C392	111.65(12)
C262-P32-C452	112.64(12)
C392-P32-C452	107.18(12)
C262-P32-C332	110.96(12)
C392-P32-C332	105.60(12)
C452-P32-C332	108.48(12)
F22-B12-F32	111.1(3)
F22-B12-F42	111.3(3)
F32-B12-F42	111.4(3)
F22-B12-F12	107.9(3)
F32-B12-F12	107.8(3)
F42-B12-F12	107.3(3)
C22-C12-P22	126.23(19)
C22-C12-P12	115.64(18)
P22-C12-P12	117.41(15)
C72-C22-C32	119.1(3)
C72-C22-C12	121.1(3)
C32-C22-C12	119.8(3)
C42-C32-C22	120.5(3)
C52-C42-C32	119.8(3)
C62-C52-C42	119.9(3)
C52-C62-C72	120.7(3)
C62-C72-C22	119.8(3)
C92-C82-C132	119.7(2)
C92-C82-P12	120.9(2)
C132-C82-P12	119.44(19)
C102-C92-C82	119.6(2)
C112-C102-C92	120.5(3)
C102-C112-C122	120.1(2)
C132-C122-C112	119.9(2)
C122-C132-C82	120.1(2)
C152-C142-C192	119.0(2)
C152-C142-P12	120.6(2)
C192-C142-P12	120.3(2)
C162-C152-C142	120.2(3)
C152-C162-C172	120.9(3)
C162-C172-C182	119.3(3)

C192-C182-C172	120.2(3)
C182-C192-C142	120.3(3)
C252-C202-C212	120.6(2)
C252-C202-P12	120.0(2)
C212-C202-P12	119.09(19)
C222-C212-C202	119.0(2)
C212-C222-C232	120.2(3)
C242-C232-C222	120.6(3)
C232-C242-C252	120.1(3)
C242-C252-C202	119.3(3)
C27A2-C262-P22	126.1(10)
C27B2-C262-P22	126.4(10)
C27A2-C262-P32	114.2(10)
C27B2-C262-P32	115.6(10)
P22-C262-P32	118.03(15)
C32A2-C27A2-C28A2	120.1(11)
C32A2-C27A2-C262	120.7(10)
C28A2-C27A2-C262	119.1(10)
C27A2-C28A2-C31A2	120.6(10)
C32A2-C29A2-C30A2	120.3(10)
C31A2-C30A2-C29A2	120.4(8)
C30A2-C31A2-C28A2	118.9(9)
C27A2-C32A2-C29A2	119.6(10)
C28B2-C27B2-C32B2	119.9(11)
C28B2-C27B2-C262	120.9(11)
C32B2-C27B2-C262	119.2(10)
C31B2-C28B2-C27B2	119.0(11)
C30B2-C29B2-C32B2	117.5(10)
C31B2-C30B2-C29B2	121.1(9)
C28B2-C31B2-C30B2	121.3(10)
C29B2-C32B2-C27B2	121.2(10)
C382-C332-C342	119.8(2)
C382-C332-P32	121.0(2)
C342-C332-P32	119.2(2)
C352-C342-C332	120.0(3)
C362-C352-C342	119.9(3)
C352-C362-C372	120.3(3)
C382-C372-C362	120.6(3)
C372-C382-C332	119.5(3)
C442-C392-C402	119.5(2)
C442-C392-P32	120.1(2)
C402-C392-P32	120.4(2)
C412-C402-C392	120.2(3)
C402-C412-C422	120.2(3)
C432-C422-C412	119.9(3)
C422-C432-C442	120.5(3)
C432-C442-C392	119.8(3)
C502-C452-C462	120.7(3)
C502-C452-P32	120.8(2)
C462-C452-P32	118.6(2)
C472-C462-C452	119.0(3)
C482-C472-C462	120.1(3)
C472-C482-C492	120.9(3)
C482-C492-C502	119.8(3)
C452-C502-C492	119.4(3)
Cl23-C13-Cl13	110.0(3)
Cl24-C14-Cl14	111.6(4)
Cl25-C15-Cl15	110.2(4)

Table C36. Anisotropic displacement parameters (Å^{2}x 10^3) for **5b**. The anisotropic displacement factor exponent takes the form: -2p^2[h^2 a*^{2}U^{11} + ... + 2 h k a* b* U^{12}]

	U^{11}	U^{22}	U^{33}	U^{23}	U^{13}	U^{12}
Au11	29(1)	14(1)	18(1)	4(1)	5(1)	0(1)
Cl11	51(1)	22(1)	25(1)	12(1)	5(1)	-1(1)
P11	18(1)	14(1)	16(1)	1(1)	2(1)	1(1)
P21	22(1)	12(1)	15(1)	3(1)	3(1)	1(1)
P31	17(1)	13(1)	16(1)	2(1)	2(1)	0(1)

B11	32(2)	24(2)	43(2)	-3(1)	13(2)	-9(1)
F11	65(2)	34(1)	49(1)	5(1)	8(1)	-11(1)
F21	31(1)	60(1)	48(1)	-4(1)	12(1)	-10(1)
F31	56(1)	34(1)	77(2)	-3(1)	23(1)	6(1)
F41	43(1)	37(1)	54(1)	1(1)	-3(1)	-12(1)
C11	22(1)	14(1)	18(1)	2(1)	3(1)	2(1)
C21	34(2)	12(1)	16(1)	0(1)	4(1)	3(1)
C31	40(2)	36(2)	22(1)	7(1)	11(1)	21(1)
C41	57(2)	48(2)	28(2)	14(1)	9(2)	36(2)
C51	67(2)	23(1)	21(1)	9(1)	10(1)	15(2)
C61	52(2)	28(2)	24(2)	7(1)	7(1)	-9(1)
C71	38(2)	23(1)	23(1)	4(1)	3(1)	-10(1)
C81	19(1)	21(1)	20(1)	5(1)	3(1)	-1(1)
C91	21(1)	24(1)	29(2)	6(1)	5(1)	4(1)
C101	20(1)	36(2)	35(2)	12(1)	5(1)	4(1)
C111	19(1)	38(2)	39(2)	14(1)	-2(1)	-7(1)
C121	33(2)	28(2)	40(2)	1(1)	-2(1)	-10(1)
C131	25(1)	23(1)	36(2)	-2(1)	4(1)	-2(1)
C141	20(1)	17(1)	19(1)	1(1)	4(1)	0(1)
C151	28(2)	63(2)	23(2)	9(1)	6(1)	18(2)
C161	31(2)	102(3)	30(2)	6(2)	10(1)	31(2)
C171	40(2)	61(2)	21(2)	0(1)	12(1)	8(2)
C181	32(2)	28(1)	18(1)	-2(1)	1(1)	0(1)
C191	28(1)	18(1)	22(1)	-2(1)	2(1)	3(1)
C201	21(1)	15(1)	17(1)	2(1)	6(1)	3(1)
C211	22(1)	21(1)	20(1)	4(1)	4(1)	1(1)
C221	21(1)	34(2)	25(1)	6(1)	4(1)	6(1)
C231	30(2)	36(2)	28(2)	17(1)	7(1)	14(1)
C241	35(2)	22(1)	31(2)	12(1)	13(1)	8(1)
C251	25(1)	17(1)	23(1)	4(1)	7(1)	2(1)
C261	22(1)	16(1)	17(1)	2(1)	3(1)	0(1)
C271	37(2)	13(1)	17(1)	2(1)	2(1)	-1(1)
C281	44(2)	20(1)	41(2)	10(1)	-17(2)	-4(1)
C291	82(3)	22(2)	61(3)	19(2)	-46(2)	-15(2)
C301	131(5)	21(2)	30(2)	13(1)	-29(2)	-12(2)
C311	130(4)	18(2)	33(2)	3(1)	35(2)	-10(2)
C321	64(2)	16(1)	34(2)	4(1)	23(2)	-1(1)
C331	21(1)	17(1)	19(1)	4(1)	4(1)	0(1)
C341	25(1)	20(1)	24(1)	4(1)	6(1)	-2(1)
C351	21(1)	30(2)	36(2)	6(1)	7(1)	-1(1)
C361	26(1)	34(2)	38(2)	7(1)	15(1)	6(1)
C371	36(2)	30(2)	30(2)	-2(1)	12(1)	6(1)
C381	26(1)	22(1)	25(1)	-2(1)	6(1)	0(1)
C391	22(1)	14(1)	19(1)	2(1)	4(1)	-2(1)
C401	25(1)	19(1)	28(1)	3(1)	-2(1)	1(1)
C411	38(2)	17(1)	31(2)	5(1)	2(1)	0(1)
C421	37(2)	21(1)	34(2)	4(1)	6(1)	-9(1)
C431	25(2)	29(2)	55(2)	5(1)	-2(1)	-8(1)
C441	23(1)	21(1)	40(2)	5(1)	-2(1)	0(1)
C451	21(1)	14(1)	20(1)	3(1)	1(1)	0(1)
C461	22(1)	21(1)	28(1)	7(1)	4(1)	1(1)
C471	20(1)	27(1)	40(2)	11(1)	-1(1)	2(1)
C481	32(2)	31(2)	39(2)	18(1)	-4(1)	4(1)
C491	40(2)	35(2)	24(2)	13(1)	1(1)	3(1)
C501	28(1)	24(1)	22(1)	5(1)	3(1)	4(1)
Au12	28(1)	16(1)	16(1)	2(1)	6(1)	0(1)
Cl12	64(1)	25(1)	23(1)	8(1)	17(1)	2(1)
P12	17(1)	13(1)	14(1)	1(1)	2(1)	1(1)
P22	21(1)	13(1)	14(1)	1(1)	3(1)	0(1)
P32	18(1)	12(1)	17(1)	0(1)	2(1)	1(1)
B12	33(2)	28(2)	38(2)	-8(1)	7(2)	1(1)
F12	60(1)	28(1)	45(1)	-5(1)	12(1)	-1(1)
F22	43(1)	61(2)	78(2)	-10(1)	31(1)	-7(1)
F32	74(2)	28(1)	58(1)	-1(1)	14(1)	12(1)
F42	51(1)	56(1)	50(1)	-14(1)	-6(1)	6(1)
C12	22(1)	15(1)	16(1)	2(1)	2(1)	1(1)
C22	34(2)	12(1)	16(1)	1(1)	2(1)	0(1)
C32	34(2)	25(1)	27(2)	5(1)	-1(1)	4(1)
C42	50(2)	24(2)	30(2)	5(1)	-10(1)	4(1)
C52	70(2)	19(1)	21(1)	4(1)	-2(2)	5(1)
C62	64(2)	26(2)	24(2)	9(1)	12(2)	0(2)
C72	37(2)	24(1)	23(1)	4(1)	7(1)	-2(1)
C82	19(1)	17(1)	15(1)	4(1)	2(1)	3(1)

C92	20(1)	17(1)	20(1)	1(1)	3(1)	2(1)
C102	20(1)	23(1)	26(1)	0(1)	2(1)	-4(1)
C112	17(1)	29(1)	23(1)	5(1)	4(1)	1(1)
C122	21(1)	27(1)	21(1)	5(1)	6(1)	8(1)
C132	21(1)	18(1)	17(1)	2(1)	2(1)	1(1)
C142	20(1)	15(1)	15(1)	-1(1)	3(1)	0(1)
C152	36(2)	60(2)	19(1)	-4(1)	-2(1)	26(2)
C162	48(2)	78(3)	28(2)	-7(2)	2(2)	41(2)
C172	43(2)	38(2)	19(1)	-4(1)	8(1)	13(1)
C182	29(1)	25(1)	18(1)	1(1)	2(1)	2(1)
C192	24(1)	22(1)	19(1)	2(1)	1(1)	5(1)
C202	19(1)	16(1)	15(1)	1(1)	3(1)	2(1)
C212	20(1)	20(1)	20(1)	2(1)	5(1)	1(1)
C222	15(1)	34(2)	22(1)	2(1)	2(1)	4(1)
C232	27(1)	40(2)	22(1)	13(1)	4(1)	14(1)
C242	30(2)	28(1)	25(1)	13(1)	8(1)	7(1)
C252	20(1)	20(1)	21(1)	4(1)	4(1)	2(1)
C262	21(1)	18(1)	17(1)	-1(1)	4(1)	2(1)
C27A2	35(5)	15(4)	16(3)	-2(3)	1(4)	-2(4)
C28A2	37(5)	24(4)	22(4)	7(3)	3(4)	2(4)
C29A2	68(6)	23(3)	30(5)	9(3)	-11(4)	-5(4)
C30A2	70(7)	15(3)	21(4)	5(3)	-11(4)	-3(4)
C31A2	68(7)	27(4)	23(4)	11(3)	-3(4)	-2(4)
C32A2	37(5)	18(3)	28(5)	5(3)	-6(4)	-3(4)
C27B2	35(5)	7(4)	20(4)	0(3)	-1(4)	-1(4)
C28B2	38(6)	22(5)	26(4)	2(3)	2(4)	-9(4)
C29B2	72(7)	25(4)	25(4)	8(3)	-10(4)	3(5)
C30B2	81(8)	25(4)	27(4)	5(3)	-2(5)	-1(5)
C31B2	70(7)	25(4)	31(4)	5(3)	0(4)	-16(4)
C32B2	39(6)	20(4)	26(5)	4(3)	-3(4)	2(5)
C332	21(1)	19(1)	17(1)	2(1)	3(1)	3(1)
C342	22(1)	18(1)	28(1)	-3(1)	4(1)	4(1)
C352	23(1)	30(2)	30(2)	1(1)	6(1)	-2(1)
C362	24(1)	31(2)	33(2)	2(1)	10(1)	6(1)
C372	33(2)	21(1)	31(2)	-2(1)	13(1)	6(1)
C382	26(1)	19(1)	24(1)	-2(1)	6(1)	-1(1)
C392	22(1)	13(1)	22(1)	-1(1)	5(1)	-1(1)
C402	24(1)	19(1)	21(1)	0(1)	2(1)	2(1)
C412	34(2)	17(1)	30(2)	4(1)	7(1)	2(1)
C422	35(2)	19(1)	41(2)	1(1)	7(1)	-8(1)
C432	34(2)	28(2)	47(2)	2(1)	-5(2)	-9(1)
C442	27(1)	20(1)	37(2)	2(1)	-3(1)	-2(1)
C452	22(1)	13(1)	19(1)	1(1)	-1(1)	0(1)
C462	20(1)	18(1)	27(1)	0(1)	0(1)	2(1)
C472	26(1)	24(1)	42(2)	2(1)	-9(1)	4(1)
C482	36(2)	33(2)	38(2)	14(1)	-13(1)	-1(1)
C492	45(2)	40(2)	23(2)	11(1)	-1(1)	0(2)
C502	30(1)	25(1)	22(1)	-1(1)	2(1)	2(1)
Cl13	61(1)	90(1)	104(1)	-58(1)	-23(1)	24(1)
C13	54(2)	50(2)	51(2)	-5(2)	-7(2)	7(2)
Cl23	112(1)	56(1)	53(1)	1(1)	-6(1)	14(1)
Cl14	76(2)	47(1)	34(1)	0(1)	14(1)	11(1)
C14	32(4)	38(4)	32(4)	2(3)	9(3)	2(3)
Cl24	53(1)	45(1)	50(1)	-6(1)	-11(1)	10(1)
Cl15	64(1)	43(1)	148(3)	6(1)	58(2)	-3(1)
C15	46(4)	42(4)	55(4)	-5(3)	13(3)	3(3)
Cl25	53(1)	45(1)	50(1)	-6(1)	-11(1)	10(1)

3.11 Crystal structure of YPPhClAuCl

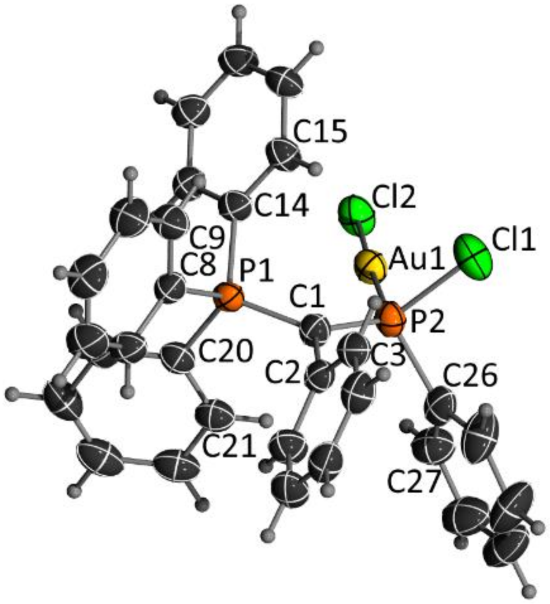

Figure S62**:** ORTEP Plot of compound **YPPhClAuCl**. Ellipsoids are drawn at the 50% probability level.

Table C37. Crystal data and structure refinement for **YPPhClAuCl**.

CCDC number	2121598	
Empirical formula	C31 H25 Au Cl2 P2	
Formula weight	727.32	
Temperature	170(2) K	
Wavelength	1.54184 Å	
Crystal system	Monoclinic	
Space group	$P2_1/n$	
Unit cell dimensions	a = 14.5305(2) Å	a= 90°.
	b = 12.19760(10) Å	b= 108.7130(10)°.
	c = 16.7287(2) Å	g = 90°.
Volume	2808.21(6) $Å^3$	
Z	4	
Density (calculated)	1.720 Mg/m^3	
Absorption coefficient	12.811 mm^{-1}	
F(000)	1416	
Crystal size	0.155 x 0.081 x 0.075 mm^3	
Theta range for data collection	3.514 to 77.110°.	
Index ranges	-16<=h<=18, -15<=k<=15, -18<=l<=20	
Reflections collected	37534	
Independent reflections	5848 [R(int) = 0.0288]	
Completeness to theta = 67.684°	99.8 %	
Absorption correction	Gaussian	
Max. and min. transmission	0.933 and 0.433	
Refinement method	Full-matrix least-squares on F^2	
Data / restraints / parameters	5848 / 0 / 325	
Goodness-of-fit on F^2	1.095	
Final R indices [I>2sigma(I)]	R1 = 0.0360, wR2 = 0.0914	
R indices (all data)	R1 = 0.0375, wR2 = 0.0924	
Extinction coefficient	n/a	
Largest diff. peak and hole	1.939 and -1.301 e.$Å^{-3}$	

Table C38. Atomic coordinates (x 10^4) and equivalent isotropic displacement parameters ($Å^2$x 10^3) for **YPPhClAuCl**. U(eq) is defined as one third of the trace of the orthogonalized U^{ij} tensor.

	x	y	z	U(eq)
Au(1)	6364(1)	2503(1)	4628(1)	39(1)
Cl(1)	7556(1)	3164(1)	3170(1)	57(1)
Cl(2)	6599(1)	1958(1)	5988(1)	48(1)
P(1)	4797(1)	1277(1)	2675(1)	32(1)
P(2)	6174(1)	3126(1)	3333(1)	37(1)
C(1)	5374(3)	2448(3)	2501(3)	36(1)
C(2)	5064(3)	2913(3)	1633(3)	36(1)
C(3)	4152(4)	3386(4)	1284(3)	46(1)
C(4)	3843(4)	3791(5)	464(3)	53(1)
C(5)	4464(4)	3752(4)	-16(3)	49(1)
C(6)	5382(4)	3299(4)	325(3)	46(1)
C(7)	5670(3)	2873(4)	1137(3)	39(1)
C(8)	5915(4)	4584(4)	3222(3)	46(1)
C(9)	5463(4)	5049(5)	3759(3)	54(1)
C(10)	5178(5)	6151(5)	3686(4)	69(2)
C(11)	5356(5)	6784(5)	3065(4)	74(2)
C(12)	5784(6)	6333(5)	2533(4)	78(2)
C(13)	6078(5)	5224(5)	2621(4)	66(2)
C(14)	4352(3)	505(3)	1702(3)	35(1)
C(15)	4946(3)	-273(4)	1496(3)	43(1)
C(16)	4625(4)	-809(4)	727(3)	54(1)
C(17)	3718(4)	-587(4)	167(3)	53(1)
C(18)	3135(4)	173(4)	364(3)	52(1)
C(19)	3438(3)	729(4)	1129(3)	42(1)
C(20)	5621(3)	390(3)	3443(2)	35(1)
C(21)	6558(3)	244(4)	3395(3)	39(1)
C(22)	7175(3)	-514(4)	3911(3)	42(1)
C(23)	6872(3)	-1124(4)	4478(3)	44(1)
C(24)	5953(3)	-971(4)	4534(3)	42(1)
C(25)	5323(3)	-226(3)	4020(3)	36(1)
C(26)	3735(3)	1543(4)	2988(3)	37(1)
C(27)	3046(3)	729(4)	2947(3)	44(1)
C(28)	2217(4)	970(5)	3148(3)	55(1)
C(29)	2065(4)	2019(6)	3394(3)	57(1)
C(30)	2750(4)	2833(5)	3451(3)	51(1)
C(31)	3585(4)	2588(4)	3245(3)	43(1)

Table C39. Bond lengths [Å] and angles [°] for **YPPhClAuCl**.

Au(1)-P(2)	2.2278(11)
Au(1)-Cl(2)	2.2870(11)
Cl(1)-P(2)	2.1112(16)
P(1)-C(1)	1.728(4)
P(1)-C(20)	1.808(4)
P(1)-C(26)	1.810(4)
P(1)-C(14)	1.810(4)
P(2)-C(1)	1.713(4)
P(2)-C(8)	1.815(5)
C(1)-C(2)	1.488(6)
C(2)-C(3)	1.391(6)
C(2)-C(7)	1.391(6)
C(3)-C(4)	1.391(7)
C(4)-C(5)	1.386(8)
C(5)-C(6)	1.386(7)
C(6)-C(7)	1.387(6)
C(8)-C(13)	1.352(8)
C(8)-C(9)	1.393(7)
C(9)-C(10)	1.400(8)
C(10)-C(11)	1.384(10)
C(11)-C(12)	1.355(10)
C(12)-C(13)	1.412(9)
C(14)-C(19)	1.392(6)
C(14)-C(15)	1.399(6)
C(15)-C(16)	1.383(6)

C(16)-C(17)	1.377(8)
C(17)-C(18)	1.365(8)
C(18)-C(19)	1.388(6)
C(20)-C(25)	1.397(6)
C(20)-C(21)	1.401(6)
C(21)-C(22)	1.381(6)
C(22)-C(23)	1.385(7)
C(23)-C(24)	1.381(7)
C(24)-C(25)	1.378(6)
C(26)-C(31)	1.385(6)
C(26)-C(27)	1.395(6)
C(27)-C(28)	1.382(7)
C(28)-C(29)	1.383(9)
C(29)-C(30)	1.388(9)
C(30)-C(31)	1.396(7)
P(2)-Au(1)-Cl(2)	176.72(4)
C(1)-P(1)-C(20)	111.6(2)
C(1)-P(1)-C(26)	113.9(2)
C(20)-P(1)-C(26)	109.5(2)
C(1)-P(1)-C(14)	109.3(2)
C(20)-P(1)-C(14)	106.95(19)
C(26)-P(1)-C(14)	105.1(2)
C(1)-P(2)-C(8)	109.0(2)
C(1)-P(2)-Cl(1)	109.33(17)
C(8)-P(2)-Cl(1)	98.05(18)
C(1)-P(2)-Au(1)	117.76(16)
C(8)-P(2)-Au(1)	113.02(16)
Cl(1)-P(2)-Au(1)	107.87(6)
C(2)-C(1)-P(2)	121.6(3)
C(2)-C(1)-P(1)	117.9(3)
P(2)-C(1)-P(1)	120.1(3)
C(3)-C(2)-C(7)	117.6(4)
C(3)-C(2)-C(1)	121.1(4)
C(7)-C(2)-C(1)	121.2(4)
C(2)-C(3)-C(4)	121.6(5)
C(5)-C(4)-C(3)	119.7(5)
C(6)-C(5)-C(4)	119.6(4)
C(5)-C(6)-C(7)	120.0(5)
C(6)-C(7)-C(2)	121.4(4)
C(13)-C(8)-C(9)	118.4(5)
C(13)-C(8)-P(2)	124.2(4)
C(9)-C(8)-P(2)	117.3(4)
C(8)-C(9)-C(10)	121.5(6)
C(11)-C(10)-C(9)	118.7(6)
C(12)-C(11)-C(10)	120.1(6)
C(11)-C(12)-C(13)	120.6(6)
C(8)-C(13)-C(12)	120.7(6)
C(19)-C(14)-C(15)	119.4(4)
C(19)-C(14)-P(1)	120.3(3)
C(15)-C(14)-P(1)	120.2(3)
C(16)-C(15)-C(14)	119.9(4)
C(17)-C(16)-C(15)	120.2(5)
C(18)-C(17)-C(16)	120.1(4)
C(17)-C(18)-C(19)	121.1(5)
C(18)-C(19)-C(14)	119.3(4)
C(25)-C(20)-C(21)	119.6(4)
C(25)-C(20)-P(1)	122.1(3)
C(21)-C(20)-P(1)	118.0(3)
C(22)-C(21)-C(20)	119.8(4)
C(21)-C(22)-C(23)	120.2(4)
C(24)-C(23)-C(22)	120.1(4)
C(25)-C(24)-C(23)	120.7(4)
C(24)-C(25)-C(20)	119.6(4)
C(31)-C(26)-C(27)	119.2(4)
C(31)-C(26)-P(1)	119.2(4)
C(27)-C(26)-P(1)	121.5(4)
C(28)-C(27)-C(26)	120.3(5)
C(27)-C(28)-C(29)	120.3(5)
C(28)-C(29)-C(30)	120.2(5)
C(29)-C(30)-C(31)	119.4(5)
C(26)-C(31)-C(30)	120.6(5)

Table C40. Anisotropic displacement parameters (Å^{2}x 10^3) for **YPPhClAuCl**. The anisotropic displacement factor exponent takes the form: -2p^2[h^2 a*^{2}U^{11} + ... + 2 h k a* b* U^{12}]

	U^{11}	U^{22}	U^{33}	U^{23}	U^{13}	U^{12}
Au(1)	37(1)	42(1)	31(1)	-2(1)	3(1)	-3(1)
Cl(1)	37(1)	79(1)	52(1)	-6(1)	12(1)	-8(1)
Cl(2)	46(1)	61(1)	34(1)	5(1)	7(1)	-7(1)
P(1)	32(1)	32(1)	27(1)	1(1)	4(1)	1(1)
P(2)	35(1)	41(1)	31(1)	-2(1)	5(1)	-5(1)
C(1)	37(2)	35(2)	32(2)	0(2)	5(2)	-3(2)
C(2)	39(2)	30(2)	32(2)	-1(2)	1(2)	-4(2)
C(3)	44(2)	49(3)	43(2)	10(2)	11(2)	4(2)
C(4)	43(3)	57(3)	48(3)	17(2)	0(2)	0(2)
C(5)	58(3)	45(3)	36(2)	5(2)	3(2)	-12(2)
C(6)	56(3)	45(2)	36(2)	-5(2)	14(2)	-12(2)
C(7)	41(2)	38(2)	35(2)	-6(2)	6(2)	-2(2)
C(8)	50(3)	39(2)	36(2)	-6(2)	-3(2)	-10(2)
C(9)	58(3)	53(3)	49(3)	0(2)	13(2)	7(2)
C(10)	77(4)	55(3)	65(4)	-14(3)	6(3)	11(3)
C(11)	99(5)	40(3)	63(4)	-8(3)	-3(3)	-2(3)
C(12)	120(6)	42(3)	71(4)	4(3)	28(4)	-18(3)
C(13)	89(4)	58(3)	59(3)	-21(3)	34(3)	-28(3)
C(14)	40(2)	30(2)	31(2)	1(2)	7(2)	-2(2)
C(15)	40(2)	47(2)	39(2)	-7(2)	8(2)	3(2)
C(16)	60(3)	51(3)	50(3)	-16(2)	16(2)	0(2)
C(17)	69(3)	52(3)	34(2)	-12(2)	9(2)	-7(2)
C(18)	57(3)	51(3)	34(2)	0(2)	-5(2)	6(2)
C(19)	44(2)	40(2)	33(2)	1(2)	0(2)	6(2)
C(20)	38(2)	35(2)	27(2)	0(2)	4(2)	2(2)
C(21)	34(2)	47(2)	32(2)	6(2)	6(2)	1(2)
C(22)	34(2)	51(3)	37(2)	0(2)	4(2)	7(2)
C(23)	47(2)	42(2)	35(2)	5(2)	0(2)	7(2)
C(24)	49(3)	41(2)	32(2)	4(2)	6(2)	3(2)
C(25)	40(2)	36(2)	31(2)	-3(2)	8(2)	-1(2)
C(26)	35(2)	43(2)	32(2)	2(2)	7(2)	3(2)
C(27)	39(2)	52(3)	39(2)	4(2)	9(2)	1(2)
C(28)	37(2)	77(4)	49(3)	10(3)	11(2)	-1(2)
C(29)	45(3)	85(4)	44(3)	14(3)	17(2)	16(3)
C(30)	53(3)	61(3)	38(2)	6(2)	14(2)	19(2)
C(31)	41(2)	47(3)	36(2)	2(2)	7(2)	7(2)

3.12 Crystal structure of YPCyClAuCl

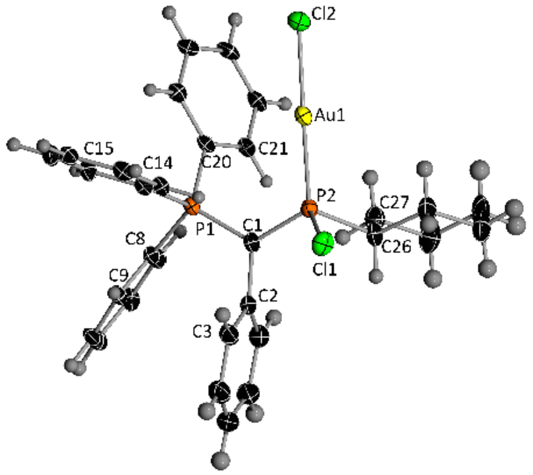

Figure C11: ORTEP Plot of compound **YPCyClAuCl**. Ellipsoids are drawn at the 50% probability level.

Table C38. Crystal data and structure refinement for **YPCyClAuCl**.

CCDC number	2121595	
Empirical formula	C31 H31 Au Cl2 P2	
Formula weight	733.36	
Temperature	99.99(13) K	
Wavelength	1.54184 Å	
Crystal system	Monoclinic	
Space group	$P2_1/c$	
Unit cell dimensions	a = 20.1097(6) Å	a= 90°.
	b = 7.83060(10) Å	b= 116.110(4)°.
	c = 20.2930(5) Å	g = 90°.
Volume	2869.45(15) $Å^3$	
Z	4	
Density (calculated)	1.698 Mg/m^3	
Absorption coefficient	12.539 mm^{-1}	
F(000)	1440	
Crystal size	0.095 x 0.039 x 0.024 mm^3	
Theta range for data collection	2.447 to 78.211°.	
Index ranges	-25<=h<=25, -8<=k<=9, -25<=l<=21	
Reflections collected	36204	
Independent reflections	6022 [R(int) = 0.0472]	
Completeness to theta = 67.684°	100.0 %	
Absorption correction	Gaussian	
Max. and min. transmission	1.000 and 0.815	
Refinement method	Full-matrix least-squares on F^2	
Data / restraints / parameters	6022 / 0 / 325	
Goodness-of-fit on F^2	1.074	
Final R indices [I>2sigma(I)]	R1 = 0.0343, wR2 = 0.0823	
R indices (all data)	R1 = 0.0379, wR2 = 0.0837	
Extinction coefficient	n/a	
Largest diff. peak and hole	2.304 and -1.623 e.$Å^{-3}$	

Table C39. Atomic coordinates (x 10^4) and equivalent isotropic displacement parameters ($Å^2$x 10^3) for **YPCyClAuCl**. U(eq) is defined as one third of the trace of the orthogonalized U^{ij} tensor.

	x	y	z	U(eq)
Cl(1)	1716(1)	9018(1)	3080(1)	29(1)
Cl(2)	3853(1)	6779(1)	5703(1)	26(1)
Au(1)	2961(1)	6512(1)	4516(1)	20(1)
P(1)	3197(1)	4988(1)	2906(1)	16(1)
P(2)	2077(1)	6446(1)	3365(1)	20(1)
C(1)	2306(2)	5674(5)	2701(2)	21(1)
C(7)	1371(2)	4476(6)	1491(2)	31(1)
C(8)	3118(2)	3854(5)	2097(2)	19(1)
C(9)	3117(2)	4819(5)	1518(2)	21(1)
C(10)	2971(2)	4032(6)	854(2)	24(1)
C(11)	2850(2)	2283(6)	772(2)	28(1)
C(12)	2860(2)	1315(5)	1350(2)	26(1)
C(13)	2984(2)	2095(5)	2008(2)	22(1)
C(14)	3895(2)	6594(5)	3056(2)	18(1)
C(15)	4561(2)	6135(5)	3034(2)	22(1)
C(16)	5096(2)	7355(5)	3145(2)	24(1)
C(17)	4972(2)	9047(5)	3275(2)	22(1)
C(18)	4319(2)	9501(5)	3300(2)	22(1)
C(19)	3776(2)	8286(5)	3189(2)	20(1)
C(20)	3566(2)	3578(5)	3690(2)	19(1)
C(21)	3102(2)	2292(5)	3735(2)	20(1)
C(22)	3369(2)	1127(5)	4302(2)	23(1)
C(23)	4102(2)	1210(5)	4834(2)	24(1)
C(24)	4561(2)	2476(5)	4797(2)	23(1)
C(25)	4303(2)	3664(5)	4229(2)	20(1)
C(26)	1180(2)	5571(6)	3238(2)	26(1)
C(27)	1258(3)	3624(5)	3351(3)	30(1)
C(28)	530(3)	2824(7)	3281(3)	41(1)
C(29)	287(3)	3625(8)	3823(4)	49(1)
C(30)	198(3)	5534(7)	3710(3)	42(1)
C(31)	908(3)	6386(6)	3764(3)	34(1)
C(2)	1790(2)	5860(6)	1902(2)	24(1)
C(3)	1767(2)	7376(6)	1527(2)	29(1)
C(4)	1355(3)	7493(7)	773(3)	39(1)
C(5)	965(3)	6105(8)	373(3)	43(1)
C(6)	971(3)	4585(8)	735(3)	40(1)

Table C40. Bond lengths [Å] and angles [°] for **YPCyClAuCl**.

Cl(1)-P(2)	2.1322(14)
Cl(2)-Au(1)	2.2936(10)
Au(1)-P(2)	2.2291(10)
P(1)-C(1)	1.736(4)
P(1)-C(20)	1.807(4)
P(1)-C(14)	1.808(4)
P(1)-C(8)	1.812(4)
P(2)-C(1)	1.714(4)
P(2)-C(26)	1.839(4)
C(1)-C(2)	1.499(5)
C(7)-C(6)	1.386(6)
C(7)-C(2)	1.401(6)
C(8)-C(9)	1.397(5)
C(8)-C(13)	1.399(5)
C(9)-C(10)	1.390(5)
C(10)-C(11)	1.388(6)
C(11)-C(12)	1.390(6)
C(12)-C(13)	1.388(6)
C(14)-C(19)	1.393(5)
C(14)-C(15)	1.406(5)
C(15)-C(16)	1.382(6)
C(16)-C(17)	1.395(6)
C(17)-C(18)	1.382(6)
C(18)-C(19)	1.389(5)
C(20)-C(21)	1.402(5)

C(20)-C(25)	1.403(6)
C(21)-C(22)	1.379(5)
C(22)-C(23)	1.394(6)
C(23)-C(24)	1.379(6)
C(24)-C(25)	1.392(6)
C(26)-C(31)	1.535(6)
C(26)-C(27)	1.539(6)
C(27)-C(28)	1.540(6)
C(28)-C(29)	1.522(8)
C(29)-C(30)	1.511(8)
C(30)-C(31)	1.536(6)
C(2)-C(3)	1.400(6)
C(3)-C(4)	1.386(6)
C(4)-C(5)	1.376(8)
C(5)-C(6)	1.397(8)

P(2)-Au(1)-Cl(2)	175.99(4)
C(1)-P(1)-C(20)	112.41(18)
C(1)-P(1)-C(14)	117.86(18)
C(20)-P(1)-C(14)	106.82(18)
C(1)-P(1)-C(8)	105.36(18)
C(20)-P(1)-C(8)	109.69(17)
C(14)-P(1)-C(8)	104.23(17)
C(1)-P(2)-C(26)	110.24(19)
C(1)-P(2)-Cl(1)	107.31(14)
C(26)-P(2)-Cl(1)	96.81(15)
C(1)-P(2)-Au(1)	117.73(14)
C(26)-P(2)-Au(1)	115.82(14)
Cl(1)-P(2)-Au(1)	106.33(5)
C(2)-C(1)-P(2)	121.2(3)
C(2)-C(1)-P(1)	116.1(3)
P(2)-C(1)-P(1)	121.8(2)
C(6)-C(7)-C(2)	121.0(5)
C(9)-C(8)-C(13)	119.7(4)
C(9)-C(8)-P(1)	117.7(3)
C(13)-C(8)-P(1)	122.3(3)
C(10)-C(9)-C(8)	119.8(4)
C(11)-C(10)-C(9)	120.4(4)
C(10)-C(11)-C(12)	119.9(4)
C(13)-C(12)-C(11)	120.2(4)
C(12)-C(13)-C(8)	120.0(4)
C(19)-C(14)-C(15)	119.7(4)
C(19)-C(14)-P(1)	120.6(3)
C(15)-C(14)-P(1)	119.7(3)
C(16)-C(15)-C(14)	120.2(4)
C(15)-C(16)-C(17)	119.7(4)
C(18)-C(17)-C(16)	120.2(4)
C(17)-C(18)-C(19)	120.6(4)
C(18)-C(19)-C(14)	119.6(4)
C(21)-C(20)-C(25)	119.3(3)
C(21)-C(20)-P(1)	117.9(3)
C(25)-C(20)-P(1)	122.7(3)
C(22)-C(21)-C(20)	120.2(4)
C(21)-C(22)-C(23)	120.3(4)
C(24)-C(23)-C(22)	119.8(4)
C(23)-C(24)-C(25)	120.7(4)
C(24)-C(25)-C(20)	119.6(4)
C(31)-C(26)-C(27)	110.7(4)
C(31)-C(26)-P(2)	112.0(3)
C(27)-C(26)-P(2)	108.6(3)
C(26)-C(27)-C(28)	111.0(4)
C(29)-C(28)-C(27)	111.1(4)
C(30)-C(29)-C(28)	110.6(5)
C(29)-C(30)-C(31)	112.0(4)
C(26)-C(31)-C(30)	111.9(4)
C(3)-C(2)-C(7)	117.8(4)
C(3)-C(2)-C(1)	120.7(4)
C(7)-C(2)-C(1)	121.2(4)
C(4)-C(3)-C(2)	121.1(5)
C(5)-C(4)-C(3)	120.5(5)
C(4)-C(5)-C(6)	119.5(4)
C(7)-C(6)-C(5)	120.1(5)

Table C41. Anisotropic displacement parameters ($Å^2$x 10^3) for **YPCyClAuCl**. The anisotropic displacement factor exponent takes the form: $-2p^2$[h^2 $a^{*2}U^{11}$ + ... + 2 h k a* b* U^{12}]

	U^{11}	U^{22}	U^{33}	U^{23}	U^{13}	U^{12}
Cl(1)	30(1)	22(1)	36(1)	0(1)	16(1)	3(1)
Cl(2)	30(1)	27(1)	22(1)	-1(1)	10(1)	0(1)
Au(1)	24(1)	19(1)	20(1)	-1(1)	13(1)	-1(1)
P(1)	20(1)	15(1)	16(1)	0(1)	10(1)	0(1)
P(2)	20(1)	21(1)	20(1)	-2(1)	11(1)	0(1)
C(1)	22(2)	20(2)	22(2)	-1(2)	12(2)	0(2)
C(7)	27(2)	40(3)	30(2)	1(2)	15(2)	-2(2)
C(8)	19(2)	20(2)	17(2)	-1(1)	9(1)	1(1)
C(9)	24(2)	22(2)	20(2)	1(2)	14(2)	2(2)
C(10)	31(2)	31(2)	18(2)	2(2)	15(2)	1(2)
C(11)	33(2)	31(2)	23(2)	-7(2)	16(2)	-2(2)
C(12)	30(2)	23(2)	30(2)	-5(2)	16(2)	-2(2)
C(13)	26(2)	21(2)	23(2)	-2(2)	14(2)	-1(2)
C(14)	23(2)	17(2)	17(2)	0(1)	11(2)	0(1)
C(15)	25(2)	22(2)	23(2)	-3(2)	15(2)	-1(2)
C(16)	23(2)	27(2)	25(2)	-2(2)	15(2)	-1(2)
C(17)	26(2)	23(2)	19(2)	-1(2)	11(2)	-5(2)
C(18)	29(2)	18(2)	22(2)	0(2)	12(2)	-1(2)
C(19)	25(2)	18(2)	20(2)	1(1)	14(2)	0(1)
C(20)	25(2)	17(2)	16(2)	-2(1)	11(2)	0(1)
C(21)	24(2)	18(2)	20(2)	0(1)	12(2)	0(1)
C(22)	33(2)	19(2)	21(2)	0(2)	17(2)	-3(2)
C(23)	38(2)	21(2)	15(2)	4(2)	14(2)	6(2)
C(24)	28(2)	25(2)	16(2)	-2(2)	8(2)	-1(2)
C(25)	25(2)	16(2)	21(2)	-2(1)	12(2)	-1(1)
C(26)	28(2)	28(2)	31(2)	-2(2)	20(2)	0(2)
C(27)	29(2)	27(2)	36(2)	1(2)	18(2)	-4(2)
C(28)	35(3)	35(3)	61(3)	-2(2)	28(2)	-7(2)
C(29)	41(3)	61(4)	61(4)	3(3)	38(3)	-12(2)
C(30)	36(3)	50(3)	54(3)	-7(3)	32(2)	-1(2)
C(31)	31(2)	40(3)	39(3)	-9(2)	23(2)	-4(2)
C(2)	21(2)	32(2)	21(2)	1(2)	12(2)	4(2)
C(3)	30(2)	36(2)	26(2)	8(2)	16(2)	7(2)
C(4)	37(3)	52(3)	32(2)	14(2)	19(2)	13(2)
C(5)	29(2)	74(4)	24(2)	5(2)	11(2)	5(2)
C(6)	28(2)	62(3)	29(2)	-6(2)	11(2)	-8(2)

3.13 Crystal structure of [YPPhClAu(tht)]$AlCl_4$

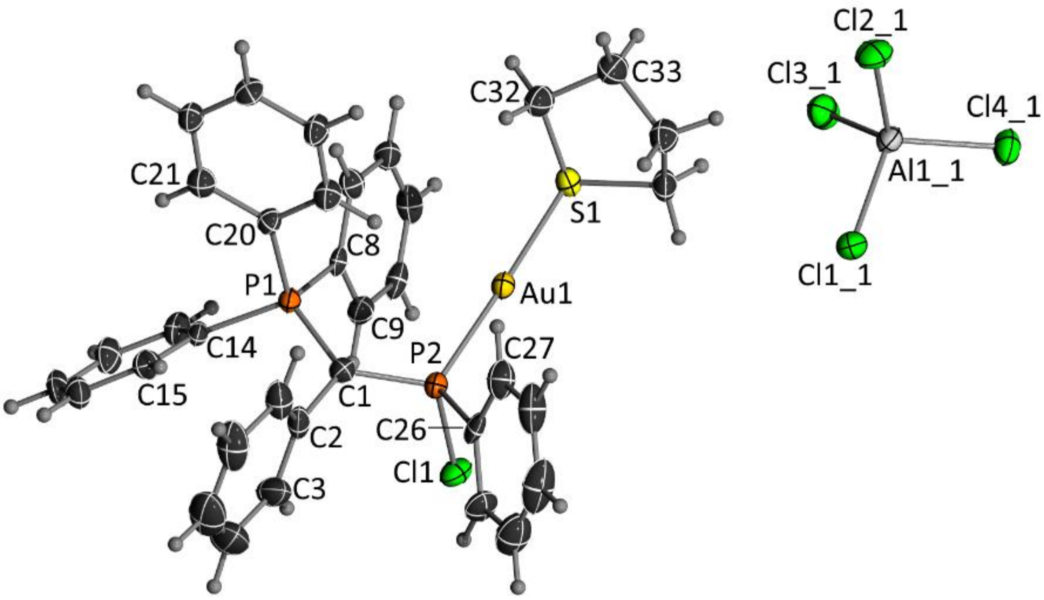

Figure S63**:** ORTEP Plot of compound **YPPhClAu(tht)AlCl$_4$**. Ellipsoids are drawn at the 50% probability level.

Table C41. Crystal data and structure refinement for **YPPhClAu(tht)AlCl$_4$**.

CCDC number	2121590	
Empirical formula	C35 H33 Al Au Cl5 P2 S	
Formula weight	948.81	
Temperature	100(2) K	
Wavelength	1.54184 Å	
Crystal system	Orthorhombic	
Space group	$P2_12_12_1$	
Unit cell dimensions	a = 9.66550(10) Å	a= 90°.
	b = 13.3221(2) Å	b= 90°.
	c = 29.0521(4) Å	g = 90°.
Volume	3740.89(9) Å^3	
Z	4	
Density (calculated)	1.685 Mg/m^3	
Absorption coefficient	12.427 mm^{-1}	
F(000)	1864	
Crystal size	0.226 x 0.121 x 0.031 mm^3	
Theta range for data collection	3.042 to 76.923°.	
Index ranges	-11<=h<=12, -16<=k<=8, -36<=l<=35	
Reflections collected	25045	
Independent reflections	7565 [R(int) = 0.0397]	
Completeness to theta = 67.684°	99.9 %	
Absorption correction	Gaussian	
Max. and min. transmission	1.000 and 0.135	
Refinement method	Full-matrix least-squares on F^2	
Data / restraints / parameters	7565 / 0 / 406	
Goodness-of-fit on F^2	1.047	
Final R indices [I>2sigma(I)]	R1 = 0.0306, wR2 = 0.0821	
R indices (all data)	R1 = 0.0310, wR2 = 0.0822	
Absolute structure parameter	-0.028(5)	
Extinction coefficient	n/a	
Largest diff. peak and hole	0.926 and -2.367 e.Å^{-3}	

Table C42. Atomic coordinates (x 10^4) and equivalent isotropic displacement parameters ($Å^2$x 10^3) for **YPPhClAu(tht)AlCl₄**. U(eq) is defined as one third of the trace of the orthogonalized U^{ij} tensor.

	x	y	z	U(eq)
Au(1)	6464(1)	5823(1)	5676(1)	22(1)
Cl(1)	5605(2)	7761(1)	6422(1)	31(1)
S(1)	6275(2)	5272(1)	4922(1)	25(1)
P(1)	9790(2)	6671(1)	6249(1)	19(1)
P(2)	6713(2)	6396(1)	6404(1)	21(1)
C(1)	8360(7)	6513(5)	6602(2)	24(1)
C(2)	8644(7)	6559(6)	7108(2)	30(1)
C(3)	8437(9)	7443(7)	7351(3)	44(2)
C(4)	8780(11)	7492(10)	7817(3)	59(3)
C(5)	9375(11)	6676(10)	8036(3)	64(3)
C(6)	9564(10)	5800(9)	7799(3)	52(2)
C(7)	9190(8)	5724(7)	7337(2)	37(2)
C(26)	5757(6)	5677(5)	6827(2)	26(1)
C(8)	9293(6)	7011(4)	5669(2)	20(1)
C(27)	5780(8)	4637(6)	6788(3)	34(2)
C(9)	8481(7)	7873(5)	5601(2)	25(1)
C(28)	5058(9)	4049(7)	7095(3)	46(2)
C(10)	8131(7)	8150(5)	5159(3)	28(1)
C(29)	4302(10)	4493(9)	7444(3)	55(3)
C(11)	8568(8)	7600(5)	4784(2)	32(1)
C(30)	4265(10)	5516(9)	7486(3)	52(2)
C(12)	9378(7)	6751(6)	4849(2)	28(1)
C(31)	4991(8)	6124(7)	7180(3)	36(2)
C(13)	9746(7)	6453(5)	5290(2)	25(1)
C(14)	10970(6)	7621(5)	6462(2)	22(1)
C(15)	11549(8)	7530(5)	6901(2)	29(1)
C(16)	12413(8)	8279(5)	7067(3)	31(2)
C(17)	12729(7)	9105(6)	6796(3)	34(2)
C(18)	12172(7)	9183(6)	6358(3)	34(2)
C(19)	11301(8)	8455(5)	6192(2)	29(1)
C(20)	10828(7)	5541(5)	6198(2)	21(1)
C(21)	12251(8)	5583(5)	6163(2)	27(1)
C(22)	12997(7)	4706(5)	6081(2)	26(1)
C(23)	12314(8)	3795(5)	6030(2)	27(1)
C(24)	10889(7)	3761(5)	6062(3)	29(1)
C(25)	10132(8)	4626(5)	6147(2)	28(1)
C(32)	7468(8)	4193(6)	4872(3)	33(1)
C(33)	6572(9)	3334(6)	4701(3)	36(2)
C(34)	5180(8)	3429(5)	4943(3)	33(2)
C(35)	4714(7)	4501(5)	4880(3)	30(1)
Cl11	1172(2)	4205(2)	4720(1)	33(1)
Cl21	2476(2)	2308(1)	4020(1)	39(1)
Cl31	2705(2)	4802(1)	3670(1)	39(1)
Cl41	-526(2)	3570(1)	3702(1)	36(1)
Al11	1449(2)	3734(1)	4020(1)	26(1)

Table C43. Bond lengths [Å] and angles [°] for **YPPhClAu(tht)AlCl₄**.

Au(1)-P(2)	2.2601(16)
Au(1)-S(1)	2.3182(15)
Cl(1)-P(2)	2.111(2)
S(1)-C(35)	1.829(7)
S(1)-C(32)	1.849(8)
P(1)-C(1)	1.734(7)
P(1)-C(8)	1.809(7)
P(1)-C(14)	1.812(6)
P(1)-C(20)	1.816(6)
P(2)-C(1)	1.700(7)
P(2)-C(26)	1.813(7)
C(1)-C(2)	1.497(8)
C(2)-C(3)	1.387(11)
C(2)-C(7)	1.400(12)
C(3)-C(4)	1.396(12)
C(4)-C(5)	1.386(17)
C(5)-C(6)	1.368(17)

C(6)-C(7)	1.394(10)
C(26)-C(27)	1.390(11)
C(26)-C(31)	1.398(10)
C(8)-C(13)	1.401(9)
C(8)-C(9)	1.405(9)
C(27)-C(28)	1.377(11)
C(9)-C(10)	1.380(10)
C(28)-C(29)	1.383(15)
C(10)-C(11)	1.379(11)
C(29)-C(30)	1.368(16)
C(11)-C(12)	1.388(11)
C(30)-C(31)	1.392(12)
C(12)-C(13)	1.388(9)
C(14)-C(15)	1.397(9)
C(14)-C(19)	1.397(10)
C(15)-C(16)	1.388(10)
C(16)-C(17)	1.387(11)
C(17)-C(18)	1.386(11)
C(18)-C(19)	1.372(10)
C(20)-C(21)	1.380(9)
C(20)-C(25)	1.399(9)
C(21)-C(22)	1.393(10)
C(22)-C(23)	1.390(10)
C(23)-C(24)	1.382(10)
C(24)-C(25)	1.388(10)
C(32)-C(33)	1.517(11)
C(33)-C(34)	1.522(11)
C(34)-C(35)	1.509(10)
Cl11-Al11	2.145(2)
Cl21-Al11	2.144(3)
Cl31-Al11	2.130(3)
Cl41-Al11	2.132(3)
P(2)-Au(1)-S(1)	177.90(6)
C(35)-S(1)-C(32)	94.1(3)
C(35)-S(1)-Au(1)	107.8(2)
C(32)-S(1)-Au(1)	105.8(2)
C(1)-P(1)-C(8)	111.6(3)
C(1)-P(1)-C(14)	112.6(3)
C(8)-P(1)-C(14)	108.1(3)
C(1)-P(1)-C(20)	112.8(3)
C(8)-P(1)-C(20)	106.2(3)
C(14)-P(1)-C(20)	105.0(3)
C(1)-P(2)-C(26)	107.2(3)
C(1)-P(2)-Cl(1)	112.8(2)
C(26)-P(2)-Cl(1)	100.3(2)
C(1)-P(2)-Au(1)	116.6(2)
C(26)-P(2)-Au(1)	113.7(2)
Cl(1)-P(2)-Au(1)	105.09(8)
C(2)-C(1)-P(2)	120.5(5)
C(2)-C(1)-P(1)	115.4(5)
P(2)-C(1)-P(1)	123.9(4)
C(3)-C(2)-C(7)	119.1(7)
C(3)-C(2)-C(1)	120.6(7)
C(7)-C(2)-C(1)	120.2(7)
C(2)-C(3)-C(4)	120.0(9)
C(5)-C(4)-C(3)	120.5(10)
C(6)-C(5)-C(4)	119.5(8)
C(5)-C(6)-C(7)	120.9(10)
C(6)-C(7)-C(2)	119.8(9)
C(27)-C(26)-C(31)	119.6(7)
C(27)-C(26)-P(2)	117.6(6)
C(31)-C(26)-P(2)	122.9(6)
C(13)-C(8)-C(9)	119.8(6)
C(13)-C(8)-P(1)	121.2(5)
C(9)-C(8)-P(1)	119.0(5)
C(28)-C(27)-C(26)	120.4(8)
C(10)-C(9)-C(8)	119.2(6)
C(27)-C(28)-C(29)	119.9(9)
C(11)-C(10)-C(9)	121.2(6)
C(30)-C(29)-C(28)	120.5(8)
C(10)-C(11)-C(12)	119.9(6)
C(29)-C(30)-C(31)	120.6(9)

C(13)-C(12)-C(11)	120.2(6)
C(30)-C(31)-C(26)	119.1(8)
C(12)-C(13)-C(8)	119.7(6)
C(15)-C(14)-C(19)	119.3(6)
C(15)-C(14)-P(1)	120.2(5)
C(19)-C(14)-P(1)	120.5(5)
C(16)-C(15)-C(14)	119.8(6)
C(17)-C(16)-C(15)	120.3(7)
C(18)-C(17)-C(16)	119.7(7)
C(19)-C(18)-C(17)	120.5(7)
C(18)-C(19)-C(14)	120.4(7)
C(21)-C(20)-C(25)	120.5(6)
C(21)-C(20)-P(1)	121.5(5)
C(25)-C(20)-P(1)	117.7(5)
C(20)-C(21)-C(22)	119.6(7)
C(23)-C(22)-C(21)	120.3(6)
C(24)-C(23)-C(22)	119.7(7)
C(23)-C(24)-C(25)	120.7(7)
C(24)-C(25)-C(20)	119.3(6)
C(33)-C(32)-S(1)	104.9(5)
C(32)-C(33)-C(34)	107.0(6)
C(35)-C(34)-C(33)	106.6(6)
C(34)-C(35)-S(1)	106.1(5)
Cl31-Al11-Cl41	111.78(12)
Cl31-Al11-Cl21	109.16(12)
Cl41-Al11-Cl21	108.89(11)
Cl31-Al11-Cl11	109.20(11)
Cl41-Al11-Cl11	109.28(12)
Cl21-Al11-Cl11	108.48(11)

Table C44. Anisotropic displacement parameters ($Å^2 x 10^3$) for **YPPhClAu(tht)AlCl₄**. The anisotropic displacement factor exponent takes the form: $-2p^2[h^2 a^{*2}U^{11} + ... + 2 h k a^* b^* U^{12}]$

	U^{11}	U^{22}	U^{33}	U^{23}	U^{13}	U^{12}
Au(1)	17(1)	25(1)	24(1)	-1(1)	-1(1)	-3(1)
Cl(1)	26(1)	30(1)	37(1)	-1(1)	4(1)	7(1)
S(1)	26(1)	26(1)	24(1)	-2(1)	-3(1)	-4(1)
P(1)	15(1)	19(1)	24(1)	-2(1)	-1(1)	-1(1)
P(2)	17(1)	24(1)	23(1)	-2(1)	0(1)	-2(1)
C(1)	21(3)	29(3)	24(3)	-3(2)	1(2)	0(3)
C(2)	20(3)	48(4)	21(3)	-1(3)	-1(3)	-11(3)
C(3)	37(4)	61(5)	34(4)	-16(4)	-4(3)	14(4)
C(4)	55(6)	83(7)	39(4)	-23(5)	-2(4)	5(6)
C(5)	53(6)	108(9)	29(4)	-4(5)	-3(4)	-15(6)
C(6)	46(5)	76(6)	32(4)	20(4)	-12(3)	-20(5)
C(7)	32(4)	48(4)	30(3)	5(3)	-6(3)	-12(4)
C(26)	10(3)	35(4)	31(3)	4(3)	-3(2)	2(3)
C(8)	12(2)	23(3)	26(3)	2(2)	-2(2)	-5(2)
C(27)	29(4)	35(4)	39(4)	9(3)	-9(3)	-3(3)
C(9)	21(3)	23(3)	32(3)	0(2)	1(3)	-1(3)
C(28)	38(4)	46(5)	54(5)	22(4)	-19(4)	-16(4)
C(10)	15(3)	26(3)	43(4)	12(3)	-6(3)	-3(2)
C(29)	37(5)	84(8)	46(5)	37(5)	-6(4)	-20(5)
C(11)	31(3)	37(4)	28(3)	10(3)	-7(3)	-10(3)
C(30)	36(5)	82(8)	36(4)	9(4)	5(4)	-6(4)
C(12)	23(3)	38(4)	25(3)	-3(3)	-1(3)	-1(3)
C(31)	27(4)	53(5)	28(3)	1(3)	11(3)	-5(3)
C(13)	25(3)	24(3)	26(3)	1(2)	-1(3)	-4(3)
C(14)	13(3)	20(3)	31(3)	-7(2)	-2(2)	0(2)
C(15)	28(3)	28(3)	30(3)	0(3)	1(3)	-1(3)
C(16)	26(3)	31(3)	36(4)	-5(3)	-8(3)	0(3)
C(17)	28(4)	26(3)	49(4)	-6(3)	-10(3)	-9(3)
C(18)	28(3)	25(3)	50(4)	0(3)	-5(3)	-7(3)
C(19)	25(3)	29(3)	34(3)	3(3)	-2(3)	-3(3)
C(20)	18(3)	22(3)	21(3)	1(2)	-2(2)	2(2)
C(21)	28(3)	29(4)	25(3)	2(2)	-1(2)	-1(3)
C(22)	22(3)	25(3)	32(3)	0(3)	-1(3)	-3(3)

C(23)	25(3)	21(3)	33(3)	0(3)	-4(3)	9(3)
C(24)	22(3)	23(3)	41(4)	1(3)	-2(3)	-3(3)
C(25)	25(3)	23(3)	35(4)	3(3)	-3(3)	0(3)
C(32)	28(3)	32(3)	39(4)	-3(3)	4(3)	-3(3)
C(33)	32(4)	33(4)	42(4)	-5(3)	2(3)	-1(3)
C(34)	28(4)	27(3)	43(4)	0(3)	-4(3)	-1(3)
C(35)	22(3)	32(4)	36(4)	-3(3)	-6(3)	-3(3)
Cl11	27(1)	36(1)	35(1)	-8(1)	4(1)	1(1)
Cl21	38(1)	33(1)	46(1)	-4(1)	1(1)	13(1)
Cl31	40(1)	39(1)	40(1)	-3(1)	6(1)	-11(1)
Cl41	25(1)	34(1)	48(1)	-1(1)	-9(1)	1(1)
Al11	19(1)	26(1)	32(1)	-2(1)	1(1)	2(1)

3.14 Crystal structure of [YPCyRh(cod)Cl]AlCl$_4$

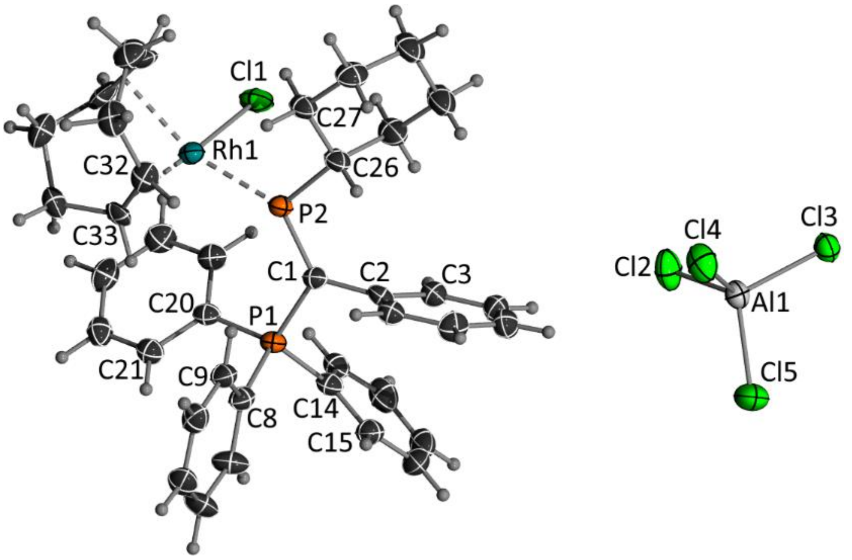

Figure S64**:** ORTEP Plot of compound **YPCyRh(COD)ClAlCl$_4$**. Ellipsoids are drawn at the 50% probability level.

Table C45. Crystal data and structure refinement for **YPCyRh(COD)ClAlCl$_4$**.

CCDC number	2121591	
Empirical formula	C39 H43 Al Cl5 P2 Rh	
Formula weight	880.81	
Temperature	100(2) K	
Wavelength	1.54184 Å	
Crystal system	Monoclinic	
Space group	P2$_1$	
Unit cell dimensions	a = 9.38480(10) Å	a= 90°.
	b = 17.5324(2) Å	b= 107.9000(10)°.
	c = 12.8617(2) Å	g = 90°.
Volume	2013.80(5) Å^3	
Z	2	
Density (calculated)	1.453 Mg/m^3	
Absorption coefficient	7.649 mm^{-1}	

F(000)	900
Crystal size	0.384 x 0.096 x 0.035 mm^3
Theta range for data collection	3.611 to 77.843°.
Index ranges	-11<=h<=11, -22<=k<=21, -13<=l<=16
Reflections collected	25287
Independent reflections	7199 [R(int) = 0.0521]
Completeness to theta = 67.684°	100.0 %
Absorption correction	Gaussian
Max. and min. transmission	1.000 and 0.207
Refinement method	Full-matrix least-squares on F^2
Data / restraints / parameters	7199 / 1 / 433
Goodness-of-fit on F^2	1.017
Final R indices [I>2sigma(I)]	R1 = 0.0399, wR2 = 0.1053
R indices (all data)	R1 = 0.0420, wR2 = 0.1066
Absolute structure parameter	-0.031(6)
Extinction coefficient	n/a
Largest diff. peak and hole	1.066 and -0.870 e.$Å^{-3}$

Table C46. Atomic coordinates (x 10^4) and equivalent isotropic displacement parameters ($Å^2$x 10^3) for **YPCyRh(COD)ClAlCl₄**. U(eq) is defined as one third of the trace of the orthogonalized U^{ij} tensor.

	x	y	z	U(eq)
Rh(1)	3704(1)	3813(1)	8761(1)	26(1)
Cl(1)	6039(2)	3219(1)	9633(1)	39(1)
Al(1)	11381(2)	4689(1)	3100(1)	30(1)
C(1)	5599(6)	5102(3)	7410(4)	24(1)
P(1)	5539(1)	5949(1)	8181(1)	24(1)
P(2)	4764(2)	4288(1)	7581(1)	25(1)
Cl(2)	11360(2)	4571(1)	4752(1)	50(1)
C(2)	6348(6)	5205(3)	6550(4)	25(1)
C(4)	8608(7)	5238(4)	6019(5)	33(1)
Cl(4)	9353(2)	4252(1)	2028(2)	50(1)
C(3)	7907(6)	5173(4)	6822(4)	31(1)
Cl(3)	13265(2)	4063(1)	2941(1)	36(1)
C(5)	7767(8)	5324(4)	4937(5)	38(1)
Cl(5)	11631(2)	5865(1)	2769(2)	56(1)
C(6)	6223(7)	5368(4)	4664(4)	36(1)
C(7)	5511(7)	5308(4)	5454(5)	29(1)
C(8)	4083(6)	6566(4)	7369(4)	28(1)
C(9)	2814(6)	6249(4)	6613(5)	32(1)
C(10)	1652(7)	6723(4)	6050(5)	36(1)
C(11)	1710(7)	7495(4)	6249(5)	39(1)
C(12)	2969(8)	7816(4)	6992(6)	43(2)
C(13)	4155(8)	7347(4)	7547(5)	38(1)
C(15)	7586(7)	6913(4)	7691(5)	33(1)
C(14)	7303(7)	6435(3)	8469(4)	29(1)
C(16)	8992(8)	7255(4)	7906(6)	41(2)
C(17)	10106(7)	7114(4)	8881(6)	44(2)
C(18)	9836(7)	6629(5)	9648(6)	42(2)
C(19)	8433(7)	6295(4)	9455(5)	32(1)
C(20)	5170(6)	5762(3)	9445(4)	26(1)
C(21)	4254(7)	6263(4)	9795(5)	31(1)
C(22)	4030(7)	6145(4)	10800(5)	34(1)
C(23)	4669(7)	5522(5)	11429(5)	38(2)
C(24)	5564(7)	5026(4)	11089(5)	39(1)
C(25)	5848(6)	5139(4)	10104(4)	30(1)
C(26)	4917(7)	3622(3)	6509(5)	31(1)
C(27)	3746(7)	2994(4)	6291(5)	33(1)
C(28)	3846(8)	2507(4)	5330(5)	38(1)
C(29)	5408(8)	2169(5)	5553(7)	45(2)
C(30)	6602(9)	2795(5)	5852(7)	50(2)
C(31)	6485(7)	3276(4)	6796(6)	42(2)

C(33)	1829(6)	4561(4)	8510(5)	30(1)
C(34)	1263(7)	4545(4)	9479(5)	35(1)
C(35)	1270(7)	3737(5)	9949(5)	42(2)
C(36)	2603(7)	3275(4)	9895(5)	37(1)
C(37)	2589(8)	2759(4)	9070(6)	38(1)
C(38)	1261(8)	2620(5)	8067(6)	45(2)
C(39)	471(7)	3354(5)	7532(5)	41(2)
C(32)	1501(6)	4032(4)	7646(5)	34(1)

Table C47. Bond lengths [Å] and angles [°] for TS-032_a.

Rh(1)-C(33)	2.138(5)
Rh(1)-C(32)	2.158(5)
Rh(1)-P(2)	2.2165(14)
Rh(1)-C(37)	2.219(6)
Rh(1)-C(36)	2.238(6)
Rh(1)-Cl(1)	2.3737(14)
Al(1)-Cl(4)	2.120(2)
Al(1)-Cl(5)	2.133(3)
Al(1)-Cl(2)	2.142(2)
Al(1)-Cl(3)	2.144(2)
C(1)-C(2)	1.493(7)
C(1)-P(2)	1.676(6)
C(1)-P(1)	1.797(6)
P(1)-C(20)	1.792(6)
P(1)-C(14)	1.797(6)
P(1)-C(8)	1.802(6)
P(2)-C(26)	1.845(6)
C(2)-C(3)	1.397(8)
C(2)-C(7)	1.399(7)
C(4)-C(5)	1.381(9)
C(4)-C(3)	1.389(8)
C(5)-C(6)	1.384(9)
C(6)-C(7)	1.381(9)
C(8)-C(13)	1.386(9)
C(8)-C(9)	1.400(8)
C(9)-C(10)	1.386(9)
C(10)-C(11)	1.376(11)
C(11)-C(12)	1.389(11)
C(12)-C(13)	1.391(9)
C(15)-C(14)	1.391(9)
C(15)-C(16)	1.397(9)
C(14)-C(19)	1.402(8)
C(16)-C(17)	1.386(10)
C(17)-C(18)	1.384(11)
C(18)-C(19)	1.391(9)
C(20)-C(21)	1.397(8)
C(20)-C(25)	1.410(8)
C(21)-C(22)	1.387(8)
C(22)-C(23)	1.381(10)
C(23)-C(24)	1.371(10)
C(24)-C(25)	1.387(9)
C(26)-C(27)	1.520(8)
C(26)-C(31)	1.529(9)
C(27)-C(28)	1.528(9)
C(28)-C(29)	1.524(10)
C(29)-C(30)	1.530(11)
C(30)-C(31)	1.510(10)
C(33)-C(32)	1.407(9)
C(33)-C(34)	1.498(8)
C(34)-C(35)	1.539(11)
C(35)-C(36)	1.510(11)
C(36)-C(37)	1.391(10)
C(37)-C(38)	1.513(10)
C(38)-C(39)	1.538(11)
C(39)-C(32)	1.511(10)
C(33)-Rh(1)-C(32)	38.2(2)
C(33)-Rh(1)-P(2)	100.82(17)
C(32)-Rh(1)-P(2)	91.03(16)

C(33)-Rh(1)-C(37)	97.3(3)
C(32)-Rh(1)-C(37)	81.9(3)
P(2)-Rh(1)-C(37)	140.43(19)
C(33)-Rh(1)-C(36)	80.4(3)
C(32)-Rh(1)-C(36)	88.2(2)
P(2)-Rh(1)-C(36)	176.8(2)
C(37)-Rh(1)-C(36)	36.4(3)
C(33)-Rh(1)-Cl(1)	159.72(16)
C(32)-Rh(1)-Cl(1)	161.61(19)
P(2)-Rh(1)-Cl(1)	86.83(6)
C(37)-Rh(1)-Cl(1)	88.15(18)
C(36)-Rh(1)-Cl(1)	92.94(18)
Cl(4)-Al(1)-Cl(5)	110.59(11)
Cl(4)-Al(1)-Cl(2)	109.29(11)
Cl(5)-Al(1)-Cl(2)	109.01(12)
Cl(4)-Al(1)-Cl(3)	110.77(10)
Cl(5)-Al(1)-Cl(3)	109.36(11)
Cl(2)-Al(1)-Cl(3)	107.75(10)
C(2)-C(1)-P(2)	123.2(4)
C(2)-C(1)-P(1)	114.2(4)
P(2)-C(1)-P(1)	122.4(3)
C(20)-P(1)-C(14)	108.8(3)
C(20)-P(1)-C(1)	113.4(3)
C(14)-P(1)-C(1)	108.8(3)
C(20)-P(1)-C(8)	108.2(3)
C(14)-P(1)-C(8)	108.8(3)
C(1)-P(1)-C(8)	108.8(3)
C(1)-P(2)-C(26)	107.0(3)
C(1)-P(2)-Rh(1)	137.4(2)
C(26)-P(2)-Rh(1)	115.4(2)
C(3)-C(2)-C(7)	118.6(5)
C(3)-C(2)-C(1)	120.3(5)
C(7)-C(2)-C(1)	121.0(5)
C(5)-C(4)-C(3)	120.2(6)
C(4)-C(3)-C(2)	120.6(5)
C(4)-C(5)-C(6)	119.5(6)
C(7)-C(6)-C(5)	121.0(5)
C(6)-C(7)-C(2)	120.1(6)
C(13)-C(8)-C(9)	119.6(6)
C(13)-C(8)-P(1)	120.5(4)
C(9)-C(8)-P(1)	119.7(5)
C(10)-C(9)-C(8)	119.3(6)
C(11)-C(10)-C(9)	120.9(6)
C(10)-C(11)-C(12)	120.2(6)
C(11)-C(12)-C(13)	119.4(7)
C(8)-C(13)-C(12)	120.6(6)
C(14)-C(15)-C(16)	119.6(6)
C(15)-C(14)-C(19)	119.8(6)
C(15)-C(14)-P(1)	120.3(4)
C(19)-C(14)-P(1)	119.8(5)
C(17)-C(16)-C(15)	120.4(7)
C(18)-C(17)-C(16)	120.1(6)
C(17)-C(18)-C(19)	120.1(6)
C(18)-C(19)-C(14)	120.0(6)
C(21)-C(20)-C(25)	120.1(5)
C(21)-C(20)-P(1)	119.1(4)
C(25)-C(20)-P(1)	120.7(4)
C(22)-C(21)-C(20)	119.4(6)
C(23)-C(22)-C(21)	120.1(6)
C(24)-C(23)-C(22)	120.9(6)
C(23)-C(24)-C(25)	120.6(6)
C(24)-C(25)-C(20)	118.9(6)
C(27)-C(26)-C(31)	110.1(5)
C(27)-C(26)-P(2)	112.2(4)
C(31)-C(26)-P(2)	111.2(4)
C(26)-C(27)-C(28)	109.4(5)
C(29)-C(28)-C(27)	111.1(5)
C(28)-C(29)-C(30)	110.8(6)
C(31)-C(30)-C(29)	111.9(6)
C(30)-C(31)-C(26)	109.3(6)
C(32)-C(33)-C(34)	126.7(6)
C(32)-C(33)-Rh(1)	71.6(3)
C(34)-C(33)-Rh(1)	110.4(4)

C(33)-C(34)-C(35)	112.6(5)
C(36)-C(35)-C(34)	112.3(5)
C(37)-C(36)-C(35)	124.8(6)
C(37)-C(36)-Rh(1)	71.1(3)
C(35)-C(36)-Rh(1)	111.0(4)
C(36)-C(37)-C(38)	124.6(7)
C(36)-C(37)-Rh(1)	72.6(4)
C(38)-C(37)-Rh(1)	107.0(4)
C(37)-C(38)-C(39)	113.8(6)
C(32)-C(39)-C(38)	114.2(5)
C(33)-C(32)-C(39)	125.1(6)
C(33)-C(32)-Rh(1)	70.1(3)
C(39)-C(32)-Rh(1)	112.2(4)

Table C48. Anisotropic displacement parameters ($Å^2$x 10^3) for **YPCyRh(COD)ClAlCl$_4$**. The anisotropic displacement factor exponent takes the form: $-2p^2[h^2 a^{*2}U^{11} + ... + 2 h k a^* b^* U^{12}]$

	U^{11}	U^{22}	U^{33}	U^{23}	U^{13}	U^{12}
Rh(1)	26(1)	22(1)	30(1)	4(1)	6(1)	0(1)
Cl(1)	31(1)	25(1)	54(1)	5(1)	3(1)	4(1)
Al(1)	27(1)	33(1)	31(1)	-1(1)	9(1)	2(1)
C(1)	22(2)	20(3)	28(2)	1(2)	5(2)	0(2)
P(1)	26(1)	18(1)	25(1)	0(1)	6(1)	0(1)
P(2)	27(1)	19(1)	30(1)	-2(1)	9(1)	0(1)
Cl(2)	44(1)	74(1)	34(1)	4(1)	16(1)	14(1)
C(2)	28(2)	19(3)	27(2)	-3(2)	7(2)	6(2)
C(4)	31(3)	34(3)	37(3)	6(2)	13(2)	5(3)
Cl(4)	29(1)	60(1)	56(1)	-14(1)	6(1)	-2(1)
C(3)	30(3)	29(3)	32(3)	-1(2)	9(2)	2(2)
Cl(3)	30(1)	37(1)	43(1)	-2(1)	14(1)	4(1)
C(5)	43(3)	39(4)	39(3)	2(3)	20(3)	6(3)
Cl(5)	61(1)	31(1)	84(1)	6(1)	32(1)	4(1)
C(6)	40(3)	43(4)	24(2)	7(2)	7(2)	3(3)
C(7)	29(3)	27(3)	30(3)	0(2)	7(2)	2(2)
C(8)	32(3)	24(3)	28(2)	-1(2)	10(2)	-4(2)
C(9)	29(3)	25(3)	42(3)	3(2)	12(2)	-1(2)
C(10)	26(3)	41(4)	42(3)	9(3)	9(2)	3(3)
C(11)	33(3)	38(4)	46(3)	16(3)	11(2)	13(3)
C(12)	50(4)	27(3)	51(4)	3(3)	13(3)	14(3)
C(13)	45(3)	22(3)	38(3)	1(2)	0(3)	2(3)
C(15)	35(3)	27(3)	38(3)	1(2)	11(2)	-1(2)
C(14)	32(3)	21(3)	32(3)	-3(2)	9(2)	0(2)
C(16)	37(3)	32(4)	59(4)	-2(3)	23(3)	-6(3)
C(17)	29(3)	37(4)	69(4)	-12(3)	22(3)	-7(3)
C(18)	27(3)	48(4)	49(3)	-9(3)	6(3)	2(3)
C(19)	33(3)	27(3)	36(3)	-4(2)	11(2)	3(2)
C(20)	26(2)	21(3)	28(2)	-1(2)	5(2)	-2(2)
C(21)	31(3)	27(3)	33(3)	-2(2)	8(2)	0(2)
C(22)	33(3)	37(4)	34(3)	-8(2)	14(2)	-3(3)
C(23)	38(3)	52(4)	26(3)	2(2)	10(2)	-6(3)
C(24)	38(3)	42(4)	33(3)	7(3)	6(2)	2(3)
C(25)	29(3)	29(3)	30(3)	1(2)	4(2)	-1(2)
C(26)	31(3)	21(3)	42(3)	-7(2)	13(2)	-1(2)
C(27)	27(3)	24(3)	47(3)	-7(2)	10(2)	-2(2)
C(28)	44(3)	29(3)	40(3)	-9(2)	10(3)	-8(3)
C(29)	44(4)	34(4)	59(4)	-17(3)	19(3)	0(3)
C(30)	43(4)	37(4)	78(5)	-22(4)	29(3)	-6(3)
C(31)	30(3)	32(4)	64(4)	-11(3)	14(3)	-7(3)
C(33)	23(2)	24(3)	40(3)	4(2)	8(2)	12(2)
C(34)	29(3)	40(4)	39(3)	-9(2)	13(2)	5(3)
C(35)	40(3)	54(5)	36(3)	2(3)	17(2)	-8(3)
C(36)	40(3)	38(4)	35(3)	18(3)	15(2)	-8(3)
C(37)	40(3)	19(3)	53(3)	10(2)	14(3)	-7(3)
C(38)	43(4)	39(4)	56(4)	-5(3)	18(3)	-15(3)
C(39)	28(3)	53(5)	37(3)	-7(3)	1(2)	-5(3)
C(32)	24(2)	45(4)	30(2)	9(2)	2(2)	3(2)

4 Computational Studies

4.1 General remarks

All computational studies were carried out without symmetry restrictions. If it was not possible to obtain starting coordinates from crystal structures GaussView 6.0[10] were used. Calculations were performed with the Gaussian16 Revision B.01[11] or the Gaussian16 Revision C.01[12] program packages using Density-Functional Theory (DFT).[13] Energy optimizations were carried out with the PW6B95D3 functional[14] and def2svp basis set[15] together with GRIMMES D3 dispersion correction with Becke-Johnson damping.[16] To determine the nature of the structure harmonic vibrational frequency analyses were performed on the same level of theory.[17] No imaginary frequencies were observed for the ground states. Single point energies were calculated on PW6B95D3[14]/def2tzvp[15] level of theory.
Chemcraft 3D[18] was used for graphical representation.

4.2 Energies

Tabelle Fehler! Kein Text mit angegebener Formatvorlage im Dokument.. Energies of the Compounds.

Compound	E(SCF)	Corr(H)	Corr(G)	ΔG [hartree]	ΔG [kJ/mol]
**3^+**	-2957.52275614	0.802216	0.673697	–	–
**$^t3^+$**	-2957.46350122	0.801013	0.671928	0.057486	150.929283
**$4a^+$**	-1881.51980999	0.499651	0.408128	–	–
**$^t4a^+$**	-1881.47716491	0.498333	0.409037	0.043554	114.351237
**$4b^+$**	-1885.14597216	0.570444	0.476616	–	–
**$^t4b^+$**	-1885.08917626	0.569155	0.475138	0.055318	145.237146
**NHP**	-608.62326436	0.133493	0.093455	–	–
**^{t}NHP**	-608.52783290	0.131419	0.087334	0.089310	234.484613

^{t} refers to the triplet state.

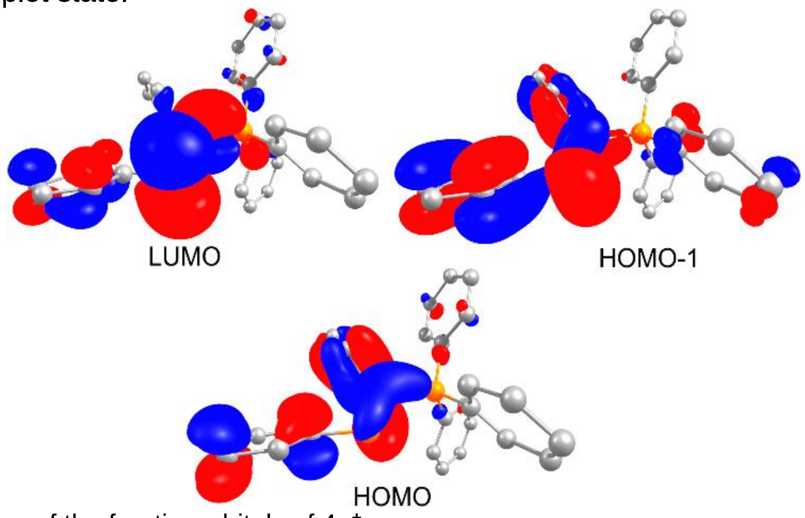

Figure S65. display of the frontier orbitals of $4a^+$.

4.3 Natural Charges and WBIs

Tabelle D1. Natural Charges and Wiberg-Bond-Indices.

		**2^+**	$4a^+$	$4b^+$	NHP
**Natural Charges**	$P^{Phosphenium}$	0.75766	0.82837	0.83949	1.12327
	C^{Ylide}	-0.91140/<br>-0.91922	-0.80653	-0.80197	–
	$P^{Phosphonium}$	1.59873/<br>1.58992	1.59573	1.59680	–
**WBI**	C^{Ylide}–$P^{Phosphenium}$/<br>C^{R}–$P^{Phosphenium}$<br>or N–P	1.3058/<br>1.3069	1.6173/<br>(1.0031)	1.6808/<br>(0.9274)	1.0201/<br>1.0202

4.4 Coordinates

2^+

E = -2957.52275614
P -0.025380 -0.439366 -0.068975
P -2.913641 -0.332729 0.008344
P 2.878412 -0.485862 -0.020260
C -1.420402 0.565871 -0.231666
C -1.514555 2.020040 -0.470107
C -2.366273 2.857105 0.264390
H -2.976377 2.446347 1.064391
C -2.426268 4.220955 0.002667
H -3.092146 4.849988 0.590199
C -1.634118 4.782258 -0.991601
H -1.675809 5.851111 -1.189566
C -0.787333 3.963371 -1.733837
H -0.160499 4.390473 -2.514012
C -0.735485 2.601010 -1.481901
H -0.072762 1.963172 -2.064740
C -3.662858 0.049919 1.617488
C -5.036083 0.226334 1.790138
H -5.712085 0.157961 0.941358
C -5.540892 0.510768 3.054804
H -6.611050 0.654332 3.186869
C -4.682905 0.617942 4.143581
H -5.082932 0.843257 5.129975
C -3.311214 0.449294 3.971751
H -2.638339 0.544473 4.821269
C -2.799207 0.173083 2.711471
H -1.724423 0.066240 2.564399
C -2.577113 -2.111130 -0.003383
C -2.612065 -2.879976 1.158810
H -2.887940 -2.429282 2.109099
C -2.293903 -4.233152 1.098429
H -2.325502 -4.834355 2.004615
C -1.940080 -4.813656 -0.113309
H -1.689119 -5.871629 -0.156230
C -1.917201 -4.047631 -1.276860
H -1.648973 -4.504476 -2.226886
C -2.239100 -2.700322 -1.226410
H -2.219897 -2.098462 -2.133902
C -4.161575 -0.079401 -1.275928
C -4.092032 0.970645 -2.194117
H -3.275115 1.684524 -2.156414

C -5.076085 1.097385 -3.168774
H -5.018284 1.915612 -3.883131
C -6.124385 0.186850 -3.233313
H -6.890576 0.292847 -3.998547
C -6.190516 -0.866587 -2.325624
H -7.002840 -1.587917 -2.381225
C -5.210978 -1.004565 -1.352178
H -5.254185 -1.841829 -0.656578
C 1.417043 0.486071 0.131238
C 1.587753 1.935866 0.349675
C 0.883592 2.573052 1.379251
H 0.227550 1.979021 2.013937
C 0.991864 3.941785 1.576378
H 0.424278 4.417724 2.373481
C 1.822204 4.705407 0.760293
H 1.907800 5.778402 0.917998
C 2.537422 4.087523 -0.258577
H 3.183225 4.675930 -0.907593
C 2.415257 2.718808 -0.465317
H 2.943787 2.254010 -1.296380
C 4.190149 0.118038 1.074529
C 4.306858 -0.372056 2.377718
H 3.662892 -1.174867 2.725675
C 5.264808 0.153194 3.235708
H 5.353761 -0.238364 4.246588
C 6.109491 1.169299 2.802191
H 6.858553 1.578842 3.476661
C 6.002497 1.655623 1.503956
H 6.664898 2.446217 1.158718
C 5.049005 1.133080 0.640105
H 4.981228 1.512774 -0.375783
C 2.524134 -2.218547 0.381592
C 2.928522 -3.251366 -0.464328
H 3.453145 -3.030874 -1.390464
C 2.647484 -4.572455 -0.129810
H 2.961736 -5.373075 -0.795744
C 1.967229 -4.866835 1.044939
H 1.750312 -5.901441 1.303090
C 1.559678 -3.838423 1.890966
H 1.017569 -4.065100 2.806412
C 1.829275 -2.519730 1.559993
H 1.479165 -1.714669 2.203296
C 3.574405 -0.468195 -1.694403
C 4.904082 -0.836807 -1.925135
H 5.553342 -1.097918 -1.090813
C 5.396915 -0.862446 -3.223431
H 6.431534 -1.146292 -3.403465
C 4.569939 -0.521096 -4.290712
H 4.961589 -0.537064 -5.305727
C 3.247612 -0.155086 -4.062853
H 2.603962 0.115400 -4.897003
C 2.747401 -0.128850 -2.766035
H 1.715700 0.164740 -2.575163

^{t}2^{+} (triplet)

E = -2957.46350122
P -0.031094 -0.907325 -0.585560
P -2.886615 -0.363712 -0.010763
P 2.890922 -0.426859 -0.160892
C -1.411192 0.250588 -0.765286

C -1.283824 1.567438 -1.346088
C -1.942543 2.695536 -0.812455
H -2.599995 2.575252 0.046408
C -1.706230 3.960292 -1.319335
H -2.200830 4.819265 -0.870855
C -0.825091 4.140319 -2.386195
H -0.645428 5.137155 -2.783015
C -0.179306 3.038201 -2.939061
H 0.501108 3.169326 -3.778206
C -0.395796 1.770631 -2.422824
H 0.108928 0.907280 -2.849042
C -2.718854 -0.335321 1.784985
C -3.097184 0.793558 2.517058
H -3.636928 1.607248 2.035663
C -2.776181 0.882266 3.865681
H -3.081750 1.756178 4.436848
C -2.057764 -0.139526 4.480681
H -1.803243 -0.064054 5.535772
C -1.669629 -1.258655 3.750593
H -1.115531 -2.059873 4.234887
C -1.995969 -1.363594 2.403304
H -1.680172 -2.232124 1.828617
C -3.140934 -2.084698 -0.504131
C -3.917251 -2.920769 0.303360
H -4.289647 -2.567773 1.263785
C -4.212378 -4.209434 -0.120969
H -4.811663 -4.861927 0.510135
C -3.744089 -4.662266 -1.350909
H -3.976110 -5.672804 -1.680609
C -2.983166 -3.825612 -2.160743
H -2.619756 -4.179166 -3.123013
C -2.682059 -2.535181 -1.743941
H -2.082371 -1.882542 -2.375177
C -4.393491 0.496889 -0.506947
C -4.491445 1.018975 -1.800662
H -3.635447 0.994604 -2.470597
C -5.689288 1.574836 -2.229611
H -5.761268 1.987088 -3.233514
C -6.791799 1.603093 -1.381145
H -7.727622 2.041445 -1.721393
C -6.703112 1.062986 -0.102570
H -7.568239 1.071654 0.556660
C -5.509170 0.504088 0.335899
H -5.452514 0.068424 1.331441
C 1.270166 0.269095 -0.159987
C 1.053744 1.535915 0.555193
C 0.211147 1.585427 1.675485
H -0.233058 0.662120 2.040610
C -0.046234 2.788598 2.320840
H -0.698172 2.800450 3.191613
C 0.530455 3.967922 1.863503
H 0.325060 4.910678 2.366271
C 1.377031 3.933056 0.758003
H 1.824268 4.851707 0.383342
C 1.638085 2.733409 0.114356
H 2.261305 2.721911 -0.776801
C 4.057072 0.707194 0.621915
C 4.154758 0.745318 2.014223
H 3.609921 0.027874 2.623602
C 4.953566 1.703657 2.624378
H 5.028241 1.731659 3.709064
C 5.656836 2.621497 1.851108

H 6.282919 3.369851 2.332516
C 5.564944 2.582069 0.463704
H 6.116742 3.297781 -0.141690
C 4.764007 1.630291 -0.153885
H 4.686118 1.610202 -1.239135
C 2.850424 -1.962953 0.806961
C 3.611041 -3.082364 0.466578
H 4.237445 -3.074240 -0.422025
C 3.554593 -4.223116 1.259284
H 4.144969 -5.095832 0.989138
C 2.743953 -4.250418 2.388802
H 2.703641 -5.145250 3.006415
C 1.977873 -3.138375 2.725015
H 1.339590 -3.162298 3.605682
C 2.019739 -1.998306 1.933829
H 1.398734 -1.138062 2.181090
C 3.546619 -0.814011 -1.799572
C 4.918405 -1.051051 -1.951466
H 5.586285 -0.980289 -1.094040
C 5.432022 -1.360604 -3.204181
H 6.498285 -1.541639 -3.320854
C 4.584722 -1.431754 -4.306394
H 4.990340 -1.672000 -5.287014
C 3.222938 -1.192635 -4.157123
H 2.561427 -1.246667 -5.018951
C 2.700224 -0.883239 -2.906935
H 1.633927 -0.709749 -2.779315

***4a*$^{+}$**

E = -1881.51980999
P 1.110613 0.237679 -0.013516
P -1.552762 1.557494 0.059160
C -0.687028 0.108897 0.004865
C -1.184030 -1.285855 -0.055127
C -1.706784 -1.777658 -1.254688
H -1.766532 -1.120900 -2.120119
C -2.141100 -3.094256 -1.342447
H -2.545253 -3.466367 -2.281462
C -2.058654 -3.934046 -0.236327
H -2.399317 -4.964771 -0.307070
C -1.544830 -3.450252 0.963232
H -1.487339 -4.099728 1.834245
C -1.106444 -2.134163 1.056190
H -0.711077 -1.758405 1.997397
C -3.312853 1.166777 0.114647
C -4.115429 2.196639 -0.404807
H -3.647113 3.099021 -0.798432
C -5.498422 2.076198 -0.425901
H -6.107093 2.874215 -0.845038
C -6.098569 0.940251 0.105302
H -7.182641 0.846377 0.102031
C -5.315274 -0.072106 0.657136
H -5.788843 -0.950890 1.089500
C -3.933001 0.031604 0.660521
H -3.342022 -0.764507 1.100910
C 1.626844 1.925580 -0.360039
C 1.170316 2.562442 -1.522344
H 0.489432 2.049482 -2.199680
C 1.585619 3.854069 -1.807581
H 1.231167 4.348866 -2.708913

C 2.450015 4.516131 -0.938319
H 2.770662 5.531139 -1.163249
C 2.903361 3.886047 0.214455
H 3.577936 4.404262 0.891999
C 2.496447 2.589286 0.507818
H 2.854680 2.099607 1.410271
C 1.746257 -0.238289 1.603934
C 1.085029 0.225544 2.746412
H 0.171094 0.810287 2.650279
C 1.595276 -0.068249 4.003149
H 1.081778 0.289572 4.892688
C 2.760571 -0.822103 4.122410
H 3.155892 -1.055011 5.108900
C 3.420825 -1.278197 2.986541
H 4.332045 -1.864261 3.082285
C 2.919781 -0.986549 1.722721
H 3.440624 -1.340491 0.835793
C 1.802305 -0.877836 -1.250704
C 2.487040 -0.377946 -2.362422
H 2.637577 0.690972 -2.486482
C 2.995805 -1.255537 -3.312782
H 3.529523 -0.862162 -4.174872
C 2.829544 -2.626742 -3.158096
H 3.229032 -3.309853 -3.904736
C 2.163086 -3.128210 -2.043423
H 2.041896 -4.201220 -1.913956
C 1.651354 -2.262540 -1.087953
H 1.143762 -2.666416 -0.215166

^{t}4a^{+} (triplet)

E = -1881.47716491
P 0.663935 0.573960 0.096888
P -1.280305 -0.282882 -2.044099
C 0.099487 -0.759529 -0.957683
C 0.758863 -2.029426 -1.028890
C 0.085392 -3.126429 -1.625426
H -0.920722 -2.978488 -2.016689
C 0.676957 -4.371755 -1.702921
H 0.135524 -5.198834 -2.156506
C 1.966764 -4.568208 -1.206231
H 2.434206 -5.548095 -1.273971
C 2.659693 -3.501286 -0.632625
H 3.671192 -3.647117 -0.260264
C 2.071447 -2.254235 -0.539902
H 2.639146 -1.434538 -0.105441
C -2.717646 -0.633901 -1.013228
C -3.897334 0.062309 -1.325658
H -3.914095 0.738261 -2.180090
C -5.030096 -0.085110 -0.537187
H -5.934436 0.468505 -0.781040
C -5.010344 -0.941601 0.559953
H -5.900104 -1.058848 1.175044
C -3.855491 -1.662884 0.860994
H -3.848091 -2.349370 1.705394
C -2.717273 -1.511328 0.084345
H -1.823126 -2.089431 0.312758
C -0.757485 1.630266 0.431981
C -1.110655 2.615119 -0.498463
H -0.466984 2.824908 -1.350475
C -2.292559 3.324826 -0.331101

H -2.564890 4.094635 -1.049511
C -3.124581 3.048203 0.749561
H -4.053008 3.601683 0.873688
C -2.772924 2.070207 1.674424
H -3.423198 1.856077 2.519333
C -1.587263 1.362187 1.522773
H -1.316785 0.600649 2.250142
C 1.299158 -0.066800 1.659153
C 0.658771 -1.154996 2.262185
H -0.193077 -1.627023 1.776401
C 1.123915 -1.640843 3.476186
H 0.628129 -2.488386 3.943873
C 2.227203 -1.050571 4.086818
H 2.592599 -1.437267 5.035753
C 2.867858 0.026339 3.484072
H 3.733858 0.482650 3.958094
C 2.409045 0.521625 2.269036
H 2.924054 1.354705 1.796574
C 1.918356 1.574697 -0.716610
C 2.174326 2.869166 -0.247354
H 1.595030 3.280172 0.578467
C 3.162542 3.633658 -0.851296
H 3.363839 4.639801 -0.490467
C 3.887492 3.115530 -1.922079
H 4.657942 3.720060 -2.395993
C 3.624461 1.834536 -2.395345
H 4.183881 1.437638 -3.239284
C 2.638724 1.058616 -1.796530
H 2.423854 0.061195 -2.175359

4b $^{+}$

E = -1885.14575903
P 1.226241 0.281500 -0.044528
P -1.559030 1.375073 0.032164
C -0.556133 0.021777 -0.001005
C -0.959837 -1.402886 0.080575
C -1.267482 -2.110448 -1.085915
H -1.222271 -1.602976 -2.047760
C -1.608524 -3.455773 -1.020726
H -1.847176 -3.996006 -1.934362
C -1.638788 -4.110676 0.206421
H -1.903624 -5.164637 0.255758
C -1.334707 -3.413400 1.371455
H -1.364240 -3.919374 2.334156
C -0.996303 -2.066109 1.312411
H -0.758810 -1.521886 2.224321
C 1.604935 1.955106 -0.577182
C 1.059141 2.438462 -1.774101
H 0.388165 1.816520 -2.364348
C 1.373526 3.718683 -2.204612
H 0.949855 4.095871 -3.132559
C 2.224909 4.519530 -1.446580
H 2.465845 5.524821 -1.785663
C 2.765924 4.041031 -0.258907
H 3.429172 4.668462 0.331942
C 2.460601 2.757922 0.180183
H 2.885222 2.386328 1.109980
C 1.881101 0.031649 1.616067
C 1.175060 0.549317 2.707046
H 0.214635 1.039392 2.554771

C 1.702328 0.433971 3.985811
H 1.153346 0.834332 4.835104
C 2.929948 -0.194543 4.178753
H 3.338953 -0.287332 5.182629
C 3.635591 -0.703288 3.093569
H 4.595970 -1.190578 3.245752
C 3.117498 -0.590624 1.808476
H 3.674529 -0.985261 0.961702
C 2.007264 -0.912047 -1.144265
C 2.635345 -0.499208 -2.322321
H 2.685635 0.554942 -2.582892
C 3.214367 -1.444094 -3.161762
H 3.704965 -1.120852 -4.076965
C 3.172721 -2.793021 -2.828635
H 3.628128 -3.528812 -3.488144
C 2.558493 -3.204965 -1.648702
H 2.532967 -4.259339 -1.383331
C 1.976523 -2.271865 -0.803366
H 1.504438 -2.600471 0.120832
C -3.256439 0.656848 0.172982
C -4.113344 1.549248 1.077188
C -3.875221 0.533087 -1.226803
H -3.185402 -0.346573 0.614357
C -5.546059 1.035353 1.148617
H -4.111888 2.576563 0.680527
H -3.677508 1.603627 2.083801
C -5.311179 0.028654 -1.140383
H -3.861145 1.517122 -1.721585
H -3.270385 -0.141289 -1.845991
C -6.161853 0.910359 -0.237430
H -6.146085 1.701551 1.780417
H -5.550574 0.051332 1.640970
H -5.742645 -0.025330 -2.147540
H -5.302749 -0.999047 -0.747740
H -7.180725 0.511001 -0.167277
H -6.251197 1.911013 -0.686556

^{t}4b^{+} (triplet)

E = -1885.08917626
P 0.998947 0.386171 -0.015327
C -0.137785 -0.798272 0.672028
P -1.414047 0.008111 1.689724
C -0.093560 -2.229690 0.500020
C 0.389945 -2.852540 -0.673857
H 0.752088 -2.248931 -1.500462
C 0.358413 -4.229884 -0.810134
H 0.719500 -4.684476 -1.730079
C -0.141517 -5.031687 0.213996
H -0.156533 -6.113670 0.103289
C -0.633534 -4.439138 1.376668
H -1.028072 -5.057804 2.179685
C -0.620990 -3.063667 1.515588
H -1.000598 -2.607431 2.428888
C -2.842267 0.001102 0.473614
H -2.449060 -0.267083 -0.515206
C -3.485511 1.384968 0.399242
H -3.808249 1.688955 1.406918
H -2.747837 2.126782 0.069229
C -4.685400 1.369701 -0.540161
H -5.150141 2.363357 -0.564219

H -4.333423 1.165114 -1.563333
C -5.699446 0.310013 -0.133319
H -6.122865 0.572499 0.847683
H -6.539525 0.294374 -0.838259
C -5.055112 -1.066296 -0.049766
H -4.722142 -1.376831 -1.051649
H -5.783667 -1.816510 0.281776
C -3.856606 -1.068733 0.894799
H -4.195190 -0.863298 1.921511
H -3.382657 -2.058952 0.909399
C 2.284752 -0.309673 -1.069279
C 3.189923 -1.194851 -0.470664
H 3.101170 -1.441545 0.586785
C 4.201641 -1.759537 -1.231643
H 4.903971 -2.449843 -0.770042
C 4.319795 -1.438583 -2.582684
H 5.116916 -1.881105 -3.176305
C 3.430154 -0.548176 -3.172467
H 3.532406 -0.288498 -4.223676
C 2.407386 0.020380 -2.419712
H 1.720648 0.723462 -2.884530
C 1.899743 1.215760 1.311358
C 2.860623 2.162850 0.933724
H 3.025394 2.391402 -0.118965
C 3.611789 2.806725 1.905657
H 4.353368 3.546740 1.613412
C 3.422296 2.496909 3.250819
H 4.016190 2.999919 4.010979
C 2.485602 1.540593 3.623839
H 2.346939 1.290170 4.672996
C 1.720784 0.895703 2.657523
H 0.995776 0.143720 2.958574
C 0.031363 1.592915 -0.942246
C -0.020812 2.940665 -0.581436
H 0.546632 3.305751 0.271099
C -0.816729 3.816173 -1.313327
H -0.859748 4.865903 -1.032235
C -1.558342 3.351449 -2.393552
H -2.180011 4.040430 -2.961301
C -1.514709 2.004360 -2.748440
H -2.100780 1.640806 -3.589527
C -0.725529 1.123108 -2.023804
H -0.702157 0.066485 -2.292683

NHP

E = -608.623264361
C -0.683744 1.331733 -0.000013
C 0.683665 1.331779 0.000008
N -1.180148 0.067211 -0.000029
H -1.343480 2.194026 -0.000012
H 1.343412 2.194060 0.000022
C -2.616101 -0.215684 0.000017
H -2.774615 -1.297173 0.000370
H -3.075948 0.213158 0.895348
H -3.075893 0.212561 -0.895630
C 2.616071 -0.215634 -0.000001
H 3.075914 0.213018 -0.895422
H 3.075883 0.212809 0.895540
H 2.774606 -1.297125 -0.000116
N 1.180119 0.067257 0.000013

P 0.000065 -1.131986 -0.000004

NHP (triplet)

E = -608.527832901
C -0.700156 1.325837 -0.000008
C 0.700081 1.325841 -0.000193
N -1.244105 0.106187 0.000413
H -1.319845 2.220436 -0.000184
H 1.319850 2.220377 -0.000134
C -2.662177 -0.190686 0.000041
H -2.933004 -0.769103 0.892260
H -3.227687 0.745009 0.000287
H -2.932743 -0.768562 -0.892625
C 2.662182 -0.190606 0.000280
H 3.227528 0.745185 -0.000569
H 2.932784 -0.767788 0.893371
H 2.933101 -0.769581 -0.891543
N 1.244028 0.106114 -0.000305
P 0.000064 -1.197627 -0.000156

4.5 Exemplary Input Files

Input File for Optimization, Frequenzy Calculation and Single Point Calculation

```
%mem=56000MB
%nprocs=16
%chk=22.chk
#p opt freq pw6b95d3 genecp

title

1 1
 C     -0.46174049    0.46146264    0.00000900
 C      0.94000951    0.46146264    0.00000900
 N     -0.90146749    1.78197064    0.00000000
 H     -1.16332849   -0.37177036    0.00010700
 H      1.58438251   -0.41124336    0.00003400
 C     -2.30588651    2.21614469   -0.00032410
 H     -2.38774492    3.15930939    0.49831360
 H     -2.64836428    2.31516090   -1.00918721
 H     -2.90381411    1.48999506    0.50966542
 C      2.81847871    2.33833523   -0.00002649
 H      3.15951016    2.44361881    1.00869196
 H      3.44032716    1.63643993   -0.51533949
 H      2.86617252    3.28657149   -0.49345662
 N      1.36400751    1.83225864    0.00000900
 P      0.20695051    2.62371164   -0.00009500

C N H P 0
def2svp
****



--Link1--
%chk=22_SP.chk
%oldchk=22.chk
%nprocs=16
%mem=32000MB
```

Supporting Information

```
#p pw6b95d3 genecp guess=read geom=allcheck

C N H P 0
def2tzvp
****
```

Input File for NBO-Analysis

```
%mem=56000MB
%nprocs=16
%chk=22_nbo.chk
#p pw6b95d3 genecp pop=(savenbo,nbo7read) guess=read geom=allcheck

C N H P 0
def2svp
****


$nbo bndidx $end
```

5 References

[1] M. Shi, B. Xu, *J. Org. Chem.*, **2002**, *67*, 1, 294-297.
[2] K. Coetzee, C. E. Strasser, S. Cronje, H. G. Raubenheimer, *Z. Naturforsch., B: J. Chem. Sci.*, **2009**, *64*, 1449.
[3] a) P. Chung Choi, J. H. Morris, *J.Chem. Soc., Dalton Trans.*, **1984**, 2119-2125 b) G. Silveira-Dorta, S. J. Álvarez-Méndez, V. S. Martín, J. M. Padrón, *Beilstein J. Org. Chem.*, **2016**, *12*, 957–962.
[4] a) A. S. Batsanov, M. G. Davidson, J. A. K. Howard, S. Lamb, C. Lustig, Chem. Commun., 1996,1791-1792, b) T. A. Albright, M. D. Gordon, W. J. Freeman, E. E. Schweizer, J. Am. Chem. Soc., 1976, 98, 20, 6249-6252, c) A. A. Skatova, I. L. Fedushkin, O. V. Maslova, M. Hummert, H. Schumann, *Russian Chemical Bulletin*, **2007**, *56*, 2284–228.
[5] H. J. Bestmann, A. Bomhard, R. Dostalek, R. Pichl, R. Riemer, R. Zimmermann, *Synthesis*, **1992**, *8*, 787-792.
[6] A. Schmidpeter, G. Jochem, C. Klinger, C. Robl, H. Nöth, *J. Organomet. Chem.*, **1997**, *529*, 87-102.
[7] G. Jochem, F. Breitsameter, A. Schier, A. Schmidpeter, *Heteroat. Chem.*, **1996**, *7*, 4, 239-247.
[8] Sheldrick, G. M. A short history of SHELX. Acta Cryst. 2008, A64, 112–122.
[9] Sheldrick, G. M. Crystal structure refinement with SHELXL. Acta Cryst. 2015, C71, 3.
[11] R. Dennington, T. A. Keith, J. M. Millam, *GaussView, Version 6.0*, Semichem Inc., Shawnee Mission, 2016.
[11] M. J. Frisch, G. W. Trucks, H. B. Schlegel, G. E. Scuseria, M. A. Robb, J. R. Cheeseman, G. Scalmani, V. Barone, G. A. Petersson, H. Nakatsuji et al., *Gaussian 16, Revision B.01*, Gaussian, Inc., Wallingford CT, **2016**.
[12] M. J. Frisch, G. W. Trucks, H. B. Schlegel, G. E. Scuseria, M. A. Robb, J. R. Cheeseman, G. Scalmani, V. Barone, G. A. Petersson, H. Nakatsuji et al., *Gaussian 16, Revision C.01*, Gaussian, Inc., Wallingford CT, **2016**.
[13] a) P. Hohenberg, W. Kohn, *Phys. Rev.* **1964**, *136*, B864-B871; b) W. Kohn, L. J. Sham, *Phys. Rev.* **1965**, *140*, A1133-A1138.
[14] Y. Zhao, D. G. Truhlar, *J. Phys. Chem. A* **2005**, *109*, 5656.
[15] F. Weigend, R. Ahlrichs, *Phys. Chem. Chem. Phys.* **2005**, *7*, 3297.
[16] [17] a) S. Grimme, J. Antony, S. Ehrlich, H. Krieg, *J. Chem. Phys.* **2010**, *132*, 154104; b) S. Grimme, S. Ehrlich, L. Goerigk, *J. Comput. Chem.* **2011**, *32*, 1456; c) D. G. A. Smith, L. A. Burns, K. Patkowski, C. D. Sherrill, *J. Phys. Chem. Lett.* **2016**, *7*, 2197.
[17] P. Deglmann, F. Furche, *J. Am. Chem. Soc.* **2002**, *117*, 9535.
[18] *Chemcraft - graphical software for visualization of quantum chemistry computations*.
